# Visible-Light-Promoted Synthesis of 1,6-Imino Alcohols by Metal-Free 1,2-Carboimination of Alkenes

**DOI:** 10.1021/acs.orglett.5c00082

**Published:** 2025-03-03

**Authors:** María J. Cabrera-Afonso, Aida Jaafar, Christian Cristóbal, Javier Adrio, Maria Ribagorda

**Affiliations:** † Departamento de Química Orgánica, Facultad de Ciencias, 16722 Universidad Autónoma de Madrid, 28049 Madrid, Spain; ‡ Institute for Advanced Research in Chemical Sciences (IAdChem), Universidad Autónoma de Madrid, 28049 Madrid, Spain; § Center for Innovation in Advanced Chemistry (ORFEO-CINQA), Universidad Autónoma de Madrid, 28049 Madrid, Spain

## Abstract

We report a metal-free synthesis of highly functionalized 1,6-amino alcohols through a visible-light 1,2-carboimination of alkenes and bifunctional starting materials prepared from commercially available alcohols. This protocol orchestrates the generation of up to four different types of radicals, which are efficiently recombined to yield 1,6-iminyl alcohols. The methodology demonstrated a broad functional group tolerance and was validated by the late-stage installation of the 1,6-amino alcohol motif in biomolecules and pharmaceuticals and the scale-up of the process. The versatility of the products was highlighted by their conversion into a variety of useful intermediates for target-directed synthesis.

## Graphical abstract

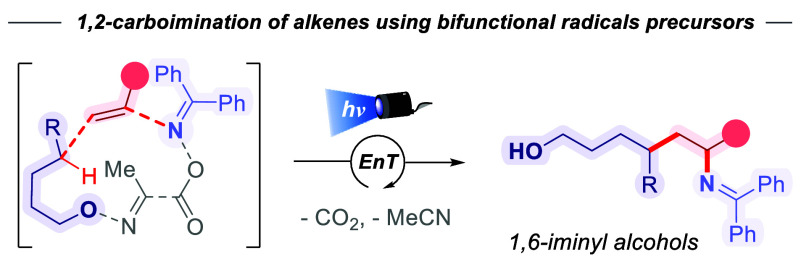

1,6-Amino-oxygenated compounds are versatile synthetic building blocks in organic chemistry, embedded in the backbone of pharmaceuticals, such as salmeterol or abediterol to treat pulmonary diseases, and other highly elaborated structures such as ALC-0315, described in the formulation of Pfizer’s coronavirus vaccine. Despite the synthetic relevance of the 1,6-amino alcohol motif, only a few methods described the synthesis of *N*-substituted 6-aminohexanols. Typically, these protocols involved the hydrogenation of caprolactams^1^ or classical reductions of 6-azidohexanols^2^ or 6-aminohexanoic acids.^3^ These methods are often limited in scope, requiring harsh conditions, which restrict their broader applicability. Therefore, the direct formation of highly substituted 1,6-amino alcohols is highly desirable. On the contrary, alkenes are highly versatile and readily accessible building blocks ideal for the flexible assembly of densely functionalized molecules with significant structural complexity and diversity. Direct 1,2-carboamination of olefins has emerged as one of the most attractive and practical approaches within the alkene difunctionalization framework, enabling the subsequent formation of C–C and C–N bonds.^4^ However, traditional 1,2-carboaminations required the use of transition metal catalysts and high temperatures, limiting the substrate scope. Recently, various visible-light conditions and 1,2-carboamination strategies have emerged, overcoming some of the previous limitations by the employment of softer conditions and presenting a broad functional group tolerance. Photochemical 1,2-carboaminations typically preceded through radical-polar crossover (RPC),^5^ a ligand-to-metal charge transfer (LMCT),^6^ or an energy transfer (EnT)^7–9^ mechanism.

Notably, EnT reactions enabled the formation of multiple bonds from commodity reagents under simple protocols using visible-light irradiation without considering redox potentials or the use of external additives. In particular, EnT 1,2-carboiminations of alkenes from *N*-oxime-type bifunctional reagents proceed through homolytic σ-bond fragmentation upon light irradiation, generating two radical species: a more reactive transient *C*-centered radical and a persistent iminyl radical (Figure 1). These radicals then react with an alkene in a one-pot process, regioselectively forming both C–C and C–N bonds. In 2020, the group of Glorius related the use of oxime esters of alkyl carboxylic acids to accomplish an intermolecular 1,2-carboimination of activated alkenes (Figure 1A.1).^7a^ The same group also described an intermolecular amino-carbonylation for the synthesis of β-amino acids by the use of bifunctional oxime oxalate ester (Figure 1A.2).^7b^ Later, Molander’s group^7c^ discovered a vicinal imino-trifluoromethylation of alkenes using oxime esters of trifluoroacetic acid (Figure 1A.3). Despite this, the potential use of an alkyl radical generated from alkoxy radicals by 1,5-HAT for the 1,2-carboimination of alkenes remains underexplored. The use of these transient radicals would enable the selective introduction of unprotected alkyl alcohol motifs, which are present in numerous biomolecules and pharmaceuticals across a wide variety of alkenes.

**Figure 1:**
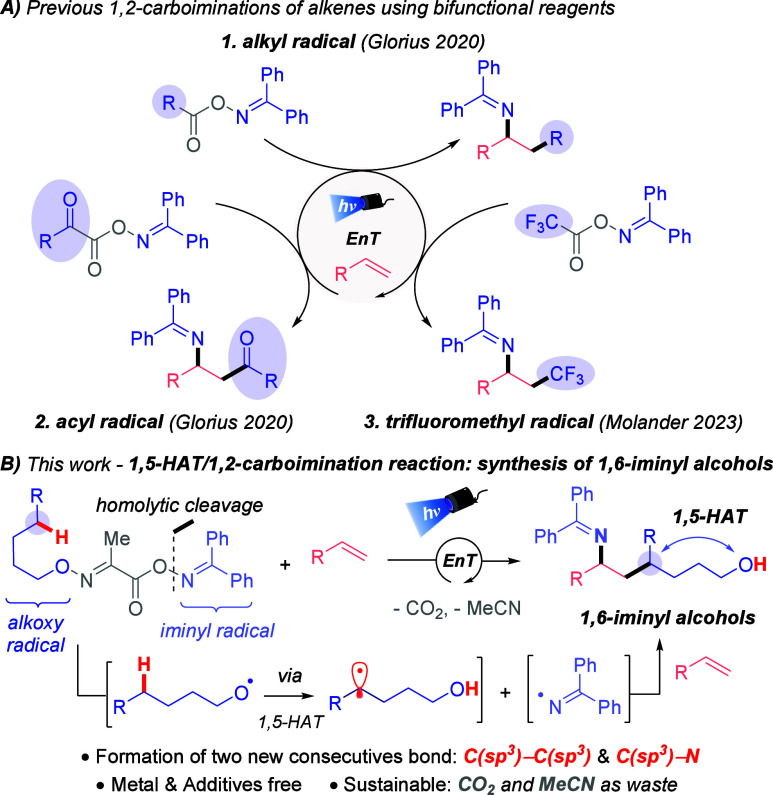
Synthesis strategies of 1,2-carboaminations of alkenes using bifunctional reagents via EnT.

Recently, we have described the synthesis of δ-amino alcohols via an EnT photocatalysis protocol from *N*-oxime-type bifunctional reagents.^10^ This protocol facilitates the synthesis of 1,4-imino alcohols from prefunctionalized alcohols without additives, generating carbon dioxide and acetonitrile as subproducts. These bifunctional reagents provided access to the corresponding alkoxy radicals, which, to date, were not accessible from typical oxime esters.^11^ Moreover, the group of Xu reported the use of a similar bifunctional reagent to perform a 1,2-diamination of alkenes.^12^ Building on our previous studies, we envisioned that this bifunctional reagent, in conjunction with commercially available alkenes, could effectively enable the preparation of 1,6-imino alcohols through the orchestrated generation and coupling of iminyl and alkyl radical intermediates (Figure 1B). This method could allow the straightforward preparation of 1,6-imino alcohols via the subsequent formation of two new bonds, C­(sp^3^)–C­(sp^3^) and C­(sp^3^)–N, in a one-step process that embrace the homolysis of the O–N bond, carbon dioxide and acetonitrile extrusion, 1,5-hydrogen atom transfer (HAT), and Giese-type and radical additions.^13,14^

To study the feasibility of the proposed 1,2-carboimination, bifunctional reagent **2a** and methyl acrylate (**1a**) were used as model substrates using 5CzBN as the organophotocatalyst.^15^ After a preliminary screening of the reaction conditions, the best results were obtained using 2.0 equiv of **2a**, 5CzBN (1 mol %), acetone (0.05 M) as a solvent, and irradiation for 2 h (blue Kessil lamp; λ_max_ = 427 nm), leading to expected 1,6-imino alcohol **3a** in 39% yield.^15,16^ Gratifyingly, changing methyl acrylate **1a** to acrylonitrile **1b** improved the yield to 90%. Further control experiments conducted in the absence of the photocatalyst or light irradiation confirmed the essential role of each component.^15^ Having established the optimal set of conditions, we investigated the scope of the protocol using various substituted alkenes, including esters, amides, and substituted styrenes (Scheme 1). In all cases, the desired 1,6-imino alcohols were obtained in good to excellent yields. The reaction using dimethyl fumarate afforded the desired compound **3c** in 60% yield. α-Methyl (**1d**) or α-phenyl (**1f**) acrylates were amenable to this protocol, generating **3d** or **3f**, respectively, in good to high yields. These examples showcased that α- or β-alkene substitution did not affect the reactivity but played a key role in stabilizing the radical intermediates. Specifically, the stabilization of the C­(sp^3^) radical intermediate formed at the alkene explains the higher yield observed for **3f** (98%), which is attributed to the highly stabilized benzylic, tertiary, α-carbonyl C­(sp^3^) radical, thereby enhancing the yield of the final product. *n*-Penthyl vinyl ketone **1g** and vinyl amide **1h** afforded 1,6-imino alcohols **3g** and **3h** in 61% and 51% yields, respectively. Versatile Weinreb amide derivative **1i** gives rise to the desired product **3i** in 51% yield. More common alkenes, such as styrenes, were successfully used, affording the corresponding 1,6-iminyl alcohols **3j**–**3n** in excellent to good yields. The position of the substituents in the aromatic ring did not have any effect on the reactivity, affording the corresponding *ortho*-, *meta*-, or *para*-substituted products **3j**–**3m** in similar yields. Disubstituted 1,1-diphenyl styrene also provides product **3n** in 70% isolated yield. Notably, the 1,6-imino alcohol motif was successfully installed in various complex biomolecules and pharmaceuticals, such as estrone (**3o**, 60%), zingerone (**3p**, 75%), flurbiprofen (**3q**, 87%), and gemfibrozil (**3r**, 73%), proving that this protocol can be used for late-stage functionalization. In all cases, a regioselective addition of the first carbon-centered radical takes place at the β-position of the activated alkene, and the generated α-radical recombines with the iminyl radical to afford iminyl alkyl alcohols **3**. Interestingly, 1,2-carboimination of bicyclo[1.1.0]­butane **1s** was also successfully achieved, affording cyclobutane **3s** in 19% isolated yield (80% of **1s** remained unreactive). Unfortunately, phenyl acetylene, non-activated alkenes, and rigid internal alkenes did not afford the corresponding desired products **3**.^15^

**Scheme 1:**
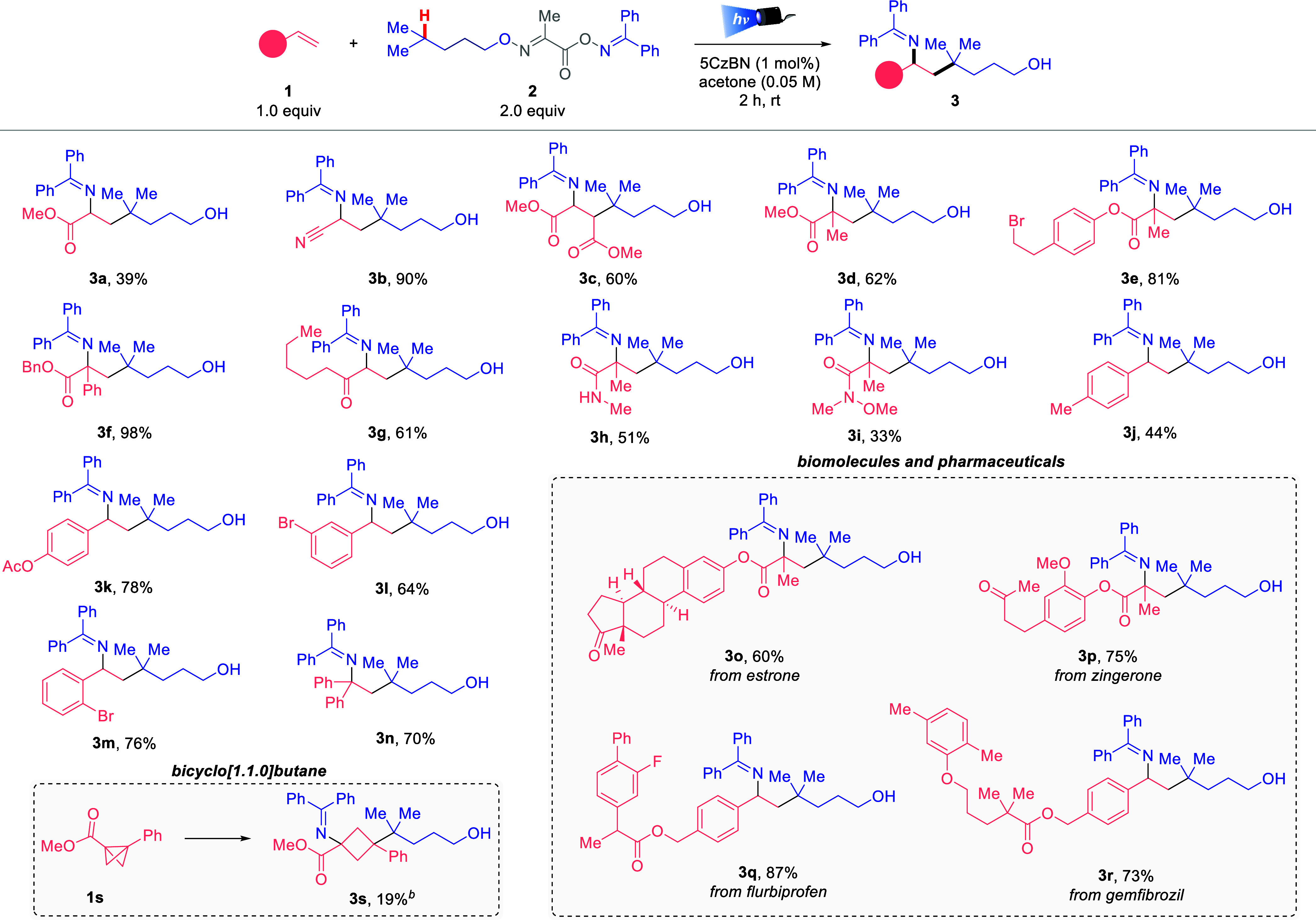
Alkene Scope^a^

a Standard conditions: **1** (0.20 mmol), **2a** (0.40 mmol), and 5CzBN (1 mol %) in dry degassed acetone (4.0 mL, 0.05 M) under irradiation (blue Kessil lamp; λ_max_ = 427 nm) for 2 h at rt under inert atmosphere.
b Eighty percent of the starting BCB remained.

Different bifunctional reagents **2** were further examined using acrylonitrile as the radical acceptor (Scheme 2). Nonfunctionalized alkyl alcohols were amenable to this transformation, demonstrating that stabilized tertiary alkyl radical precursor **2a** afforded the desired compound and the final 1,6-imino alcohol structures can be prepared from nonstabilized primary (**3t**) and secondary (**3u** and **3ab–3af**) alkyl radical precursors to afford the desired products in good to high yields. Additionally, this 1,2-carboimination protocol presented excellent functional group tolerance, generating the desired 1,6-imino alcohol product carrying a chlorine (**3v**), azide (**3w**), ester (**3x**), ether (**3y** and **3ag**), or thioether (**3ah**) functional group. Electron-poor and electron-rich heterocycles were suitable for this transformation, providing pyridine derivative **3z** in 75% yield and thiophene **3aa** in 40% yield. Furthermore, *N*-Boc- and silyl-protected groups remained intact after irradiation, and products **3ai** and **3aj** were obtained in 83% and 89% yields, respectively. Benzylic and α-alkyne radical precursors (**2s** and **2t**, respectively) did not afford the expected 1,2-carboimination product, but the corresponding 1,4-amino alcohols derived from the direct insertion of the iminyl radical.^10,15^ The scale-up of this transformation was performed using a homemade continuous-flow photoreactor, affording the desired product **3b** in 84% yield isolated yield after irradiation for 5 h, with a residence time (*t*_R_) of 15 min.^15^

**Scheme 2:**
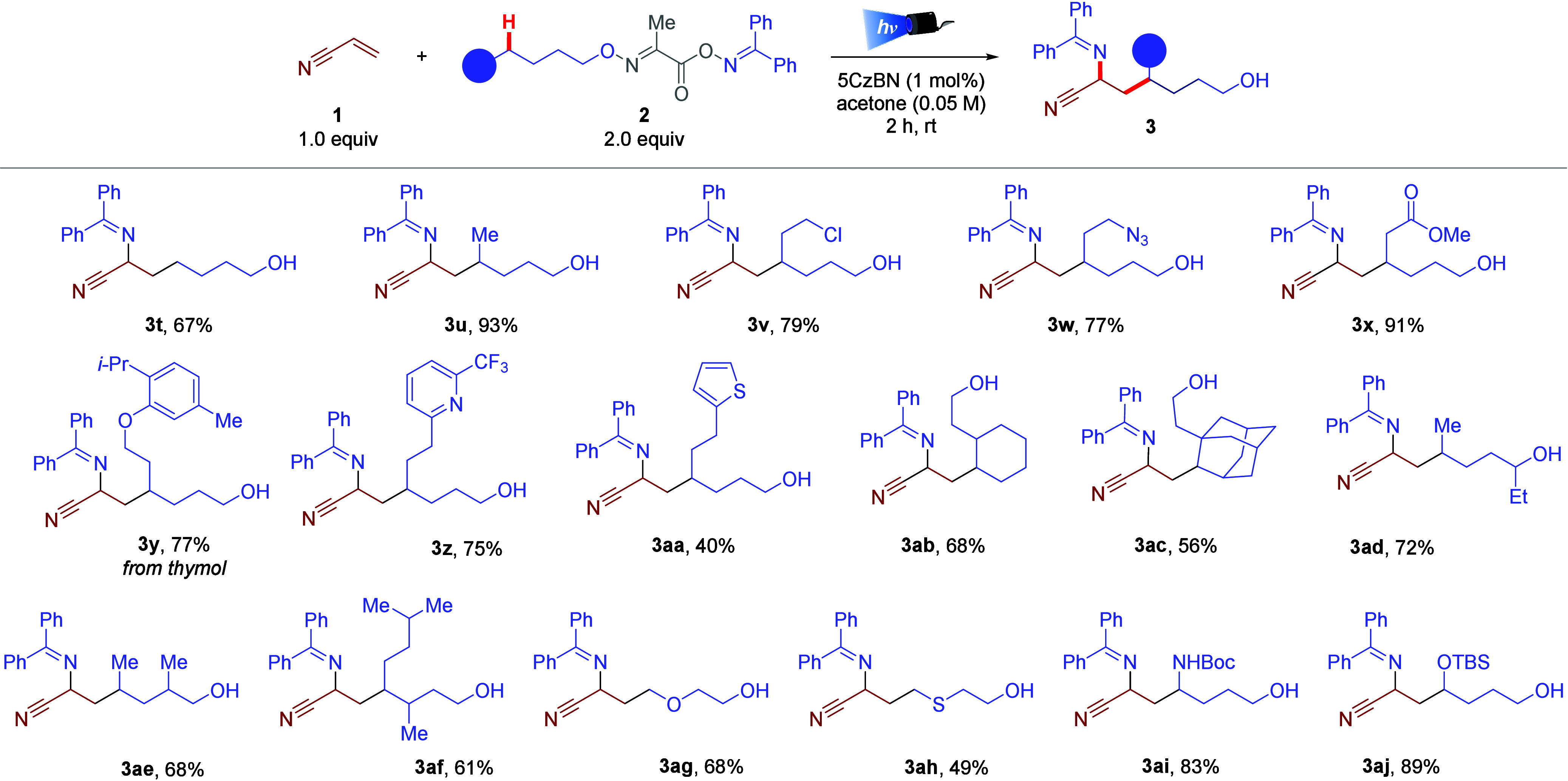
Bifunctional Reagent Scope^a^

a Standard conditions: **1b** (0.20 mmol), **2** (0.40 mmol), and 5CzBN (1 mol %) in dry degassed acetone (4.0 mL, 0.05 M) under irradiation (blue Kessil lamp; λ_max_ = 427 nm) for 2 h at rt under inert atmosphere.

The utility of these 1,6-iminyl alkyl alcohols as important synthetic intermediates was proven with the derivatization of four products to the corresponding amino esters or imino acids (Scheme 3). Compounds **3f**, **3s**, and **3ag** were subjected to a Steglich esterification process, followed by imine hydrolysis, to afford zingerone–indomethacin derivative **4**, gemfibrozil–feboxostat derivative **5**, and flurbiprofen derivative **6** in good yields. Finally, the potential preparation of 1,6-amino acids for its application in the synthesis of linkers and highly functionalized polymers was proven by the selective alcohol oxidation of **3b** mediated by PIDA and TEMPO (Scheme 3).

**Scheme 3:**
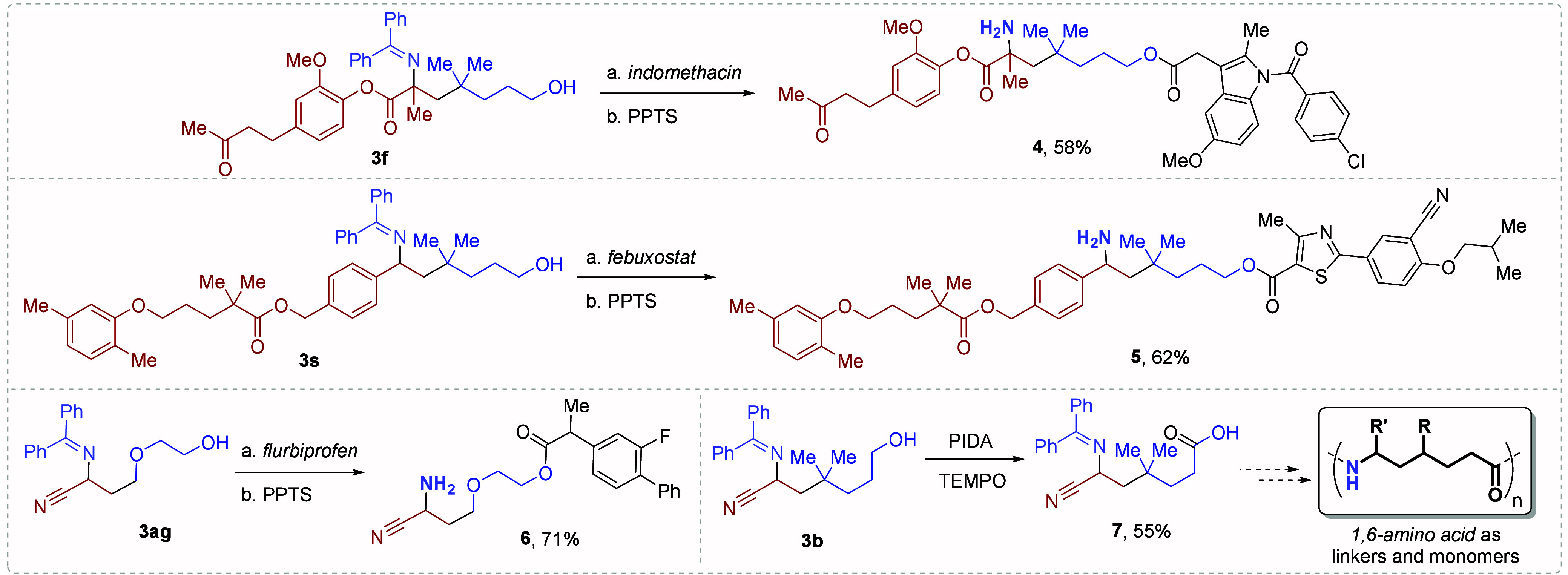
Derivatization Reactions

To elucidate the mechanism behind this EnT process, experiments were performed.^15^ The photochemical quantum yield (Φ) of this multicomponent reaction was experimentally determined using two different alkenes (**1b** and **1f**) and bifunctional reagents (**2a** and **2b**), to form **3f** and **3t**, respectively. Both measurements gave a Φ value ∼65, suggesting a radical chain mechanism initiated by the photocatalyst action. Moreover, the on/off-light experiment supported the hypothesis of a radical chain mechanism versus a radical–radical coupling. The presence of 4.0 equiv of TEMPO under the standard conditions completely stifled the reaction, with the methyl methacrylate and bifunctional reagent **2a** being recovered intact. Additionally, the irradiation of the mixture of **2b** and acrylonitrile in acetone with a purple Kessil lamp (λ_max_ = 390 nm), in the absence of the photocatalyst, afforded the desired product **3t** in 33% yield. The direct excitation of the bifunctional reagent and the TEMPO experiment ruled out the possibility of a redox process and proved the existence of an energy transfer event between the photocatalyst and bifunctional reagent **2**, in which the alkenes act as radical acceptors.

On the basis of these observations, our previous results,^10^ and similar energy transfer processes,^7c^ a possible mechanism for the synthesis of 1,6-amino alcohols **3** was proposed (Figure 2). The process starts with the irradiation of 5CzBN to generate excited triplet state 5CzBN* (*E*_T_ = 2.68 eV).^17^ 5CzBN* interacts with bifunctional reagent **2** to form excited triplet state [**2**]* in an energy transfer event, which induces the homolytic cleavage of the O–N bond, affording iminyl radical **B** and alkoxy radical **A**, with extrusion of CO_2_ and acetonitrile. Later, **A** abstracts a hydrogen from position C5 (1,5-HAT) and generates C­(sp^3^) radical **C**, which engages in a Giese-type addition with alkene **1** to form C­(sp^3^) radical intermediate **D**. Given the experimental quantum yield (Φ ∼ 60), the radical–radical coupling pathway reported in several energy transfer reactions is dismissed for this transformation, favoring the radical chain pathway. Thus, **D** reacts with another molecule of bifunctional reagent **2** to achieve the desired 1,6-amino alcohol **3** and alkoxy radical **A** with the release of carbon dioxide and acetonitrile, re-establishing the cycle.^15^

**Figure 2:**
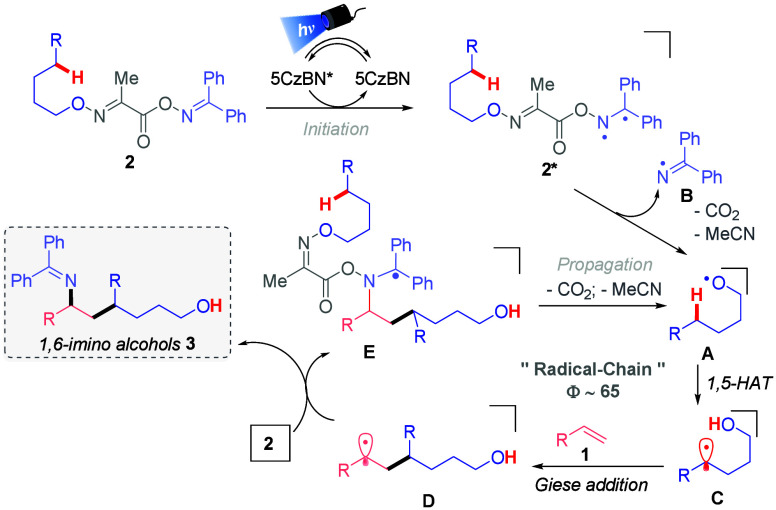
Proposed mechanism.

In summary, an operationally simple 1,2-carboimination of alkenes to synthesize highly functionalized 1,6-imino alcohols was described. We have uncovered a domino process that embraces the formation of iminyl radicals and alkyl radicals, generated from alkoxy radicals by a 1,5-HAT process, with the 1,2-carboimination of alkenes. Activated alkenes with electron-withdrawing groups and also various styrene-type alkenes were amenable to this method, yielding excellent results. Scale-up was efficiently achieved using a custom-built continuous-flow system, which maintained reactivity comparable to that observed in batch processing. Moreover, this multicomponent reaction proved to be highly effective for the late-stage functionalization of complex molecules, enabling the incorporation of the 1,6-imino alcohol motif in various biomolecules and pharmaceuticals. The synthetic utility of the 1,6-imino alcohol motifs was further highlighted by several derivatizations, leading to the isolation of highly functionalized molecules, such as gemfibrozil–febuxostat derivative **5** or 1,6-imino acid **7**, a potential monomer for producing complex nylon derivatives.

## Supplementary Material



## Data Availability

The data underlying this study are available in the published article and its Supporting Information.

## Notes

Details about the preparation of starting materials, general procedures, and analytical data [nuclear magnetic resonance (NMR), mass spectrometry, and melting points] along with copies of the NMR spectra (PDF)
