# Supplementary material for: Visible-Light-Promoted Synthesis of 1,6-Imino Alcohols by Metal-Free 1,2-Carboimination of Alkenes
Source: Org Lett. 2025 Mar 3;27(10):2346–51. doi: 10.1021/acs.orglett.5c00082 (PMC12129255; doi:10.1021/acs.orglett.5c00082)
Supplement: Supplementary file 1 [file ol5c00082_si_001.pdf]

Supporting Information

Visible-Light-Promoted Synthesis of 1,6-Imino Alcohols by Metal-Free 1,2-Carboimination of Alkenes

María J. Cabrera-Afonso,*[a] Aida Jaafar,[a] Christian Cristóbal,[a] Javier Adrio,*[a,b,c] Maria Ribagorda*[a,b]

[a] *Departamento de Química Orgánica, Facultad de Ciencias, Universidad Autónoma de Madrid, 28049 Madrid, Spain.*

[b] *Institute for Advanced Research in Chemical Sciences (IAdChem), Universidad Autónoma de Madrid, 28049 Madrid, Spain.*

[c] *Center for Innovation in Advanced Chemistry (ORFEO-CINQA) UAM, 28049 Madrid, Spain*

*To whom correspondence should be addressed. E-mail: mjesus.cabrera@uam.es; javier.adrio@uam.es; maria.ribagorda@uam.es;

TABLE OF CONTENT

1. General Considerations 2
2. Synthesis of Starting Materials 3
3. Synthesis of 1,6-Imino Alcohols: Reaction Workflow, Optimization and Characterization 9
   3.1. Reaction Workflow: 9
   3.2. Reaction Optimization 9
   3.3. General Procedure 11
   3.4. Unsuccessful Results: 12
   3.5. Characterization Data: 12
4. Derivatization Reactions 30
5. Large Scale Synthesis of 3b 33
6. Mechanistic Investigations 34
   6.1. UV/vis studies 34
   6.2. Quantum yield 34
   6.3. TEMPO trapping experiment 39
   6.4. Direct Excitation 41
   6.5. Light ON / OFF experiment 42
   6.6. Akyl radical proof experiment 43
   6.7. Proposed mechanism and detailed intermediates from D to final compound 3 44
7 NMR Spectra 45

1. General Considerations

1.1 General: All chemical transformations that requiered inert atmosphere were done using Schlenk line techniques. For purple and blue light irradiation a Kessil PR160-purple LED lamp (30 W High Luminous DEX 2100 LED, λ_{max} = 390 nm), a Kessil PR160-blue LED lamp (30 W High Luminous DEX 2100 LED, λ_{max} = 427 nm) or a Kessil PR160-blue LED LED lamp (30 W High Luminous DEX 2100 LED, λ_{max} = 456 nm) was placed 4 cm away from the reaction vials. NMR spectra (^{1}H, ^{13}C, ^{19}F) were acquired on a Bruker Avance 300 and 500 MHz spectrometers. Chemical shifts (δ) are reported in ppm relative to residual solvent signals ($CDCl_3$, δ_H = 7.26 ppm, δ_C = 77.16 ppm). ^{13}C NMR and ^{19}F spectra were acquired on a broad band decoupled mode. The following abbreviations are used to describe peak patterns when appropriate: s (singlet), d (doublet), t (triplet), q (quartet), quint (quintet), m (multiplet), bs (broad singlet). Analytical thin layer chromatography (TLC) was performed using pre-coated aluminum-backed plates (60 Å porosity, 250 μm thickness) in heptane/EtOAc as the eluent, and visualized using potassium permanganate stain, and/or UV light. Reactions were monitored by ^{1}H NMR, and/or TLC. Flash column chromatography was accomplished using silica gel Merck-60 from Aldrich. High Resolution Mass Spectrometry (HRMS) were registered in a spectrometer Bruker maXis IITM (Q-TOF) or a GCT Agilent Technologies 6890 N using Atmospheric Pressure Chemical Ionization (APCI) or Electrospray Ionization (ESI) method. Melting points (°C) were measured using Büchi Melting Point B-540 apparatus in open capillary tubes, and the values are uncorrected. UV/vis measurements were measured in a 1 cm quartz cuvette using a JASCO V-660 UV/vis spectrophotometer. The continuous-flow experiment was carried out using a homemade flow setup with a syringe pump (Chemyx Fusion 100), two Kessil lamps, and 1.36 mL perfluoroalcoxy (PFA) reactor coil (inner diameter: 1.6 mm, external diameter 3.0 mm). Photoredox-catalyzed reactions were performed using 8 mL Screw Neck Vial (clear glass, 45 x 14.7 mm) with screw cap 13 mm black Sil/PTFE septum.

1.2 Chemicals: Deuterated NMR solvents were purchased from Sigma Aldrich. CH_2Cl_2, absolute ethanol, methanol, dry THF and dry acetone were obtained from Carlo Erba, and used as received. Alcohols, triphenylphosphine, *N*-hydroxyphtalimide, DIAD, DMAP, hydrazine hydrate, methyl pyruvate, pyruvic acid, and EDC·HCl were purchased from commercial suppliers and used as received. Diphenylmethanone oxime,[1] alkenes **1e-h**, **1k**[2] and **1r-s**,[3] BCB **1l**,[4] and 5CzBN[5] were synthesized following reported procedures. The synthesis of the bifunctional reagents **2a-m** and **2o** were described in our previous publication.[6]

---

[1] Gao, Y.; Liu, J.; Li, Z.; Guo, T.; Xu, S.; Zhu, H.; Wei, F.; Chen, S.; Gebru, H.; Guo, K. *J. Org. Chem.* **2018**, *83*, 2040-2049.
[2] Hell, S. M.; Meyer, C. F.; Laudadio, G.; Misale, A.; Willis, M. C.; Noel, T.; Trabanco, A. A.; Gouverneur, V. *J. Am. Chem. Soc.* **2020**, *142*, 720-725.
[3] a) Wang, Z.; Yang, Y.*RSC Adv.*, **2020**, *10*, 29263-29267; b) Granados, A.; Dhungana, R. K.; Sharique, M.; Majhi, J.; Molander, G. A., *Org. Lett.* **2022**, *24*, 4750-4755.
[4] Ma, X.; Sloman, D. L.; Han, Y.; Bennett, D. J.*Org. Lett.* **2019**, *21* 7199-7203.
[5] Lu, J.; Pattengale, B.; Liu, Q.; Yang, S.; Shi, W.; Li, S.; Huang, J.; Zhang, J. *J. Am. Chem. Soc.* **2018**, *140*, 13719-13725.
[6] Cristóbal, C.; Alonso, I.; Cabrera-Afonso, M. J.; Adrio, J.; Ribagorda, M. *Org. Chem. Front.* **2024**, *11*, 7037-7043.

Synthesis of Starting Materials

The synthesis of the bifunctional reagents **2a-m** and **2o** were described in our previous publication.[6]

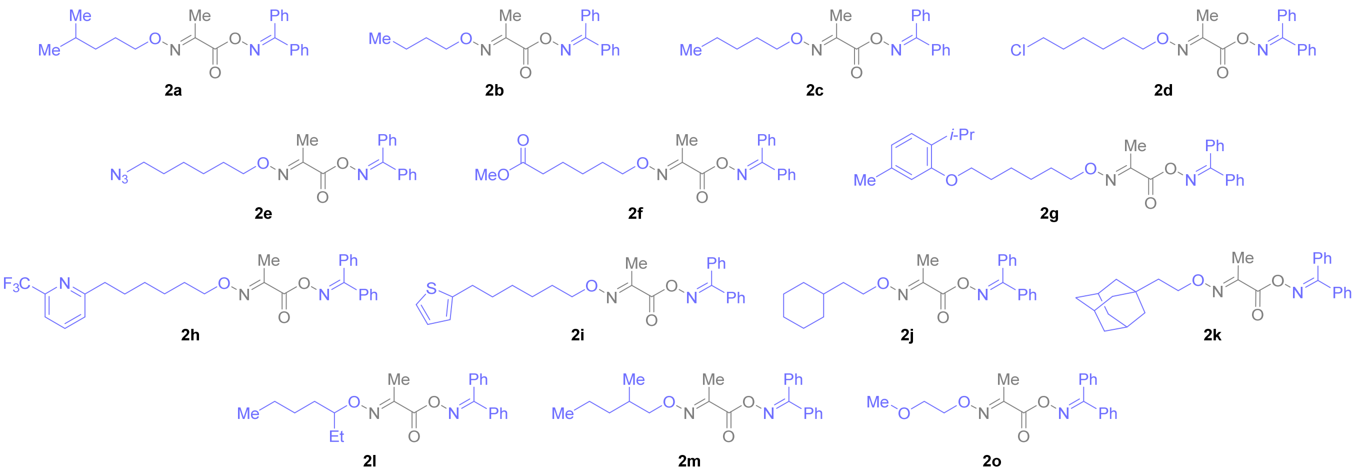

Synthesis of bifunctional *N*-oxime 2n

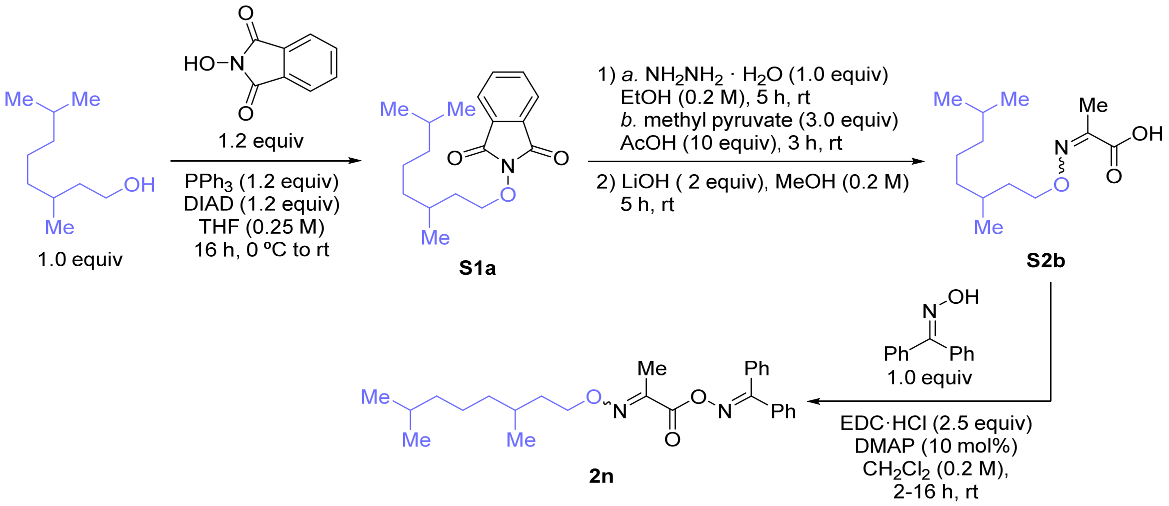

A solution of 3,7-dimethyloctan-1-ol (1.0 mL, 5.00 mmol, 1.0 equiv), *N*-hydroxyphthalimide (979 mg, 6.00 mmol, 1.2 equiv) and triphenylphosphine (1.6 g, 6.00 mmol, 1.2 equiv) in THF (20 mL, 0.25 M) under a nitrogen atmosphere was cooled to 0 ºC with an icebath. Diisopropylazodicarboxylate 1.0 M in Toluene (1.2 mL, 6.00 mmol, 1.2 equiv) was added dropwise to the solution at 0 ºC. The resulting mixture was stirred at 0 ºC for 10 minutes, after that the icebath was removed, and the reaction was stirred at room temperature for 16 h or until the reaction was complete by TLC analysis. Then, the reaction mixture was concentrated under reduced pressure, and the resultant phthalimide **S1a** was then used directly for the next step.

To a stirred suspension of the previous phtalimide **S1a** in absolute ethanol (25 mL, 0.2 M), hydrazine 1.0 M in THF (5 mL, 5.00 mL, 1.0 equiv) was added in a single portion. The mixture was stirred at room temperature for 5 hours,

and the formation of a white precipitate was observed. The white solid was filtered off through a plug of Celite, and the filter pad was washed with a minimal amount of absolute ethanol. Then, methyl pyruvate (1.4 mL, 15 mmol, 3.0 equiv) and acetic acid (2.9 mL, 50 mmol, 10.0 equiv) were added to the solution, and the resulting mixture was stirred for 12 h at room temperature. Upon completion, the reaction mixture was concentrated under reduced preassure, and washed with water. The crude mixture was subjected to purification by flash column chromatography (0-10 % AcOEt in heptane) to obtain the corresponding ester intermediate. Then, this ester was dissolved in mixture of methanol/H_2O (25 mL, 0.2 M, 3:1), and LiOH·H_2O (630 mg, 15 mmol, 3.0 equiv) was added. The resulting mixture was stirred at room temperature until the reaction was complete by TLC analysis (3-5 h). The reaction was concentrated under reduced pressure, acidified using a 10% aq HCl soln, and extracted with AcOEt (2x20 mL). The combined organic layers were dried (Na_2SO_4), and the volatiles were removed under reduced pressure, obtaining **S2a**. (NOTE: No special caution for avoid O_2 in this reaction.).

To a stirred solution of diphenylmethanone oxime (334 mg, 1.60 mmol, 1.0 equiv), the corresponding α-oxime acid **S2a** (595 mg, 2.40 mmol, 1.5 equiv), and 4-dimethylaminopyridine (21 mg, 0.17 mmol, 10 mol%) in CH_2Cl_2 (8.2 mL, 0.2 M), 1-ethyl-3-(3-dimethylaminopropyl)carbodiimide hydrochloride (786 mg, 4.10 mmol, 2.5 equiv) was added. The reaction was monitored by TLC analysis, and upon completion (2 – 16 h), the mixture was poured into a separatory funnel and washed with 10% aq HCl soln (4x20 mL). The organic layer was washed with satd NaCl soln, dried (Na_2SO_4), and the volatiles were removed under reduced pressure. The crude mixture was subjected to purification by flash column chromatography to obtain **2n** (620 mg, 1.46 mmol, 90 %). (NOTE: No special caution for avoid O_2 and H_2O in this reaction.).

^{1}H NMR (300 MHz, $CDCl_3$), δ (ppm) = 7.69 – 7.57 (m, 2H), 7.51 – 7.24 (m, 8H), 4.28 – 4.14 (m, 2H), 1.95 (s, 3H), 1.71 – 1.62 (m, 1H), 1.58 – 1.43 (m, 3H), 1.33 – 1.23 (m, 3H), 1.19 – 1.09 (m, 3H), 0.91 – 0.85 (m, 9H). **^{13}C{^{1}H} NMR** (76 MHz, $CDCl_3$), δ (ppm) = 165.9, 161.3, 147.1, 134.7, 132.3, 131.0, 129.8, 129.4 (2C), 129.3 (2C), 128.4 (2C), 128.0 (2C), 74.3, 39.2, 37.3, 35.9, 29.8, 28.0, 24.6, 22.7, 22.6, 19.7, 11.2. **HRMS (APCI)** calcd for $C_{26}H_{35}N_2O_3$ $[M+H]^+$: 423.2642, found 423.2646.

Synthesis of bifunctional *N*-oxime 2p

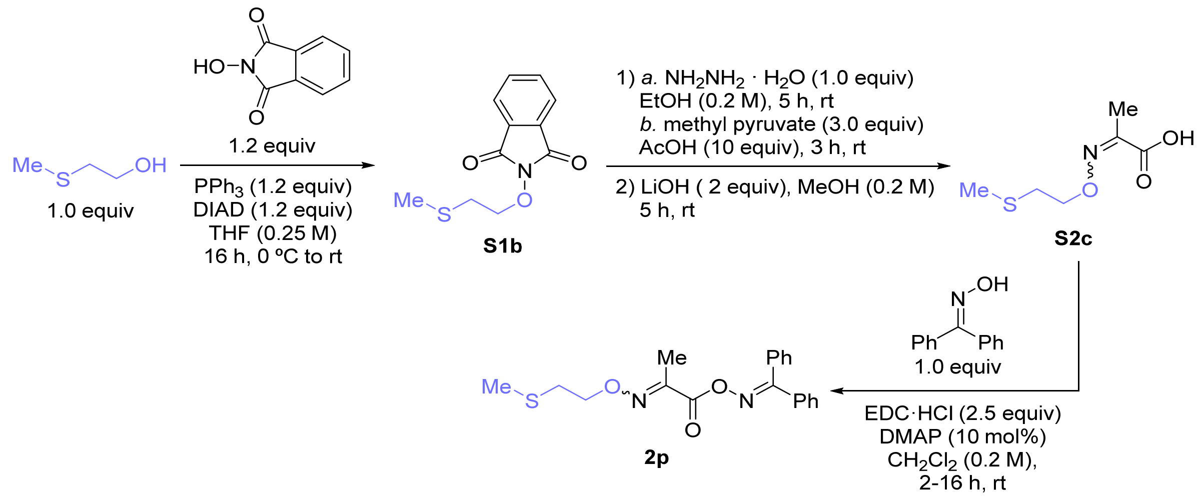

A solution of 2-(methylthio)ethan-1-ol (0.5 mL, 5.00 mmol, 1.0 equiv), *N*-hydroxyphthalimide (979 mg, 6.00 mmol, 1.2 equiv) and triphenylphosphine (1.6 g, 6.00 mmol, 1.2 equiv) in THF (20 mL, 0.25 M) under a nitrogen atmosphere was cooled to 0 ºC with an icebath. Diisopropylazodicarboxylate 1.0 M in Toluene (1.2 mL, 6.00 mmol, 1.2 equiv) was added dropwise to the solution at 0 ºC. The resulting mixture was stirred at 0 ºC for 10 minutes, after that the icebath was removed, and the reaction was stirred at room temperature for 16 h or until the reaction was complete by TLC analysis. Then, the reaction mixture was concentrated under reduced pressure, and the resultant phthalimide **S1a** was then used directly for the next step.

To a stirred suspension of the previous phtalimide **S1b** in absolute ethanol (25 mL, 0.2 M), hydrazine 1.0 M in THF (5 mL, 5.00 mL, 1.0 equiv) was added in a single portion. The mixture was stirred at room temperature for 5 hours, and the formation of a white precipitate was observed. The white solid was filtered off through a plug of Celite, and the filter pad was washed with a minimal amount of absolute ethanol. Then, pyruvic acid (1.0 mL, 15.00 mmol, 3.0 equiv) was added to the solution, and the resulting mixture was stirred for 12 h at room temperature. Upon completion, the reaction mixture was concentrated under reduced pressure. The crude mixture was dissolved in EtOAc and extracted with brine (5x20mL), dried (Na_2SO_4), and the volatiles were removed under reduced pressure. The crude mixture employed without further purification in the next reaction step.

To a stirred solution of diphenylmethanone oxime (651 mg, 3.30 mmol, 1.0 equiv), the corresponding α-oxime acid **S2b** (886 mg, 5.00 mmol, 1.5 equiv), and 4-dimethylaminopyridine (41 mg, 0.33 mmol, 10 mol%) in CH_2Cl_2 (16.5 mL, 0.2 M), 1-ethyl-3-(3-dimethylaminopropyl)carbodiimide hydrochloride (1.6 g, 8.25 mmol, 2.5 equiv) was added. The reaction was monitored by TLC analysis, and upon completion (2 – 16 h), the mixture was poured into a separatory funnel and washed with 10% aq HCl soln (4x20 mL). The organic layer was washed with satd NaCl soln, dried (Na_2SO_4), and the volatiles were removed under reduced pressure. The crude mixture was subjected to purification by flash column chromatography to obtain **2p** (217 mg, 0.61 mmol, 19 %). (NOTE: No special caution for avoid O_2 and H_2O in this reaction.).

^{1}H NMR (300 MHz, $CDCl_3$), δ (ppm) = 7.68 – 7.52 (m, 2H), 7.51 – 7.33 (m, 8H), 4.29 (t, *J* = 7.0 Hz, 2H), 2.70 (t, *J* = 7.0 Hz, 2H), 2.10 (s, 3H), 1.98 (s, 3H). **^{13}C{^{1}H} NMR** (76 MHz, $CDCl_3$), δ (ppm) = 166.2, 161.2, 148.2, 134.7, 132.5, 131.1, 129.9, 129.4 (4C), 128.5 (2C), 128.1 (2C), 74.4, 32.8, 16.0, 11.4. **HRMS (APCI)** calcd for $C_{19}H_{21}N_2O_3S$ $[M+H]^+$: 357.1267, found 357.1268.

Synthesis of bifunctional *N*-oxime 2q

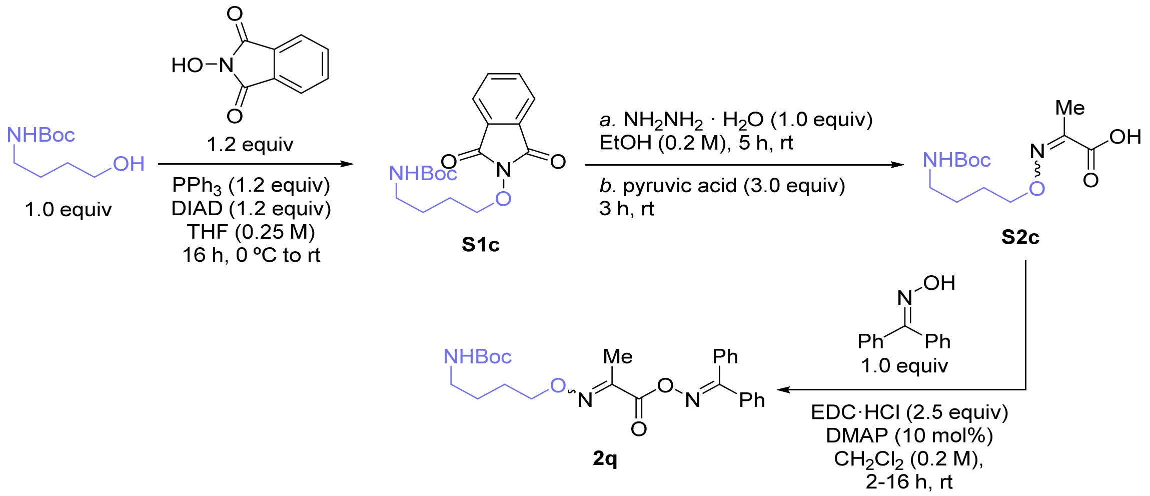

A solution of *tert*-butyl (4-hydroxybutyl)carbamate (3.2 g, 17.0 mmol, 1.0 equiv), *N*-hydroxyphthalimide (3.3 g, 20.4 mmol, 1.2 equiv) and triphenylphosphine (4.1 g, 6.20 mmol, 1.2 equiv) in THF (68 mL, 0.25 M) under a nitrogen atmosphere was cooled to 0 ºC with an icebath. Diisopropylazodicarboxylate 1.0 M in Toluene (4.1 mL, 20.4 mmol, 1.2 equiv) was added dropwise to the solution at 0 ºC. The resulting mixture was stirred at 0 ºC for 10 minutes, after that the icebath was removed, and the reaction was stirred at room temperature for 16 h or until the reaction was complete by TLC analysis. Then, the reaction mixture was concentrated under reduced pressure, and the resultant phthalimide **S1a** was then used directly for the next step.

To a stirred suspension of the previous phtalimide **S1c** in absolute ethanol (85 mL, 0.2 M), hydrazine 1.0 M in THF (17 mL, 17.0 mmol, 1.0 equiv) was added in a single portion. The mixture was stirred at room temperature for 5 hours, and the formation of a white precipitate was observed. The white solid was filtered off through a plug of Celite, and the filter pad was washed with a minimal amount of absolute ethanol. Then, pyruvic acid (3.6 mL, 51.00 mmol, 3.0 equiv) was added to the solution, and the resulting mixture was stirred for 12 h at room temperature. Upon completion, the reaction mixture was concentrated under reduced pressure. The crude mixture was dissolved in EtOAc and extracted with brine (5x20mL), dried (Na_2SO_4), and the volatiles were removed under reduced pressure. The crude mixture employed without further purification in the next reaction step.

To a stirred solution of diphenylmethanone oxime (2.3 g, 11.30 mmol, 1.0 equiv), the corresponding α-oxime acid **S2c** (4.6 g, 17 mmol, 1.5 equiv), and 4-dimethylaminopyridine (139 mg, 1.1 mmol, 10 mol%) in CH_2Cl_2 (56.5 mL, 0.2 M), 1-ethyl-3-(3-dimethylaminopropyl)carbodiimide hydrochloride (5.4 g, 28.3 mmol, 2.5 equiv) was added. The reaction was monitored by TLC analysis, and upon completion (2 – 16 h), the mixture was poured into a separatory funnel and washed with 10% aq HCl soln (4x20 mL). The organic layer was washed with satd NaCl soln, dried (Na_2SO_4), and the volatiles were removed under reduced pressure. The crude mixture was subjected to purification by flash column chromatography to obtain **2q** (1.1 g, 2.34 mmol, 21%). (NOTE: No special caution for avoid O_2 and H_2O in this reaction.).

^{1}H NMR (500 MHz, $CDCl_3$), δ (ppm) = 7.66 – 7.58 (m, 2H), 7.49 – 7.40 (m, 4H), 7.42 – 7.33 (m, 4H), 4.55 (bs, 1H), 4.16 (t, *J* = 6.5 Hz, 2H), 3.11 (q, *J* = 6.7 Hz, 2H), 1.94 (s, 3H), 1.71 – 1.62 (m, 2H), 1.56 – 1.48 (m, 2H), 1.43 (s, 9H). **$^{13}C\{^{1}H\}$ NMR** (126 MHz, $CDCl_3$), δ (ppm) = 166.2, 161.3, 156.1, 147.6, 134.7, 132.5, 131.1, 129.9, 129.5 (2C), 129.4 (2C), 128.5 (2C), 128.1 (2C), 79.2, 75.3, 40.4, 28.5 (3C), 26.6, 26.5, 11.3. **HRMS (APCI)** calcd for $C_{25}H_{32}N_3O_5$ $[M+H]^+$: 454.2336, found 454.2341.

Synthesis of bifunctional *N*-oxime 2r

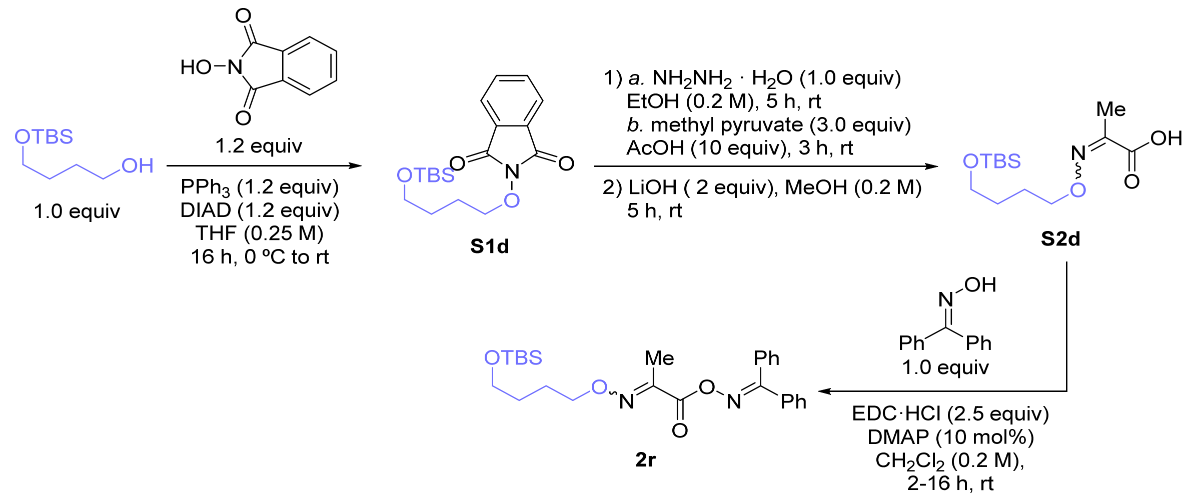

A solution of 4-((*tert*-butyldimethylsilyl)oxy)butan-1-ol (2.0 g, 10.00 mmol, 1.0 equiv), *N*-hydroxyphthalimide (1.9 g, 12.00 mmol, 1.2 equiv) and triphenylphosphine (3.2 g, 12.00 mmol, 1.2 equiv) in THF (40 mL, 0.25 M) under a nitrogen atmosphere was cooled to 0 ºC with an icebath. Diisopropylazodicarboxylate (2.4 g, 12.00 mmol, 1.2 equiv) was added dropwise to the solution at 0 ºC. The resulting mixture was stirred at 0 ºC for 10 minutes, after that the icebath was removed, and the reaction was stirred at room temperature for 16 h or until the reaction was complete by TLC analysis. Then, the reaction mixture was concentrated under reduced pressure, and the resultant phthalimide **S1a** was then used directly for the next step.

To a stirred suspension of the previous phtalimide **S1d** in absolute ethanol (40 mL, 0.2 M), hydrazine 1.0 M in THF (10 mL, 10.00 mmol, 1.0 equiv) was added in a single portion. The mixture was stirred at room temperature for 5 hours, and the formation of a white precipitate was observed. The white solid was filtered off through a plug of Celite, and the filter pad was washed with a minimal amount of absolute ethanol. Then, methyl pyruvate (3.1 g, 30.00 mmol, 3.0 equiv) and acetic acid (6.0 g, 100.00 mmol, 10.0 equiv) were added to the solution, and the resulting mixture was stirred for 12 h at room temperature. Upon completion, the reaction mixture was concentrated under reduced preassure, and washed with water. The crude mixture was subjected to purification by flash column chromatography (0-10 % AcOEt in heptane) to obtain the corresponding ester intermediate. Then, this ester was dissolved in mixture of methanol/H_2O (50 mL, 0.2 M, 3:1), and LiOH (1.3 g, 30.00 mmol, 3.0 equiv) was added. The resulting mixture was stirred at room temperature until the reaction was complete by TLC analysis (3 h). The reaction was concentrated under reduced pressure, acidified using a 10% aq HCl soln, and extracted with AcOEt

(2x20 mL). The combined organic layers were dried (Na_2SO_4), and the volatiles were removed under reduced pressure, obtaining **S2d**. (NOTE: No special caution for avoid O_2 in this reaction.).

To a stirred solution of diphenylmethanone oxime (500 mg, 2.53 mmol, 1.0 equiv), the corresponding α-oxime acid **S2d** (1.1 g, 3.80 mmol, 1.5 equiv), and 4-dimethylaminopyridine (31 mg, 0.25 mmol, 10 mol%) in CH_2Cl_2 (12.7 mL, 0.2 M), 1-ethyl-3-(3-dimethylaminopropyl)carbodiimide hydrochloride (1.2 g, 6.33 mmol, 2.5 equiv) was added. The reaction was monitored by TLC analysis, and upon completion (16 h), the mixture was poured into a separatory funnel and washed with 10% aq HCl soln (4x20 mL). The organic layer was washed with satd NaCl soln, dried (Na_2SO_4), and the volatiles were removed under reduced pressure. The crude mixture was subjected to purification by flash column chromatography to obtain **2r** (654 mg, 1.44 mmol, 60 %). (NOTE: No special caution for avoid O_2 and H_2O in this reaction.).

^{1}H NMR (300 MHz, $CDCl_3$), δ (ppm) = 7.65 – 7.58 (m, 2H), 7.50 – 7.33 (m, 8H), 4.19 (t, *J* = 6.4 Hz, 2H), 3.62 (t, *J* = 6.3 Hz, 2H), 1.96 (s, 3H), 1.75 – 1.65 (m, 2H), 1.60 – 1.51 (m, 2H), 0.90 (s, 9H), 0.05 (s, 6H). **^{13}C{^{1}H} NMR** (75 MHz, $CDCl_3$), δ (ppm) = 166.1, 161.4, 147.4, 134.8, 132.5, 131.1, 129.9, 129.6 (2C), 129.4 (2C), 128.5 (2C), 128.1 (2C), 75.7, 62.9, 29.3, 26.1 (3C), 25.7, 18.5, 11.3, -5.2 (2C). **HRMS (APCI)** calcd for $C_{26}H_{37}N_2O_4Si$ $[M+H]^+$: 469.2517, found 469.2515.

2. Synthesis of 1,6-Imino Alcohols: Reaction Workflow, Optimization and Characterization

3.1. Reaction Workflow:

All photoredox reactions were performed with a Kessil PR160-blue LED lamp (30 W High Luminous DEX 2100 LED, λ_{max} = 427 nm). The lamp was placed 4 cm away from the reaction vials, and cooled at 25ºC by an external fan. A typical reaction setup is shown below.

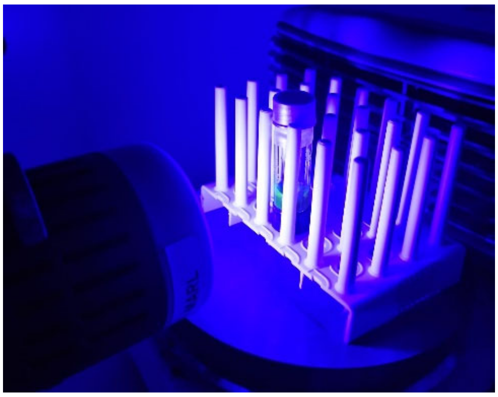

Figure S1. Reaction setup for the visible light-assisted synthesis of 1,6-aminoalcohols

3.2. Reaction Optimization

The initial reaction conditions were selected based on our preliminary studies for the generation of δ-amino alcohols via the EnT photocatalysis protocol using N-oxime-type bifunctional reagents, where the best results were obtained using 5CzBn (1 mol%) as the organo-PC catalyst in acetone (0.1 M) under blue light irradiation (λmax = 427 nm).[6] Additionally, in the present work, we have investigated the influence of the bifunctional reagent-to-alkene ratio (Table S1), concentration and reaction time (Table S2), different solvents (Table S3), and control experiments (Table S4).

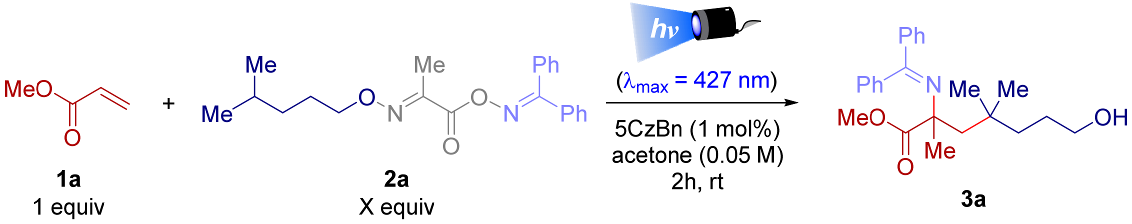

Table S1. Equivalents of **2a**[a]

Entry	Equiv of 2a	3a (%)[b]
**1**	1.5	39
**2**	2.0	47
**3**	2.5	47
**4**	3.0	47

[a] Optimization reactions were performed using methyl acrylate (**1a**, 0.05 mmol), bifunctional reagent **2a** (x equiv) and 5CzBN (1 mol%), in dry degassed acetone (1.0 mL, 0.05 M) under blue Kessil irradiation (λ_{max} = 427 nm) for 2 hours at room temperature under inert atmosphere. [b] All the reactions were analyzed by ^{1}H NMR using 1,3,5-trimethoxybenzene as internal standard.

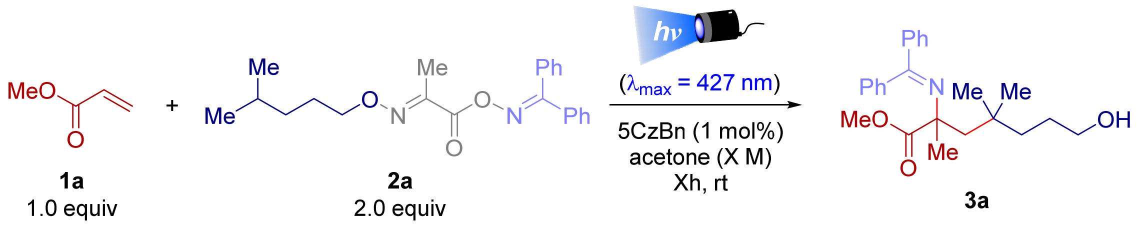

Table S2. Concentration and reaction time evaluation[a]

Entry	Acetone [M]	Time (h)	3a (%)[b]
**1**	0.05	2	47 (39)[c]
**2**	0.1	2	31
**3**	0.2	2	47 (20)[c]
**4**	0.05	16	30

[a] Optimization reactions were performed using methyl acrylate (**1a**, 0.05 mmol), bifunctional reagent **2a** (2.0 equiv) and 5CzBN (1 mol%), in dry degassed acetone (x M) under blue Kessil irradiation (λ_{max} = 427 nm) for 2 hours at room temperature under inert atmosphere. [b] All the reactions were analyzed by ^{1}H NMR using 1,3,5-trimethoxybenzene as internal standard. [c]Isolated yield at 0.2 mmol scale.

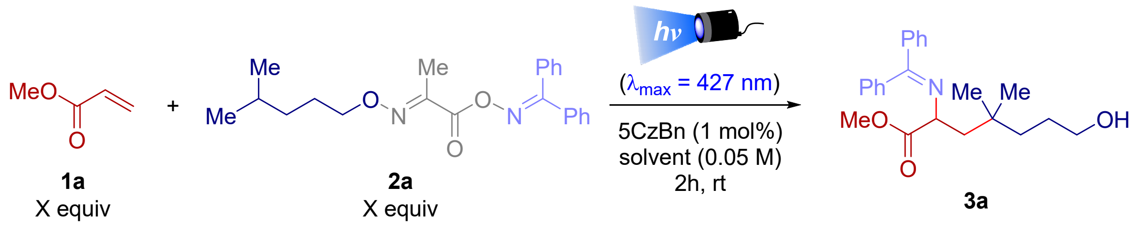

Table S3. Screening of solvents[a]

Entry	Solvent	3a (%)[b]
**1**	acetone	47 (39)[c]
**2**	acetonitrile	10
**3**	ethyl acetate	47
**4**	dichloromethane	18

[a] Optimization reactions were performed using methyl acrylate (**1a**, 0.05 mmol), bifunctional reagent **2a** (2.0 equiv) and 5CzBN (1 mol%), in a dry degassed solvent (1.0 mL, 0.05 M) under blue Kessil irradiation (λ_{max} = 427 nm) for 2 hours at room temperature under inert atmosphere. [b] All the reactions were analyzed by ^{1}H NMR using 1,3,5-trimethoxybenzene as internal standard. [c] Isolated yield at 0.2 mmol scale.

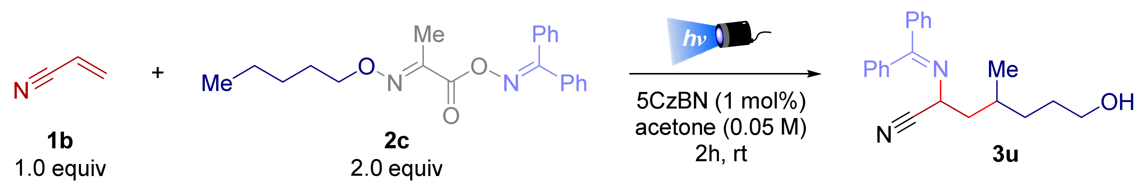

Table S4. Control experiments[a]

Entry	Variation of std. conditions	3a (%)[b]
**1**	no	93[c]
**2**	No 5CzBN	nd
**3**	No light	nd
**4**	Open-to-air	64

[a] Control experiments were performed using acrylonitrile (**1b**, 0.05 mmol), **2c** (2.0 equiv), and 5CzBN (1 mol%), in dry degassed acetone (1.0 mL, 0.05 M) under blue Kessil irradiation (λ_{max} = 427 nm) for 2 hours at room temperature under inert atmosphere. [b] All the reactions were analyzed by ^{1}H NMR using 1,3,5-trimethoxybenzene as internal standard. [c] Isolated yield. Abbreviations: std., standard; nd, no detected.

3.3. General Procedure

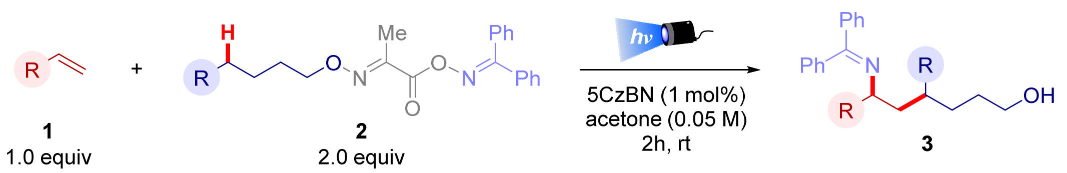

To an 8 mL vial equipped with a magnetic stir bar was added alkene **1** (0.20 mmol, 1.0 equiv, *if solid*), the bifunctional reagent **2** (0.40 mmol, 2.0 equiv), and 5BzCN (1.9 mg, 2.00 µmol, 1 mol%). The vial was sealed with a cap containing a Sil/PTFE septum, evacuated, and backfilled with nitrogen. After this process was repeated 3 times, anhydrous degassed acetone (4.0 mL, 0.05 M), and alkene **1** (0.20 mmol, 1.0 equiv, *if liquid*) were added via syringe. The reaction mixture was irradiated with a Kessil PR160-blue LED lamp (30 W High Luminous DEX 2100 LED, λ_{max} = 427 nm) for 2 h as described in the "Workflow" section. The lamp was placed 4 cm away from the reaction vials, and cooled at room temperature by an external fan. Upon completion, the volatiles were removed under reduced pressure, and the crude mixture was subjected to purification by column chromatography to afford compound **2**.

3.4. Unsuccessful Results:

All the crude mixtures were analyzed by ^{1}H-NMR.

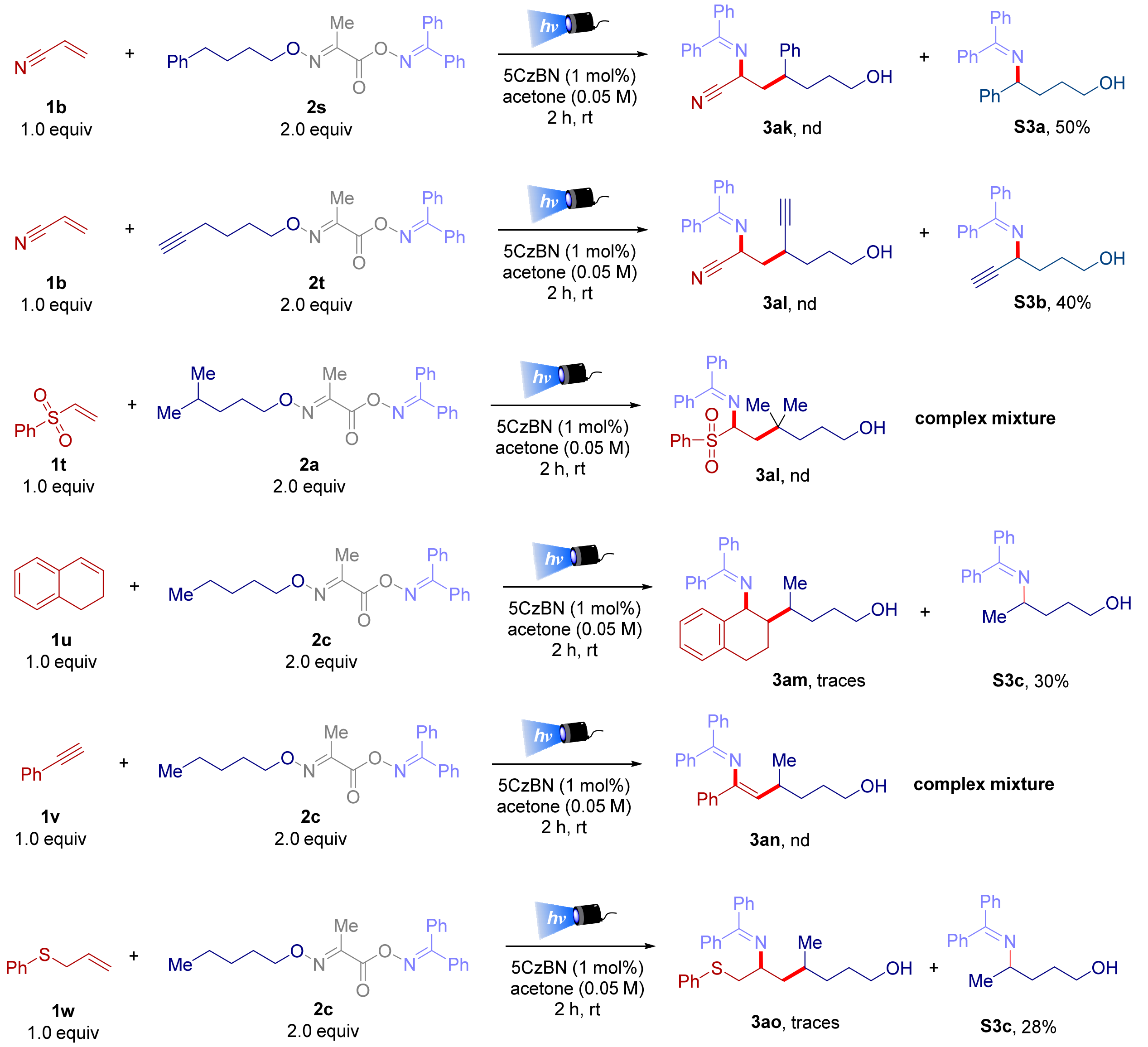

3.5. Characterization Data:

Methyl 2-((diphenylmethylene)amino)-7-hydroxy-4,4-dimethylheptanoate (3a)

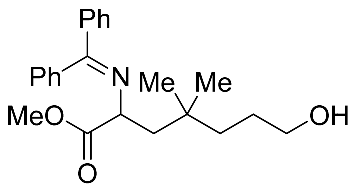

Prepared according to the *General Procedure* from methyl acrylate (17 mg, 0.20 mmol, 1.0 equiv) and bifunctional reagent **2a** (147 mg, 0.40 mmol, 2.0 equiv). After a chromatographic purification (5 – 30% AcOEt in heptane), the title compound **3a** was obtained as a yellow oil (29 mg, 0.08 mmol, 39%). **^{1}H NMR** (300 MHz, $CDCl_3$), δ (ppm)

= 7.66 – 7.59 (m, 2H), 7.51 – 7.42 (m, 3H), 7.39 – 7.28 (m, 3H), 7.22 – 7.16 (m, 2H), 4.19 (dd, *J* = 6.9, 5.3 Hz, 1H), 3.71 (s, 3H), 3.47 (t, *J* = 6.6 Hz, 2H), 2.09 and 1.81 (AB system, Δν = 82.7 Hz, *J* = 14.2, 6.9, 5.3 Hz, 2H), 1.50 – 1.40 (m, 2H), 1.26 (bs, 1H), 1.14 – 1.02 (m, 2H), 0.75 (d, *J* = 1.3 Hz, 6H). **^{13}C{^{1}H} NMR** (75 MHz, $CDCl_3$), δ (ppm) = 173.8, 169.6, 139.7, 136.6, 130.5, 129.0 (2C), 128.9, 128.7 (2C), 128.2 (2C), 128.0 (2C), 63.8, 63.5, 52.3, 45.3, 38.3, 32.9, 27.6, 27.6, 27.5. **HRMS (APCI)** calcd for $C_{23}H_{29}NO_3$ $[M]^+$: 367.2142, found 367.2143.

2-((Diphenylmethylene)amino)-7-hydroxy-4,4-dimethylheptanenitrile (3b)

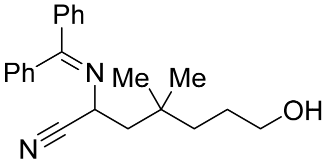

Prepared according to the *General Procedure* from acrylonitrile (11 mg, 0.20 mmol, 1.0 equiv) and bifunctional reagent **2a** (145 mg, 0.40 mmol, 2.0 equiv). After a chromatographic purification (5 – 30% AcOEt in heptane), the title compound **3b** was obtained as a yellow oil (60 mg, 0.18 mmol, 90%). **^{1}H NMR** (300 MHz, $CDCl_3$), δ (ppm) = 7.68 – 7.60 (m, 2H), 7.57 – 7.40 (m, 4H), 7.39 – 7.31 (m, 2H), 7.26 – 7.20 (m, 2H), 4.29 (dd, *J* = 7.2, 6.0 Hz, 1H), 3.51 (t, *J* = 6.5 Hz, 2H), 2.09 and 1.80 (AB system, Δν = 86.5 Hz, *J* = 14.3, 7.2, 6.0 Hz, 2H), 1.58 – 1.40 (m, 3H), 1.23 – 1.10 (m, 2H), 0.82 (d, *J* = 1.8 Hz, 6H). **^{13}C{^{1}H} NMR** (75 MHz, $CDCl_3$), δ (ppm) = 172.2, 138.5, 135.4, 131.2, 129.5, 129.1 (4C), 128.3 (2C), 127.4 (2C), 120.7, 63.4, 50.1, 46.1, 38.1, 32.8, 27.4, 27.4, 27.3. **HRMS (APCI)** calcd for $C_{22}H_{27}N_2O$ $[M+H]^+$: 335.2118, found 335.2118.

Dimethyl 2-((diphenylmethylene)amino)-3-(5-hydroxy-2-methylpentan-2-yl) succinate (3c)

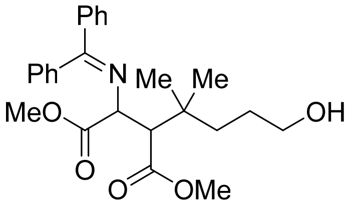

Prepared according to the *General Procedure* from dimethyl fumarate (29 mg, 0.20 mmol, 1.0 equiv) and bifunctional reagent **2a** (145 mg, 0.40 mmol, 2.0 equiv). The title compound was obtained as mixture of diastereomers (1:2) (anti:syn), and after a chromatographic purification (5 – 20% AcOEt in heptane) the inseparable mixture **3c** was obtained as a yellow oil (51 mg, 0.12 mmol, 60%). The combined data for both isomers is detailed down below. **^{1}H NMR** (300 MHz, $CDCl_3$), δ (ppm) = 7.63 – 7.52 (m, 3H), 7.50 – 7.40 (m, 4H), 7.40 – 7.33 (m, 2H), 7.32 – 7.28 (m, 3H), 7.27 – 7.18 (m, 3H), 4.61 (d, *J* = 8.9 Hz, 0.5H), 4.42 (d, *J* = 7.3 Hz, 1H), 3.70 (s, 3H), 3.65 (s, 1.5H), 3.63 (s, 3H), 3.61 (s, 1.5H), 3.56 – 3.44 (m, 4H), 3.35 (d, *J* = 8.9 Hz, 0.5H), 1.61 – 1.45 (m, 4H), 1.36 – 1.19 (m, 3.5H), 0.88 (d, *J* = 1.4 Hz, 3H), 0.85 (d, *J* = 7.0 Hz, 6H). **^{13}C{^{1}H} NMR** (75 MHz, $CDCl_3$), δ (ppm) = 174.0, 172.6, 172.2, 171.6 (2C), 170.4, 139.7 (2C), 136.1, 135.9, 130.7, 130.6, 129.1 (6C), 129.0 (2C), 128.5 (2C), 128.4 (2C), 128.2 (4C), 128.1 (2C), 66.1, 64.8, 63.6, 63.5, 57.1, 56.5, 52.5, 52.4, 51.3 (2C), 37.5, 37.3, 35.8, 35.1, 27.4, 27.1, 26.6, 25.7, 25.2, 25.1. **HRMS (ESI)** calcd for $C_{25}H_{31}NO_5$ $[M+H]^+$: 426.2275, found 426.2273.

Methyl 2-((diphenylmethylene)amino)-7-hydroxy-2,4,4-trimethylheptanoate (3d)

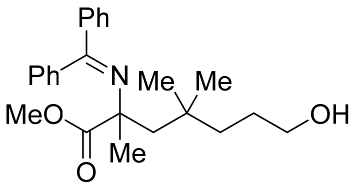

Prepared according to the *General Procedure* from methyl methacrylate (21 mg, 0.20 mmol, 1.0 equiv) and bifunctional reagent **2a** (145 mg, 0.40 mmol, 2.0 equiv). After a chromatographic purification (5 – 20% AcOEt in heptane), the title compound **3d** was obtained as a yellow oil (49 mg, 0.13 mmol, 62%). **^{1}H NMR** (300 MHz, $CDCl_3$), δ (ppm) = 7.58–7.52 (m, 2H), 7.41–7.36 (m, 3H), 7.35 – 7.26 (m, 3H), 7.18 – 7.11 (m, 2H), 3.58 (t, *J* = 6.6 Hz, 2H), 3.36 (s, 3H), 2.17 and 2.02 (AB system, Δν = 44.2 Hz, *J* = 14.4 Hz, 2H), 1.62 – 1.53 (m, 2H), 1.40 (s, 3H), 1.38 – 1.29 (m, 3H), 1.00 (d, *J* = 14.5 Hz, 6H). **^{13}C{^{1}H} NMR** (76 MHz, $CDCl_3$), δ (ppm) = 176.1, 165.2, 141.5, 137.9, 129.9, 128.7 (2C), 128.6 (2C), 128.4, 128.1 (2C), 127.8 (2C), 67.1, 63.9, 53.9, 51.6, 40.7, 33.8, 28.4, 28.2, 27.7, 26.3. **HRMS (APCI)** calcd for $C_{24}H_{32}NO_3$ $[M+H]^+$: 382.2377, found 382.2372.

4-(2-Bromoethyl)phenyl 2-((diphenylmethylene)amino)-7-hydroxy-2,4,4-trimethylheptanoate (3e)

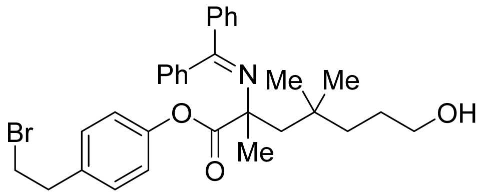

Prepared according to the *General Procedure* from 4-(2-bromoethyl)phenyl methacrylate (54 mg, 0.20 mmol, 1.0 equiv) and bifunctional reagent **2a** (145 mg, 0.40 mmol, 2.0 equiv). After a chromatographic purification (10 – 30% AcOEt in heptane), the title compound **3e** was obtained as a yellow oil (89 mg, 0.16 mmol, 81%). **^{1}H NMR** (300 MHz, $CDCl_3$), δ (ppm) = 7.65 – 7.54 (m, 2H), 7.43 – 7.27 (m, 6H), 7.25 – 7.14 (m, 4H), 6.97 – 6.86 (m, 2H), 3.69 (t, J = 7.4 Hz, 1H), 3.56 (q, J = 7.7, 7.1 Hz, 3H), 3.14 and 3.05 (AB system, Δν = 28.2 Hz, *J* = 7.5 Hz, 2H), 2.30 and 2.04 (AB system, Δν = 80.5 Hz, *J* = 14.4 Hz, 2H), 1.61 – 1.55 (m, 2H), 1.47 – 1.38 (m, 2H), 1.37 (s, 3H), 1.20 (bs, 1H), 1.10 (d, J = 7.1 Hz, 6H). Rotamers were observed in the ^{13}C NMR spectra. **^{13}C{^{1}H} NMR** (75 MHz, $CDCl_3$), δ (ppm) = 174.2, 165.7, 150.0, 141.5, 138.8, 136.2, 135.4, 130.0, 129.7, 129.5, 128.7 (2C), 128.6 (2C), 128.4, 128.1 (2C), 128.0 (2C), 121.5, 121.4, 67.6, 63.8, 53.7, 44.9, 40.7, 38.8, 38.6, 34.1, 32.8, 28.5, 28.1, 27.7, 27.0. **HRMS (APCI)** calcd for $C_{31}H_{37}BrNO_3$ $[M+H]^+$: 550.1951, found 550.1951.

Benzyl 2-((diphenylmethylene)amino)-7-hydroxy-4,4-dimethyl-2-phenylheptanoate (3f)

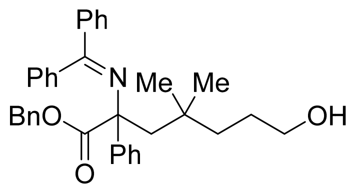

Prepared according to the *General Procedure* from benzyl 2-phenylacrylate (48 mg, 0.20 mmol, 1.0 equiv) and bifunctional reagent **2a** (145 mg, 0.40 mmol, 2.0 equiv). After a chromatographic purification (5 – 20% AcOEt in heptane), the title compound **3f** was obtained as a yellow oil (101 mg, 0.20 mmol, 98%). **^{1}H NMR** (300 MHz, $CDCl_3$), δ (ppm) = 7.80 – 7.71 (m, 2H), 7.69 – 7.56 (m, 2H), 7.47 – 7.21 (m, 10H), 7.21 – 7.10 (m, 4H), 6.83 – 6.73 (m, 2H), 4.70 and 4.39 (AB system, Δν = 93.2 Hz, *J* = 12.8 Hz, 2H), 3.37 (t, *J* = 6.6 Hz, 2H), 2.57and 2.44 (AB system, Δν = 36.4 Hz, *J* = 14.8 Hz, 2H), 1.49 – 1.37 (m, 2H), 1.27 – 1.13 (m, 3H), 0.87 (s, 3H), 0.77 (s, 3H). **^{13}C{^{1}H} NMR** (76 MHz, $CDCl_3$), δ (ppm) = 173.7, 165.5, 144.9, 141.2, 137.0, 135.7, 130.2, 128.8 (2C), 128.7 (2C), 128.5, 128.2 (5C), 128.1 (2C), 127.7, 127.6 (2C), 127.3 (2C), 126.8, 126.5, 71.9, 65.8, 63.8, 50.6, 40.7, 34.1, 29.2, 29.0, 27.6. **HRMS (APCI)** calcd for $C_{35}H_{38}NO_3$ $[M+H]^+$: 520.2846, found 520.2847.

7-((Diphenylmethylene)amino)-12-hydroxy-9,9-dimethyldodecan-6-one (3g)

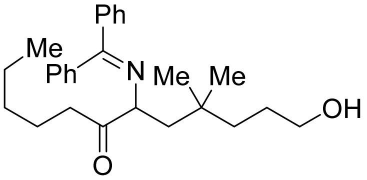

Prepared according to the *General Procedure* from oct-1-en-3-one (25 mg, 0.20 mmol, 1.0 equiv) and bifunctional reagent **2a** (145 mg, 0.40 mmol, 2.0 equiv). After a chromatographic purification (5 – 20% AcOEt in heptane), the title compound **3g** was obtained as a yellow oil (49 mg, 0.12 mmol, 61%). **^{1}H NMR** (300 MHz, $CDCl_3$), δ (ppm) = 7.69 – 7.62 (m, 2H), 7.50 – 7.39 (m, 3H), 7.40 – 7.30 (m, 3H), 7.11 – 7.03 (m, 2H), 4.07 (dd, *J* = 7.9, 4.0 Hz, 1H), 3.47 (t, *J* = 6.6 Hz, 2H), 2.54 (td, *J* = 7.3, 2.9 Hz, 2H), 1.92 and 1.73 (AB system, Δν = 56.2 Hz, *J* = 14.0, 7.9, 4.1 Hz, 2H), 1.56 – 1.43 (m, 5H), 1.33 – 1.20 (m, 4H), 1.14 – 1.06 (m, 2H), 0.86 (t, *J* = 6.9 Hz, 3H), 0.77 (s, 6H). **^{13}C{^{1}H} NMR** (126 MHz, $CDCl_3$), δ (ppm) = 211.7, 168.3, 139.6, 136.6, 130.4, 128.8, 128.7 (2C), 128.7 (2C), 128.2 (2C), 127.8 (2C), 71.1, 63.7, 45.3, 39.4, 38.3, 33.3, 31.6, 27.9, 27.8, 27.5, 23.4, 22.6, 14.0. **HRMS (APCI)** calcd for $C_{27}H_{38}NO_2$ $[M+H]^+$: 408.2897, found 408.2894.

2-((Diphenylmethylene)amino)-7-hydroxy-*N*,2,4,4-tetramethylheptanamide (3h)

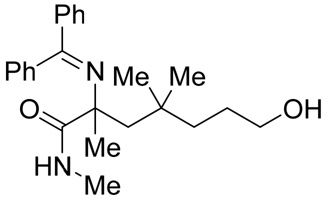

Prepared according to the *General Procedure* from *N*-methylmethacrylamide (19 mg, 0.20 mmol, 1.0 equiv) and bifunctional reagent **2a** (145 mg, 0.40 mmol, 2.0 equiv). After a chromatographic purification (30 – 60% AcOEt in heptane), the title compound **3h** was obtained as a yellow oil (38 mg, 0.10 mmol, 51%). **^{1}H NMR** (500 MHz, $CDCl_3$), δ (ppm) = 8.26 (q, *J* = 5.0 Hz, 1H), 7.54 – 7.49 (m, 2H), 7.45 – 7.38 (m, 4H), 7.37 – 7.32 (m, 2H), 7.26 – 7.19 (m, 2H), 3.65 – 3.57 (m, 1H), 3.52 – 3.42 (m, 1H), 2.93 (d, *J* = 4.9 Hz, 3H), 2.21 (d, *J* = 14.5 Hz, 2H), 1.62 –

1.37 (m, 4H), 1.17 – 1.08 (m, 1H), 1.01 (s, 3H), 0.92 (d, J = 39.7 Hz, 6H). **$^{13}C\{^1H\}$ NMR** (126 MHz, $CDCl_3$), δ (ppm) = 177.8, 165.1, 141.0, 138.8, 130.3, 128.6, 128.4 (2C), 128.2 (2C), 128.1 (4C), 67.1, 63.4, 50.9, 39.2, 34.0, 29.3, 28.7, 28.3, 27.4, 26.7. **HRMS (APCI)** calcd for $C_{24}H_{33}N_2O_2$ $[M+H]^+$: 381.2537, found 381.2541.

2-((Diphenylmethylene)amino)-7-hydroxy-*N*-methoxy-*N*,2,4,4-tetramethylheptanamide (3i)

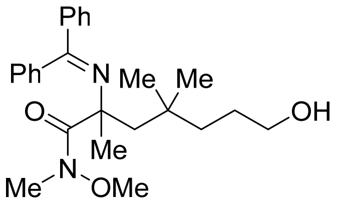

Prepared according to the *General Procedure* from *N*-methoxy-*N*-methylmethacrylamide (26 mg, 0.20 mmol, 1.0 equiv) and bifunctional reagent **2a** (145 mg, 0.40 mmol, 2.0 equiv). After a chromatographic purification (5 – 20% AcOEt in heptane), the title compound **3i** was obtained as a yellow oil (27 mg, 0.07 mmol, 33%). **^1H NMR** (300 MHz, $CDCl_3$), δ (ppm) = 7.65 – 7.53 (m, 2H), 7.43 – 7.26 (m, 6H), 7.21 – 7.10 (m, 2H), 3.57 (t, J = 6.6 Hz, 2H), 3.56 (s, 3H), 3.11 (s, 3H), 2.24 and 1.76 (AB system, Δν = 145.4 Hz, J = 14.4 Hz, 2H), 1.60 – 1.53 (m, 2H), 1.38 – 1.30 (m, 2H), 1.27 (bs, 1H), 1.17 (s, 3H), 1.03 (d, J = 23.3 Hz, 6H). **$^{13}C\{^1H\}$ NMR** (75 MHz, $CDCl_3$), δ (ppm) = 176.3, 163.2, 141.1, 138.8, 129.7, 128.4 (2C), 128.3, 128.0 (4C), 127.9 (2C), 68.5, 63.9 (2C), 60.5, 51.9, 41.0, 33.9, 28.8, 27.9, 27.7, 26.5. **HRMS (APCI)** calcd for $C_{25}H_{35}N_2O_3$ $[M+H]^+$: 411.2642, found 411.2646.

6-((Diphenylmethylene)amino)-4,4-dimethyl-6-(*p*-tolyl)hexan-1-ol (3j)

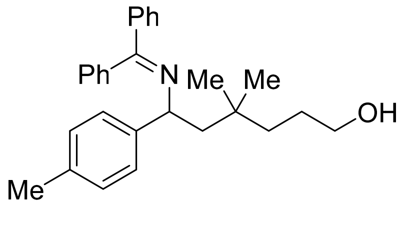

Prepared according to the *General Procedure* from 1-methyl-4-vinylbenzene (23 mg, 0.20 mmol, 1.0 equiv) and bifunctional reagent **2a** (145 mg, 0.40 mmol, 2.0 equiv). After a chromatographic purification (5 – 10% AcOEt in heptane), the title compound **3j** was obtained as a yellow oil (35 mg, 0.09 mmol, 44%). **^1H NMR** (300 MHz, $CDCl_3$), δ (ppm) = 7.69 – 7.61 (m, 2H), 7.45 – 7.37 (m, 3H), 7.37 – 7.28 (m, 3H), 7.17 – 7.10 (m, 2H), 7.10 – 7.05 (m, 2H), 7.05 – 6.98 (m, 2H), 4.46 (dd, J = 8.0, 4.2 Hz, 1H), 3.49 – 3.38 (m, 2H), 2.32 (s, 3H), 2.08 and 1.73 (AB system, Δν = 105.6 Hz, J = 14.2, 8.0, 4.3 Hz, 2H), 1.54 – 1.34 (m, 2H), 1.18 – 1.02 (m, 3H), 0.77 (s, 6H). **$^{13}C\{^1H\}$ NMR** (76 MHz, $CDCl_3$), δ (ppm) = 165.3, 144.1, 140.2, 137.4, 136.0, 129.9, 129.1 (2C), 128.6 (2C), 128.5, 128.3 (2C), 128.1 (2C), 128.0 (2C), 127.2 (2C), 64.0, 63.9, 51.2, 38.3, 33.3, 28.4 (2C), 27.7, 21.2. **HRMS (APCI)** calcd for $C_{28}H_{34}NO$ $[M+H]^+$: 400.2635, found 400.2627.

4-(1-((Diphenylmethylene)amino)-6-hydroxy-3,3-dimethylhexyl)phenyl Acetate (3k)

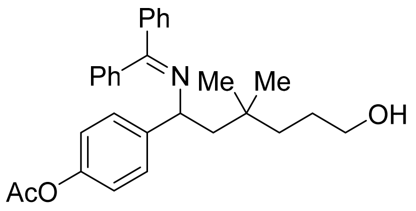

Prepared according to the *General Procedure* from 4-vinylphenyl acetate (33 mg, 0.20 mmol, 1.0 equiv) and bifunctional reagent **2a** (145 mg, 0.40 mmol, 2.0 equiv). After a chromatographic purification (5 – 25% AcOEt in heptane), the title compound **3k** was obtained as a yellow oil (70 mg, 0.16 mmol, 78%). ^{1}**H NMR** (300 MHz, $CDCl_3$), δ (ppm) = 7.65 – 7.58 (m, 2H), 7.42 – 7.35 (m, 3H), 7.34 – 7.20 (m, 5H), 7.03 – 6.92 (m, 4H), 4.46 (dd, *J* = 7.6, 4.6 Hz, 1H), 3.43 – 3.26 (m, 2H), 2.24 (s, 3H), 2.00 and 1.74 (AB system, Δν = 79.3 Hz, *J* = 14.2, 7.6, 4.7 Hz, 2H), 1.52 – 1.37 (m, 2H), 1.30 – 1.23 (m, 1H), 1.11 – 0.96 (m, 2H), 0.74 (d, *J* = 3.6 Hz, 6H). ^{13}**C{^{1}H} NMR** (126 MHz, $CDCl_3$), δ (ppm) = 169.7, 165.7, 149.2, 144.4, 140.0, 137.2, 130.0, 128.6 (2C), 128.5, 128.3 (2C), 128.3 (2C), 128.1 (2C), 127.8 (2C), 121.4 (2C), 63.7, 63.6, 51.1, 38.1, 33.3, 28.4 (2C), 27.6, 21.2. **HRMS (APCI)** calcd for $C_{29}H_{34}NO_3$ $[M+H]^+$: 444.2533, found 444.2534.

6-(3-Bromophenyl)-6-((diphenylmethylene)amino)-4,4-dimethylhexan-1-ol (3l)

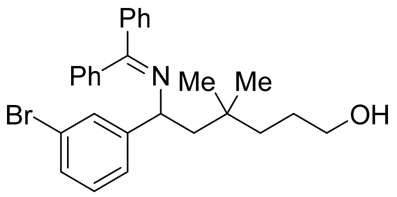

Prepared according to the *General Procedure* from 1-bromo-3-vinylbenzene (37 mg, 0.20 mmol, 1.0 equiv) and bifunctional reagent **2a** (145 mg, 0.40 mmol, 2.0 equiv). After a chromatographic purification (5 – 10% AcOEt in heptane), the title compound **3l** was obtained as a yellow oil (59 mg, 0.13 mmol, 64%). ^{1}**H NMR** (300 MHz, $CDCl_3$), δ (ppm) = 7.71 – 7.64 (m, 2H), 7.47 – 7.29 (m, 8H), 7.17 – 7.10 (m, 2H), 7.07 – 6.95 (m, 2H), 4.45 (dd, J = 8.2, 4.0 Hz, 1H), 3.46 (t, J = 6.6 Hz, 2H), 2.11 and 1.70 (AB system, Δν = 124.0 Hz, *J* = 14.2, 8.2, 4.0 Hz, 2H), 1.58 – 1.37 (m, 2H), 1.28 (bs, 1H), 1.21 – 1.04 (m, 2H), 0.79 (d, J = 1.3 Hz, 6H). ^{13}**C{^{1}H} NMR** (126 MHz, $CDCl_3$), δ (ppm) = 166.3, 149.3, 139.8, 137.1, 130.3, 130.1, 130.1, 129.7, 128.7 (3C), 128.4 (2C), 128.2 (2C), 127.8 (2C), 125.9, 122.5, 63.8, 63.8, 51.2, 38.3, 33.4, 28.3, 28.2, 27.6. **HRMS (APCI)** calcd for $C_{27}H_{31}BrNO$ $[M+H]^+$: 464.1584, found 464.1579.

6-(2-Bromophenyl)-6-((diphenylmethylene)amino)-4,4-dimethylhexan-1-ol (3m)

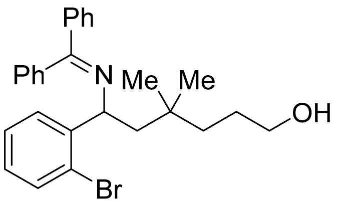

Prepared according to the *General Procedure* from 1-bromo-2-vinylbenzene (37 mg, 0.20 mmol, 1.0 equiv) and bifunctional reagent **2a** (145 mg, 0.40 mmol, 2.0 equiv). After a chromatographic purification (5 – 10% AcOEt in heptane), the title compound **3m** was obtained as a yellow oil (71 mg, 0.15 mmol, 76%). **^{1}H NMR** (300 MHz, $CDCl_3$), δ (ppm) = 7.77 – 7.62 (m, 3H), 7.48 – 7.31 (m, 7H), 7.26 (td, *J* = 7.4, 1.3 Hz, 1H), 7.04 (td, *J* = 7.6, 1.8 Hz, 1H), 6.98 – 6.89 (m, 2H), 4.91 (dd, *J* = 9.3, 2.8 Hz, 1H), 3.50 (t, *J* = 6.7 Hz, 2H), 2.07 (dd, *J* = 14.1, 9.3 Hz, 1H), 1.69 – 1.46 (m, 3H), 1.37 (bs, 1H), 1.28 – 1.10 (m, 2H), 0.86 (d, *J* = 8.3 Hz, 6H). **^{13}C{^{1}H} NMR** (126 MHz, $CDCl_3$), δ (ppm) = 167.0, 146.5, 139.9, 137.3, 132.6, 130.1, 130.0, 128.7 (2C), 128.6 (2C), 128.5, 128.2 (2C), 127.8, 127.7, 127.6 (2C), 121.9, 64.0, 62.7, 50.0, 38.6, 33.6, 28.2 (2C), 27.7. **HRMS (APCI)** calcd for $C_{27}H_{31}BrNO$ $[M+H]^+$: 464.1584, found 464.1589.

6-((Diphenylmethylene)amino)-4,4-dimethyl-6,6-diphenylhexan-1-ol (3n)

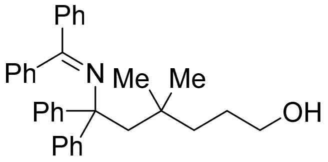

Prepared according to the *General Procedure* from ethene-1,1-diyldibenzene (36 mg, 0.20 mmol, 1.0 equiv) and bifunctional reagent **2a** (145 mg, 0.40 mmol, 2.0 equiv). After a chromatographic purification (5 – 10% AcOEt in heptane), the title compound **3n** was obtained as a yellow oil (64 mg, 0.14 mmol, 70%). **^{1}H NMR** (300 MHz, $CDCl_3$), δ (ppm) = 7.75 – 7.65 (m, 2H), 7.52 – 7.27 (m, 5H), 7.23 – 6.91 (m, 11H), 6.56 – 6.42 (m, 2H), 3.40 (t, *J* = 6.6 Hz, 2H), 2.50 (s, 2H), 1.48 – 1.34 (m, 2H), 1.28 (s, 1H), 1.09 – 1.00 (m, 2H), 0.75 (s, 6H). **^{13}C{^{1}H} NMR** (75 MHz, $CDCl_3$), δ (ppm) = 164.8, 151.3 (2C), 142.2, 138.9, 129.8, 128.4 (4C), 128.1 (4C), 127.6 (4C), 127.5 (2C), 127.2 (2C), 126.6, 125.6 (2C), 68.9, 64.0, 51.0, 40.7, 34.3, 29.3 (2C), 27.7. **HRMS (APCI)** calcd for $C_{33}H_{35}NO$ $[M]^+$: 461.2713, found 461.2712.

(8*R*,9*S*,13*S*,14*S*)-13-Methyl-17-oxo-7,8,9,11,12,13,14,15,16,17-decahydro-6*H*-cyclopenta[*a*]phenanthren-3-yl 2-((diphenylmethylene)amino)-7-hydroxy-2,4,4-trimethylheptanoate (3o)

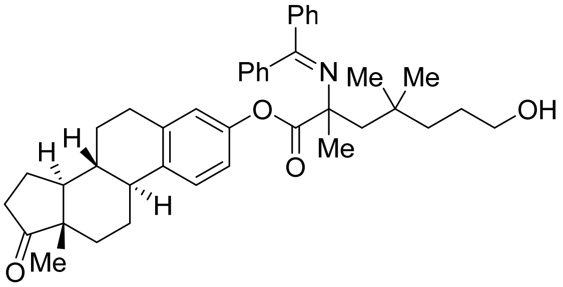

Prepared according to the *General Procedure* from estrone methacrylate derivative **1g** (68 mg, 0.20 mmol, 1.0 equiv) and bifunctional reagent **2a** (145 mg, 0.40 mmol, 2.0 equiv). After a chromatographic purification (10 – 30% AcOEt in heptane), the title compound **3o** was obtained as a white solid (74 mg, 0.12 mmol, 60%). **mp** = 58 – 60 ºC **^{1}H NMR** (300 MHz, $CDCl_3$), δ (ppm) = 7.63 – 7.56 (m, 2H), 7.44 – 7.37 (m, 3H), 7.36 – 7.27 (m, 3H), 7.25 – 7.21 (m, 2H), 6.73 (dd, *J* = 8.5, 2.0 Hz, 1H), 6.63 (dd, *J* = 3.8, 2.5 Hz, 1H), 3.57 (t, *J* = 6.5 Hz, 2H), 2.94 – 2.84 (m, 2H), 2.51 (dd, *J* = 18.3, 8.4 Hz, 1H), 2.43 – 2.36 (m, 1H), 2.35 – 2.21 (m, 2H), 2.17 – 1.95 (m, 5H), 1.65 – 1.32 (m, 15H), 1.10 (d, *J* = 6.9 Hz, 6H), 0.91 (s, 3H). **^{13}C{^{1}H} NMR** (126 MHz, $CDCl_3$), δ (ppm) = 220.9, 174.4, 165.6, 148.9, 141.5, 138.8, 137.8, 137.1, 129.9, 128.7 (2C), 128.6 (2C), 128.4, 128.1 (2C), 128.0 (2C), 126.2, 121.4, 118.5, 67.5, 63.8, 53.8, 50.5, 48.0, 44.2, 40.7, 38.1, 35.9, 34.1, 31.6, 29.5, 28.5, 28.1, 27.7, 27.0, 26.4, 25.8, 21.7, 13.9. **HRMS (APCI)** calcd for $C_{41}H_{49}NO_4$ $[M]^+$: 619.3656, found 619.3656.

2-Methoxy-4-(3-oxobutyl)phenyl 2-((diphenylmethylene)amino)-7-hydroxy-2,4,4-trimethylheptanoate (3p)

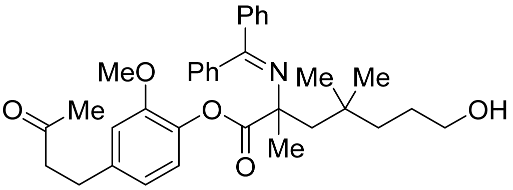

Prepared according to the *General Procedure* from 2-methoxy-4-(3-oxobutyl)phenyl methacrylate (53 mg, 0.20 mmol, 1.0 equiv) and bifunctional reagent **2a** (145 mg, 0.40 mmol, 2.0 equiv). After a chromatographic purification (10 – 40% AcOEt in heptane), the title compound **3p** was obtained as a yellow oil (81 mg, 0.15 mmol, 75%). **^{1}H NMR** (300 MHz, $CDCl_3$), δ (ppm) = 7.66 – 7.58 (m, 2H), 7.44 – 7.36 (m, 3H), 7.35 – 7.27 (m, 4H), 7.26 – 7.25 (m, 1H), 6.81 (d, *J* = 8.0 Hz, 1H), 6.76 – 6.69 (m, 2H), 3.74 (s, 3H), 3.57 (t, *J* = 6.5 Hz, 2H), 2.90 – 2.84 (m, 2H), 2.78 – 2.71 (m, 2H), 2.38 and 1.92 (AB system, Δν = 138.6 Hz, *J* = 14.5 Hz, 2H), 2.14 (s, 3H), 1.67 – 1.51 (m, 3H), 1.47 – 1.39 (m, 2H), 1.28 (s, 3H), 1.12 (d, *J* = 8.3 Hz, 6H). **^{13}C{^{1}H} NMR** (76 MHz, $CDCl_3$), δ (ppm) = 207.9, 174.2, 165.3, 151.1, 141.6, 139.7, 139.3, 138.5, 129.8, 128.7 (2C), 128.5 (2C), 128.2, 128.0 (2C), 127.9 (2C), 122.6, 120.2, 112.8, 68.0, 63.9, 55.8, 52.9, 45.2, 40.7, 34.2, 30.2, 29.7, 28.8, 27.9, 27.7, 27.2. **HRMS (APCI)** calcd for $C_{34}H_{42}NO_5$ $[M+H]^+$: 544.3057, found 544.3059.

4-(1-((Diphenylmethylene)amino)-6-hydroxy-3,3-dimethylhexyl)benzyl 2-(2-Fluoro-[1,1'-biphenyl]-4-yl)propanoate (3q)

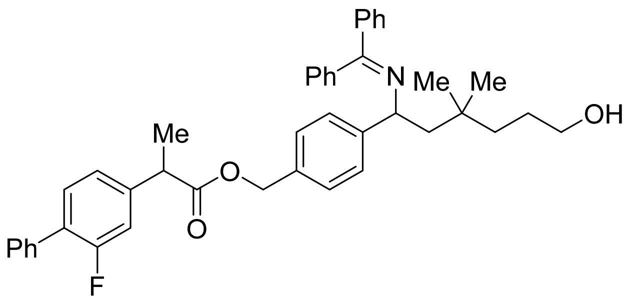

Prepared according to the *General Procedure* from flurbiprofen styrene derivative **1r** (72 mg, 0.20 mmol, 1.0 equiv) and bifunctional reagent **2a** (145 mg, 0.40 mmol, 2.0 equiv). The title compound was obtained as mixture of diastereomers (1:1), and after a chromatographic purification (5 – 15% AcOEt in heptane) the inseparable mixture **3q** was obtained as a yellow oil (111 mg, 0.17 mmol, 87%). The combined data for both isomers is detailed down below. **^{1}H NMR** (300 MHz, $CDCl_3$), δ (ppm) = 7.74 – 7.61 (m, 2H), 7.59 – 7.50 (m, 2H), 7.48 – 7.27 (m, 10H), 7.26 – 7.10 (m, 6H), 7.07 – 6.94 (m, 2H), 5.16 and 5.09 (AB system, Δν = 24.1 Hz, *J* = 12.4, 1.9 Hz, 2H), 4.50 (dd, *J* = 8.0, 4.2 Hz, 1H), 3.81 (q, *J* = 7.2 Hz, 1H), 3.43 (t, *J* = 6.6 Hz, 2H), 2.10 and 1.74 (AB system, Δν = 107.4 Hz, *J* = 14.2, 8.0, 4.0 Hz, 2H),1.56 (d, *J* = 7.1 Hz, 3H), 1.40 – 1.28 (m, 3H), 1.19 – 1.03 (m, 2H), 0.78 (s, 6H). **^{13}C{^{1}H} NMR** (75 MHz, $CDCl_3$), δ (ppm) = 173.9 (2C), 165.7 (d, $J_{C\text{-}F}$ = 1.2 Hz, 2C), 159.8 (d, $J_{C\text{-}F}$ = 248.4 Hz, 2C), 147.1 (2C), 141.9 (d, $J_{C\text{-}F}$ = 7.7 Hz, 2C), 140.0 (2C), 137.3 (2C), 135.6 (4C), 134.0 (2C), 130.9 (d, $J_{C\text{-}F}$ = 3.9 Hz, 2C), 130.0 (2C), 129.1 (d, $J_{C\text{-}F}$ = 2.9 Hz, 4C), 128.6 (4C), 128.5 (8C), 128.3 (4C), 128.2 (2C), 128.1 (4C), 127.9 (4C), 127.8 (2C), 127.4 (4C), 123.7 (d, $J_{C\text{-}F}$ = 1.7 Hz, 2C), 115.6 (d, $J_{C\text{-}F}$ = 1.7 Hz), 115.3 (d, $J_{C\text{-}F}$ = 1.6 Hz), 66.7 (2C), 64.0 (2C), 63.8 (2C), 51.1 (2C), 45.2 , 45.1, 38.2 (2C), 33.3 (4C), 28.4, 28.3, 27.6 (2C), 18.5 (2C). **^{19}F{^{1}H} NMR** (282 MHz, $CDCl_3$) δ (ppm) = -117.51 (s, 1F), -117.52 (s, 1F). **HRMS (APCI)** calcd for $C_{43}H_{45}FNO_3$ $[M+H]^+$: 642.3365, found 642.3371.

4-(1-((Diphenylmethylene)amino)-6-hydroxy-3,3-dimethylhexyl)benzyl 5-(2,5-Dimethylphenoxy)-2,2-dimethylpentanoate (3r)

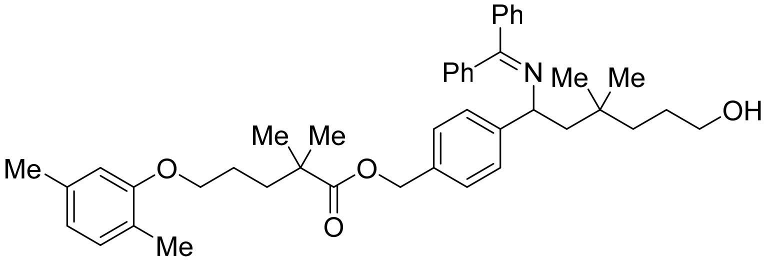

Prepared according to the *General Procedure* from gemfibrozil styrene derivative **1s** (73 mg, 0.20 mmol, 1.0 equiv) and bifunctional reagent **2a** (145 mg, 0.40 mmol, 2.0 equiv). After a chromatographic purification (5 – 20% AcOEt in heptane), the title compound **3r** was obtained as a yellow oil (95 mg, 0.15 mmol, 73%). **^{1}H NMR** (300 MHz, $CDCl_3$), δ (ppm) = 7.71 – 7.59 (m, 2H), 7.44 – 7.28 (m, 6H), 7.25 – 7.22 (m, 4H), 7.07 – 6.91 (m, 3H), 6.68 – 6.61

(m, 1H), 6.61 – 6.56 (m, 1H), 5.08 (s, 2H), 4.49 (dd, *J* = 8.0, 4.2 Hz, 1H), 3.93 – 3.84 (m, 2H), 3.43 (q, *J* = 5.9 Hz, 2H), 2.29 (s, 3H), 2.14 (s, 3H), 2.12 – 2.03 (m, 1H), 1.78 – 1.67 (m, 5H), 1.53 – 1.35 (m, 2H), 1.24 (s, 6H), 1.16 – 1.08 (m, 2H), 0.77 (s, 6H). **$^{13}C\{^1H\}$ NMR** (75 MHz, $CDCl_3$), δ (ppm) = 177.8, 165.7, 157.1, 146.9, 140.0, 137.3, 136.5, 134.5, 130.4, 130.0, 128.6 (2C), 128.5, 128.3 (2C), 128.1 (2C), 128.0 (2C), 127.9 (2C), 127.4 (2C), 123.7, 120.8, 112.1, 68.1, 66.2, 64.0, 63.8, 51.2, 42.3, 38.3, 37.3, 33.3, 28.4, 28.3, 27.6, 25.3 (3C), 21.5, 15.9. **HRMS (APCI)** calcd for $C_{43}H_{54}NO_4$ $[M+H]^+$: 648.4047, found 648.4044.

Methyl 1-((diphenylmethylene)amino)-3-(5-hydroxy-2-methylpentan-2-yl)-3-phenylcyclobutane-1-carboxylate (3s)

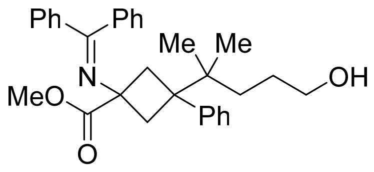

Prepared according to the *General Procedure* from methyl 3-phenylbicyclo[1.1.0]butane-1-carboxylate (38 mg, 0.20 mmol, 1.0 equiv) and bifunctional reagent **2a** (145 mg, 0.40 mmol, 2.0 equiv). The title compound was obtained as mixture of diastereomers (1:3), and after a chromatographic purification (5 – 20% AcOEt in heptane) the inseparable mixture **3s** was obtained as a yellow oil (17 mg, 0.04 mmol, 19%). The combined data for both isomers is detailed down below. **^1H NMR** (300 MHz, $CDCl_3$), δ (ppm) = 7.62 – 7.51 (m, 2H), 7.38 – 7.26 (m, 4H), 7.23 – 7.06 (m, 5.6H), 6.95 – 6.83 (m, 1.4H), 6.79 – 6.66 (m, 0.6H), 6.64 – 6.53 (m, 1.3H), 3.70 (s, 2.3H), 3.61 – 3.49 (m, 2H), 3.30 (s, 0.7H), 3.06 – 2.95 (m, 1.7H), 2.85 – 2.80 (m, 0.3H), 2.77 – 2.65 (m, 2H), 1.59 – 1.42 (m, 3H), 1.26 – 1.15 (m, 2H), 0.95 – 0.80 (m, 6H). **$^{13}C\{^1H\}$ NMR** (76 MHz, $CDCl_3$), δ (ppm) = 177.5, 177.0, 168.7, 165.7, 148.0 (2C), 141.3 (2C), 138.4 (2C), 129.9 (2C), 128.4 (4C), 128.1 (4C), 128.0 (4C), 127.9 (4C), 127.7 (2C), 127.6 (4C), 126.8 (2C), 125.8 (4C), 63.8 (2C), 63.0 (2C), 52.3 (2C), 51.7 (2C), 43.3 (2C), 36.3 (2C), 35.7 (2C), 28.0 (2C), 23.4 (6C). **HRMS (APCI)** calcd for $C_{31}H_{36}NO_3$ $[M+H]^+$: 470.2690, found 470.2689.

2-((Diphenylmethylene)amino)-7-hydroxyheptanenitrile (3t)

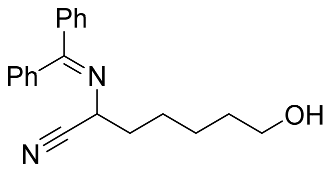

Prepared according to the *General Procedure* from acrylonitrile (11 mg, 0.20 mmol, 1.0 equiv) and bifunctional reagent **2b** (134 mg, 0.40 mmol, 2.0 equiv). After a chromatographic purification (10 – 25% AcOEt in heptane), the title compound **3t** was obtained as a yellow oil (41 mg, 0.13 mmol, 67%). **^1H NMR** (300 MHz, $CDCl_3$), δ (ppm) = 7.72 – 7.58 (m, 2H), 7.56 – 7.41 (m, 4H), 7.39 – 7.31 (m, 2H), 7.24 – 7.14 (m, 2H), 4.22 (t, *J* = 6.8 Hz, 1H), 3.60 (t, *J* = 6.4 Hz, 2H), 2.06 – 1.79 (m, 2H), 1.59 – 1.29 (m, 7H). **$^{13}C\{^1H\}$ NMR** (75 MHz, $CDCl_3$), δ (ppm) = 172.9,

138.6, 135.4, 131.3, 129.5, 129.1 (4C), 128.4 (2C), 127.5 (2C), 119.8, 62.7, 53.1, 34.9, 32.5, 25.5, 25.3. **HRMS (ESI)** calcd for $C_{20}H_{23}N_2O$ [M+H]$^+$: 307.1805, found 307.1798.

2-((Diphenylmethylene)amino)-7-hydroxy-4-methylheptanenitrile (3u)

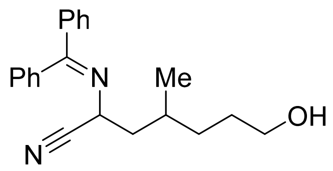

Prepared according to the *General Procedure* from acrylonitrile (11 mg, 0.20 mmol, 1.0 equiv) and bifunctional reagent **2c** (140 mg, 0.40 mmol, 2.0 equiv). The title compound was obtained as mixture of diastereomers (1:1), and after a chromatographic purification (5 – 20% AcOEt in heptane) the inseparable mixture **3u** was obtained as a yellow oil (59 mg, 0.19 mmol, 93%). The combined data for both isomers is detailed down below. **^{1}H NMR** (300 MHz, $CDCl_3$), δ (ppm) = 7.67 – 7.59 (m, 2H), 7.54 – 7.40 (m, 4H), 7.38 – 7.31 (m, 2H), 7.25 – 7.15 (m, 2H), 4.38 – 4.20 (m, 1H), 3.61 – 3.51 (m, 2H), 2.11 – 1.93 (m, 1H), 1.85 – 1.68 (m, 1H), 1.67 – 1.41 (m, 4H), 1.33 – 1.23 (m, 1H), 1.19 – 1.08 (m, 1H), 0.76 (t, J = 6.4 Hz, 3H). **^{13}C{^{1}H} NMR** (75 MHz, $CDCl_3$), δ (ppm) = 173.1, 172.8, 138.5, 138.5, 135.4, 135.3, 131.3, 131.2, 129.5 (2C), 129.1 (4C), 129.0 (4C), 128.3 (4C), 127.5 (2C), 127.4 (2C), 120.1, 120.0, 62.9 (2C), 51.8, 51.2, 41.9, 41.8, 32.6 (2C), 29.9, 29.8, 29.7, 29.3, 19.4, 19.2. **HRMS (ESI)** calcd for $C_{21}H_{25}N_2O$ [M+H]$^+$: 321.1961, found 321.1961.

4-(2-Chloroethyl)-2-((diphenylmethylene)amino)-7-hydroxyheptanenitrile (3v)

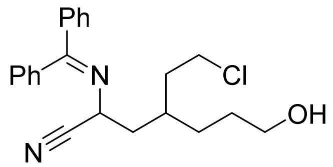

Prepared according to the *General Procedure* from acrylonitrile (11 mg, 0.20 mmol, 1.0 equiv) and bifunctional reagent **2d** (134 mg, 0.40 mmol, 2.0 equiv). The title compound was obtained as mixture of diastereomers (1:1), and after a chromatographic purification (5 – 25% AcOEt in heptane) the inseparable mixture **3v** was obtained as a yellow oil (58 mg, 0.16 mmol, 79%). The combined data for both isomers is detailed down below. **^{1}H NMR** (300 MHz, $CDCl_3$), δ (ppm) = 7.69 – 7.59 (m, 2H), 7.57 – 7.50 (m, 3H), 7.48 – 7.42 (m, 1H), 7.40 – 7.32 (m, 2H), 7.26 – 7.21 (m, 2H), 4.29 (t, J = 7.1 Hz, 1H), 3.59 – 3.44 (m, 4H), 2.02 – 1.74 (m, 3H), 1.72 – 1.59 (m, 2H), 1.53 – 1.19 (m, 5H). **^{13}C{^{1}H} NMR** (75 MHz, $CDCl_3$), δ (ppm) = 173.4 (2C), 138.5, 138.4, 135.3 (2C), 131.4 (2C), 129.7, 129.6, 129.2 (6C), 129.1 (2C), 128.4 (4C), 127.6 (2C), 127.5 (2C), 119.9, 119.7, 62.8, 62.8, 51.4, 51.3, 42.4, 42.3, 38.7, 38.5, 36.5, 36.3, 32.1, 32.0, 29.2, 29.0, 28.9, 28.8. **HRMS (APCI)** calcd for $C_{22}H_{26}ClN_2O$ [M+H]$^+$: 369.1728, found 369.1725.

4-(2-Azidoethyl)-2-((diphenylmethylene)amino)-7-hydroxyheptanenitrile (3w)

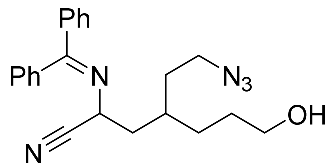

Prepared according to the *General Procedure* from acrylonitrile (11 mg, 0.20 mmol, 1.0 equiv) and bifunctional reagent **2e** (162 mg, 0.40 mmol, 2.0 equiv). The title compound was obtained as mixture of diastereomers (1:1), and after a chromatographic purification (5 – 20% AcOEt in heptane) the inseparable mixture **3w** was obtained as a yellow oil (58 mg, 0.15 mmol, 77%). The combined data for both isomers is detailed down below. **^{1}H NMR** (300 MHz, $CDCl_3$), δ (ppm) = 7.68 – 7.56 (m, 2H), 7.55 – 7.42 (m, 4H), 7.39 – 7.32 (m, 2H), 7.25 – 7.13 (m, 2H), 4.38 – 4.18 (m, 1H), 3.60 – 3.47 (m, 2H), 3.31 – 3.17 (m, 2H), 2.01 – 1.80 (m, 2H), 1.76 – 1.56 (m, 2H), 1.53 – 1.40 (m, 4H), 1.34 – 1.20 (m, 2H). **^{13}C{^{1}H} NMR** (75 MHz, $CDCl_3$), δ (ppm) = 173.3 (2C), 138.4, 138.3, 135.2 (2C), 131.4 (2C), 129.7, 129.6, 129.2 (4C), 129.1 (4C), 128.4 (4C), 127.5 (4C), 119.8, 119.7, 62.7, 62.6, 51.4, 51.4, 49.1, 48.9, 38.8, 38.6, 32.5 (2C), 32.0, 31.9, 29.3, 29.2, 29.0, 28.9. **HRMS (APCI)** calcd for $C_{22}H_{26}N_5O$ $[M+H]^+$: 376.2132, found 376.2130.

Methyl 3-(2-Cyano-2-((diphenylmethylene)amino)ethyl)-6-hydroxyhexanoate (3x)

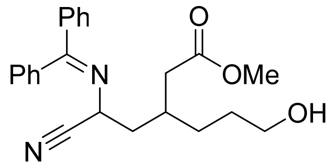

Prepared according to the *General Procedure* from acrylonitrile (11 mg, 0.20 mmol, 1.0 equiv) and bifunctional reagent **2f** (163 mg, 0.40 mmol, 2.0 equiv). The title compound was obtained as mixture of diastereomers (1:1), and after a chromatographic purification (10 – 30% AcOEt in heptane) the inseparable mixture **3x** was obtained as a yellow oil (68 mg, 0.18 mmol, 91%). The combined data for both isomers is detailed down below. **^{1}H NMR** (300 MHz, $CDCl_3$), δ (ppm) = 7.67 – 7.56 (m, 2H), 7.54 – 7.40 (m, 4H), 7.38 – 7.32 (m, 2H), 7.25 – 7.17 (m, 2H), 4.37 – 4.21 (m, 1H), 3.62 (s, 1.5H), 3.59 (s, 1.5H), 3.60 – 3.49 (m, 2H), 2.33 – 2.13 (m, 2H), 2.12 – 1.86 (m, 3H), 1.78 – 1.58 (m, 1H), 1.56 – 1.40 (m, 2H), 1.38 – 1.24 (m, 2H). **^{13}C{^{1}H} NMR** (75 MHz, $CDCl_3$), δ (ppm) = 173.4, 173.3, 172.8 (2C), 138.4, 138.3, 135.2 (2C), 131.4, 131.3, 129.6, 129.5, 129.1 (8C), 128.4 (4C), 127.5 (4C), 119.7, 119.6, 62.5 (2C), 51.7 (2C), 51.5, 51.3, 39.1, 38.5, 38.4, 38.1, 31.8, 31.5, 29.8, 29.7, 29.4, 29.2. **HRMS (APCI)** calcd for $C_{23}H_{27}N_2O_3$ $[M+H]^+$: 379.2016, found 379.2014.

2-((Diphenylmethylene)amino)-7-hydroxy-4-(2-(2-isopropyl-5-methylphenoxy)ethyl)heptanenitrile (3y)

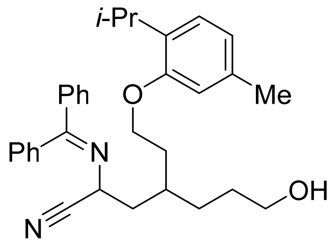

Prepared according to the *General Procedure* from acrylonitrile (11 mg, 0.20 mmol, 1.0 equiv) and bifunctional reagent **2g** (204 mg, 0.40 mmol, 2.0 equiv). The title compound was obtained as mixture of diastereomers (1:1), and after a chromatographic purification (5 – 20% AcOEt in heptane) the inseparable mixture **3y** was obtained as a yellow oil (74 mg, 0.15 mmol, 77%). The combined data for both isomers is detailed down below. **^{1}H NMR** (300 MHz, $CDCl_3$), δ (ppm) = 7.70 – 7.60 (m, 2H), 7.57 – 7.43 (m, 3H), 7.46 – 7.26 (m, 3H), 7.26 – 7.20 (m, 2H), 7.13 – 7.04 (m, 1H), 6.81 – 6.70 (m, 1H), 6.63 (s, 1H), 4.43 – 4.28 (m, 1H), 4.02 – 3.88 (m, 2H), 3.57 (t, *J* = 6.3 Hz, 2H), 3.29 – 3.11 (m, 1H), 2.35 (s, 1.5H), 2.32 (s, 1.5H), 2.18 – 1.76 (m, 4H), 1.78 – 1.63 (m, 2H), 1.63 – 1.47 (m, 2H), 1.41 – 1.28 (m, 2H), 1.19 – 1.09 (m, 6H). **^{13}C{^{1}H} NMR** (76 MHz, $CDCl_3$), δ (ppm) = 173.2 (2C), 157.0, 155.9, 138.4 (2C), 136.3 (2C), 135.3 (2C), 134.1, 134.0, 131.3 (2C), 129.6, 129.5, 129.2 (2C), 129.1 (6C), 128.4 (2C), 128.3 (2C), 127.5 (4C), 125.9 (2C), 121.2 (2C), 120.0, 119.8, 112.2, 112.1, 65.3, 65.2, 62.9 (2C), 51.5, 51.3, 39.3, 38.9, 33.3, 32.8, 31.6, 31.5, 29.4, 29.2, 29.1, 29.0, 26.7, 26.5, 23.0, 22.9, 22.8 (2C), 21.5, 21.4. **HRMS (ESI)** calcd for $C_{32}H_{39}N_2O_2$ $[M+H]^+$: 483.3006, found 483.3000.

2-((Diphenylmethylene)amino)-7-hydroxy-4-(2-(6-(trifluoromethyl)pyridin-2-yl)ethyl)heptanenitrile (3z)

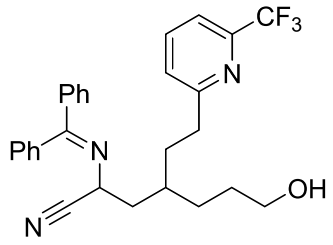

Prepared according to the *General Procedure* from acrylonitrile (11 mg, 0.20 mmol, 1.0 equiv) and bifunctional reagent **2h** (203 mg, 0.40 mmol, 2.0 equiv). The title compound was obtained as mixture of diastereomers (1:1), and after a chromatographic purification (10 – 30% AcOEt in heptane) the inseparable mixture **3z** was obtained as a yellow oil (72 mg, 0.15 mmol, 75%). The combined data for both isomers is detailed down below. **^{1}H NMR** (500 MHz, $CDCl_3$), δ (ppm) = 7.75 – 7.64 (m, 1H), 7.64 – 7.54 (m, 2H), 7.52 – 7.39 (m, 5H), 7.37 – 7.32 (m, 2H), 7.29 – 7.20 (m, 1H), 7.21 – 7.10 (m, 2H), 4.38 – 4.23 (m, 1H), 3.57 – 3.49 (m, 2H), 2.86 – 2.68 (m, 2H), 2.10 – 1.96 (m, 1H), 1.92 – 1.79 (m, 1H), 1.70 – 1.23 (m, 8H). **^{13}C{^{1}H} NMR** (126 MHz, $CDCl_3$), δ (ppm) = 173.1 (2C), 162.9,

162.8, 147.8 (q, J_{C-F} = 34.1 Hz, 2C), 138.4 (2C), 137.6 (2C), 135.3, 135.2, 131.3 (2C), 129.5 (2C), 129.2 (4C), 129.1 (4C), 128.4 (4C), 127.5 (4C), 125.7 (2C), 121.7 (q, J_{C-F} = 273.3 Hz, 2C), 120.0, 119.9, 117.8 (q, J_{C-F} = 2.9 Hz, 2C), 62.8 (2C), 51.6, 51.5, 38.9, 38.7, 35.0, 34.6, 33.9, 33.7, 32.9 (2C), 29.4, 29.1, 29.0 (2C). **$^{19}F\{^1H\}$ NMR** (471 MHz, $CDCl_3$) δ (ppm) = -68.0 (s, 3F). **HRMS (APCI)** calcd for $C_{28}H_{29}F_3N_3O$ $[M+H]^+$: 480.2257, found 480.2246.

2-((Diphenylmethylene)amino)-7-hydroxy-4-(2-(thiophen-2-yl)ethyl)heptanenitrile (3aa)

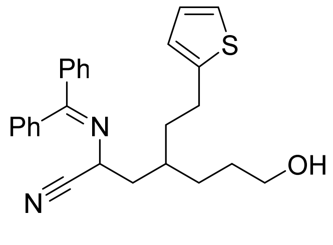

Prepared according to the *General Procedure* from acrylonitrile (11 mg, 0.20 mmol, 1.0 equiv) and bifunctional reagent **2i** (178 mg, 0.40 mmol, 2.0 equiv). The title compound was obtained as mixture of diastereomers (1:1), and after a chromatographic purification (10 – 30% AcOEt in heptane) the inseparable mixture **3aa** was obtained as a yellow oil (34 mg, 0.13 mmol, 40%). The combined data for both isomers is detailed down below. **^1H NMR** (500 MHz, $CDCl_3$), δ (ppm) = 7.67 – 7.56 (m, 2H), 7.52 – 7.42 (m, 4H), 7.38 – 7.34 (m, 2H), 7.24 – 7.17 (m, 2H), 7.11 – 7.05 (m, 1H), 6.91 – 6.83 (m, 1H), 6.74 – 6.66 (m, 1H), 4.33 – 4.26 (m, 1H), 3.59 – 3.48 (m, 2H), 2.87 – 2.67 (m, 2H), 2.06 – 1.96 (m, 1H), 1.94 – 1.82 (m, 1H), 1.71 – 1.61 (m, 1H), 1.58 – 1.48 (m, 3H), 1.41 – 1.19 (m, 4H). **$^{13}C\{^1H\}$ NMR** (126 MHz, $CDCl_3$), δ (ppm) = 173.1 (2C) 145.0, 144.90, 138.5 (2C), 135.4, 135.3, 131.4 (2C), 129.6, 129.5, 129.2 (4C), 129.1 (4C), 128.4 (4C), 127.5 (4C), 126.9, 126.8, 124.3 (2C), 123.1 (2C), 120.0, 119.9, 63.0 (2C), 51.6, 51.5, 38.9, 38.7, 35.5 (2C), 33.9, 33.6, 29.4, 29.2, 29.1, 29.0, 27.0, 26.7. **HRMS (APCI)** calcd for $C_{26}H_{29}N_2OS$ $[M+H]^+$: 417.1995, found 417.1988.

2-((Diphenylmethylene)amino)-3-(2-(2-hydroxyethyl)cyclohexyl)propanenitrile (3ab)

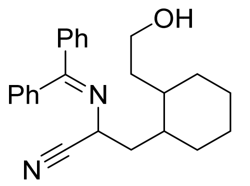

Prepared according to the *General Procedure* from acrylonitrile (11 mg, 0.20 mmol, 1.0 equiv) and bifunctional reagent **2j** (156 mg, 0.40 mmol, 2.0 equiv). The title compound was obtained as mixture of diastereomers (1:1:1:1), and after a chromatographic purification (5 – 25% AcOEt in heptane) the inseparable mixture **3ab** was obtained as a yellow oil (49 mg, 0.14 mmol, 68%). The combined data for both isomers is detailed down below. **^1H NMR** (300 MHz, $CDCl_3$), δ (ppm) = 7.69 – 7.57 (m, 2H), 7.57 – 7.41 (m, 4H), 7.42 – 7.29 (m, 2H), 7.27 – 7.13 (m, 2H), 4.40 – 4.17 (m, 1H), 3.75 – 3.44 (m, 2H), 2.45 – 2.33 (m, 0.5H), 1.90 – 1.00 (m, 13.5H), 0.95 – 0.74 (m, 1H). **$^{13}C\{^1H\}$ NMR** (76 MHz, $CDCl_3$), δ (ppm) = 173.2, 172.4, 138.5 (2C), 135.5, 135.3, 131.3, 131.2, 129.5 (2C), 129.1 (8C),

128.4 (4C), 127.6 (2C), 127.4 (2C), 120.4, 119.9, 60.7 (2C), 52.1, 50.9, 38.9, 38.8, 38.4, 38.3, 37.9 (2C), 36.5, 36.4, 31.8, 31.7, 31.6, 31.5, 28.5, 28.4, 25.7, 25.6. **HRMS (APCI)** calcd for $C_{24}H_{29}N_2O$ $[M+H]^+$: 361.2274, found 361.2278.

2-((Diphenylmethylene)amino)-3-((1*r*,3*s*,5*R*,7*S*)-1-(2-hydroxyethyl)adamantan-2-yl)propanenitrile (3ac)

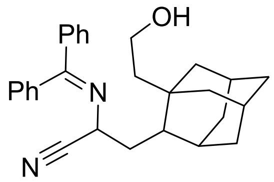

Prepared according to the *General Procedure* from acrylonitrile (11 mg, 0.20 mmol, 1.0 equiv) and bifunctional reagent **2k** (176 mg, 0.40 mmol, 2.0 equiv). The title compound was obtained as mixture of diastereomers (1:1:1:1), and after a chromatographic purification (5 – 25% AcOEt in heptane) the inseparable mixture **3ac** was obtained as a yellow oil (46 mg, 0.11 mmol, 56%). The combined data for both isomers is detailed down below. **^{1}H NMR** (300 MHz, $CDCl_3$), δ (ppm) = 7.71 – 7.61 (m, 2H), 7.55 – 7.41 (m, 4H), 7.43 – 7.30 (m, 2H), 7.25 – 7.13 (m, 2H), 4.36 – 4.15 (m, 1H), 3.81 – 3.56 (m, 2H), 2.37 – 2.18 (m, 1H), 1.92 – 1.28 (m, 18H). **^{13}C{^{1}H} NMR** (76 MHz, $CDCl_3$), δ (ppm) = 173.7, 172.4, 138.5, 138.3, 135.5, 135.2, 131.4, 131.3, 129.5, 129.4, 129.1 (6C), 129.1 (2C), 129.0 (2C), 128.5 (2C), 128.4 (2C), 127.4 (4C), 120.2, 119.9, 58.1, 58.0, 52.5, 51.2, 43.6 (2C), 43.3, 43.2 (2C), 42.3, 38.7, 38.6, 38.5, 38.4, 37.6 (2C), 34.3 (2C), 33.0, 32.5, 31.3, 31.0, 30.4, 30.1, 28.4 (2C), 28.3, 28.2. **HRMS (APCI)** calcd for $C_{28}H_{33}N_2O$ $[M+H]^+$: 413.2587, found 413.2591.

2-((Diphenylmethylene)amino)-7-hydroxy-4-methylnonanenitrile (3ad)

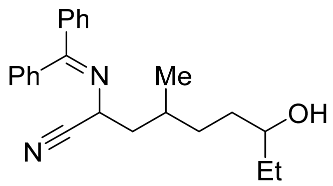

Prepared according to the *General Procedure* from acrylonitrile (11 mg, 0.20 mmol, 1.0 equiv) and bifunctional reagent **2l** (151 mg, 0.40 mmol, 2.0 equiv). The title compound was obtained as mixture of diastereomers (1:1:1:1), and after a chromatographic purification (5 – 20% AcOEt in heptane) the inseparable mixture **3ad** was obtained as a yellow oil (50 mg, 0.14 mmol, 72%). The combined data for both isomers is detailed down below. **^{1}H NMR** (300 MHz, $CDCl_3$), δ (ppm) = 7.68 – 7.57 (m, 2H), 7.56 – 7.39 (m, 4H), 7.41 – 7.28 (m, 2H), 7.25 – 7.12 (m, 2H), 4.43 – 4.16 (m, 1H), 3.49 – 3.38 (m, 1H), 2.13 – 1.91 (m, 1H), 1.81 – 1.56 (m, 2H), 1.49 – 1.32 (m, 5H), 1.29 – 1.19 (m, 1H), 1.17 – 1.05 (m, 1H), 0.98 – 0.69 (m, 6H). **^{13}C{^{1}H} NMR** (126 MHz, $CDCl_3$), δ (ppm) = 173.0 (2C), 172.7 (2C), 138.6, 138.5 (3C), 135.4 (2C), 135.3 (2C), 131.3 (2C), 131.2 (2C), 129.5 (4C), 129.1 (16C), 128.3 (8C), 127.6 (4C), 127.5 (4C), 120.1 (2C), 120.0 (2C), 73.4 (3C), 73.3, 51.9, 51.8, 51.3, 51.2, 42.0, 41.9, 41.8 (2C), 34.0 (2C), 33.9 (2C), 32.6, 32.5 (3C), 30.3 (2C), 30.2 (2C), 30.0, 29.9, 29.6 (2C), 19.6, 19.4, 19.3, 19.2, 10.0 (3C), 9.9. **HRMS (APCI)** calcd for $C_{23}H_{29}N_2O$ $[M+H]^+$: 349.2274, found 349.2269.

2-((Diphenylmethylene)amino)-7-hydroxy-4,6-dimethylheptanenitrile (3ae)

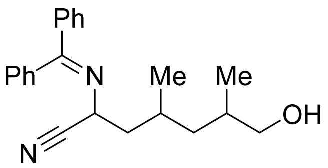

Prepared according to the *General Procedure* from acrylonitrile (11 mg, 0.20 mmol, 1.0 equiv) and bifunctional reagent **2m** (145 mg, 0.40 mmol, 2.0 equiv). The title compound was obtained as mixture of diastereomers (1:1:1:1), and after a chromatographic purification (5 – 20% AcOEt in heptane) **3ae** was obtained as an inseparable mixture of diastereomers (1:1) with the appearance of a yellow oil (17 mg, 0.05 mmol, 26%) and **3ae'** was obtained as an inseparable mixture of diastereomers (1:1) with the appearance of a yellow oil (28 mg, 0.08 mmol, 42%).

Data of **3ae.** ^{1}**H NMR** (300 MHz, $CDCl_3$), δ (ppm) = 7.67 – 7.55 (m, 2H), 7.54 – 7.31 (m, 6H), 7.25 – 7.14 (m, 2H), 4.39 – 4.12 (m, 1H), 3.51 – 3.32 (m, 2H), 2.19 – 1.97 (m, 1H), 1.83 – 1.60 (m, 3H), 1.48 – 1.16 (m, 3H), 0.93 – 0.72 (m, 6H). ^{13}**C{**^{1}**H} NMR** (126 MHz, $CDCl_3$), δ (ppm) = 173.2, 172.7, 138.6 (2C), 135.4, 135.3, 131.3 (2C), 129.6, 129.5, 129.1 (8C), 128.4 (4C), 127.6 (2C), 127.5 (2C), 120.2, 120.0, 68.2, 68.1, 52.0, 51.1, 41.7, 41.6, 40.8 (2C), 33.1, 33.0, 27.5, 27.0, 20.4, 20.1, 17.2, 17.1. **HRMS (APCI)** calcd for $C_{22}H_{27}N_2O$ $[M+H]^+$: 335.2118, found 335.2113.

Data of **3ae'.** ^{1}**H NMR** (300 MHz, $CDCl_3$), δ (ppm) = 7.67 – 7.55 (m, 2H), 7.54 – 7.30 (m, 6H), 7.25 – 7.14 (m, 2H), 4.41 – 4.13 (m, 1H), 3.47 – 3.33 (m, 2H), 2.11 – 1.88 (m, 1H), 1.81 – 1.59 (m, 3H), 1.50 – 1.38 (m, 1H), 1.22 – 1.10 (m, 1H), 1.05 – 0.91 (m, 2H), 0.95 – 0.68 (m, 6H). ^{13}**C{**^{1}**H} NMR** (126 MHz, $CDCl_3$), δ (ppm) = 173.2, 172.8, 138.6 (2C), 135.4, 135.3, 131.3 (2C), 129.6, 129.5, 129.1 (6C), 128.4 (4C), 127.6 (2C), 127.5 (2C), 120.1, 120.0, 68.7 (2C), 51.7, 51.2, 42.8, 42.7, 40.4, 40.2, 33.2, 33.0, 27.2, 27.0, 19.2, 19.1, 16.4 (2C). **HRMS (APCI)** calcd for $C_{22}H_{27}N_2O$ $[M+H]^+$: 335.2118, found 335.2112.

2-((Diphenylmethylene)amino)-4-(4-hydroxybutan-2-yl)-7-methyloctanenitrile (3af)

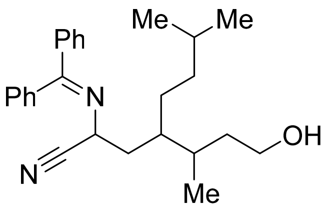

Prepared according to the *General Procedure* from acrylonitrile (11 mg, 0.20 mmol, 1.0 equiv) and bifunctional reagent **2n** (168 mg, 0.40 mmol, 2.0 equiv). The title compound was obtained as mixture of diastereomers (1:1:1:1), and after a chromatographic purification (5 – 10% AcOEt in heptane) the inseparable mixture **3af** was obtained as

a yellow oil (48 mg, 0.12 mmol, 61%). The combined data for both isomers is detailed down below. **^{1}H NMR** (300 MHz, $CDCl_3$), δ (ppm) = 7.69 – 7.59 (m, 2H), 7.59 – 7.38 (m, 4H), 7.40 – 7.29 (m, 2H), 7.27 – 7.14 (m, 2H), 4.37 – 4.16 (m, 1H), 3.75 – 3.46 (m, 2H), 2.11 – 1.85 (m, 1H), 1.74 – 0.96 (m, 11H), 0.88 – 0.68 (m, 9H). **^{13}C{^{1}H} NMR** (126 MHz, $CDCl_3$), δ (ppm) = 173.1, 172.8 (2C), 172.6, 138.5 (4C), 135.5, 135.4, 135.3 (2C), 131.3 (4C), 129.5 (4C), 129.2 (2C), 129.1 (14C), 128.4 (8C), 127.6 (2C), 127.5 (6C), 120.2 (2C), 120.1 (2C), 61.4, 61.3 (3C), 52.9, 52.6, 52.1, 51.6, 39.7, 39.6, 39.4, 39.1, 36.9 (3C), 36.8, 36.8 (2C), 36.7, 36.5, 36.4, 36.3, 36.1, 35.6, 31.2, 30.8, 30.7, 30.3, 29.1, 28.5 (3C), 28.4 (2C), 27.8, 27.3, 22.8 (3C), 22.7 (2C), 22.6 (2C), 22.5, 15.8, 15.6, 15.3, 15.1. **HRMS (APCI)** calcd for $C_{26}H_{34}N_2O$ $[M+H]^+$: 391.2744, found 391.2739.

2-((Diphenylmethylene)amino)-4-(2-hydroxyethoxy)butanenitrile (3ag)

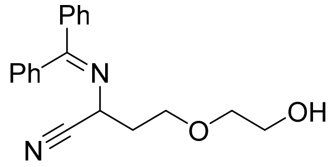

Prepared according to the *General Procedure* from acrylonitrile (11 mg, 0.20 mmol, 1.0 equiv) and bifunctional reagent **2o** (135 mg, 0.40 mmol, 2.0 equiv). After a chromatographic purification (10 – 30% AcOEt in heptane), the title compound **3ag** was obtained as a yellow oil (41 mg, 0.13 mmol, 68%). **^{1}H NMR** (300 MHz, $CDCl_3$), δ (ppm) = 7.69 – 7.61 (m, 2H), 7.54 – 7.42 (m, 4H), 7.39 – 7.33 (m, 2H), 7.25 – 7.17 (m, 2H), 4.45 (dd, J = 7.1, 6.4 Hz, 1H), 3.71 – 3.51 (m, 4H), 3.51 – 3.39 (m, 2H), 2.30 – 2.12 (m, 2H), 1.90 (t, J = 5.9 Hz, 1H). **^{13}C{^{1}H} NMR** (75 MHz, $CDCl_3$), δ (ppm) = 173.8, 138.5, 135.3, 131.4, 129.6, 129.1 (4C), 128.4 (2C), 127.5 (2C), 119.8, 72.1, 66.6, 61.8, 50.1, 35.0. **HRMS (APCI)** calcd for $C_{19}H_{21}N_2O_2$ $[M+H]^+$: 309.1598, found 309.1601.

2-((Diphenylmethylene)amino)-4-((2-hydroxyethyl)thio)butanenitrile (3ah)

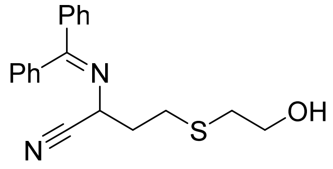

Prepared according to the *General Procedure* from acrylonitrile (11 mg, 0.20 mmol, 1.0 equiv) and bifunctional reagent **2p** (141 mg, 0.40 mmol, 2.0 equiv). After a chromatographic purification (10 – 35% AcOEt in heptane), the title compound **3ah** was obtained as a yellow oil (32 mg, 0.10 mmol, 49%). **^{1}H NMR** (300 MHz, $CDCl_3$), δ (ppm) = 7.69 – 7.59 (m, 2H), 7.57 – 7.42 (m, 4H), 7.40 – 7.32 (m, 2H), 7.25 – 7.18 (m, 2H), 4.42 (t, J = 6.6 Hz, 1H), 3.70 (t, J = 6.0 Hz, 2H), 2.74 – 2.60 (m, 4H), 2.24 – 2.15 (m, 2H), 2.08 (bs, 1H). **^{13}C{^{1}H} NMR** (126 MHz, $CDCl_3$), δ (ppm) = 174.1, 138.4, 135.2, 131.5, 129.6, 129.2 (2C), 129.1 (2C), 128.4 (2C), 127.5 (2C), 119.2, 60.6, 51.7, 35.3, 34.8, 27.6. **HRMS (APCI)** calcd for $C_{19}H_{21}N_2OS$ $[M+H]^+$: 325.1369, found 325.1361.

***tert*-Butyl (1-cyano-1-((diphenylmethylene)amino)-6-hydroxyhexan-3-yl)carbamate (3ai)**

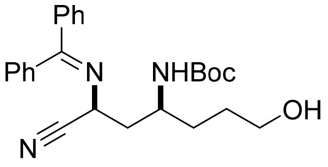

***syn*-3ai**

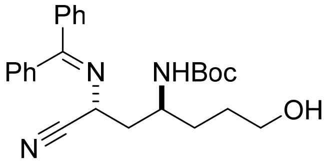

***anti*-3ai'**

Prepared according to the *General Procedure* from acrylonitrile (11 mg, 0.20 mmol, 1.0 equiv) and bifunctional reagent **2q** (180 mg, 0.40 mmol, 2.0 equiv). The title compound was obtained as mixture of diastereomers (1:1), and after a chromatographic purification (10 – 40% AcOEt in heptane) ***syn*-3ai** was obtained as a white solid (27 mg, 0.06 mmol, 32%) and ***anti*-3ai'** was obtained as a yellow oil (43 mg, 0.10 mmol, 51%).

Data of ***syn*-3ai. mp** = 149 – 151 ºC. **^{1}H NMR** (300 MHz, $CDCl_3$), δ (ppm) = 7.73 – 7.60 (m, 2H), 7.57 – 7.40 (m, 4H), 7.39 – 7.31 (m, 2H), 7.25 – 7.16 (m, 2H), 4.53 (d, J = 8.7 Hz, 1H), 4.35 (t, J = 6.9 Hz, 1H), 3.81 – 3.50 (m, 3H), 2.28 – 2.13 and 2.12 – 1.94 (AB system, Δν = 52.6 Hz, 2H), 1.81 (s, 1H), 1.65 – 1.49 (m, 4H), 1.27 (s, 9H).**^{13}C{^{1}H} NMR** (126 MHz, $CDCl_3$), δ (ppm) = 173.5, 155.5, 138.4, 135.3, 131.4, 129.5, 129.2 (2C), 129.2 (2C), 128.4 (3C), 127.5, 119.5, 79.4, 62.5, 51.4, 48.8, 40.2, 32.3, 28.7, 28.3 (3C). **HRMS (APCI)** calcd for $C_{25}H_{32}N_3O_3$ $[M+H]^+$: 422.2438, found 422.2436.

Data of ***anti*-3ai'**. **^{1}H NMR** (500 MHz, $CDCl_3$), δ (ppm) = 7.69 – 7.55 (m, 2H), 7.54 – 7.39 (m, 4H), 7.38 – 7.30 (m, 2H), 7.29 – 7.23 (m, 2H), 4.50 (d, *J* = 9.4 Hz, 1H), 4.28 (dd, *J* = 9.5, 3.9 Hz, 1H), 3.73 – 3.51 (m, 3H), 2.42 – 2.27 (m, 1H), 1.95 – 1.90 (m, 2H), 1.62 – 1.51 (m, 4H), 1.40 (s, 9H).**^{13}C{^{1}H} NMR** (126 MHz, $CDCl_3$), δ (ppm) = 175.0, 155.7, 138.6, 135.5, 131.4, 129.4, 129.1 (2C), 129.1 (2C), 128.4 (2C), 127.6 (2C), 119.6, 79.5, 62.4, 49.5, 47.5, 40.3, 32.6, 28.7, 28.5 (3C). **HRMS (APCI)** calcd for $C_{25}H_{32}N_3O_3$ $[M+H]^+$: 422.2438, found 422.2429.

4-((*tert*-Butyldimethylsilyl)oxy)-2-((diphenylmethylene)amino)-7-hydroxyheptanenitrile (3aj)

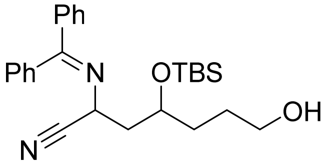

Prepared according to the *General Procedure* from acrylonitrile (11 mg, 0.20 mmol, 1.0 equiv) and bifunctional reagent **2r** (186 mg, 0.40 mmol, 2.0 equiv). The title compound was obtained as mixture of diastereomers (1:1), and after a chromatographic purification (5 – 20% AcOEt in heptane) the inseparable mixture **3aj** was obtained as a yellow oil (77 mg, 0.18 mmol, 89%). The combined data for both isomers is detailed down below. **^{1}H NMR** (300

MHz, $CDCl_3$), δ (ppm) = 7.68 – 7.58 (m, 2H), 7.55 – 7.40 (m, 4H), 7.39 – 7.31 (m, 2H), 7.27 – 7.18 (m, 2H), 4.44 (dd, *J* = 9.9, 5.6 Hz, 0.5H), 4.28 (dd, *J* = 8.0, 6.3 Hz, 0.5H), 4.07 – 3.95 (m, 0.5H), 3.95 – 3.83 (m, 0.5H), 3.66 – 3.50 (m, 2H), 2.33 – 2.16 (m, 1H), 2.06 – 1.80 (m, 2H), 1.63 – 1.46 (m, 4H), 0.81 (s, 4.5H), 0.70 (s, 4.5H), 0.03 (s, 2H), 0.02 (s, 2H), -0.02 (s, 2H), -0.11 (s, 2H). **$^{13}C\{^1H\}$ NMR** (126 MHz, $CDCl_3$), δ (ppm) = 173.2 (2C), 138.6 (2C), 135.4, 135.1, 131.3 (2C), 129.6, 129.5, 129.2 (2C), 129.1 (6C), 128.3 (4C), 127.7 (2C), 127.4 (2C), 120.0, 119.7, 69.0, 68.8, 62.9, 62.8, 51.5, 49.8, 41.6 (2C), 34.0, 33.1, 27.8, 27.7, 25.9 (3C), 25.8 (3C), 18.1, 17.9, -4.3 (2C) -4.5, -5.0. **HRMS (ESI)** calcd for $C_{26}H_{37}N_2O_2Si$ $[M+H]^+$: 437.2619, found 437.2616.

3. Derivatization Reactions

Steglich esterification & hydrolysis

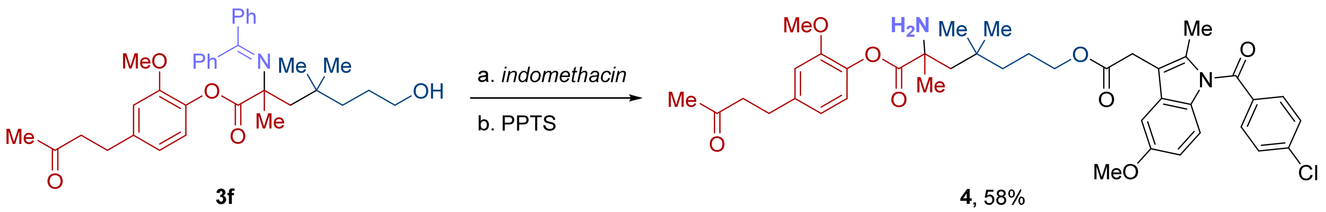

Amine **4** was synthesized following a modified reported procedure.[5] To a stirred solution of **3f** (81 mg, 0.15 mmol, 1.0 equiv), indomethacin (80 mg, 0.22 mmol, 1.5 equiv), and 4-dimethylaminopyridine (2 mg, 0.01 mmol, 10 mol%) in CH_2Cl_2 (0.7 mL), *N*-(3-dimethylaminopropyl)-*N'*-ethylcarbodiimidehydrochloride (71 mg, 0.37 mmol, 2.5 equiv) was added at room temperature. The reaction was monitored by TLC analysis, and upon completion (16 h), the mixture was poured into a separatory funnel and washed with brine (2 x 20 mL). The organic layer was dried ($MgSO_4$), and the volatiles were removed under reduced pressure. In a round-bottom flask, the crude mixture was dissolved in THF (2.8 mL) and H_2O (0.2 mL), and pyridinium *p*-toluene sulfonate (43 mg, 0.18 mmol, 1.2equiv) was added. The reaction was stirred at room temperature for 18 h and then diluted with EtOAc and H_2O. The mixture was poured into a separatory funnel, and the aqueous layer was extracted with EtOAc (10 mL x 2). The combined organic layers were dried ($MgSO_4$), and the volatiles were removed under reduced pressure. The crude mixture was subjected to purification by flash column chromatography (0 – 15% *i*-PrOH in CH_2Cl_2) to obtain **5** as yellow oil (62 mg, 0.09 mmol, 58%).

^1H NMR (300 MHz, $CDCl_3$) δ (ppm) = 7.67 – 7.59 (m, 2H), 7.48 – 7.41 (m, 2H), 6.96 (d, *J* = 2.5 Hz, 1H), 6.87 (dd, *J* = 9.9, 8.6 Hz, 2H), 6.78 (d, *J* = 1.9 Hz, 1H), 6.72 (dd, *J* = 8.1, 1.9 Hz, 1H), 6.65 (dd, *J* = 9.0, 2.5 Hz, 1H), 4.08 (t, *J* = 6.6 Hz, 2H), 3.81 (s, 3H), 3.77 (s, 3H), 3.64 (s, 2H), 2.91 – 2.82 (m, 2H), 2.80 – 2.68 (m, 2H), 2.37 (s, 3H), 2.13 (s, 3H), 2.03 (d, *J* = 14.6 Hz, 1H), 2.16 – 1.91 (bs, 2H), 1.68 (d, *J* = 14.6 Hz, 1H), 1.65 – 1.57 (m, 2H), 1.49 (s, 3H), 1.33 – 1.23 (m, 2H), 0.99 (d, *J* = 11.4 Hz, 6H). **$^{13}C\{^1H\}$ NMR** (76 MHz, $CDCl_3$) δ (ppm) = 207.7, 171.0 (2C), 168.4, 156.1, 151.0, 140.2, 139.3, 138.3, 136.0, 134.1, 131.3 (2C), 130.9, 130.8, 129.2 (2C), 122.2, 120.4, 115.0, 112.8, 112.8, 111.7, 101.6, 65.8, 58.5, 55.8, 55.8, 50.3, 45.2, 40.7, 33.6, 30.5, 30.4, 30.2, 29.7, 28.1, 27.4, 23.6, 13.5. **HRMS (APCI)** calcd for $C_{40}H_{48}ClN_2O_8$ $[M+H]^+$: 719.3094, found 719.3081.

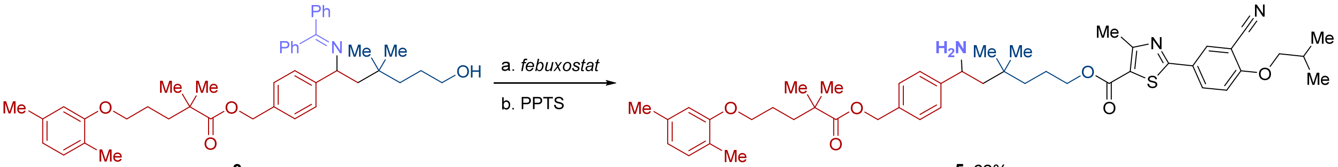

3s → **5**, 62%

Amine **5** was synthesized following a modified reported procedure.[6] To a stirred solution of **3s** (95 mg, 0.15 mmol, 1.0 equiv), febuxostat (69 mg, 0.23 mmol, 1.5 equiv), and 4-dimethylaminopyridine (2 mg, 0.01 mmol, 10 mol%) in CH_2Cl_2 (0.8 mL), *N*-(3-dimethylaminopropyl)-*N*′-ethylcarbodiimide hydrochloride (70 mg, 0.37 mmol, 2.5 equiv) was added at room temperature. The reaction was monitored by TLC analysis, and upon completion (16 h), the mixture was poured into a separatory funnel and washed with brine (2 x 20 mL). The organic layer was dried ($MgSO_4$), and the volatiles were removed under reduced pressure. In a round-bottom flask, the crude mixture was dissolved in THF (2.7 mL) and H_2O (0.2 mL), and pyridinium *p*-toluene sulfonate (44 mg, 0.18 mmol, 1.2equiv) was added. The reaction was stirred at room temperature for 18 h, and then diluted with EtOAc and H_2O. The mixture was poured into a separatory funnel, and the aqueous layer was extracted with EtOAc (10 mL x 2). The combined organic layers were dried ($MgSO_4$), and the volatiles were removed under reduced pressure. The crude mixture was subjected to purification by flash column chromatography (0 – 15% *i*-PrOH in CH_2Cl_2) to obtain **6** as yellow oil (71 mg, 0.09 mmol, 62%).

^{1}H NMR (300 MHz, $CDCl_3$), δ (ppm) = 8.14 (d, *J* = 2.3 Hz, 1H), 8.06 (dd, *J* = 8.8, 2.3 Hz, 1H), 7.37 – 7.31 (m, 2H), 7.30 – 7.25 (m, 2H), 6.98 (dd, *J* = 8.2, 5.3 Hz, 2H), 6.63 (d, *J* = 7.5 Hz, 1H), 6.57 (s, 1H), 5.06 (s, 2H), 4.14 (t, *J* = 6.6 Hz, 2H), 4.23 – 4.02 (m, 1H), 3.91 – 3.84 (m, 4H), 2.74 (s, 3H), 2.28 (s, 3H), 2.25 – 2.16 (m, 1H), 2.14 (s, 3H), 1.89 – 1.54 (m, 8H), 1.36 – 1.24 (m, 2H), 1.22 (s, 6H), 1.09 (d, *J* = 6.7 Hz, 6H), 1.09 – 1.05 (m, 1H), 0.86 (d, *J* = 9.2 Hz, 6H). **^{13}C{^{1}H} NMR** (76 MHz, $CDCl_3$), δ (ppm) = 177.6, 167.3, 162.6, 162.1, 161.2, 157.0, 136.5, 132.7, 132.2, 130.4 (2C), 128.3 (2C), 127.0, 126.1, 123.6, 121.9, 120.8 (2C), 115.5, 112.7, 112.0 (2C), 103.1, 75.8, 68.0, 66.1, 65.9, 42.2, 38.8, 37.2, 33.0, 28.3, 27.8, 27.7, 25.2 (3C), 23.6, 21.5, 19.2 (3C), 17.6, 15.9. **HRMS (APCI)** calcd for $C_{46}H_{60}N_3O_6S$ $[M+H]^+$: 782.4197, found 782.4190.

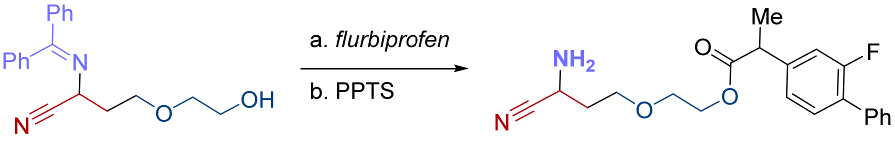

3ag → **6**, 71%

Amine **6** was synthesized following a modified reported procedure.[5] To a stirred solution of **3ag** (46 mg, 0.05 mmol, 1.0 equiv), flurbiprofen (49 mg, 0.20 mmol, 1.5 equiv), and 4-dimethylaminopyridine (2 mg, 0.01 mmol, 10 mol%) in CH_2Cl_2 (0.7 mL), *N*-(3-dimethylaminopropyl)-*N*′-ethylcarbodiimide hydrochloride (64 mg, 0.33 mmol, 2.5 equiv) was added at room temperature. The reaction was monitored by TLC analysis, and upon completion (16 h), the mixture was poured into a separatory funnel and washed with brine (2 x 20 mL). The organic

layer was dried ($MgSO_4$), and the volatiles were removed under reduced pressure. In a round-bottom flask, the crude mixture was dissolved in THF (2.5 mL) and H_2O (0.18 mL), and pyridinium *p*-toluene sulfonate (40 mg, 0.16 mmol, 1.2equiv) was added. The reaction was stirred at room temperature for 18 h, and then diluted with EtOAc and H_2O. The mixture was poured into a separatory funnel, and the aqueous layer was extracted with EtOAc (10 mL x 2). The combined organic layers were dried ($MgSO_4$), and the volatiles were removed under reduced pressure. The crude mixture was subjected to purification by flash column chromatography (50 – 90% AcOEt in heptane) to obtain **6** as yellow oil (35 mg, 0.09 mmol, 71%) as an inseparable mixture of diastereomers (1:1). The combined data for both isomers is detailed below.

^{1}H NMR (300 MHz, $CDCl_3$), δ (ppm) = 7.59 – 7.48 (m, 2H), 7.48 – 7.32 (m, 4H), 7.20 – 7.10 (m, 2H), 4.32 – 4.19 (m, 2H), 3.91 – 3.75 (m, 2H), 3.72 – 3.54 (m, 4H), 2.08 – 1.87 (m, 4H), 1.55 (d, J = 7.2 Hz, 3H). **^{13}C {^{1}H}NMR** (76 MHz, $CDCl_3$), δ (ppm) = 174.0 (2C), 159.8 (d, $J_{C\text{-}F}$ = 248.3 Hz, 2C), 141.9 (d, $J_{C\text{-}F}$ = 7.6 Hz, 2C), 135.5 (2C), 131.0, 130.9, 129.1 (2C), 129.0 (2C), 128.6 (4C), 128.0 (2C), 127.8 (2C), 123.8, 123.7, 121.8 (2C), 115.4 (d, $J_{C\text{-}F}$ = 23.7 Hz, 2C), 69.1 (2C), 67.1, 67.1, 64.0, 63.9, 45.1 (2C), 41.1 (2C), 35.0 (2C), 18.5 (2C). **^{19}F{^{1}H} NMR** (282 MHz, $CDCl_3$) δ (ppm) = -117.5 (s, 1F), -117.6 (s, 1F). **HRMS (APCI)** calcd for $C_{21}H_{24}FN_2O_3$ $[M+H]^+$: 371.1765, found 371.1759.

Oxidation & hydrolisis

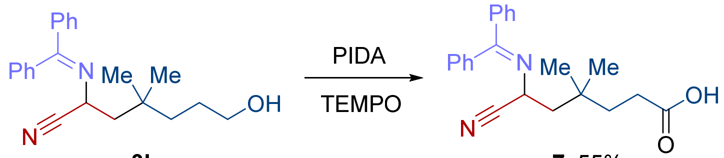

3b **7**, 55%

Amine **8** was synthesized following a modified reported procedure.[7] To a stirred solution of **3b** (50 mg, 0.15 mmol, 1.0 equiv) in CH_2Cl_2: H_2O (2:1, 4.0 mL), PIDA (116 mg, 0.36 mmol, 2.4 equiv) and TEMPO (5 mg, 0.03 mmol, 1.0 equiv) were added, and the mixture was stirred overnight at room temperature. The reaction mixture was quenched with $Na_2S_2O_3$ and extracted with CH_2Cl_2 (10 mL x 3) in a separatory funnel. The combined organic layers were dried ($MgSO_4$), and the volatiles were removed under reduced pressure. The crude mixture was subjected to purification by flash column chromatography (50 – 100% AcOEt in heptane) to obtain **7** as yellow oil (28 mg, 0.08 mmol, 55%).

^{1}H NMR (300 MHz, $CDCl_3$), δ (ppm) = 7.70 – 7.60 (m, 2H), 7.57 – 7.40 (m, 4H), 7.39 – 7.31 (m, 2H), 7.27 – 7.23 (m, 2H), 4.29 (t, J = 6.5 Hz, 1H), 2.37 – 2.21 (m, 2H), 2.04 and 1.90 (AB system, Δν = 42.4 Hz, J = 14.3, 6.5 Hz, 2H), 1.56 – 1.45 (m, 2H), 0.87 (s, 3H), 0.82 (s, 3H). The proton from the acid was not observed. **^{13}C{^{1}H} NMR** (76 MHz, $CDCl_3$), δ (ppm) = 179.6, 172.7, 138.5, 135.4, 131.3, 129.6, 129.2 (2C), 129.1 (2C), 128.4 (2C), 127.4

[7] Epp, J. B.; Widlanski, T. S. *J. Org. Chem.* **1999**, *64*, 293-295.

(2C), 120.5, 50.0, 45.9, 36.5, 32.8, 29.2, 27.1, 26.9. **HRMS (APCI)** calcd for $C_{22}H_{24}N_2O_2$ $[M]^+$: 348.1576, found 348.1573.

4. Large Scale Synthesis of 3b

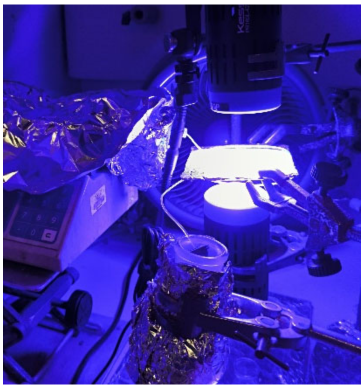

Figure S2. Gram scale synthesis reaction setup of **3b**.

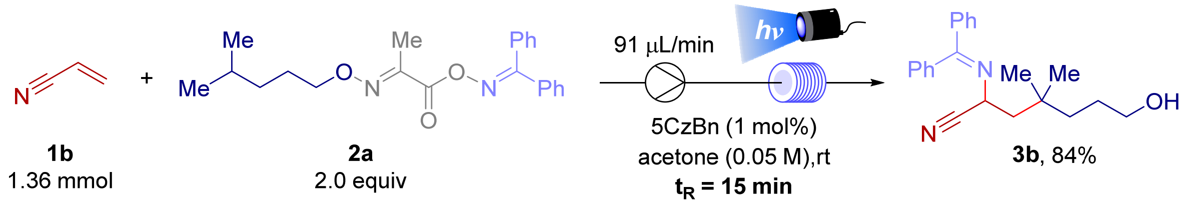

To a 100mL round bottom flask equipped with a magnetic stir bar was added the bifunctional reagent **2a** (1.0 g, 2.73 mmol, 2.0 equiv) and 5BzCN (13 mg, 13.6 μmol, 1 mol%). The flask was sealed with a rubber septum, evacuated, and back-filled with nitrogen. After this process was repeated 3 times, anhyd degassed acetone (21.0 mL, c = 0.06 M), and alkene **1b** (72 mg, 1.36 mmol, 1.0 equiv) were added via syringe. The reaction mixture was transferred to a 25 mL syringe and connected to our homemade flow system. The mixture was pumped through a PFA HPLC tube (total volume of the system: 1.36 mL), and irradiated with two Kessil PR160-blue LED lamp (30 W High Luminous DEX 2100 LED, λ_{max} = 427 nm) for 4 h 24 min (residence time: 15 min, flow rate: 91 μL/min) as described in Figure S2. The lamps were placed 2 cm away from the reaction system, and cooled at room temperature by an external fan. Upon completion, the volatiles were removed under reduced pressure, and the crude mixture was subjected to purification by flash column chromatography (5 – 30% AcOEt in heptane). The title compound **3b** was obtained as a yellow oil (381 mg, 1.14 mmol, 84%).

5. Mechanistic Investigations

6.1. UV/vis studies

UV/vis absorption spectra were measured in a 1 cm quartz cuvette using JASCO V-660 UV/vis spectrophotometer. Absorption spectra of **2b** (0.05 M) and 5CzBN (0.1 mM) were recorded in acetone.

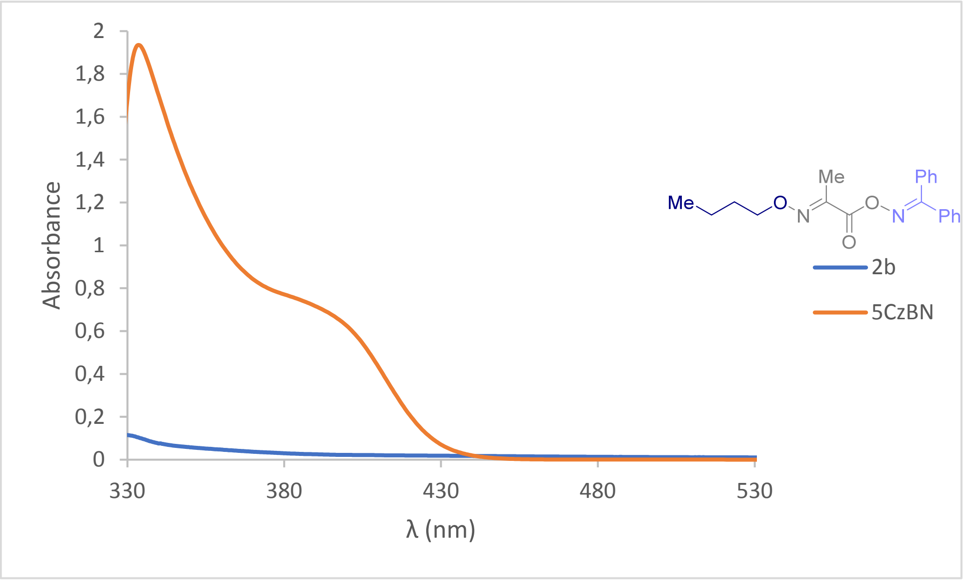

Figure S3. UV/vis absorption spectra of **2b** and 5CzBN.

6.2. Quantum yield

The quantum yield of the reaction was determined using the procedure reported previously.[9-10] Alkenes **1f** and **1b**, and bifunctional reagents **2a** and **2b** were used, respectively, as model substrates to calculated the quantum yield, using trimethoxybenzene as internal standard in a proportion 1:1 with the alkenes.

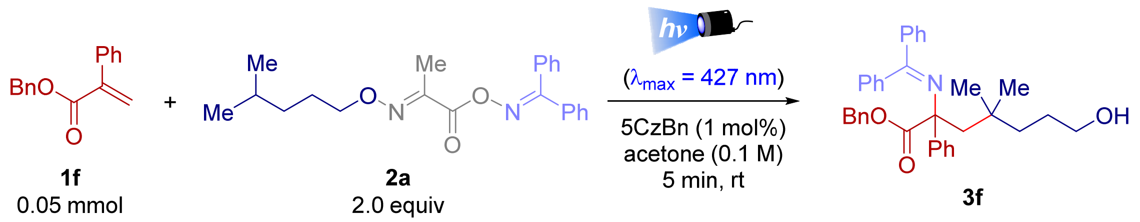

Reaction 1. Reaction performed under standard conditions (*General Procedure*) using alkene**1f** (0.05 mmol) and bifunctional reagent **2a** (2.0 equiv) to formed the final product **3f**, and stopped after 5 min of irradiation.

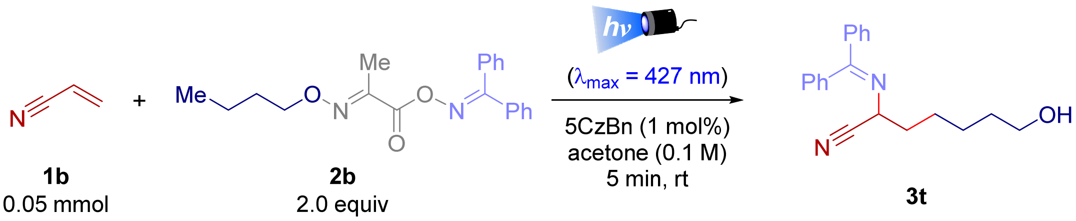

Reaction 2. Reaction performed under standard conditions (*General Procedure*) using alkene**1b** (0.05 mmol) and bifunctional reagent **2b** (2.0 equiv) to formed the final product **3t**, and stopped after 5 min of irradiation.

The quantum yield of the reaction is defined as:

$$\Phi = \frac{\text{mol of product formed}}{\text{mol of photon flux} \times \text{t} \times \text{f}} \qquad (1)$$

where Φ is the quantum yield of the reaction, t is the time of the reaction (s), f is the incident light absorbed by the organophotocatalyst at 427 nm and the photon flux is calculated by standard ferrioxalate actinometry[11] (section C).

A) *Incident light absorbed by 5CzBn (f)*

The fraction of light, f, absorbed was determined according to *equation 2*:

$$f = 1 - 10^{-A} \qquad (2)$$

where A is the absorbance of the fully soluble 5CzBn in acetone at 427 nm. The absorbance of the organophotocatalyst was measured by adding a solution of 5CzBn (0.9 mg, 0.001 mmol) in acetone (1 mL) to a quartz cuvette equipped with a Teflon cap. The absorbance of the solution was recorded, and the absorbance value at 427 nm was determined to be 2.16507 (Figure S4), indicating the fraction of light absorbed is ~1 according to equation 2.

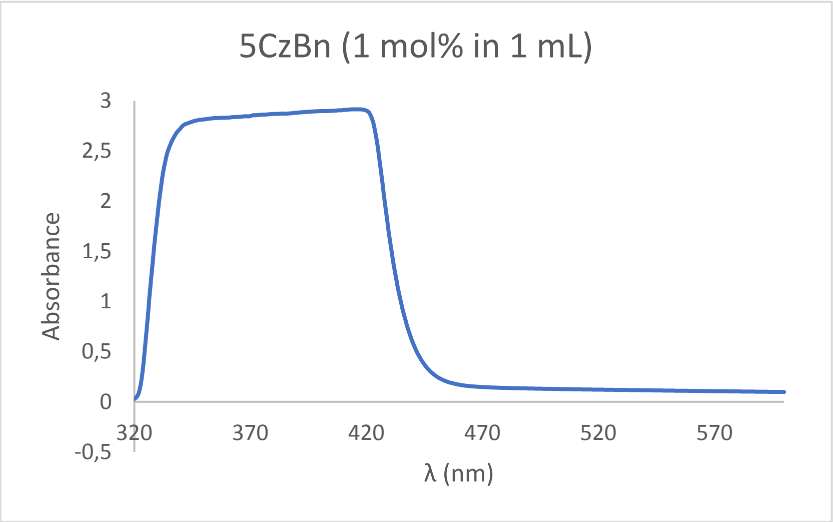

Figure S4. Absorption spectrum of 5CzBn in acetone.

B) The photoredox reaction

The photoinduced transformations were performed as described in *General Procedure*. Afterwards, 1,3,5-trimethoxybenzene was added as internal standard, and the volatiles were removed under vacuum. The yields of the reactions were determined by ^{1}H NMR, where 15 μmols (30%) of **3f** and 15 μmols (30%) of **3t** were respectively obtained after 300 s.

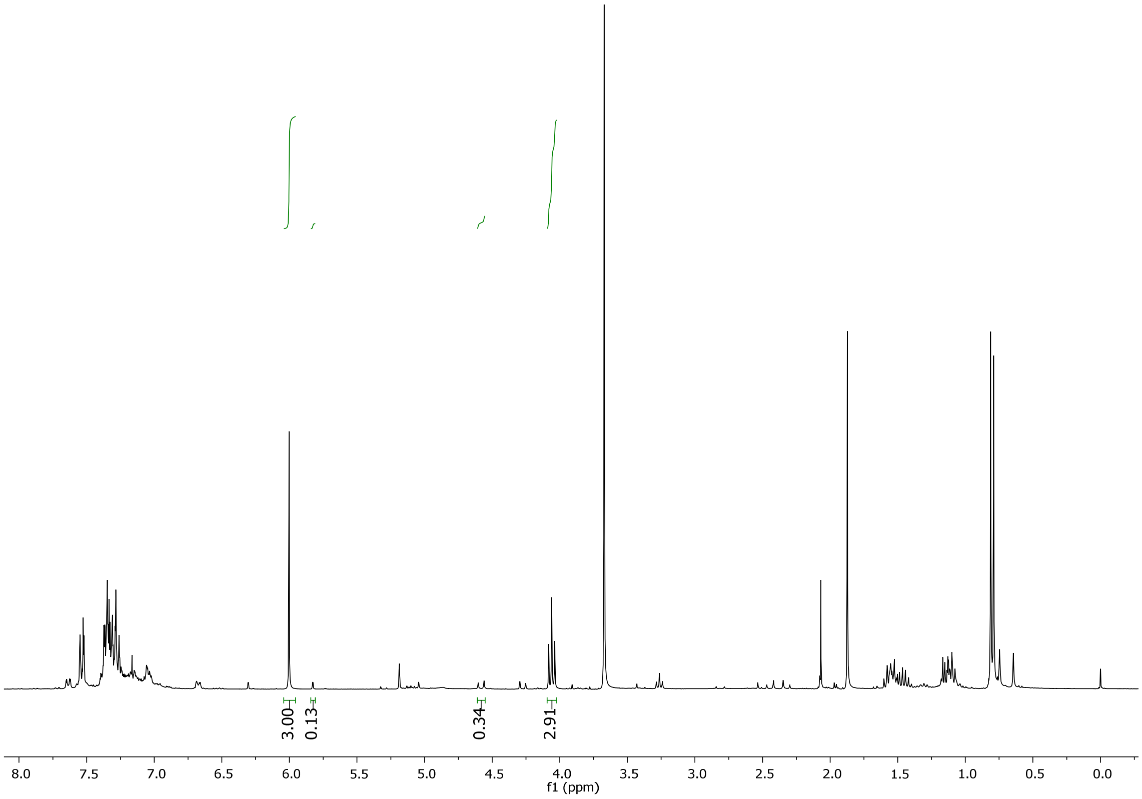

Figure S5. Crude mixture of reaction 1 with 1,3,5-trimethoxybenzene after 5 min of irradiation at 427 nm.

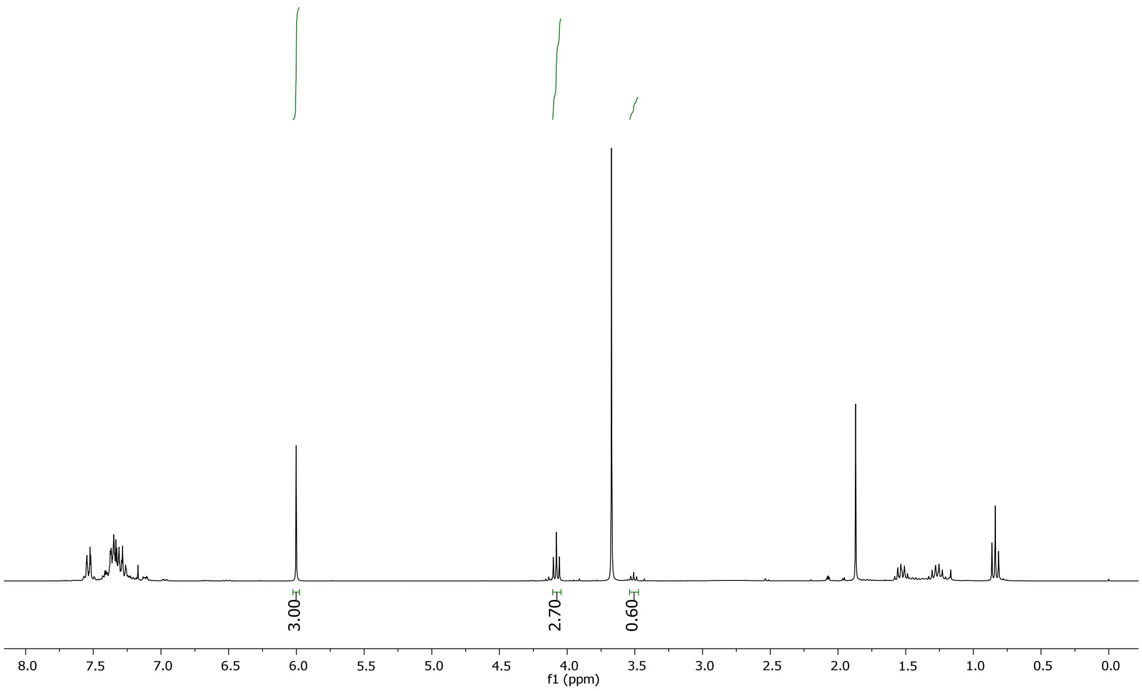

Figure S6. Crude mixture of reaction 2 with 1,3,5-trimethoxybenzene after 5 min of irradiation at 427 nm.

C) Photon flux at 406 nm.

Standard ferrioxalate actinometry was used to determine the photon flux of the spectrophotometer using equations 3 and 4.[9-11] For the ferrioxalate actinometer the production of iron(II) ions proceeds by the following reactions:[11]

$$[Fe(C_2O_4)_n]^{(3-2n)+} \xrightarrow{h\nu} Fe^{2+} + (n\text{-}1)(C_2O_4)^{2-} + C_2O_4^{-}$$

$$[Fe(C_2O_4)_n]^{(3-2n)+} + C_2O_4^{-} \longrightarrow Fe^{2+} + n(C_2O_4)^{2-} + 2CO_2$$

The moles of Fe^{2+} formed are determined spectrophotometrically by development with 1,10-phenanthroline (phen) to form the red $[Fe(phen)_3]^{2+}$ moiety (λ = 510 nm).[9-11] The photon flux is defined as:

$$\text{Photon flux} = \frac{\text{mol }(Fe^{2+})}{\Phi(Fe^{2+}) \times \text{t} \times \text{f}} \qquad (3)$$

Where Φ is the quantum yield for the ferrioxalate actinometer (1.188 at λ = 406 nm),[11] t is the time (s), and f > 0.999, and the mol of Fe^{2+} are calculated according to *equation 4*.

$$\text{mol }(Fe^{2+}) = \frac{\text{V} \times \Delta\text{A}}{\text{l} \times \varepsilon} \qquad (4)$$

Where V is the total volume of the solution, ΔA is the difference in absorbance between irradiated and non-irradiated solutions, l is the path length (1.0 cm), ε is the molar absorptivity at 510 nm (11,110 L $mol^{-1}cm^{-1}$).[11]

D) ***Experimental.***

The following solutions were prepared in the dark (flasks were wrapped in aluminum foil) and stored in the dark at room temperature:

- *Ferrioxalate solution (0.15 M):* Potassium ferrioxalate hydrate (2.21 g) was added to a flask wrapped in aluminum foil containing H_2SO_4 (30 mL, 0.05 M). The flask was stirred for complete solvation of the green solid in complete darkness. It is noteworthy that the solution should not be exposed to any incident light.
- *Developer solution:* 1,10-Phenanthroline (50 mg) and sodium acetate (11.25 g) was added to a flask containing H_2SO_4 (50 mL, 0.5 M) and sonicated until completely solvated.

The absorbance of the non-irradiated sample. The buffered solution of phen (0.35 mL) was added to a ferrioxalate (2.0 mL) in a vial that had been covered with aluminum foil {lights of the laboratory were switched off}. The vial was capped and allowed to rest for 1 h and then transferred to a cuvette. The absorbance of the non-irradiated was measured at 515 nm to be 0.290 (Figure S7).

The absorbance of the irradiated sample. In a glass vial equipped with a stir bar was added the ferrioxalate solution (2.0 mL), and the stirred solution was irradiated for 75.0 s at λ = 427 nm. After irradiation, the buffered phen solution (0.35 mL) was added to the cuvette and allowed to rest for 1 h in the dark to allow the ferrous ions to coordinate completely to phen. The absorbance was measured at 515 nm to be 0.631 (Figure S7).

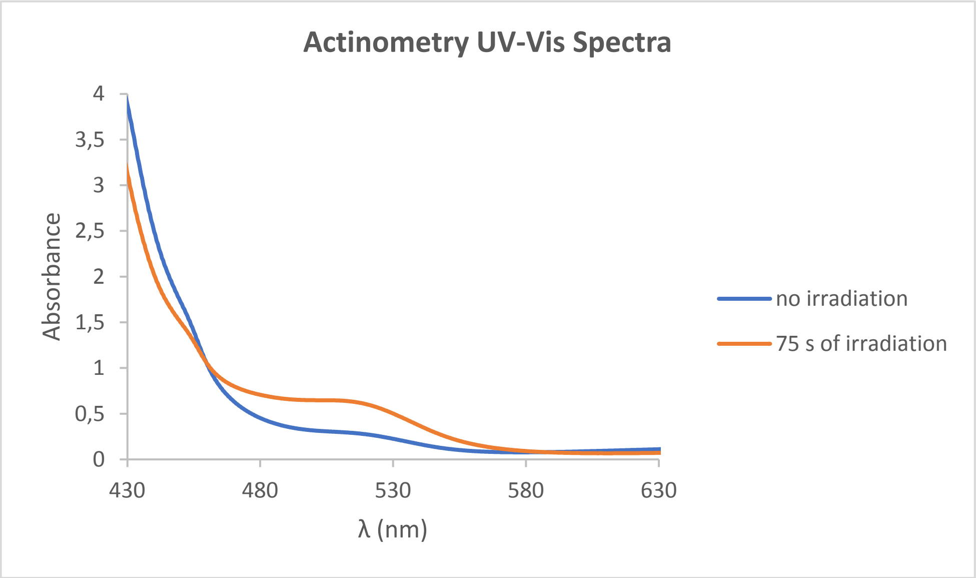

Figure S7. Absorption spectra for irradiated and non-irradiated samples of $[Fe(phen)_3]^{2+}$.

Photon flux sample calculation. Sample calculation:

$$\text{mol } (Fe^{2+}) = \frac{\text{V} \times \Delta\text{A}}{\text{l} \times \varepsilon} \qquad (4)$$

$$\text{mol } (Fe^{2+}) = \frac{0.00235 \text{ L} \times 0.342}{1.00\ cm \times 11.110 \text{ L}mol^{-1}cm^{-1}} = 7.227 \times 10^{-8}\ mol$$

$$\text{Photon flux} = \frac{\text{mol } (Fe^{2+})}{\Phi(Fe^{2+}) \times \text{t} \times \text{f}} \qquad (3)$$

$$\text{Photon flux} = \frac{7.227 \times 10^{-8}\ mol}{1.188 \times 75.0 \text{ s} \times 1.00} = 8.112 \times 10^{-10} \text{ einstein s}^{-1}$$

E) Quantum yield of the reactions.

Therefore, the quantum yield of the Reaction 1 is determined to be:

$$\Phi = \frac{\text{mol of product formed}}{\text{mol of photon flux} \times \text{t} \times \text{f}} \qquad (1)$$

$$\Phi = \frac{1.5 \times 10^{-5}\ mol}{8.112 \times 10^{-10} \text{ einstein s}^{-1} \times 300 \text{ s} \times 1} = \mathbf{69.9}$$

Therefore, the quantum yield of the Reaction 2 is determined to be:

$$\Phi = \frac{\text{mol of product formed}}{\text{mol of photon flux} \times \text{t} \times \text{f}} \qquad (1)$$

$$\Phi = \frac{1.5 \times 10^{-5}\ mol}{8.112 \times 10^{-10} \text{ einstein s}^{-1} \times 300 \text{ s} \times 1} = \mathbf{61.6}$$

The quantum yield studies indicate that this is most likely a radical-chain process as evidenced by the Φ value. In other words, the quantum yield value indicated that ~65 equivalents of product are formed for every photon absorbed.

6.3. TEMPO trapping experiment

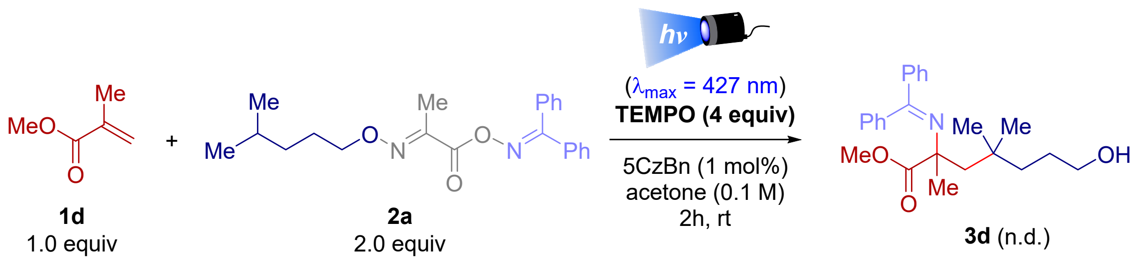

The reaction was performed according to the *General Procedure* using **1a** (5 mg, 0.05 mmol, 1 equiv) and **2a** (34 mg, 0.10 mmol, 2 equiv) in the presence of TEMPO (29 mg, 0.19 mmol, 4.0 equiv). The crude mixtures of the reaction with and without TEMPO were analyzed by GC-MS, showing that the reaction was completely inhibited in the presence of TEMPO, and **2a** was unreactive.

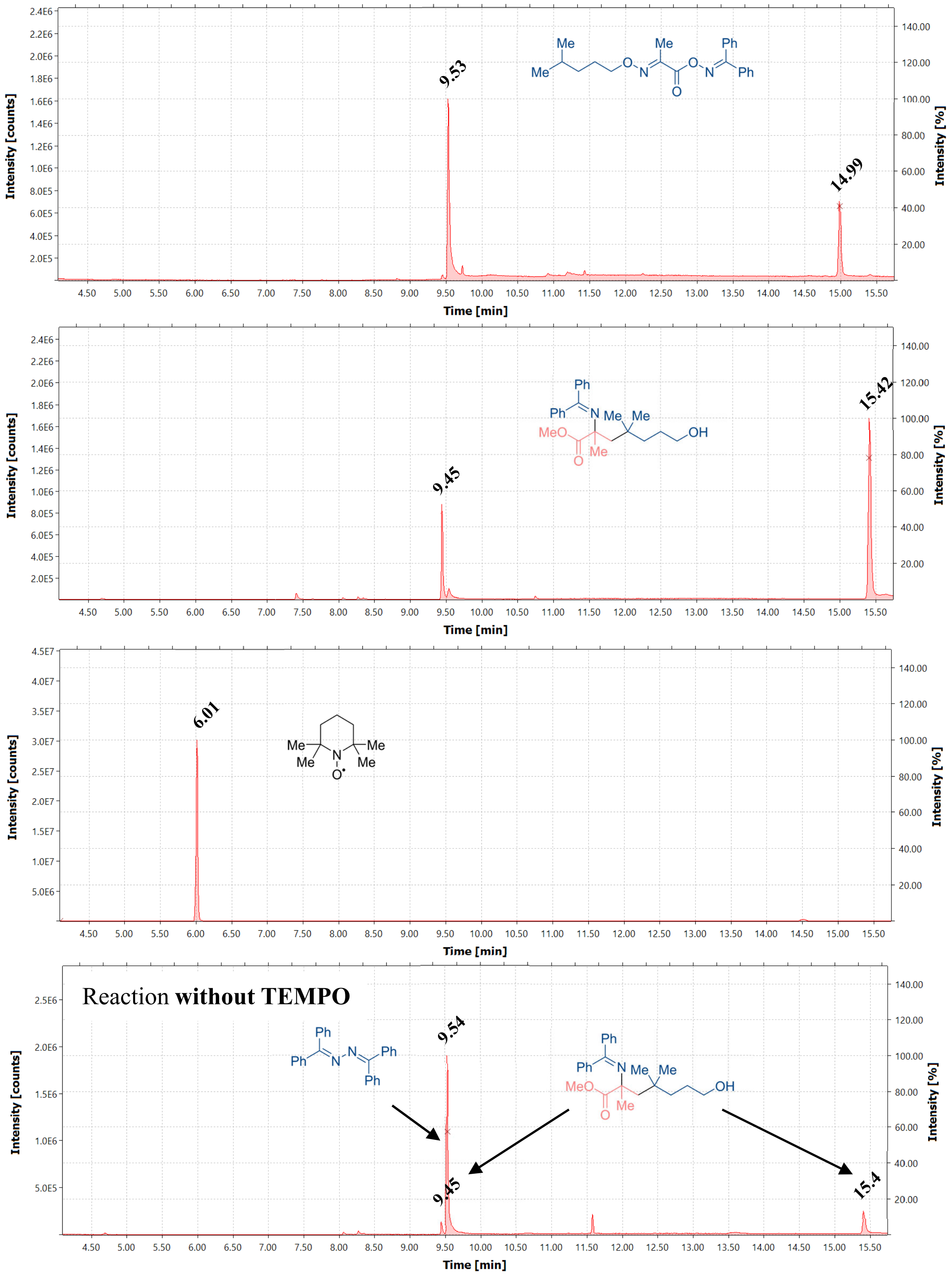

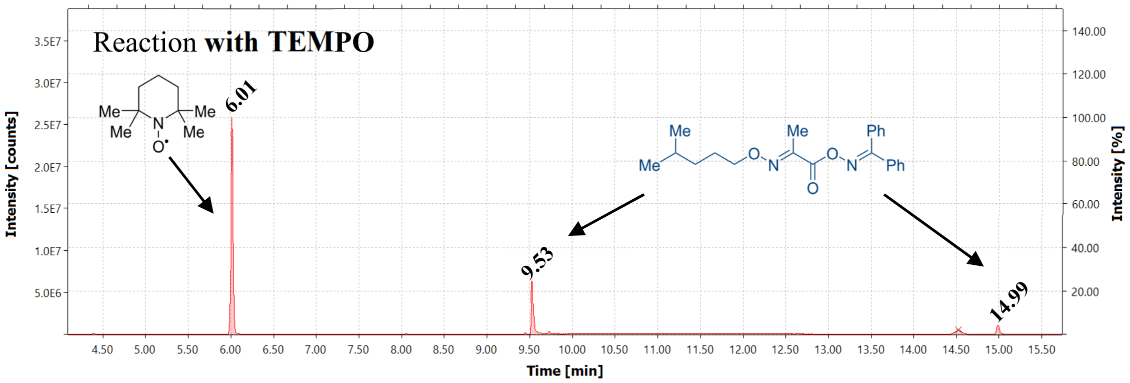

Figure S8. GC-MS spectra of **2a**, final product **3d**, TEMPO, and the reaction mixtures with and without TEMPO.

6.4. Direct Excitation

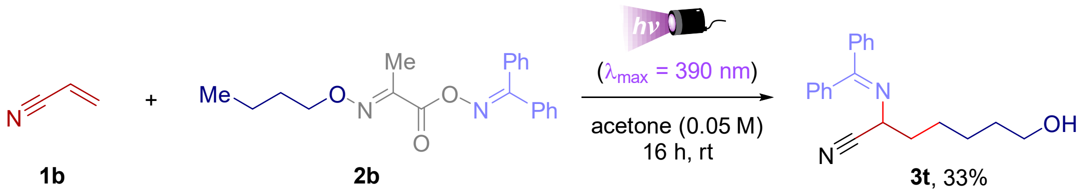

To an 8 mL vial equipped with a magnetic stir bar was added the bifunctional reagent **2b** (41.3 mg, 0.12 mmol, 2.0 equiv). The vial was sealed with a cap containing a Sil/PTFE septum, evacuated, and backfilled with nitrogen. After this process was repeated 3 times, anhydrous degassed acetone (1.2 mL, 0.05 M), and alkene **1** (0.06 mmol, 1.0 equiv) were added via syringe. The reaction mixture was irradiated with a Kessil PR160-purple LED lamp (30 W High Luminous DEX 2100 LED, λ_{max} = 390 nm) for 16 h as described in the "Workflow" section. The lamp was placed 4 cm away from the reaction vials, and cooled at room temperature by an external fan. Upon completion, the volatiles were removed under reduced pressure, and the crude was analyzed using 1,3,5-trimethoxybenzene as internal standard. Final **3t** product was formed in 33% ^{1}H NMR yield, while 66% of starting bifunctional reagent **2b** remained.

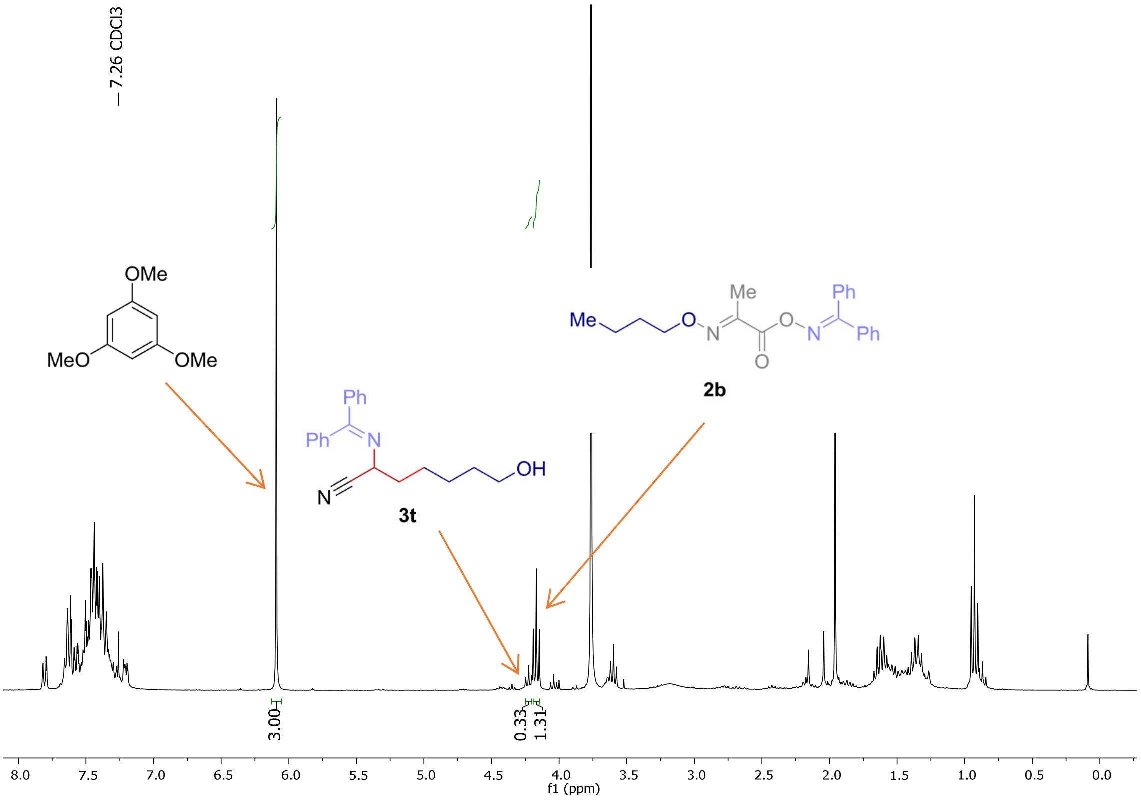

Figure S9. ^{1}H NMR of the reaction mixture with 1,3,5-trimethoxybenzene.

6.5.Light ON / OFF experiment

The reaction was performed under standard conditions using acrylonitrile and **2c** as model substrate. Each 5 or 15 min, an aliquot was analyzed by ^{1}H NMR using trimethoxybenzene as internal standard. The data was plotted resulting in the following graphic:

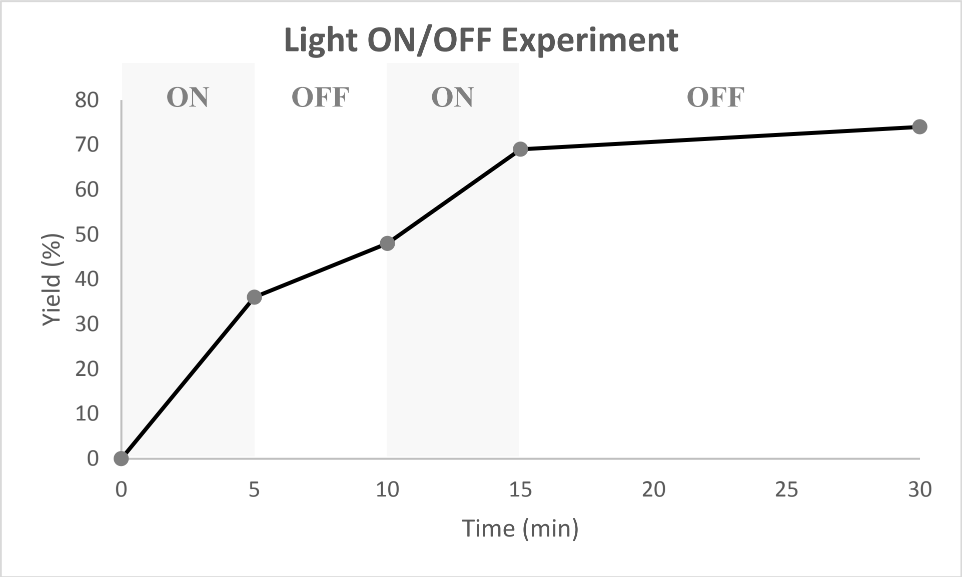

Figure S10. Light ON/OFF experiment.

6.6. Akyl radical proof experiment

To prove the involvement of alkyl radical process a radical clock experiment was carried out using methyl (*E*)-penta-2,4-dienoate as an alkene and bifunctional oxime **2t**. Under the optimized conditions a 50/50 mixture of iminyl acohols **3x** and **8x** was observed by ^{1}H-NMR. The formation of iminyl alcohol **8x** can be explained by the initial formation of the radical intermediate **D**, that could be delocalized to a second radical intermediate **D'** to give the 1,8-iminyl alcohol upon reaction with the iminyl radical.

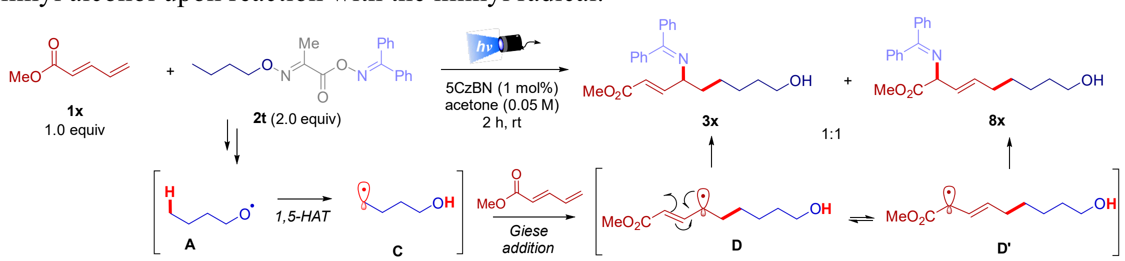

Figure S11

8x: 5.77 (ddt, *J* = 15.4, 6.6, 1.4 Hz, H^3)
5.49 (dtd, *J* = 15.4, 6.8, 1.2 Hz, H^2)
4.62 (dd, *J* = 6.6, 1.1 Hz, H^1)

3x: 5.82 (dd, *J* = 15.7, 1.6 Hz, H^3)
3,98 (m, H^1)

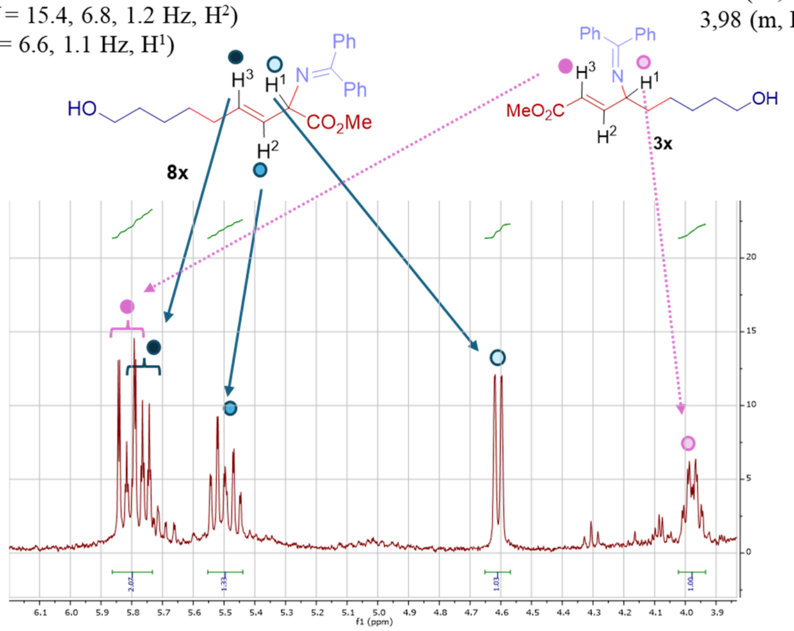

^{1}H NMR (300 MHz, CDCl$_3$), δ (ppm)

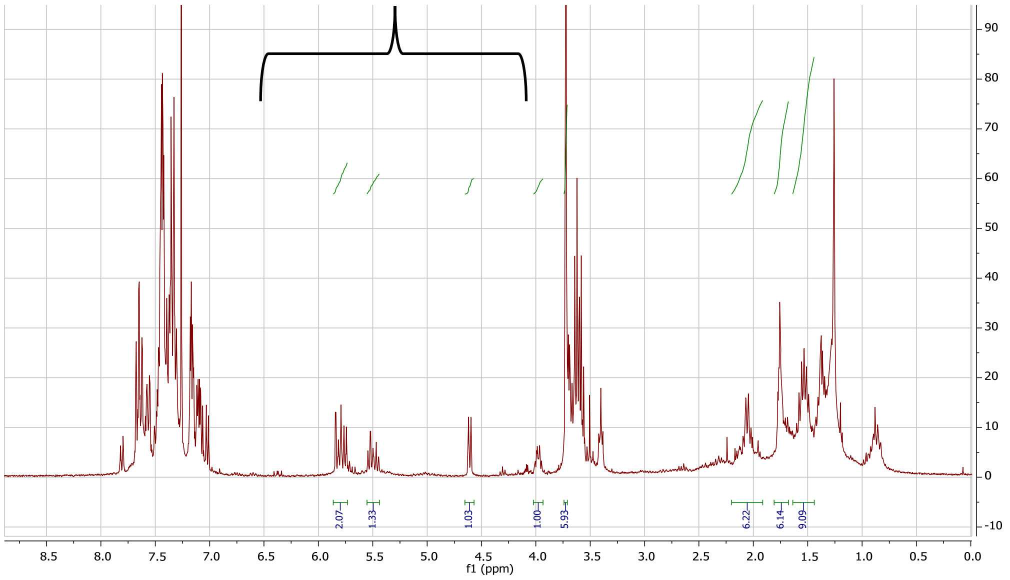

Figure S12

6.7. Proposed mechanism and detailed intermediates from D to final compound 3

The mechanism was proposed based on the experimental investigations, together with the theoretical calculations performed in our previous work by dispersion corrected density functional theory (DFT).[6] The radical intermediate **D** can react with the bifunctional reagent **2** *via* TSD, forming the radical intermediate **E**. Moreover, intermediate **E** affords the desired 1,6-amino alcohol **3** *via* TSE through the release of CO_2, MeCN and the *O*-radical **A**, regenerating the cycle.

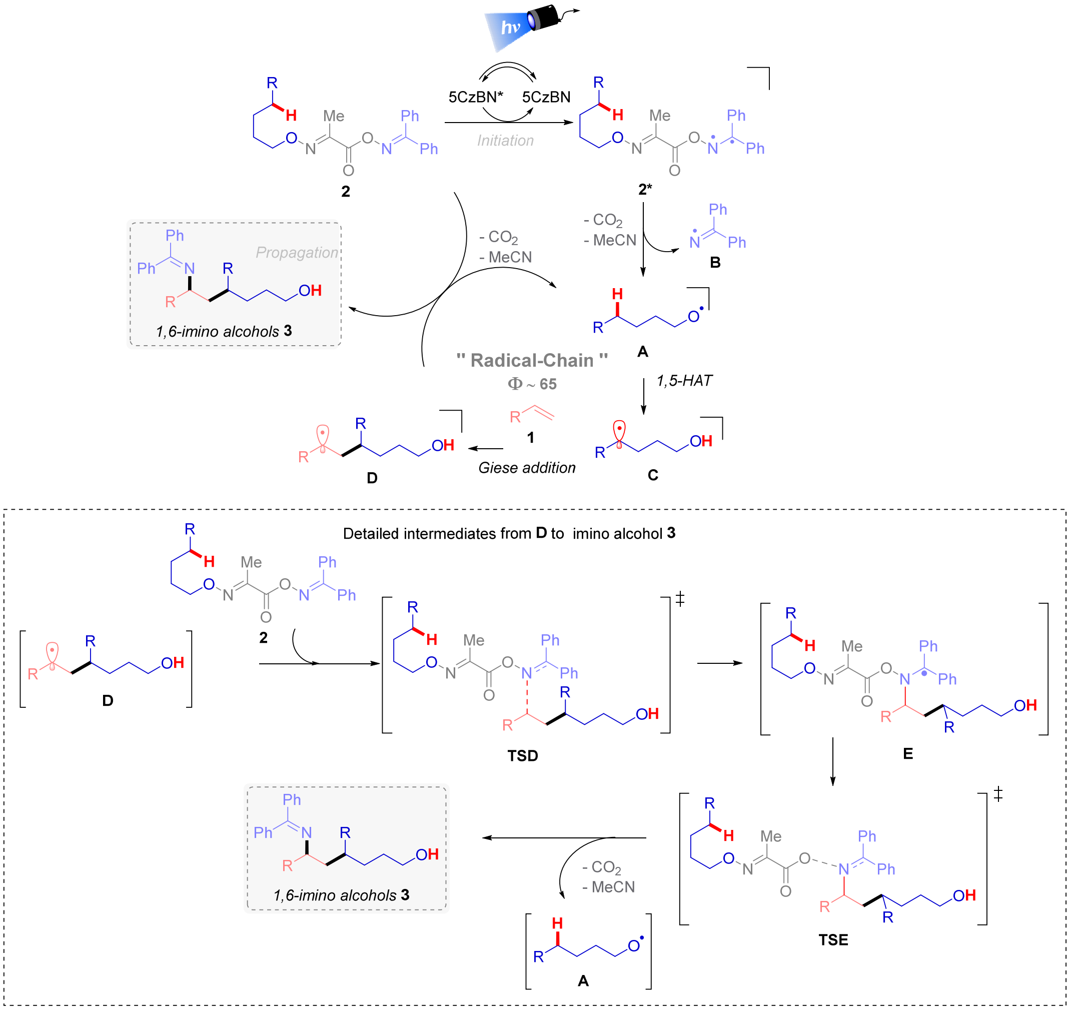

Figure S13

This proposed mechanism is consistent with previous reports on similar transformation,[8] and with the light on/off experiment.

[8] (a) Majhi, J.; Dhungana, R. K.; Rentería-Gómez, Á.; Sharique, M.; Li, L.; Dong, W.; Gutierrez, O.; Molander, G. A. Metal-Free Photochemical Imino-Alkylation of Alkenes with Bifunctional Oxime Esters. *J. Am. Chem. Soc.* **2022**, *144*, 15871-15878. (b) Laskar, R.; Dutta, S.; Spies, J. C.; Mukherjee, P.; Rentería-Gómez, A.; Thuelemann, R. E.; Daniliuc, C. G.; Gutierrez, O.; Glorius, F. γ-Amino Alcohols via Energy Transfer Enabled Brook Rearrangement. *J. Am. Chem. Soc.* **2024**, *146*, 10899-10907. (c) Ghosh, S. K.; Hu, M.; Comito R. J., One-Pot Synthesis of Primary and Secondary Aliphatic Amines via Mild and Selective sp3 C-H Imination, *Chem. Eur. J.* **2021**, *27*, 17601-17608.

7 NMR Spectra

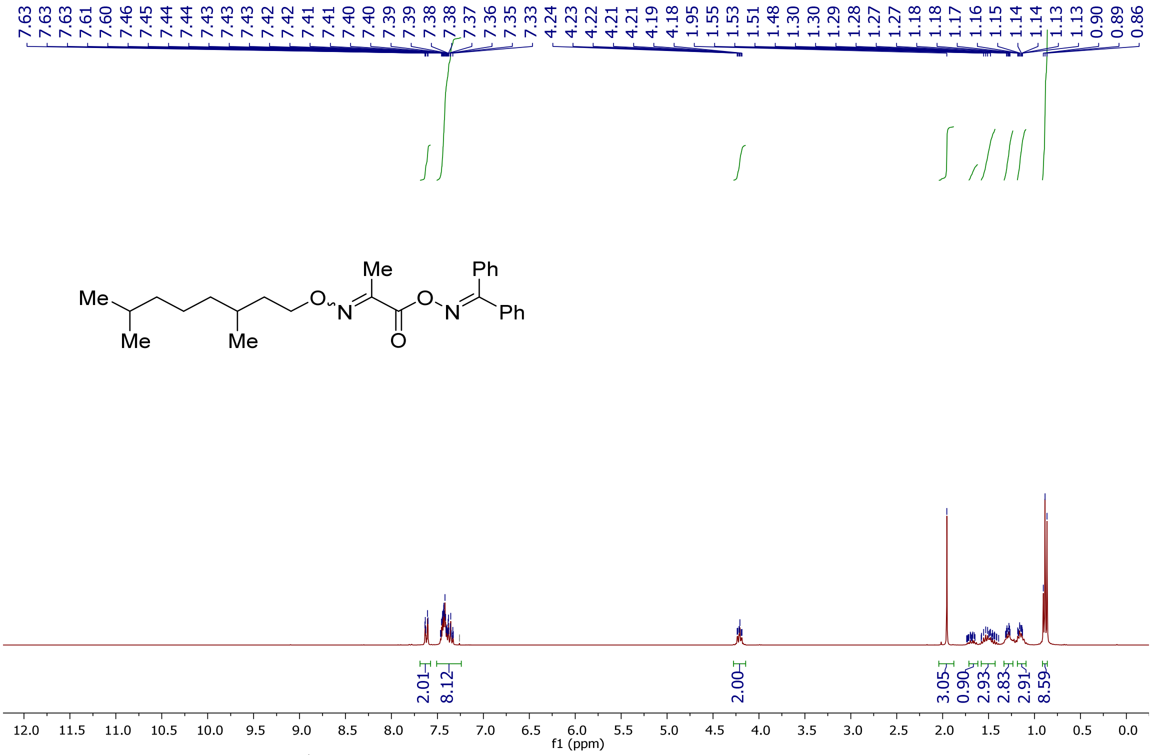

^{1}H NMR (300 MHz, CDCl$_3$) of compound **2n**.

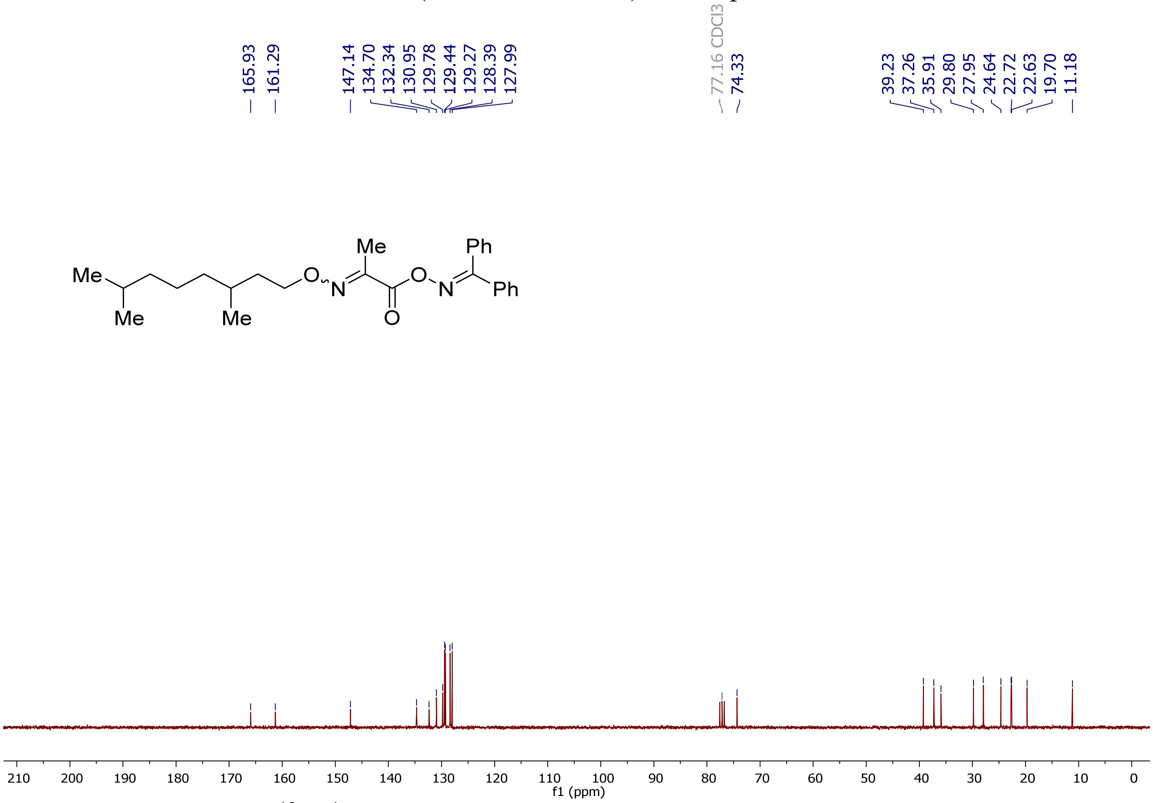

^{13}C{^{1}H} NMR (76 MHz, CDCl$_3$) of compound **2n.**

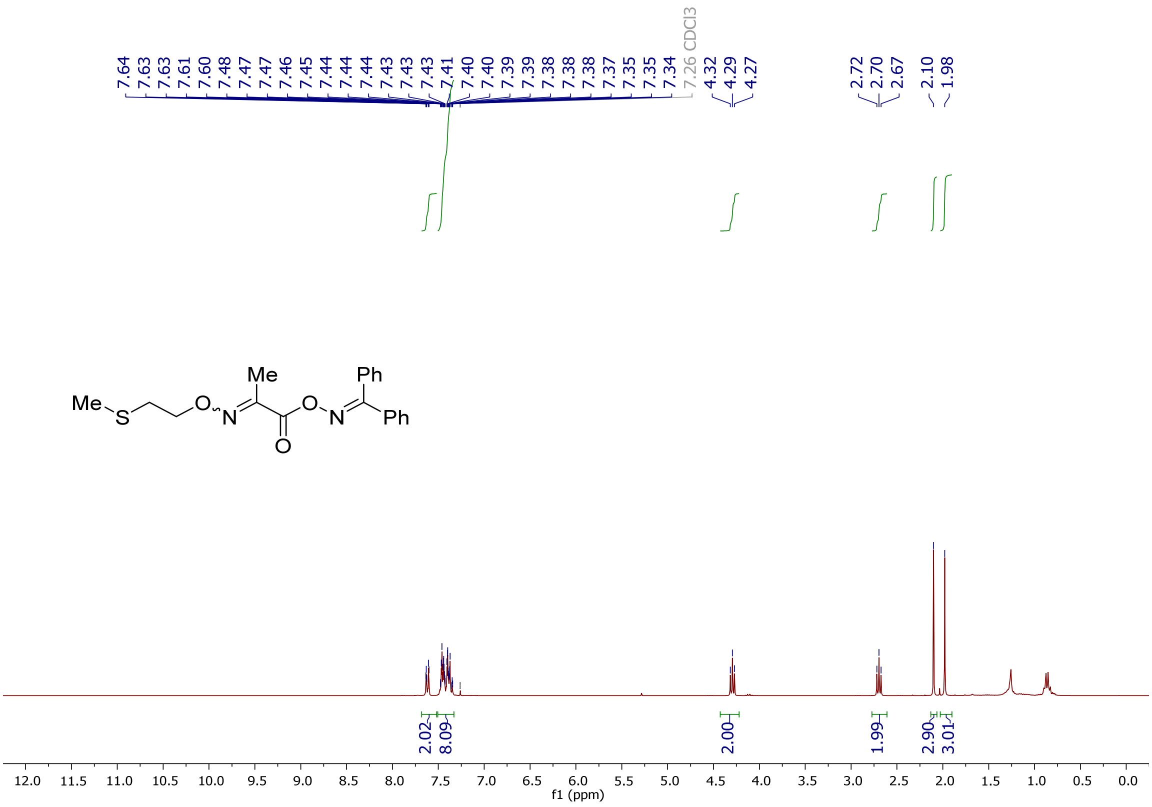

^{1}H NMR (300 MHz, $CDCl_3$) of compound **2p**.

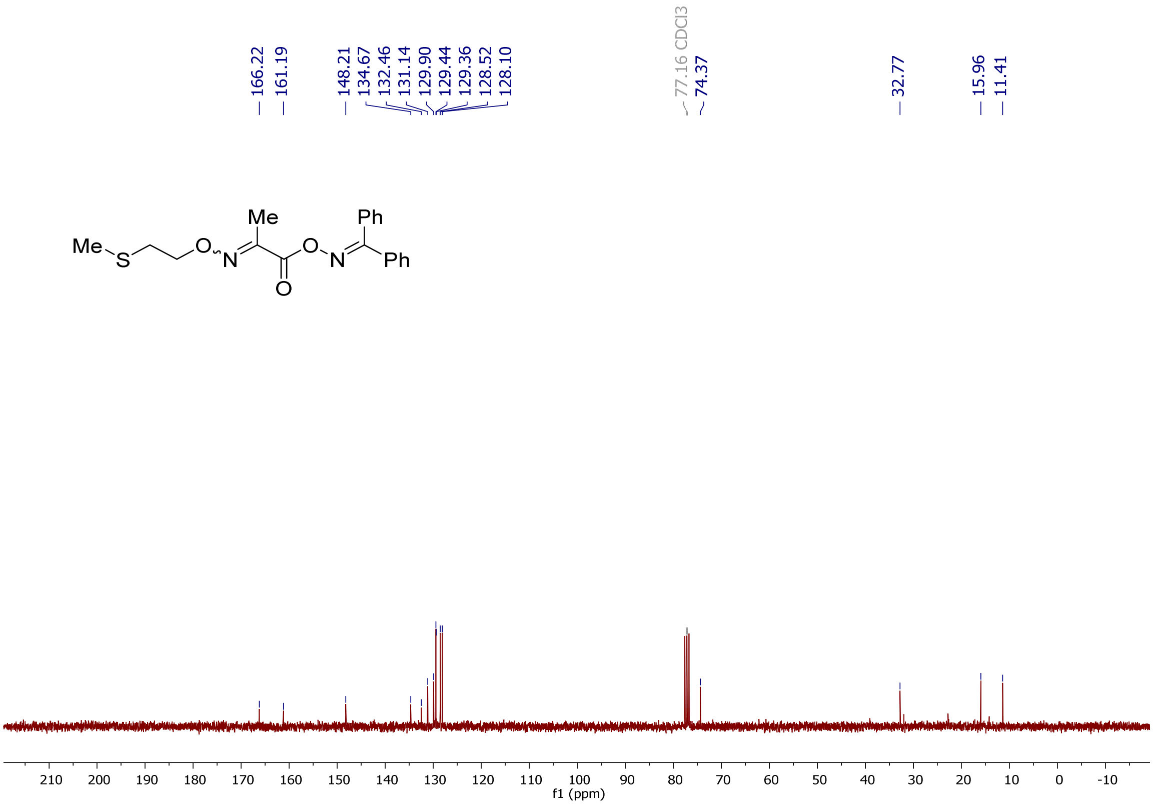

^{13}C{^{1}H} NMR (76 MHz, $CDCl_3$) of compound **2p.**

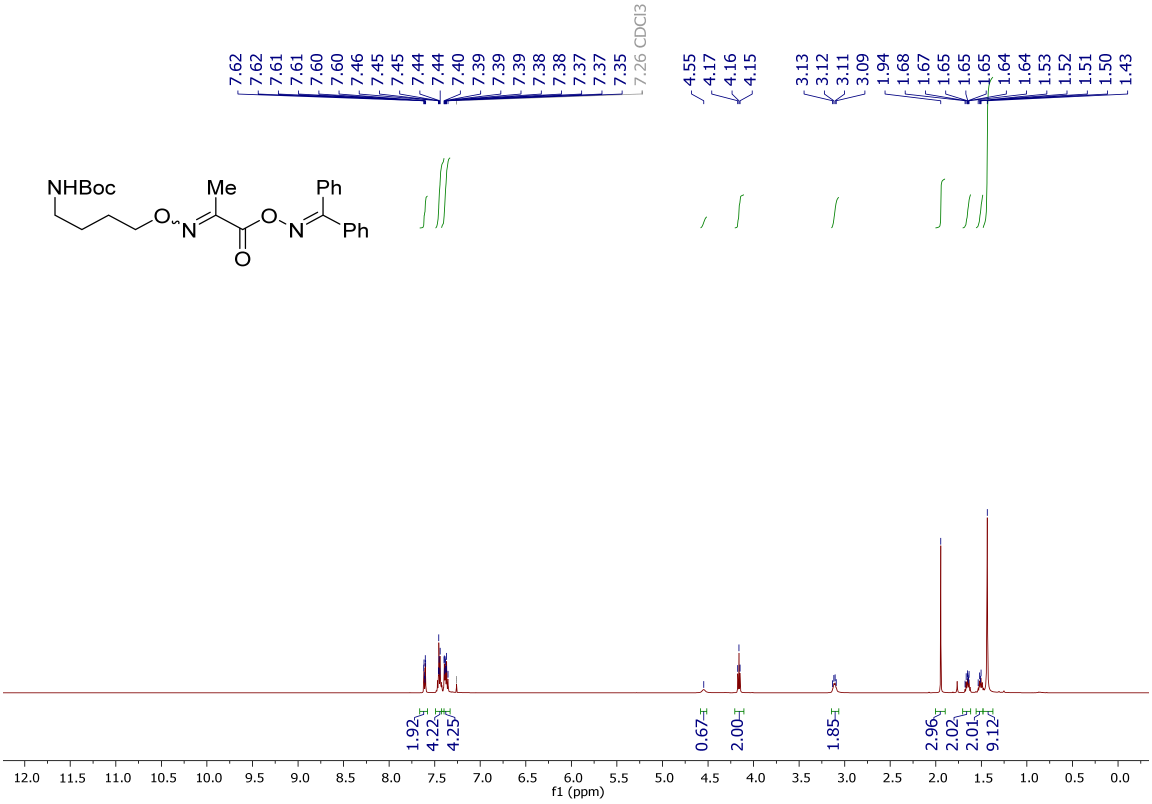

^{1}H NMR (500 MHz, $CDCl_3$) of compound **2q**.

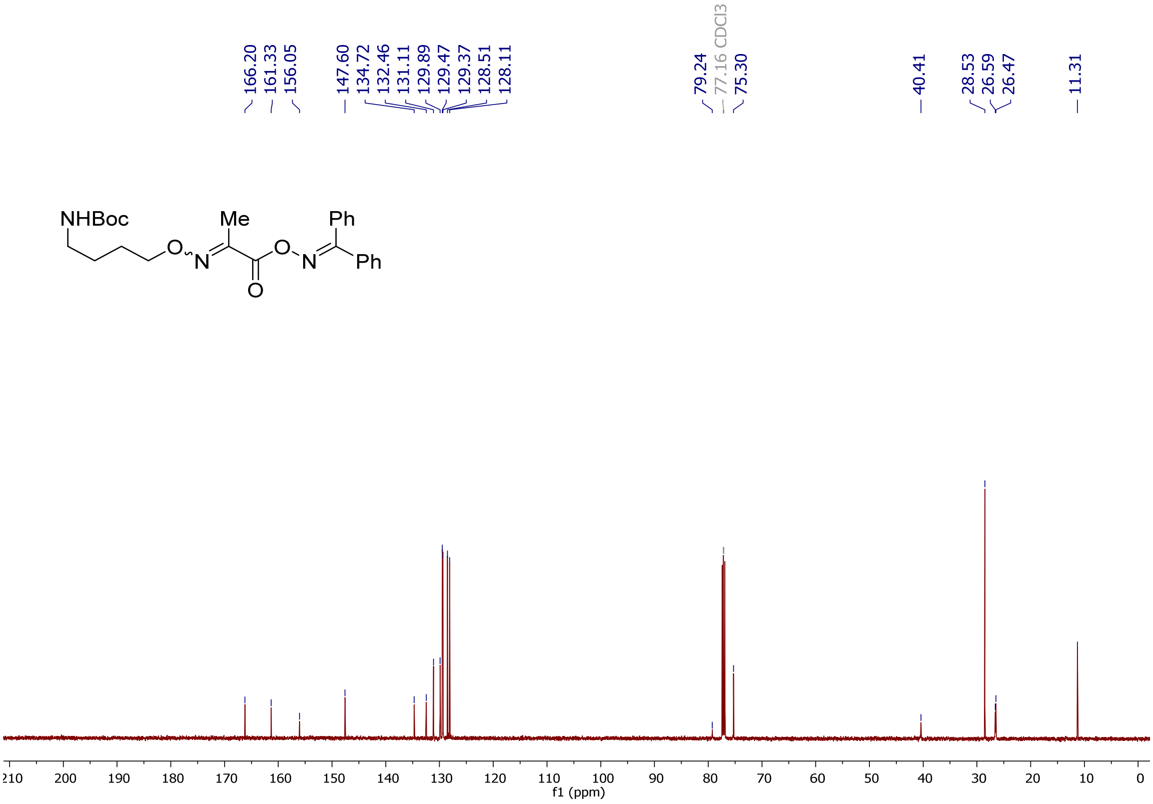

$^{13}C\{^1H\}$ NMR (126 MHz, $CDCl_3$) of compound **2q.**

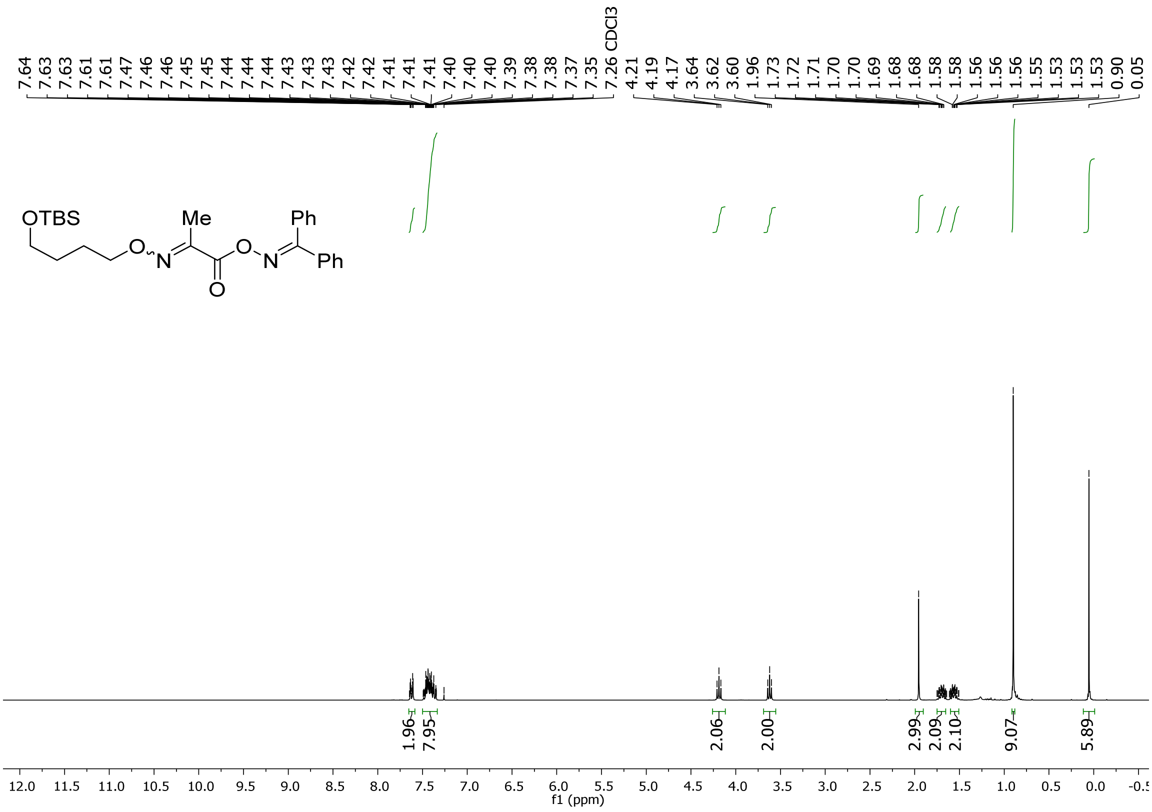

^{1}H NMR (300 MHz, $CDCl_3$) of compound **2r**.

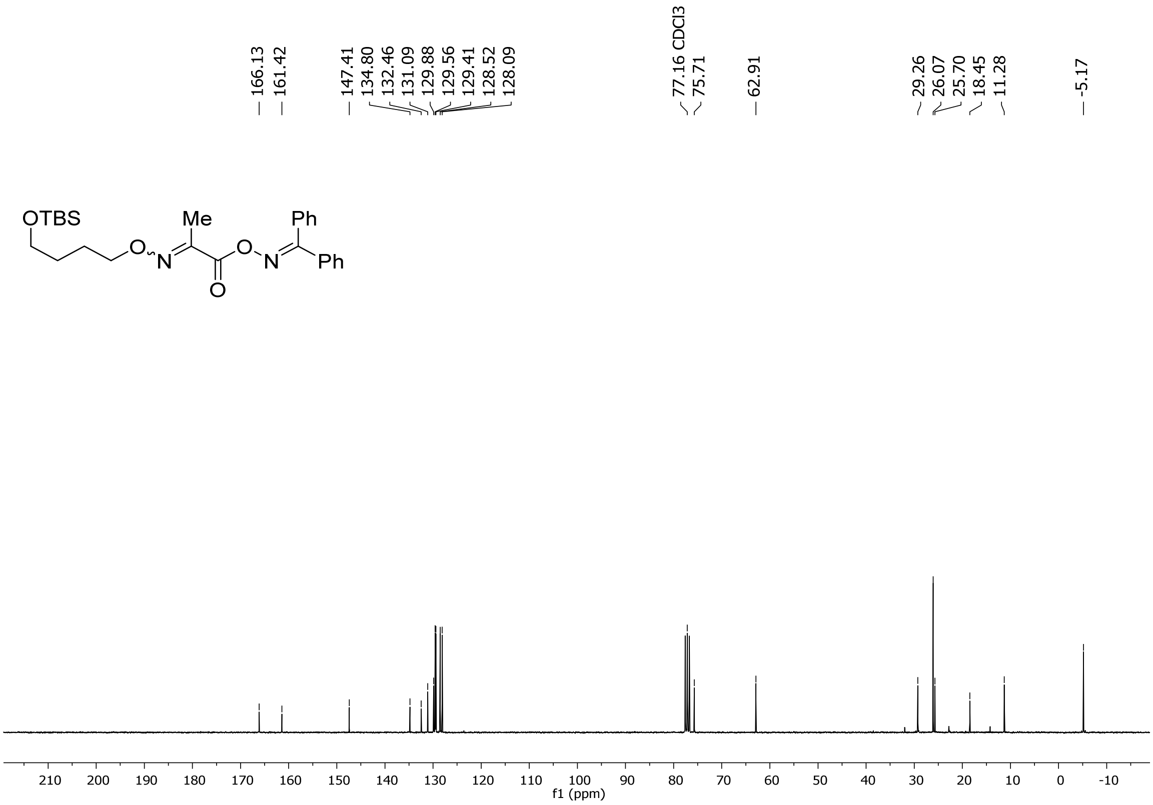

^{13}C{^{1}H} NMR (75 MHz, $CDCl_3$) of compound **2r.**

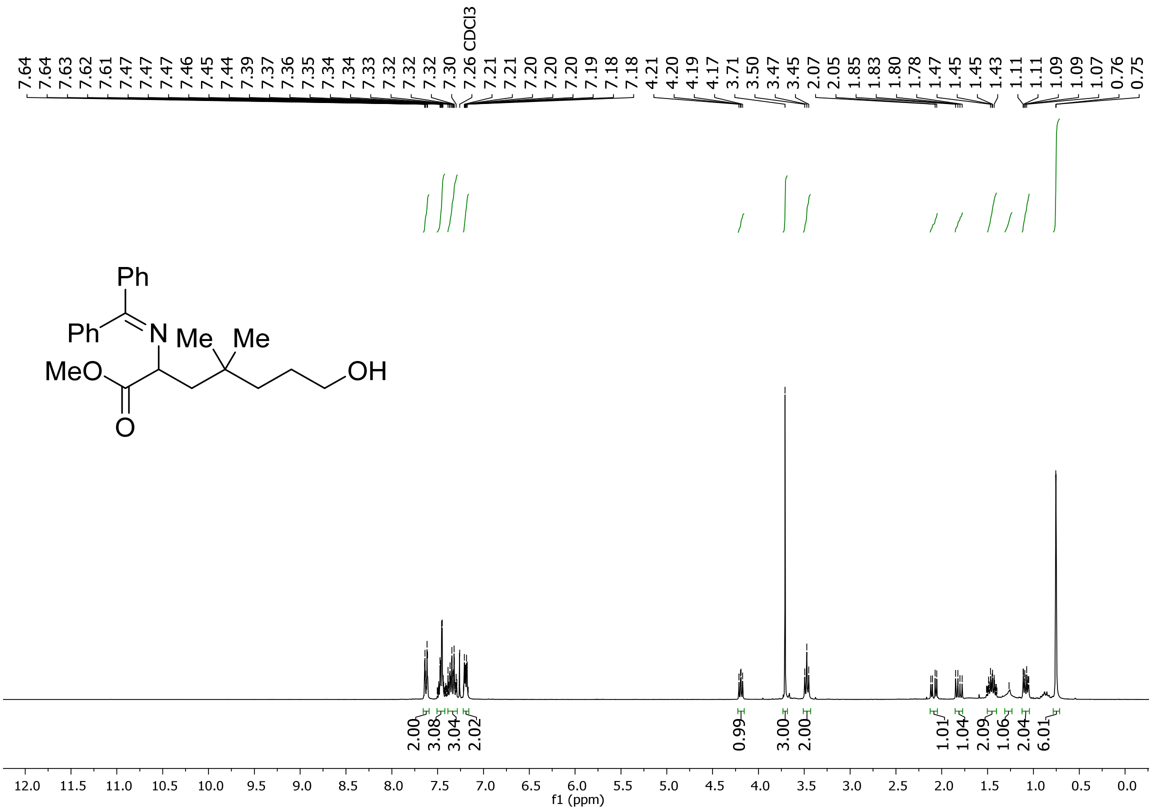

^{1}H NMR (300 MHz, CDCl$_3$) of compound **3a**.

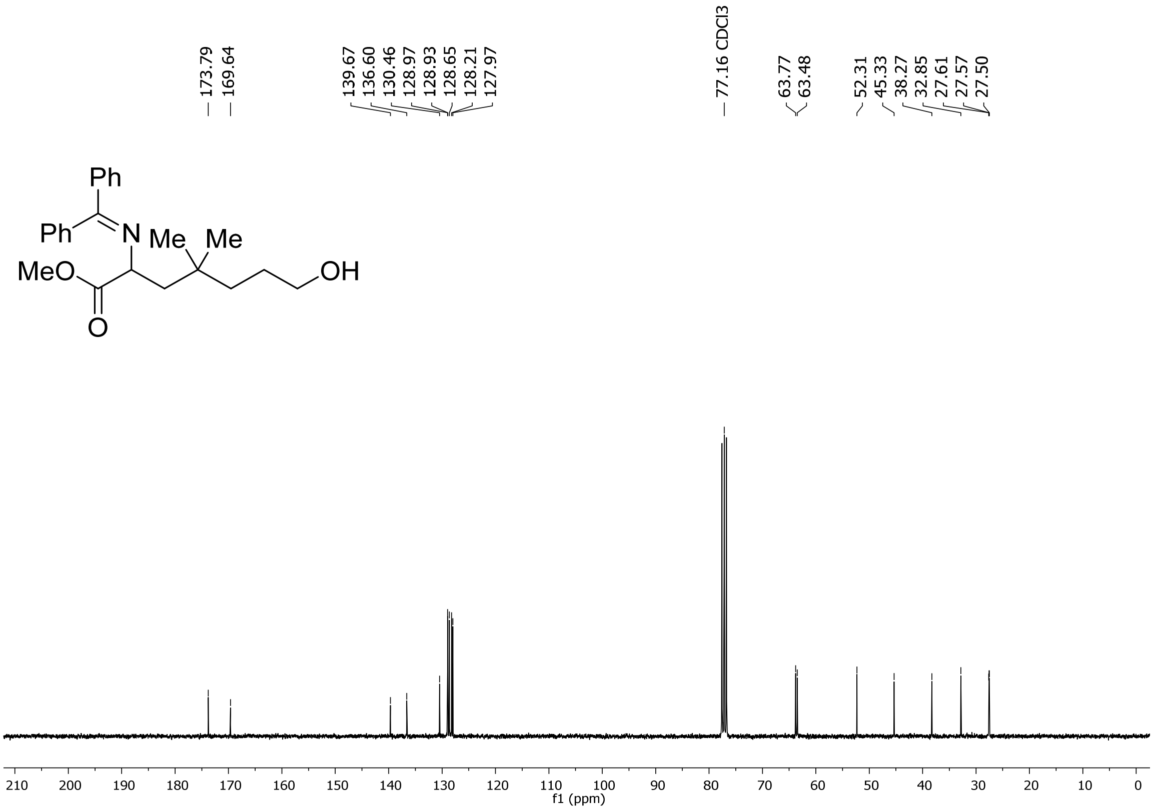

^{13}C{^{1}H} NMR (75 MHz, CDCl$_3$) of compound **3a.**

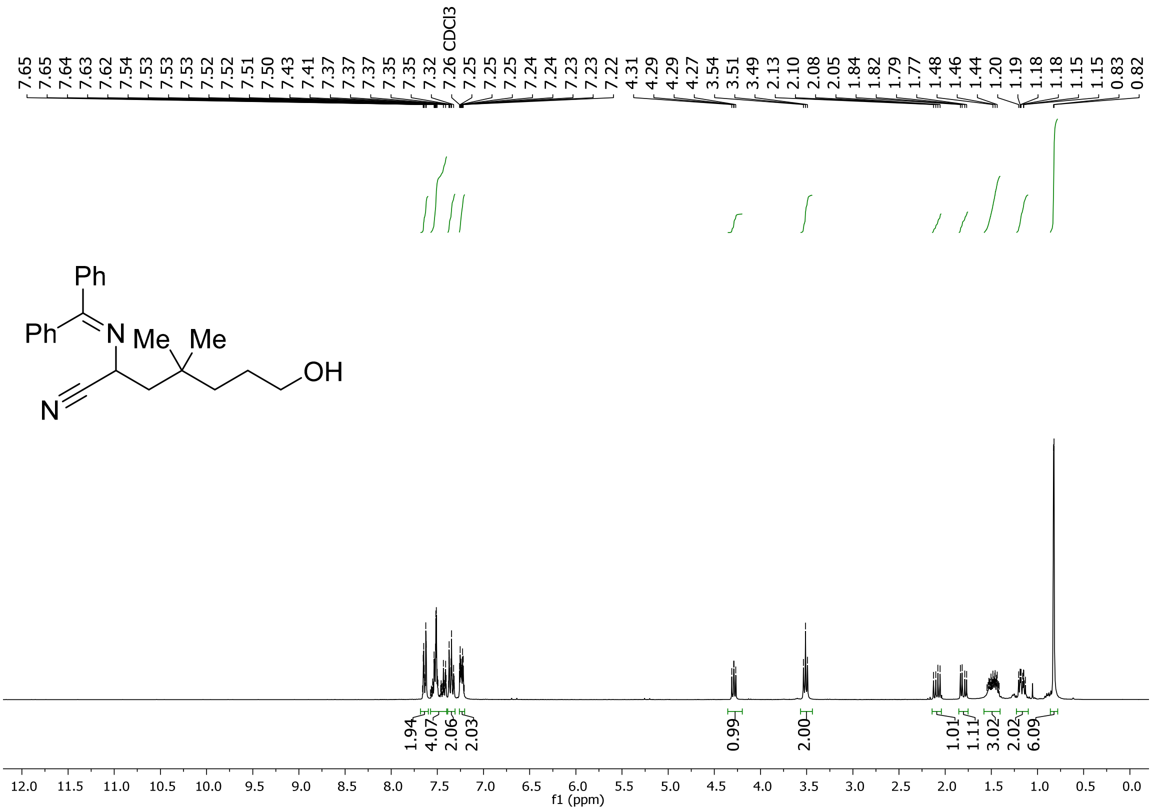

^{1}H NMR (300 MHz, $CDCl_3$) of compound **3b**.

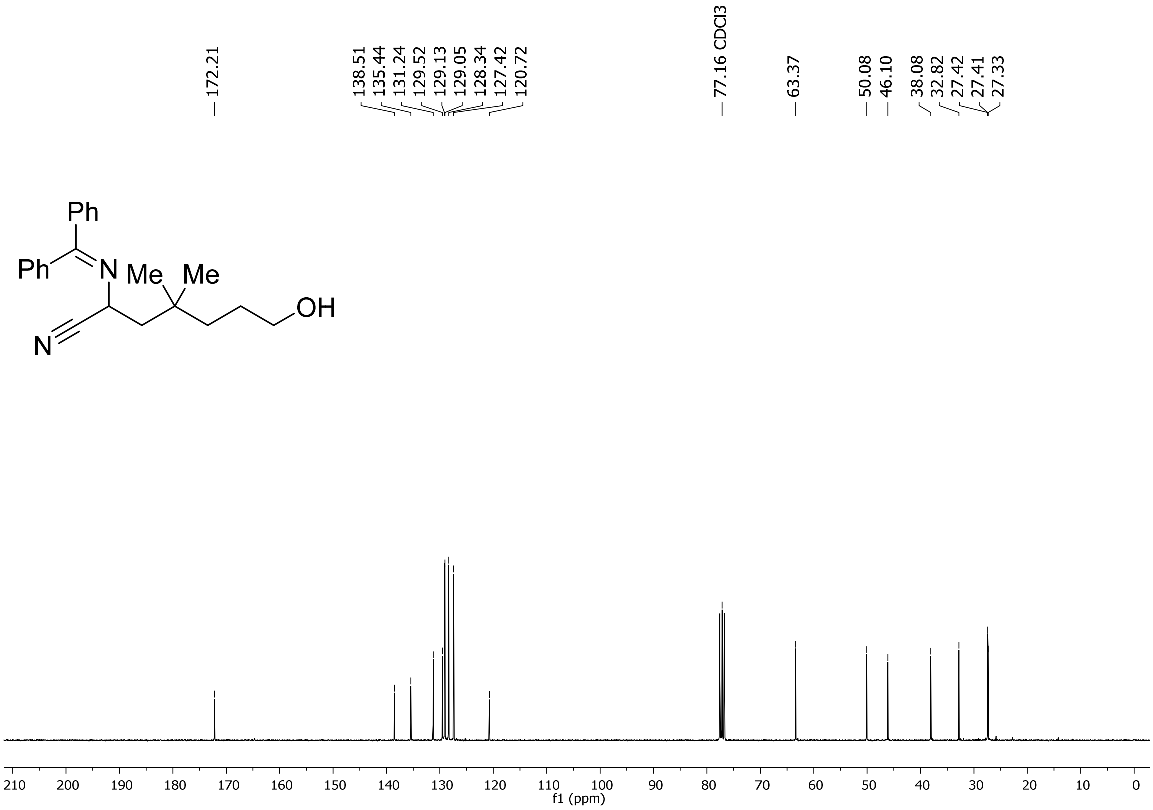

^{13}C{^{1}H} NMR (75 MHz, $CDCl_3$) of compound **3b.**

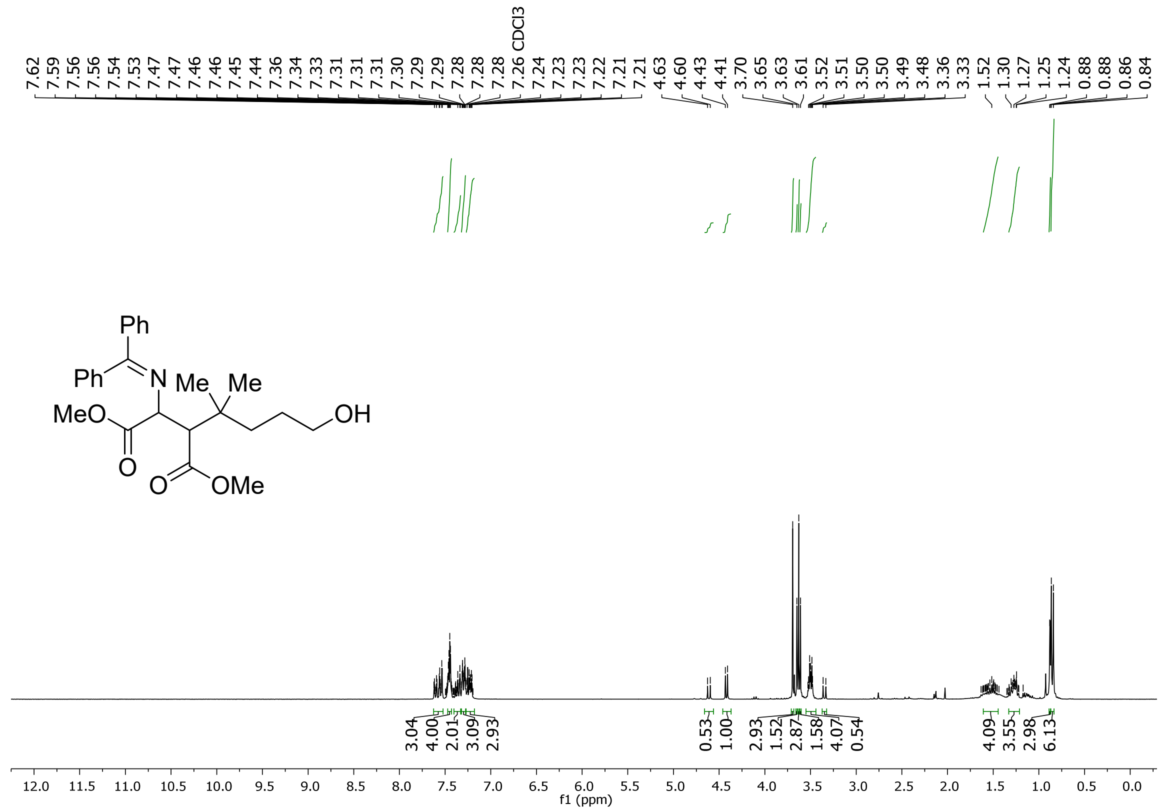

^{1}H NMR (300 MHz, $CDCl_3$) of compound **3c**.

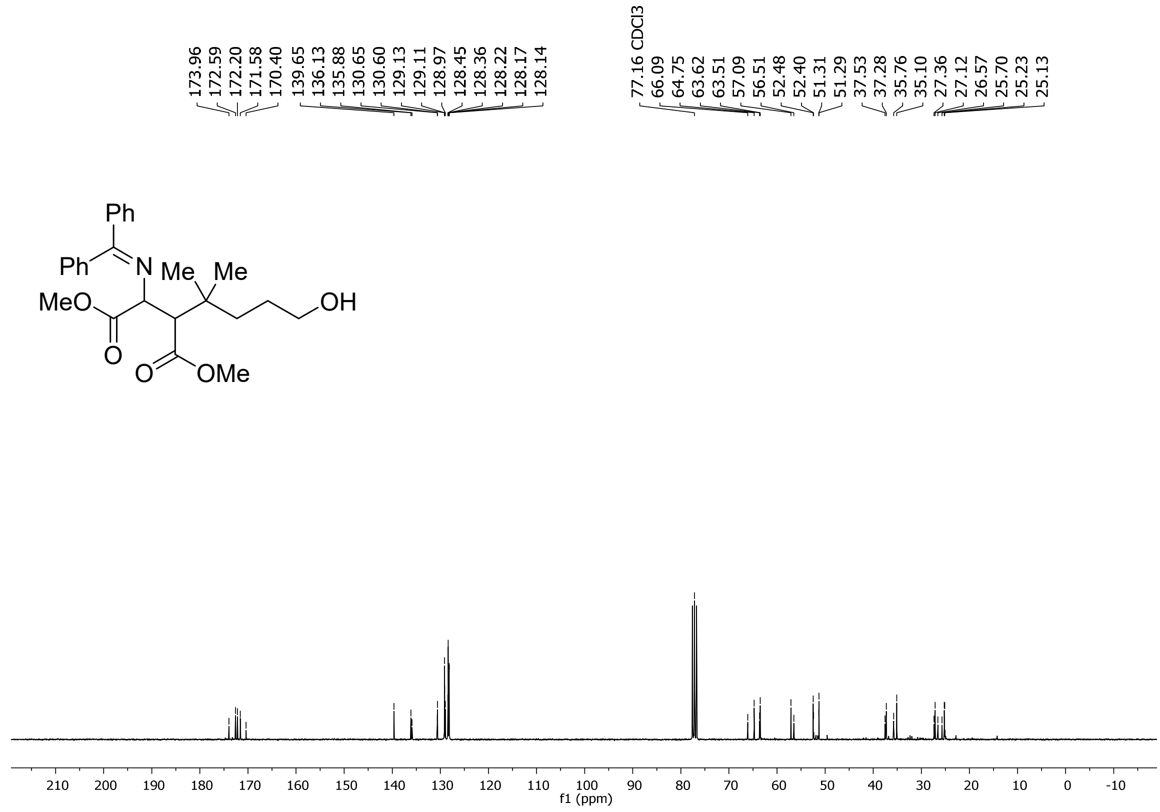

^{13}C{^{1}H} NMR (75 MHz, $CDCl_3$) of compound **3c.**

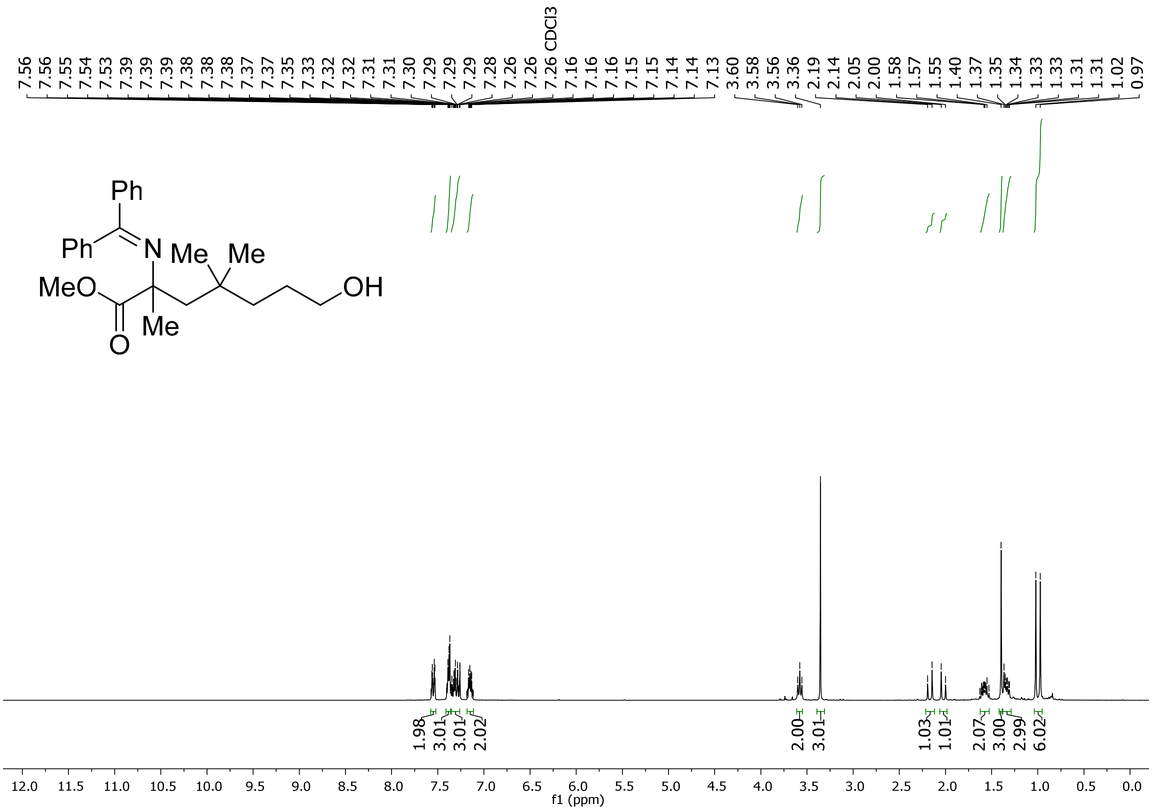

^{1}H NMR (300 MHz, $CDCl_3$) of compound **3d**.

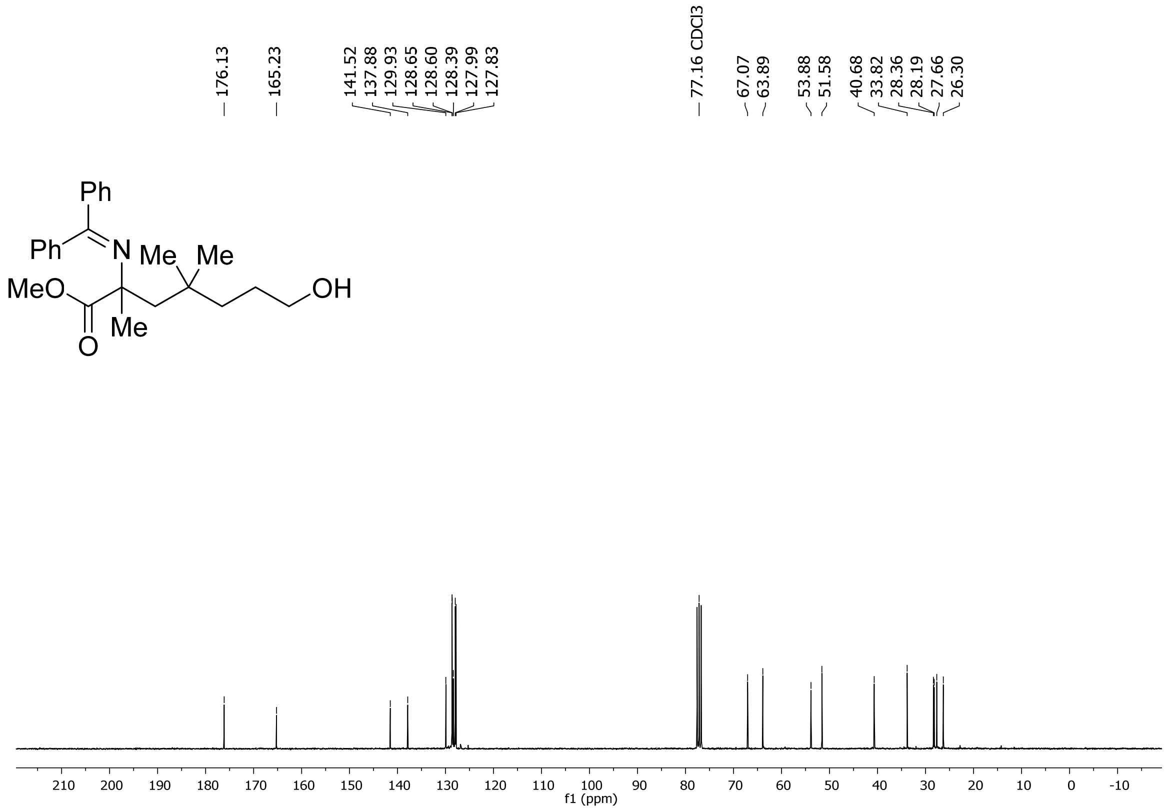

$^{13}C\{^{1}H\}$ NMR (76 MHz, $CDCl_3$) of compound **3d.**

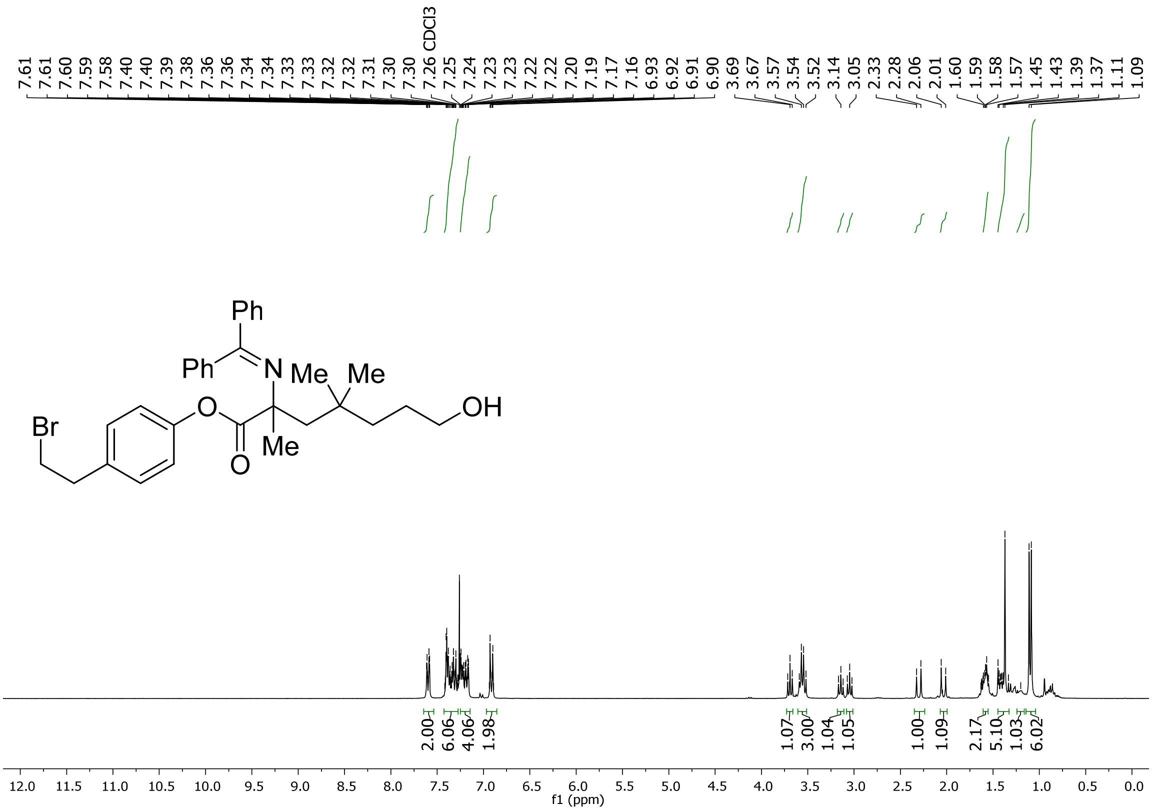

^{1}H NMR (300 MHz, $CDCl_3$) of compound **3e**.

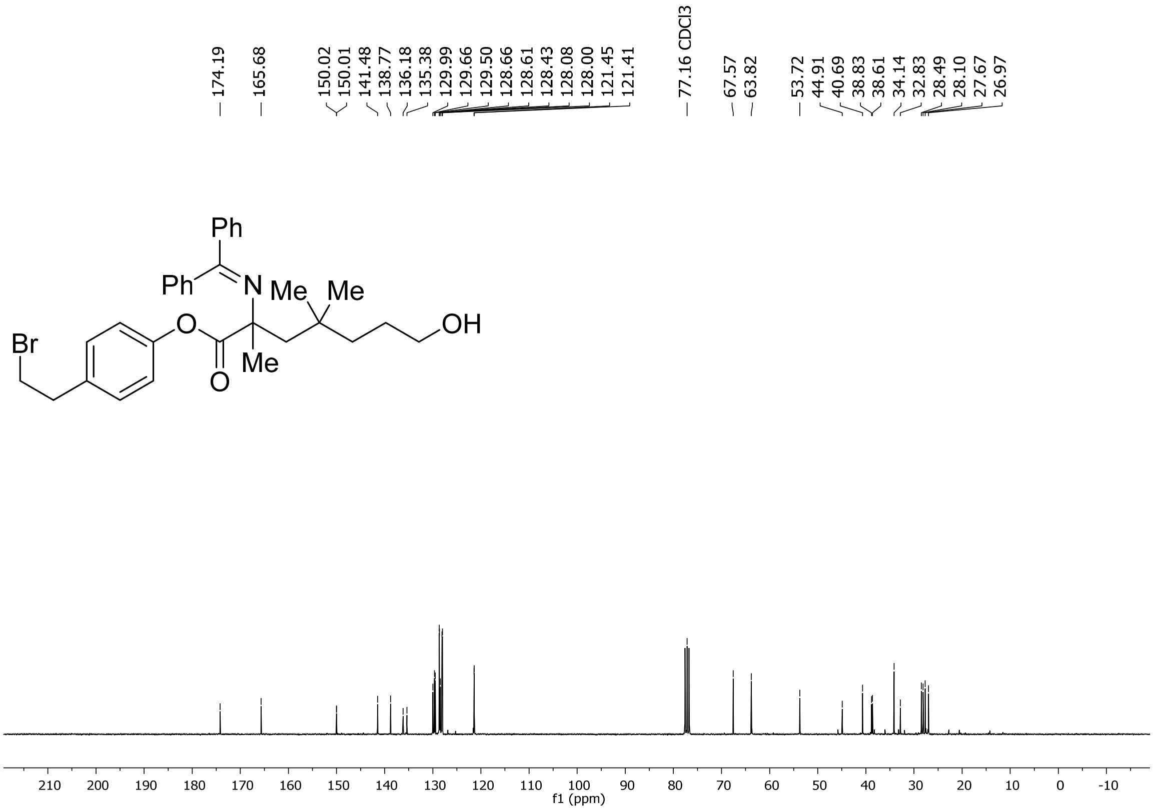

^{13}C{^{1}H} NMR (75 MHz, $CDCl_3$) of compound **3e.**

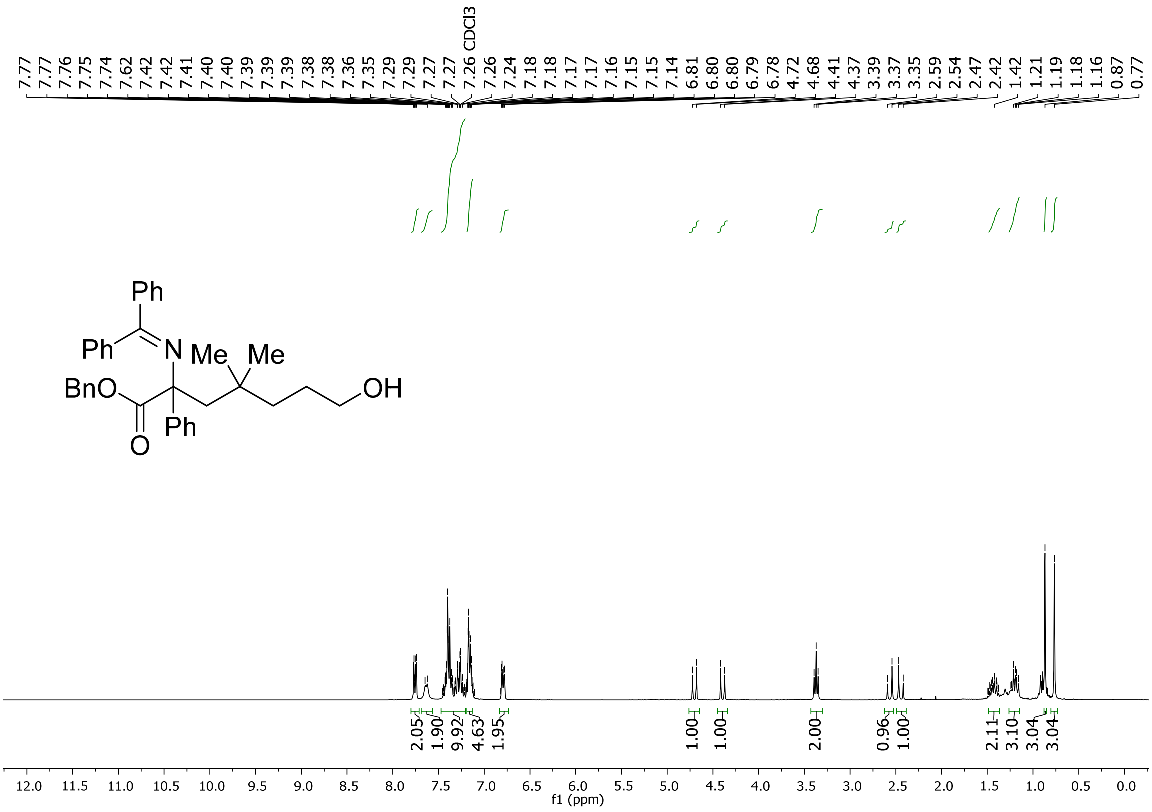

^{1}H NMR (300 MHz, $CDCl_3$) of compound **3f**.

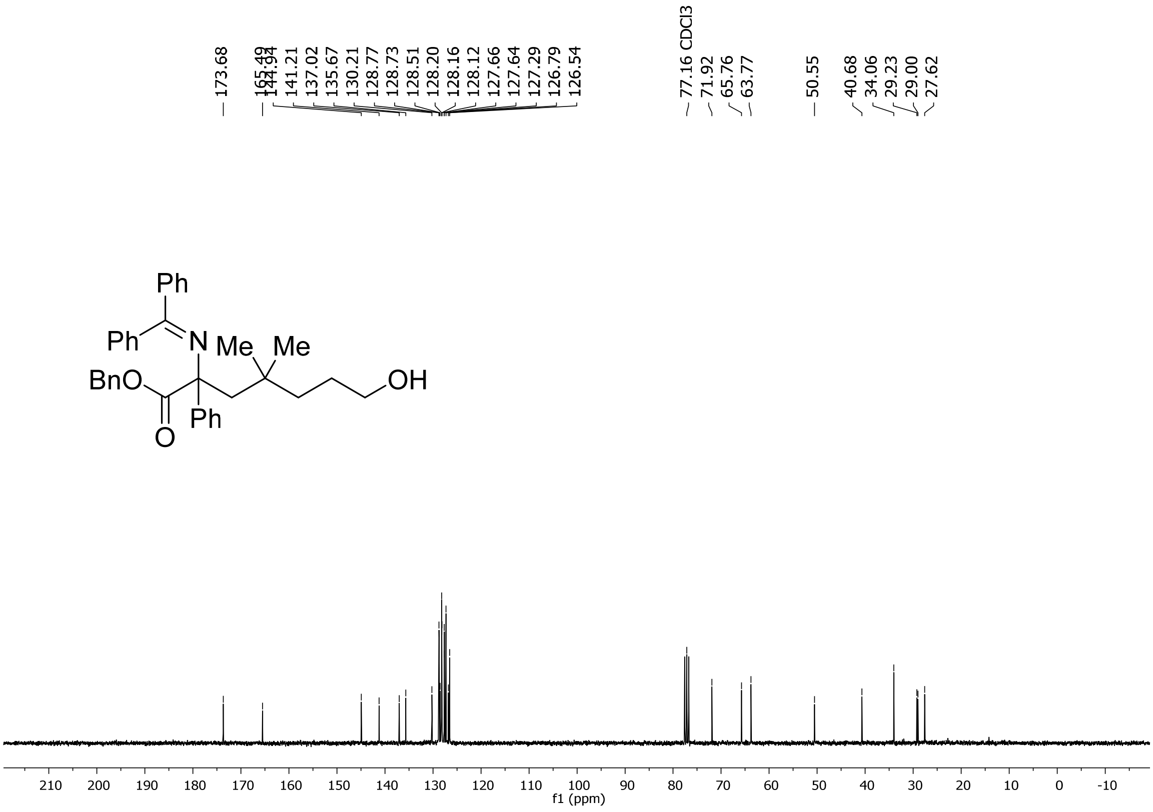

^{13}C{^{1}H} NMR (76 MHz, $CDCl_3$) of compound **3f.**

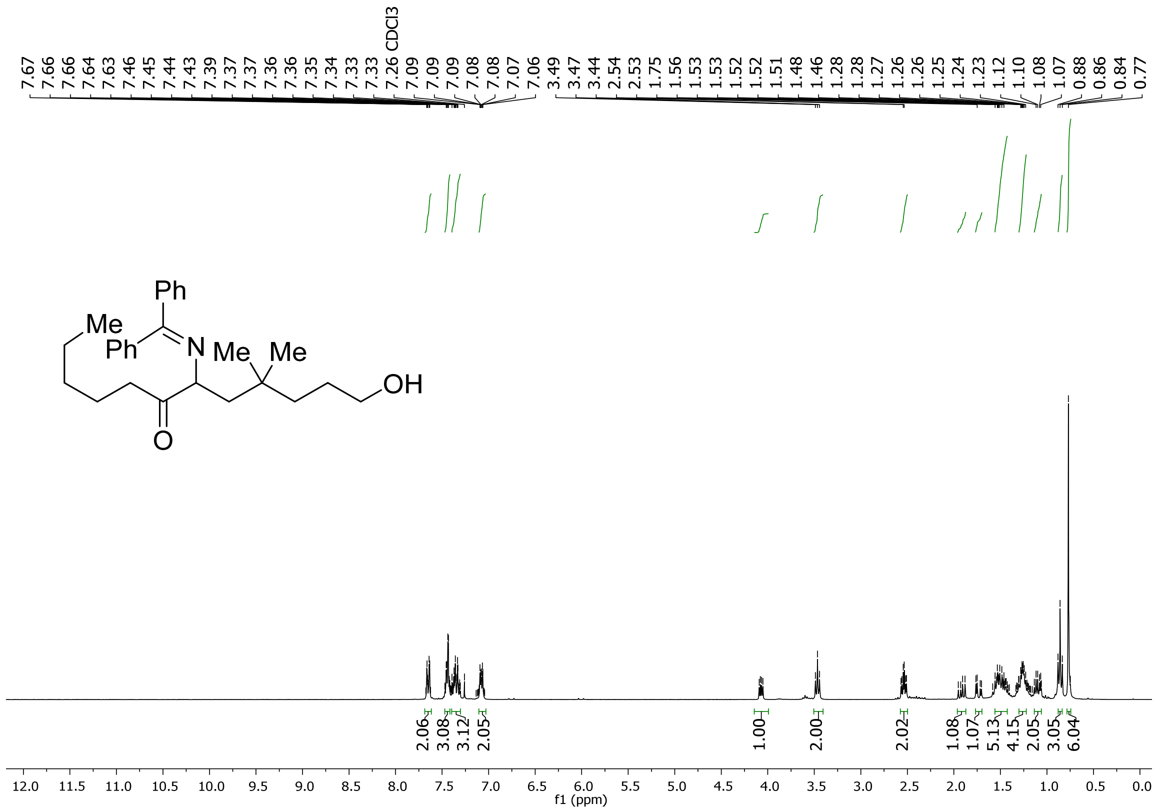

^{1}H NMR (300 MHz, $CDCl_3$) of compound **3g**.

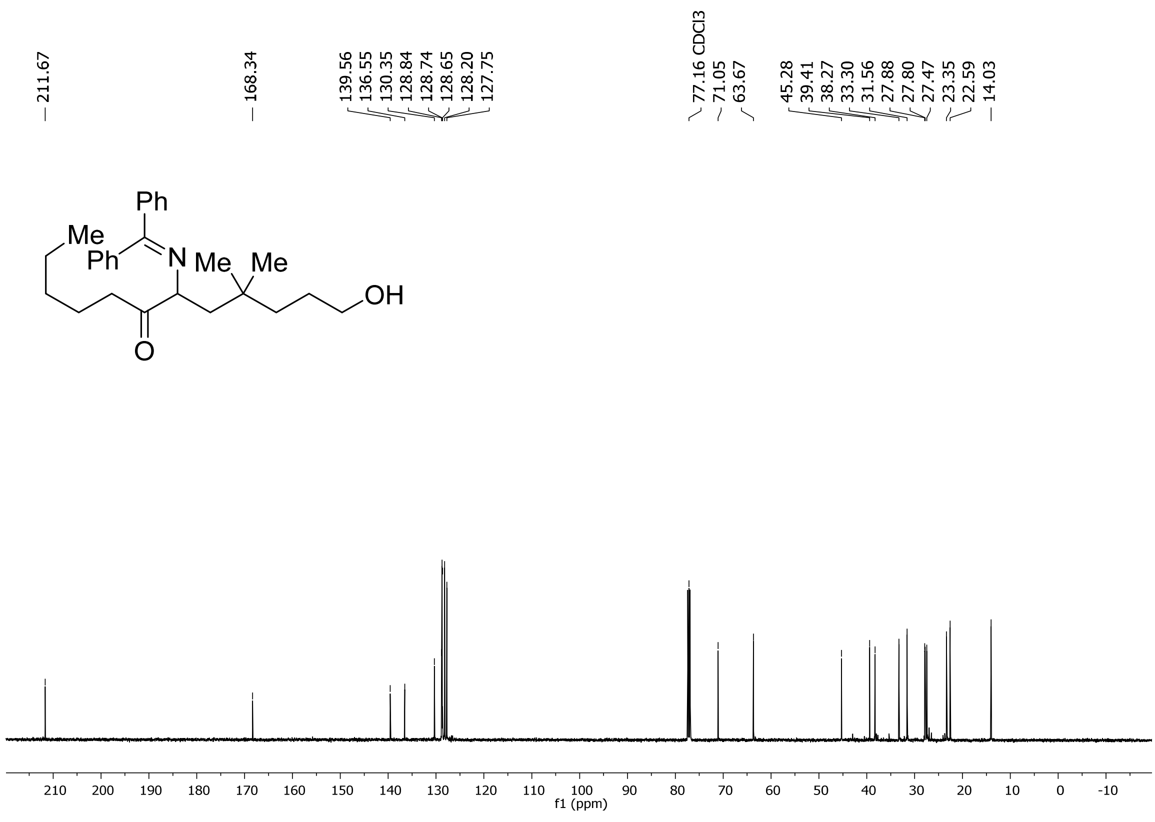

^{13}C{^{1}H} NMR (126 MHz, $CDCl_3$) of compound **3g.**

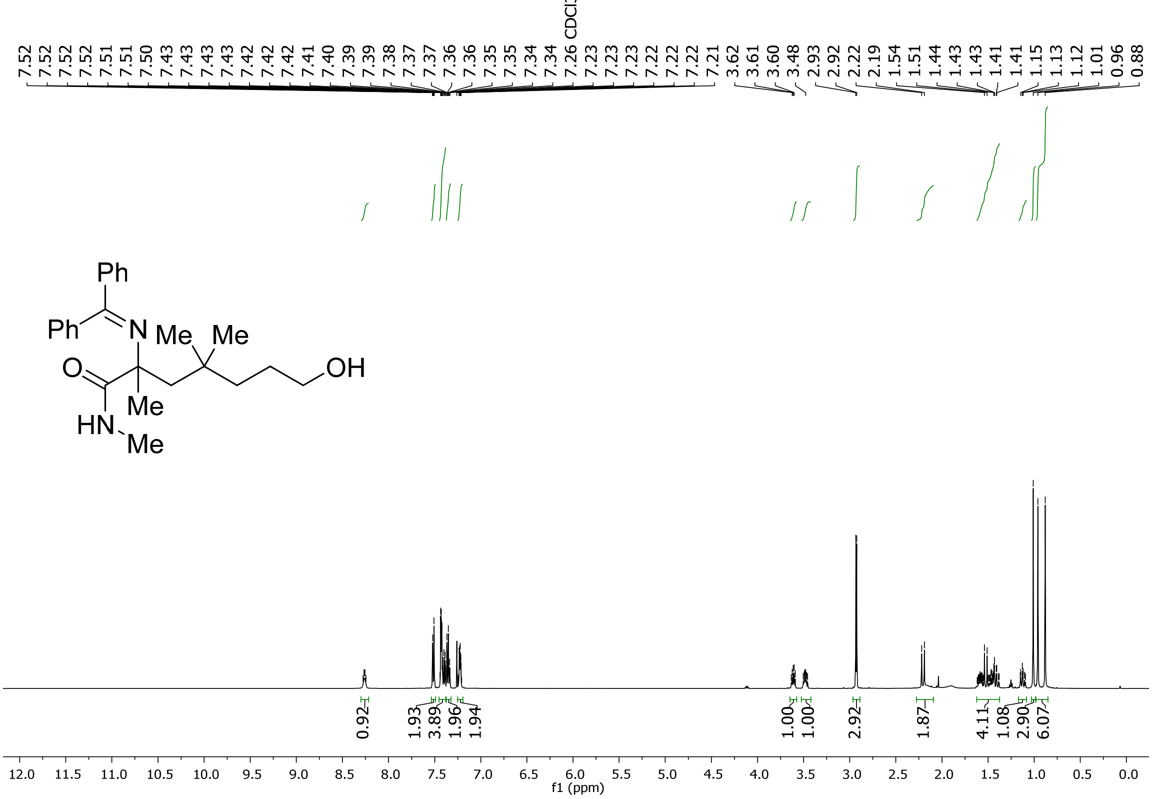

^{1}H NMR (500 MHz, $CDCl_3$) of compound **3h**.

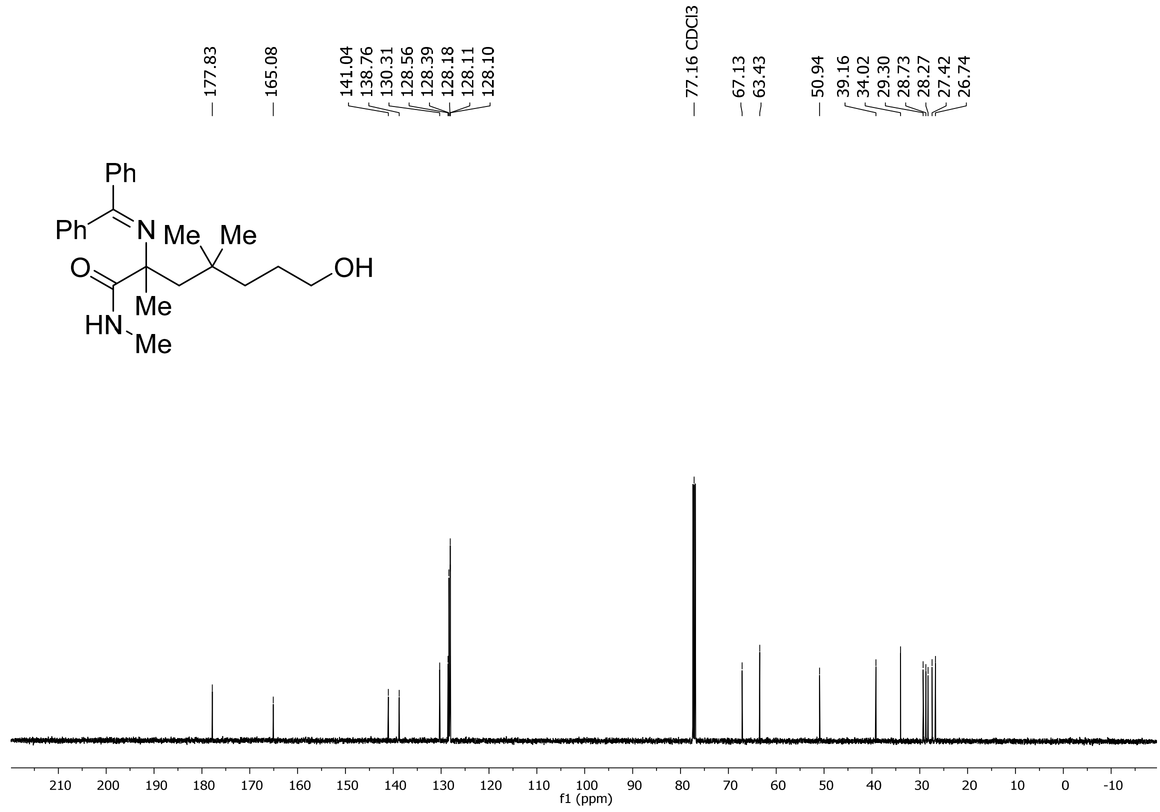

$^{13}C\{^1H\}$ NMR (126 MHz, $CDCl_3$) of compound **3h.**

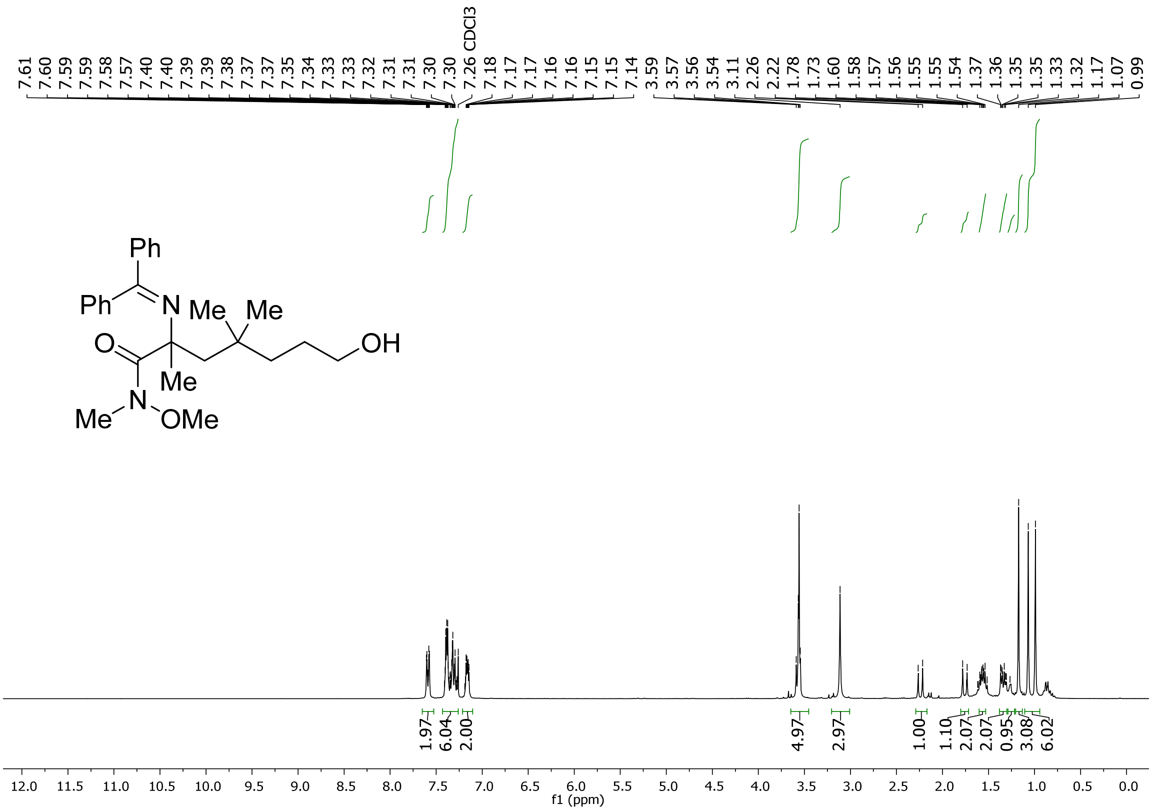

^{1}H NMR (300 MHz, $CDCl_3$) of compound **3i**.

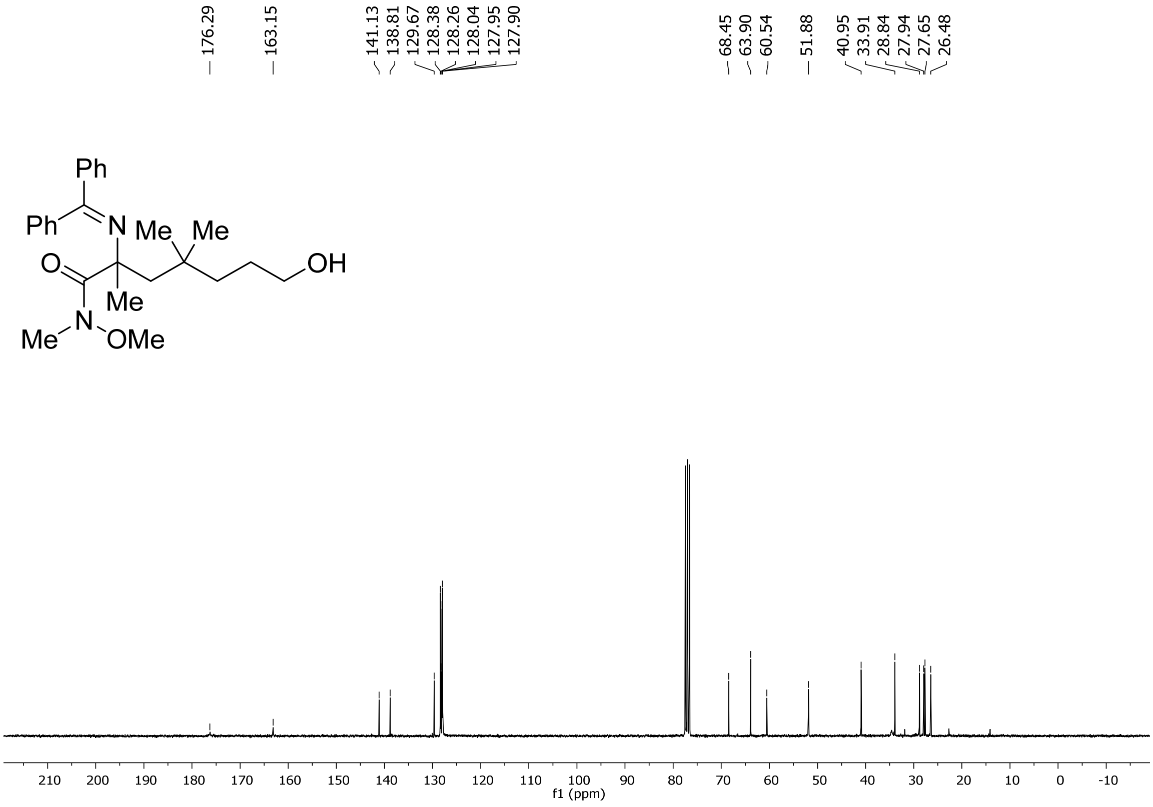

$^{13}C\{^{1}H\}$ NMR (75 MHz, $CDCl_3$) of compound **3i.**

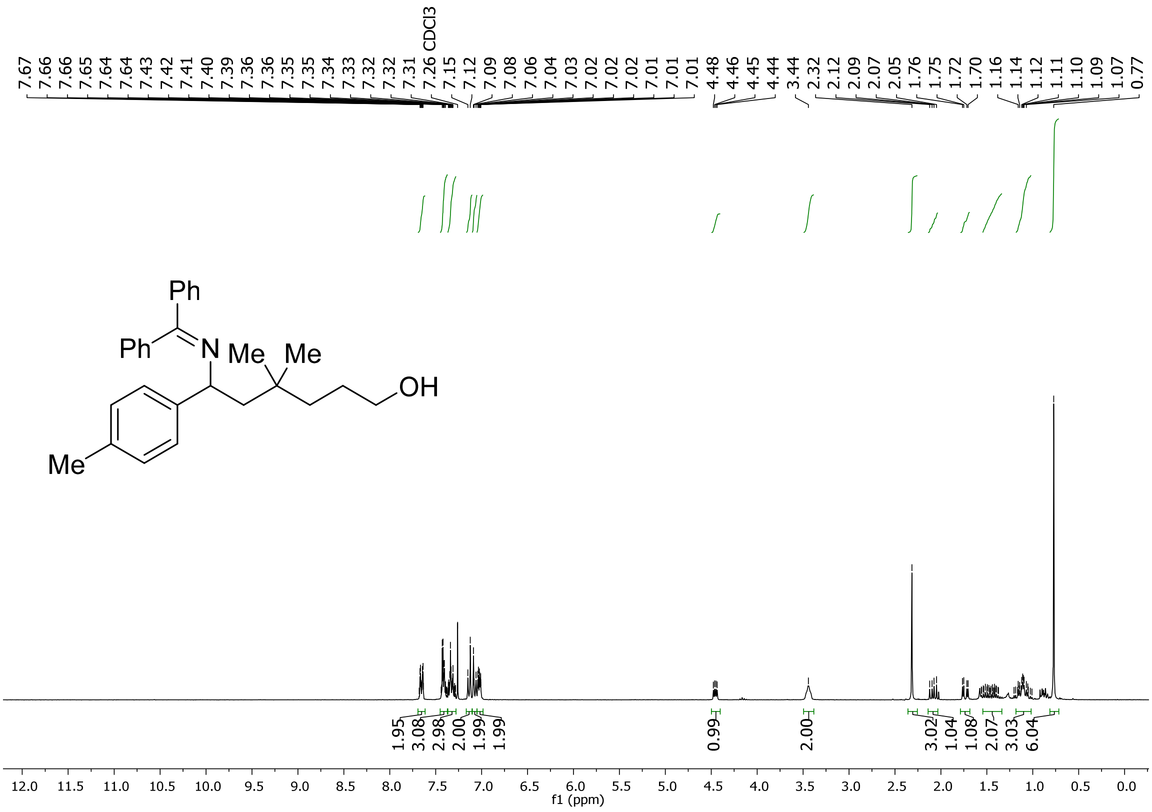

^{1}H NMR (300 MHz, $CDCl_3$) of compound **3j**.

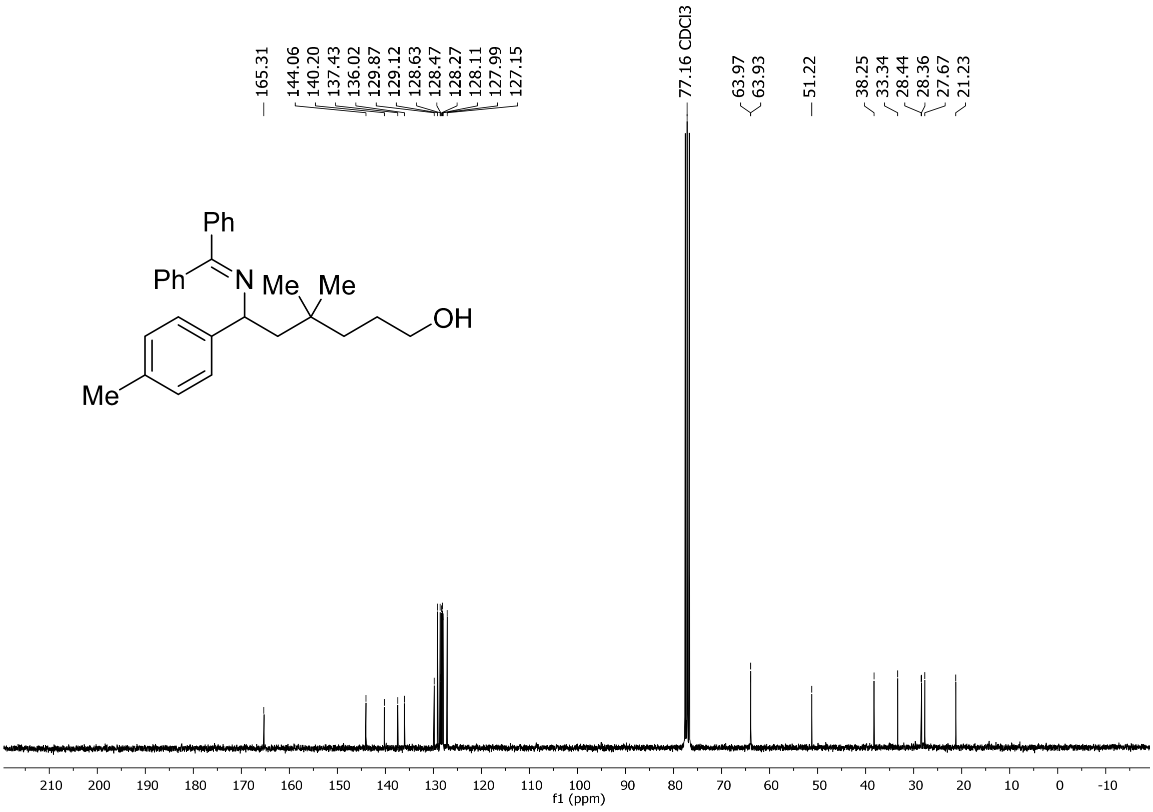

^{13}C{^{1}H} NMR (76 MHz, $CDCl_3$) of compound **3j.**

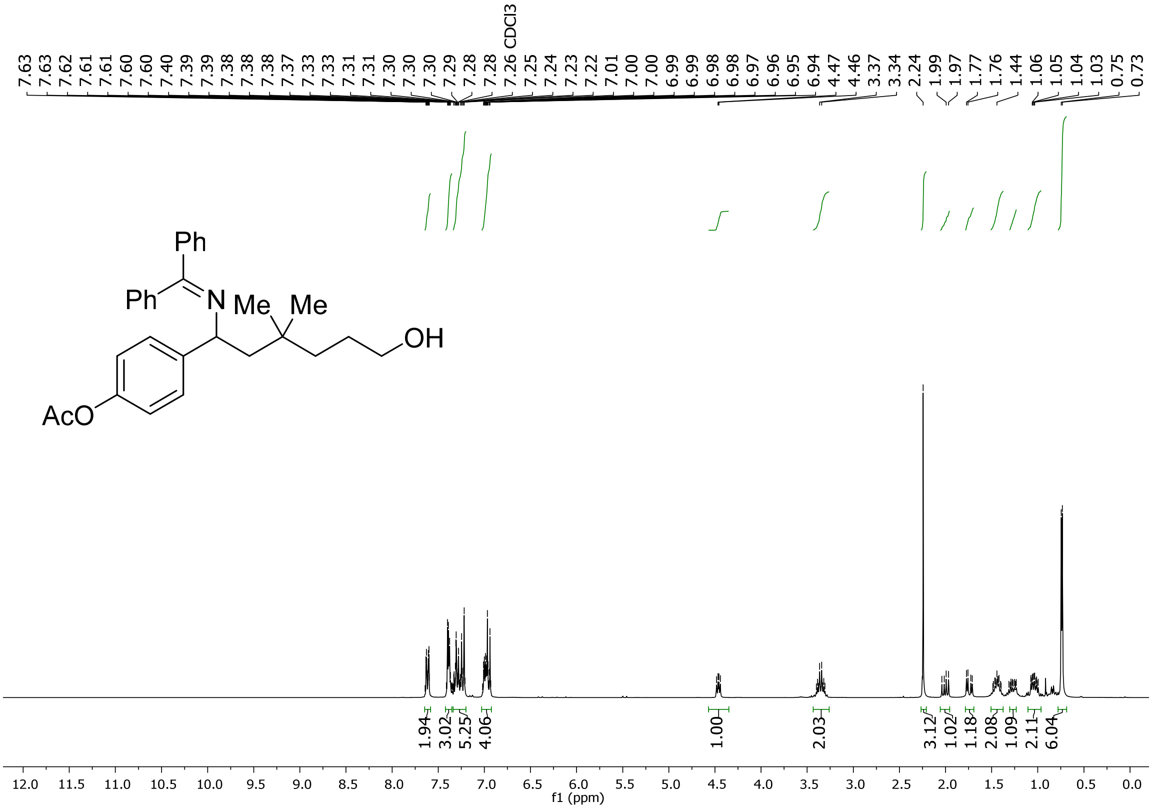

^{1}H NMR (300 MHz, $CDCl_3$) of compound **3k**.

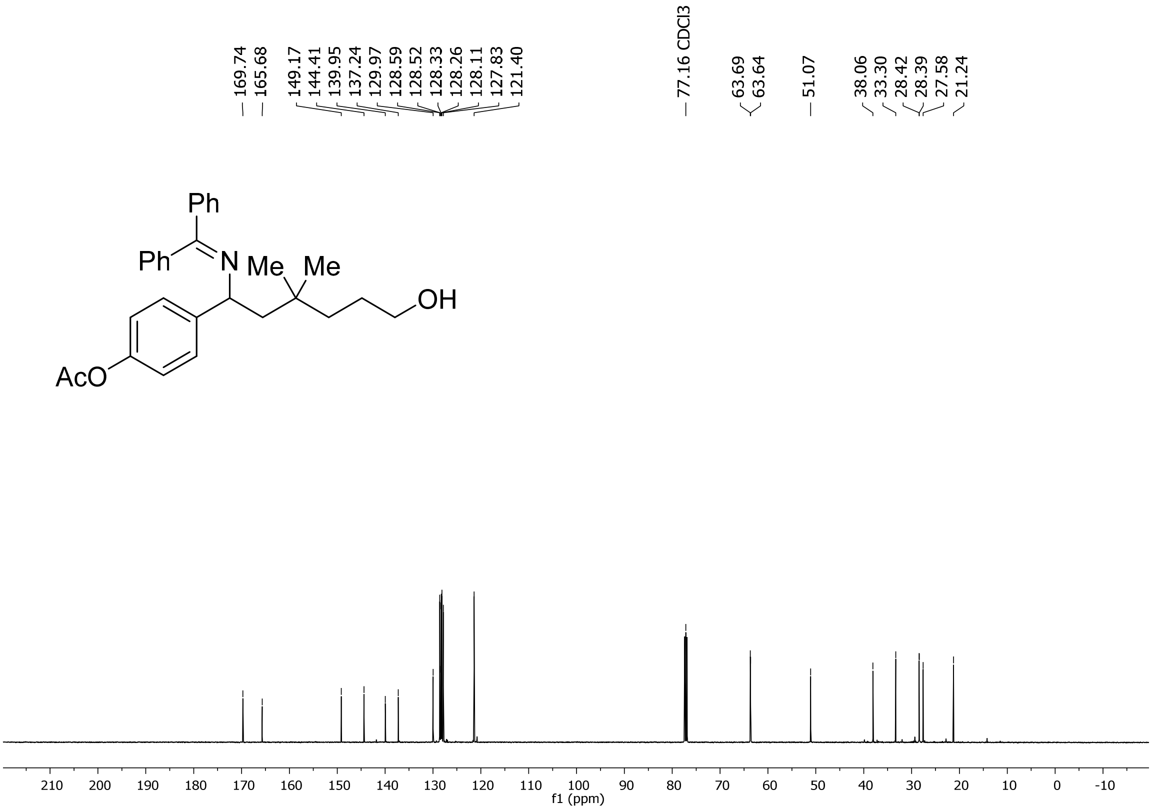

^{13}C{^{1}H} NMR (126 MHz, $CDCl_3$) of compound **3k.**

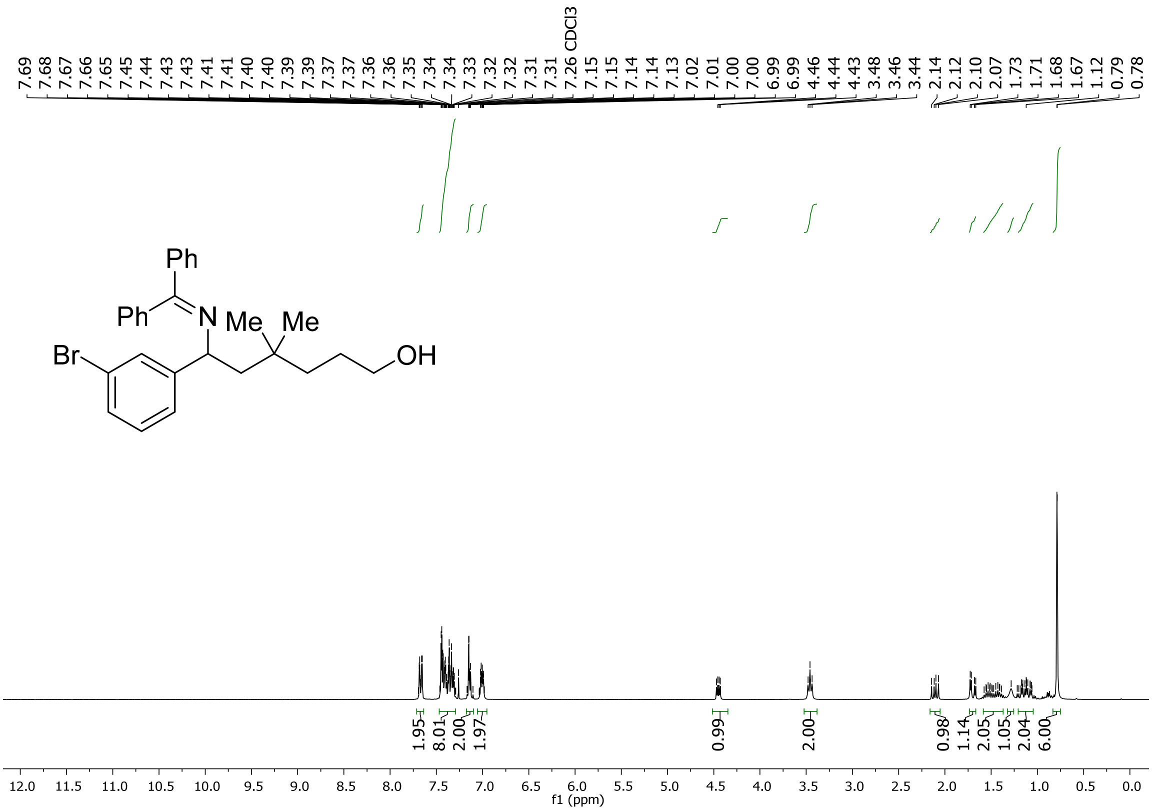

^{1}H NMR (300 MHz, $CDCl_3$) of compound **3l**.

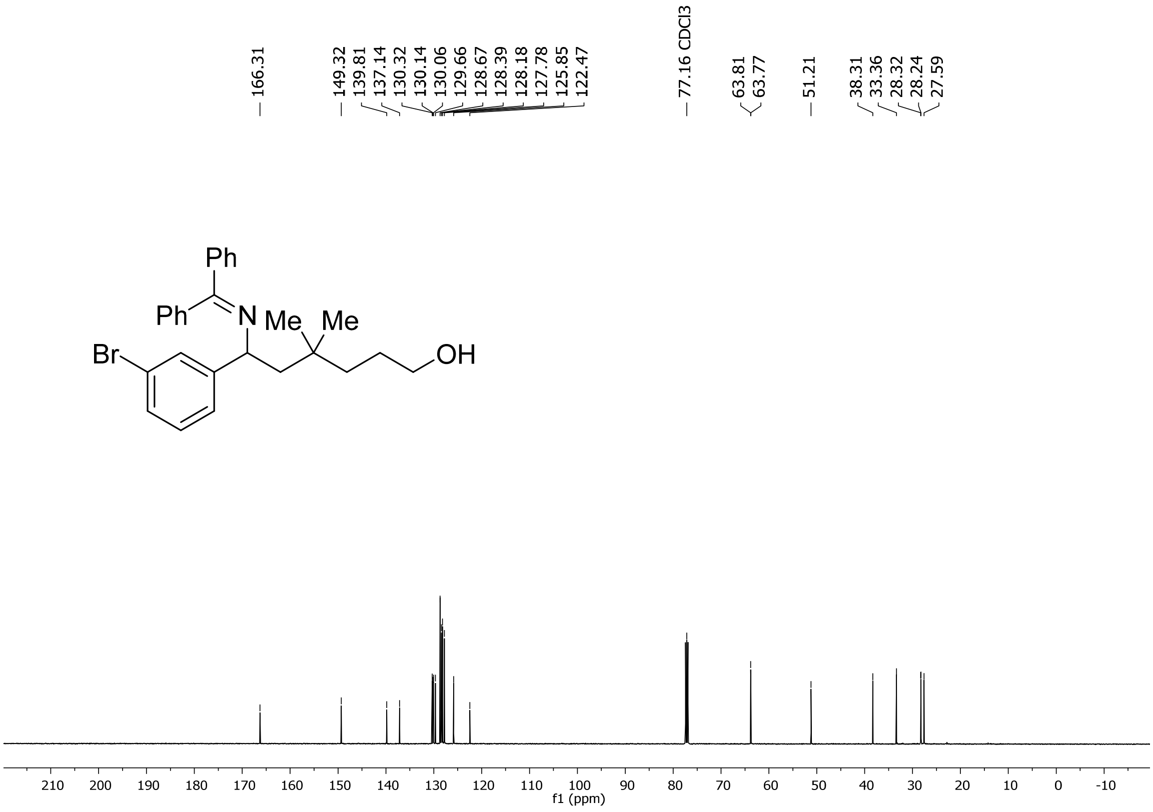

^{13}C{^{1}H} NMR (126 MHz, $CDCl_3$) of compound **3l.**

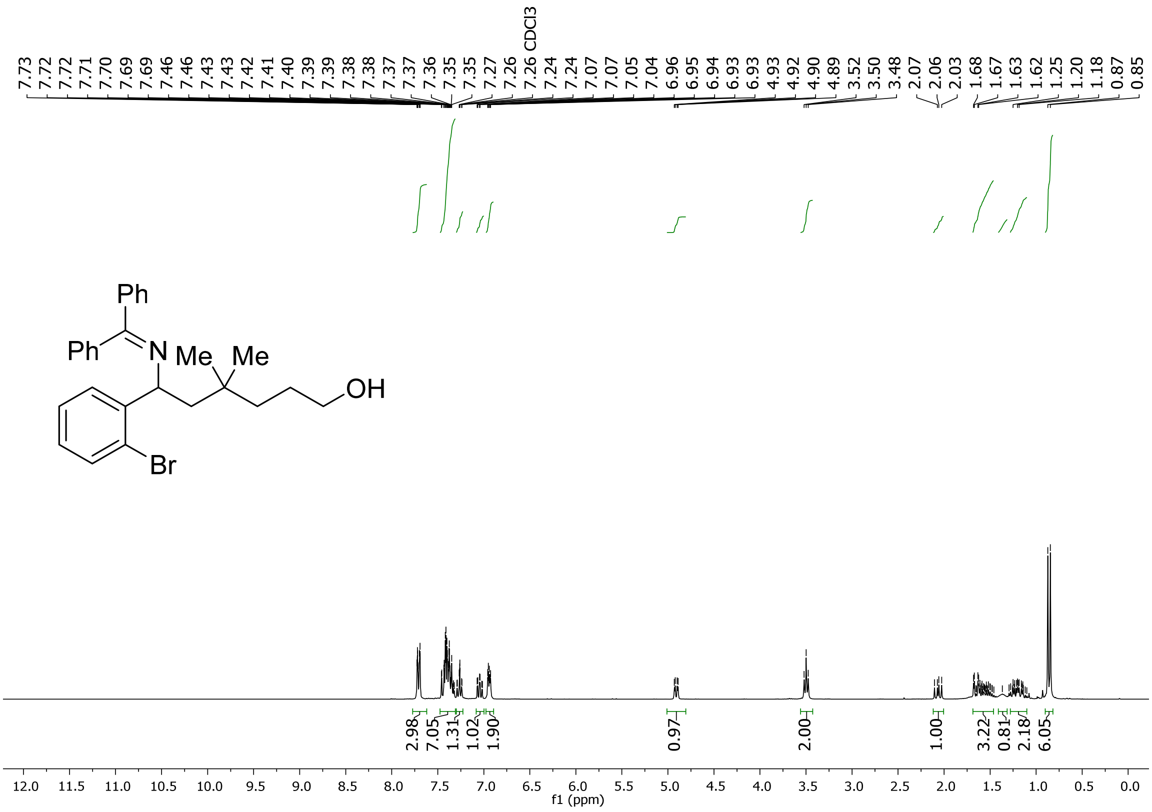

^{1}H NMR (300 MHz, $CDCl_3$) of compound **3m**.

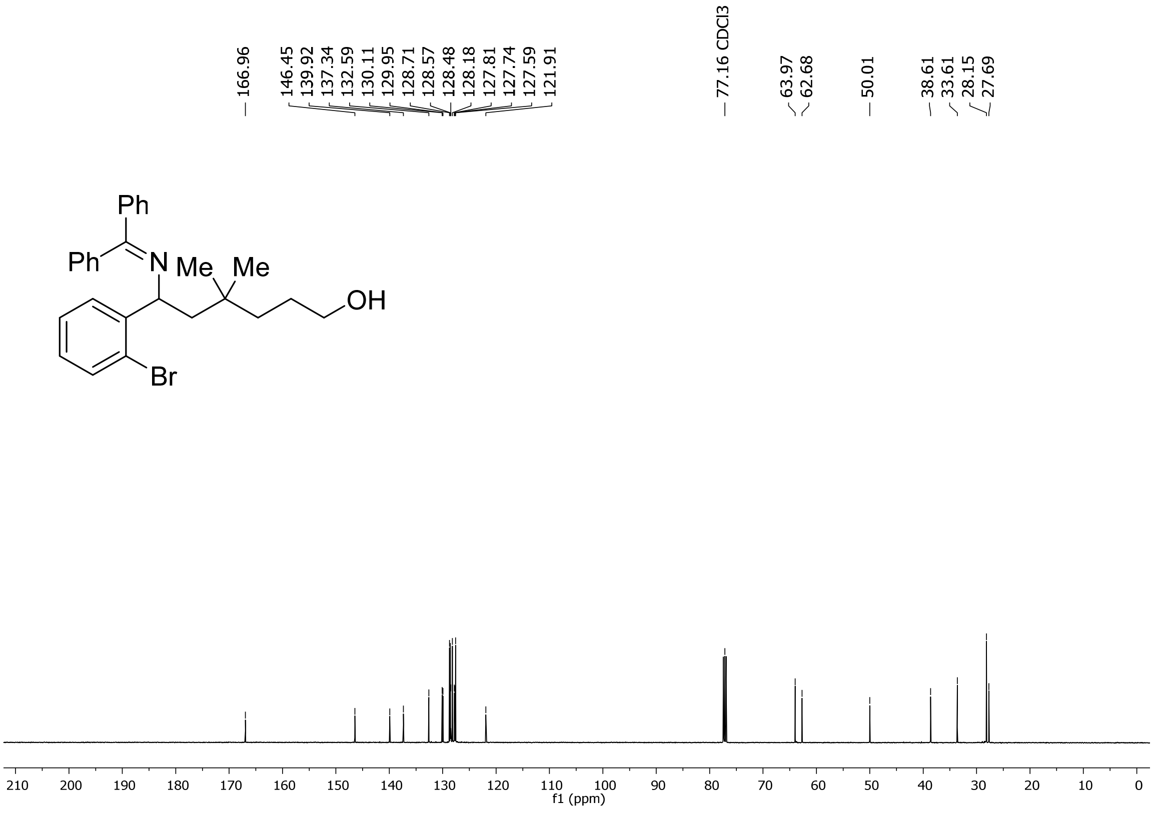

$^{13}C\{^{1}H\}$ NMR (126 MHz, $CDCl_3$) of compound **3m.**

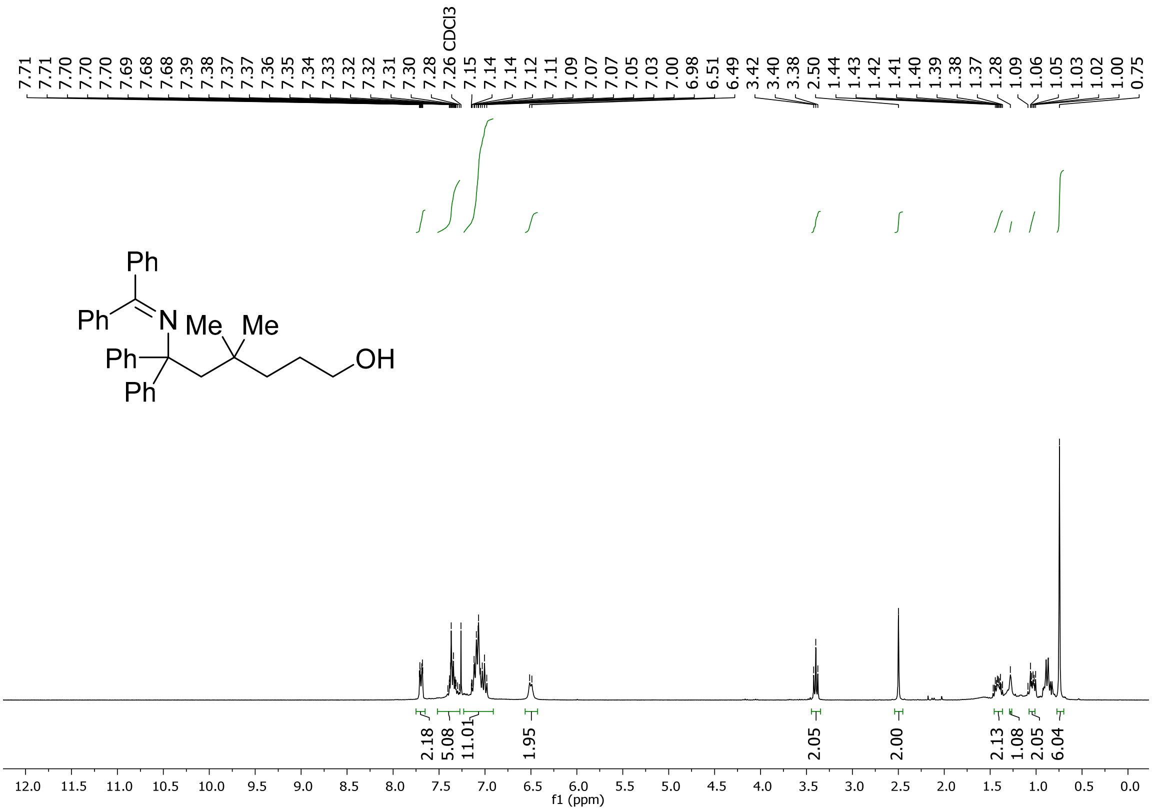

^{1}H NMR (300 MHz, $CDCl_3$) of compound **3n**.

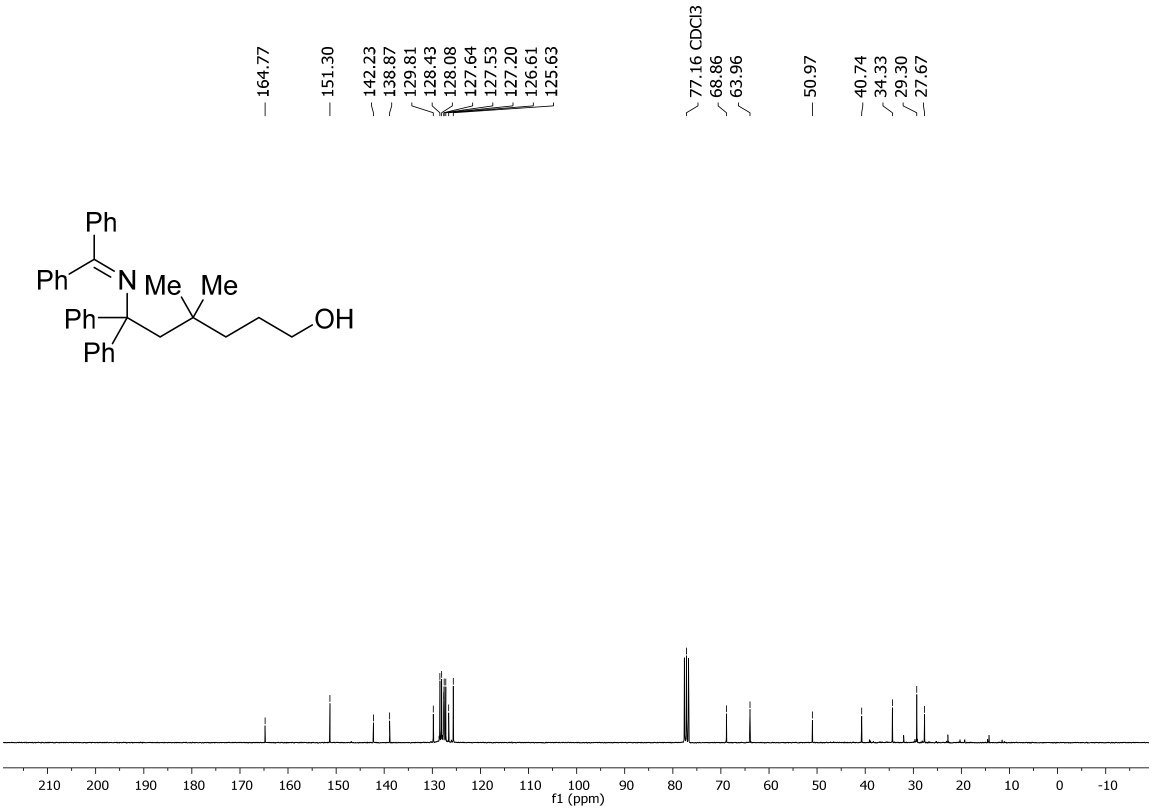

^{13}C{^{1}H} NMR (75 MHz, $CDCl_3$) of compound **3n.**

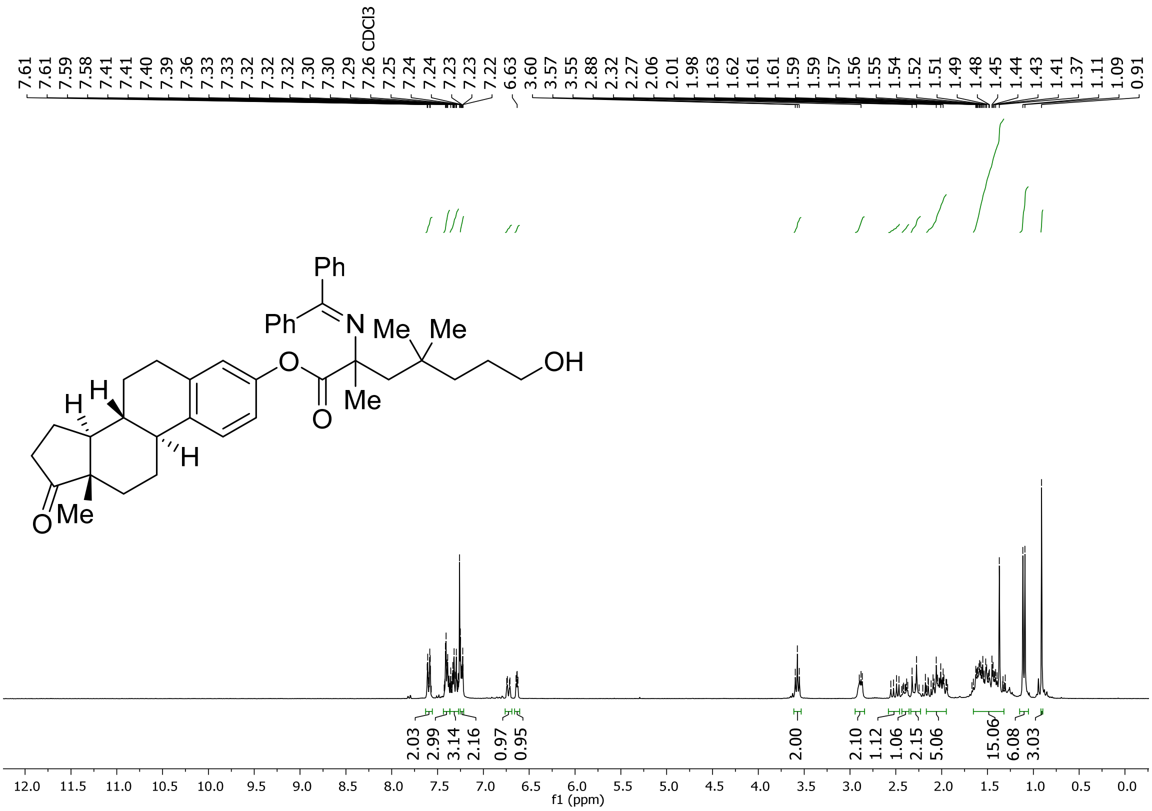

^{1}H NMR (300 MHz, $CDCl_3$) of compound **3o**.

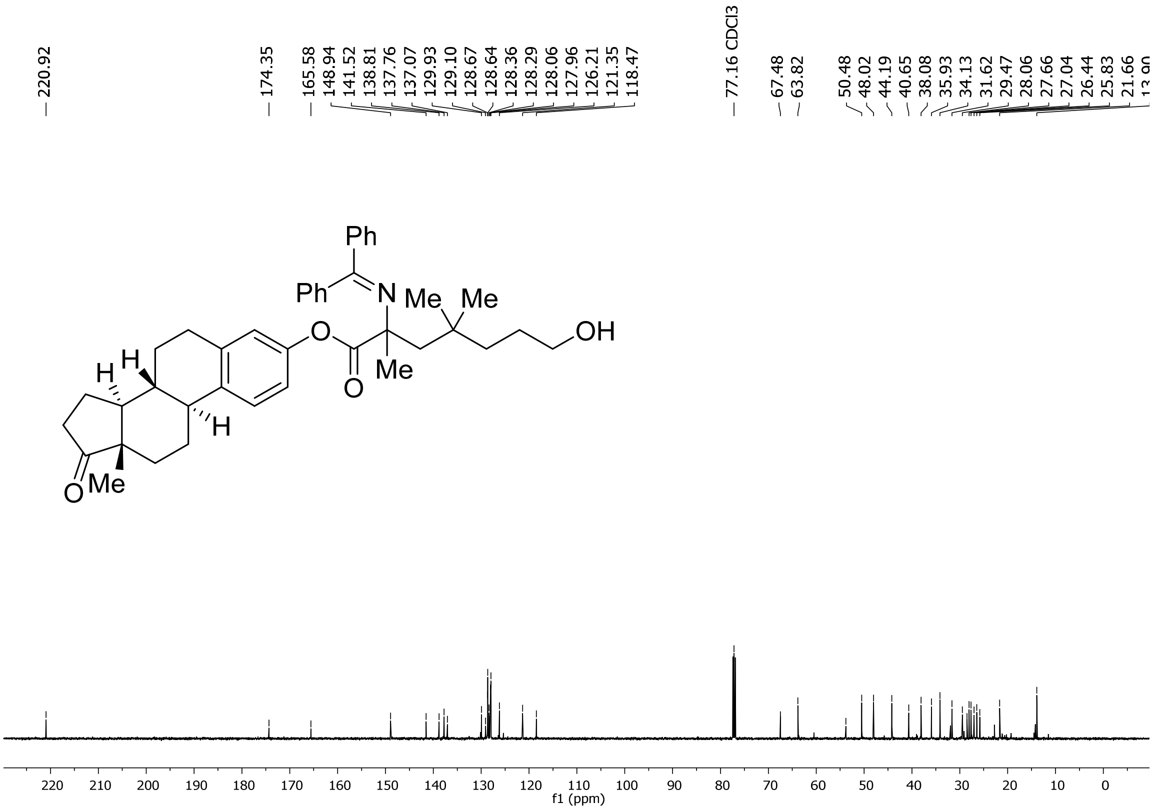

$^{13}C\{^{1}H\}$ NMR (126 MHz, $CDCl_3$) of compound **3o.**

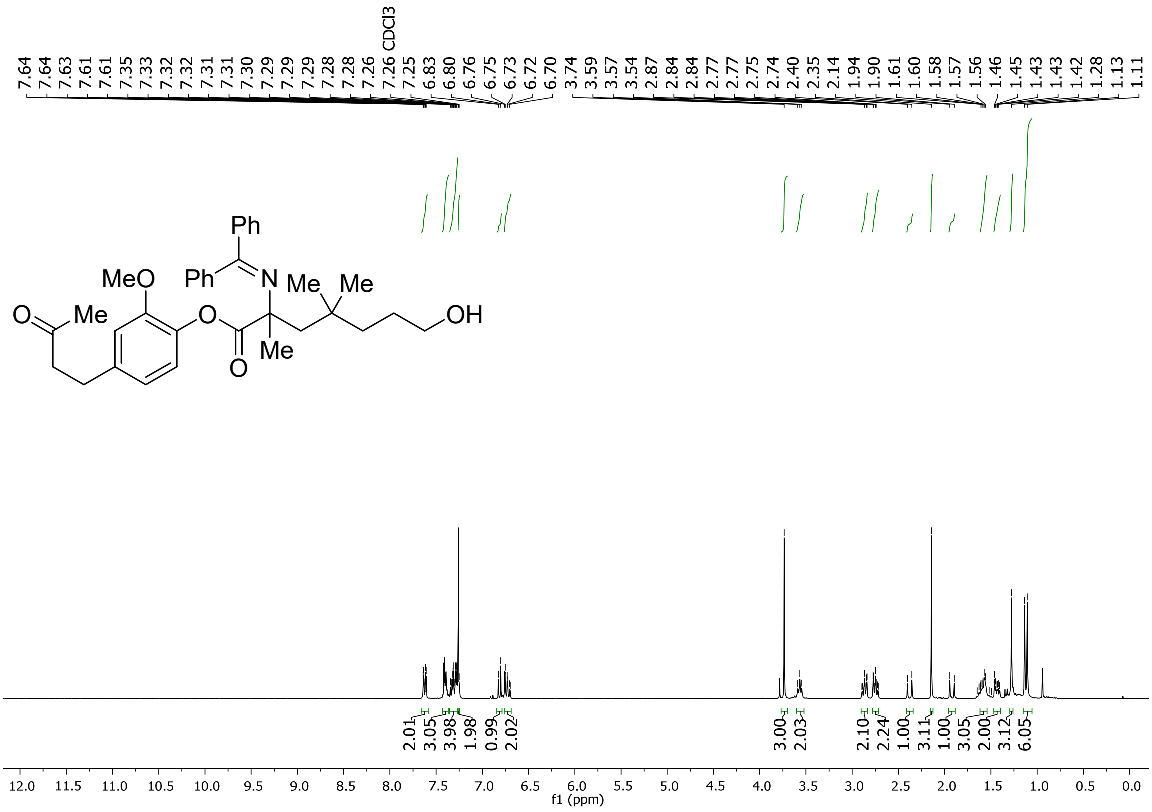

^{1}H NMR (300 MHz, $CDCl_3$) of compound **3p**.

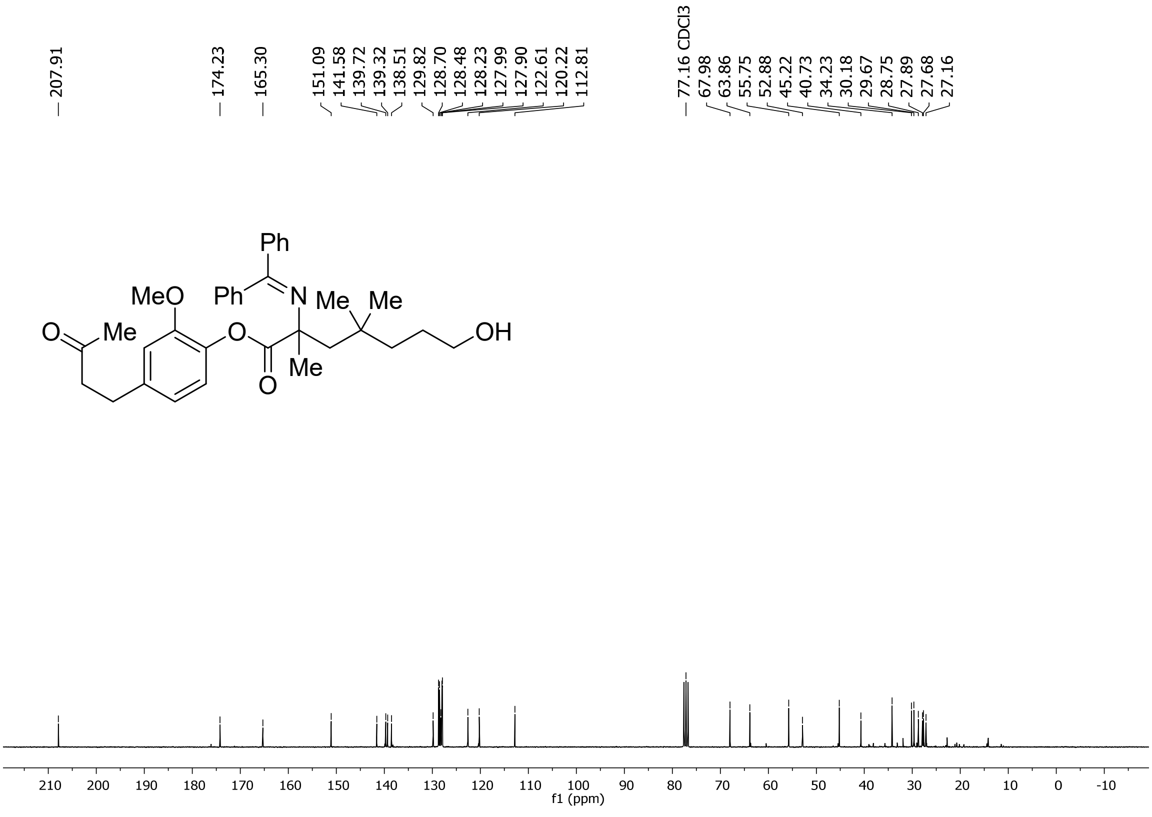

$^{13}C\{^1H\}$ NMR (76 MHz, $CDCl_3$) of compound **3p.**

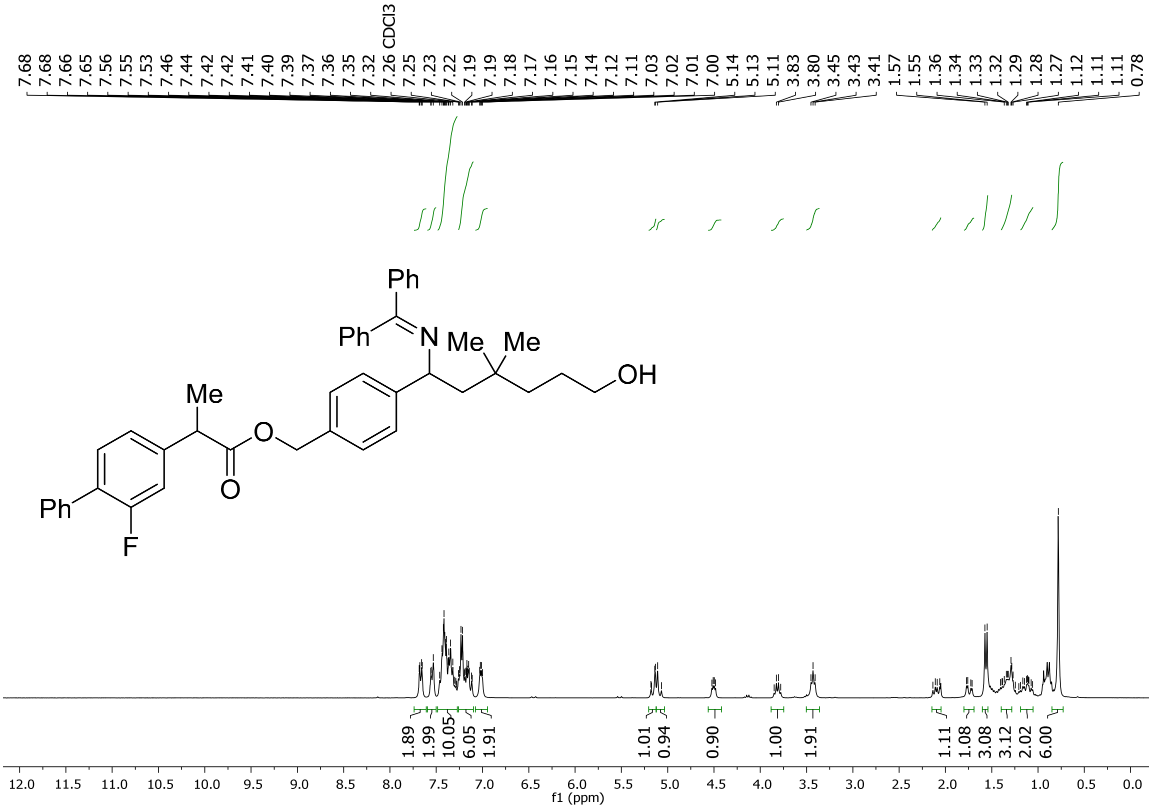

^{1}H NMR (300 MHz, $CDCl_3$) of compound **3q**.

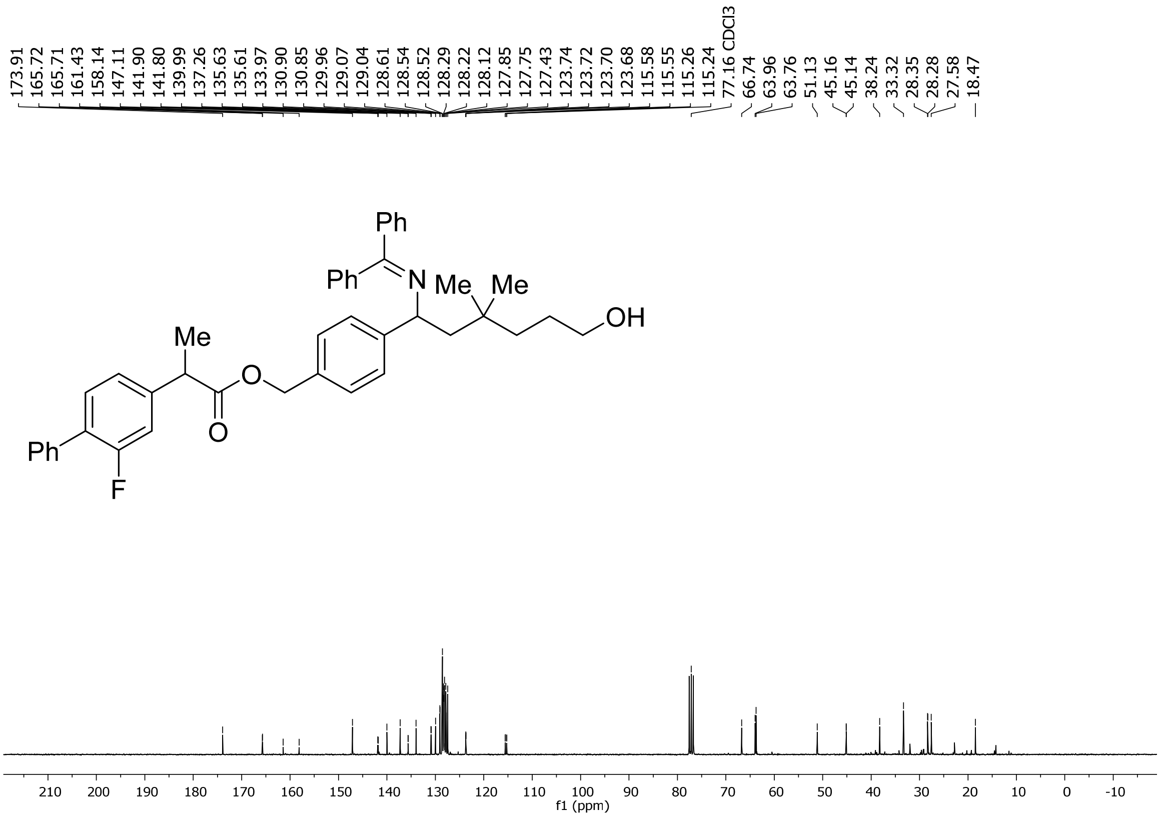

$^{13}C\{^{1}H\}$ NMR (75 MHz, $CDCl_3$) of compound **3q.**

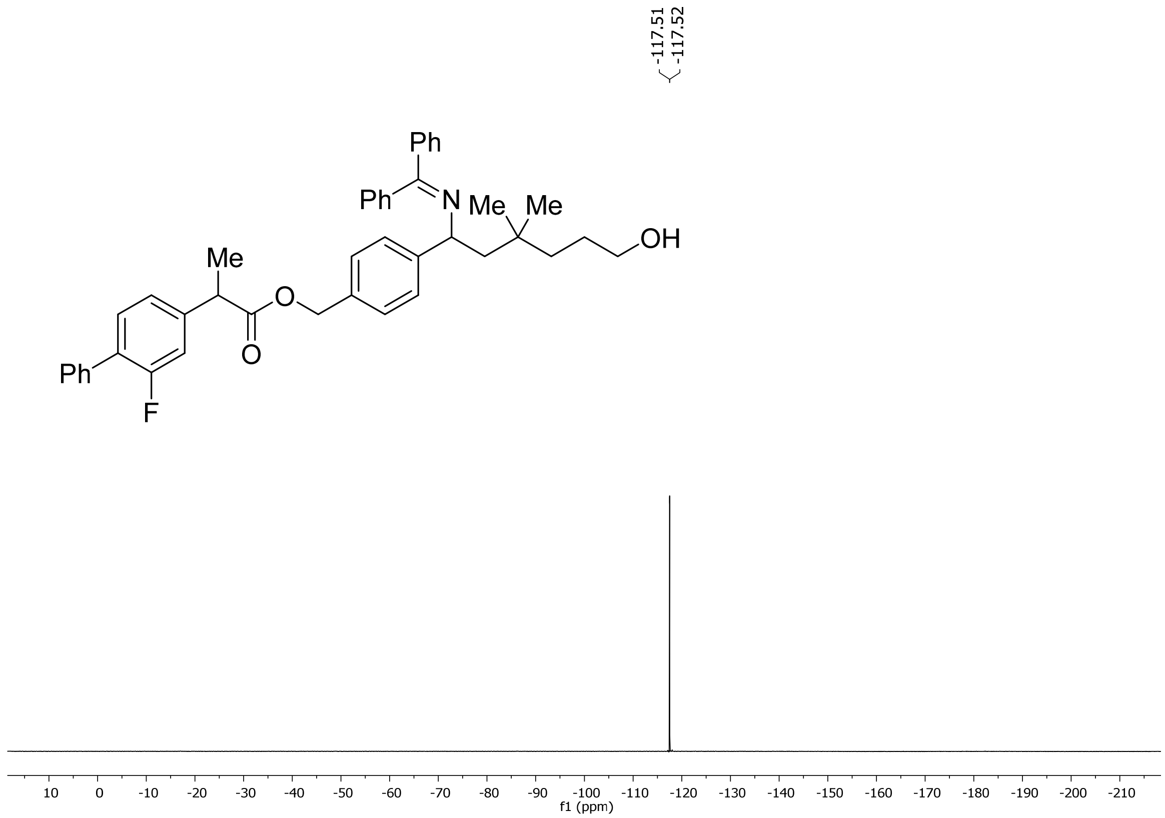

$^{19}F\{^{1}H\}$ NMR (282 MHz, $CDCl_3$) of compound **3q.**

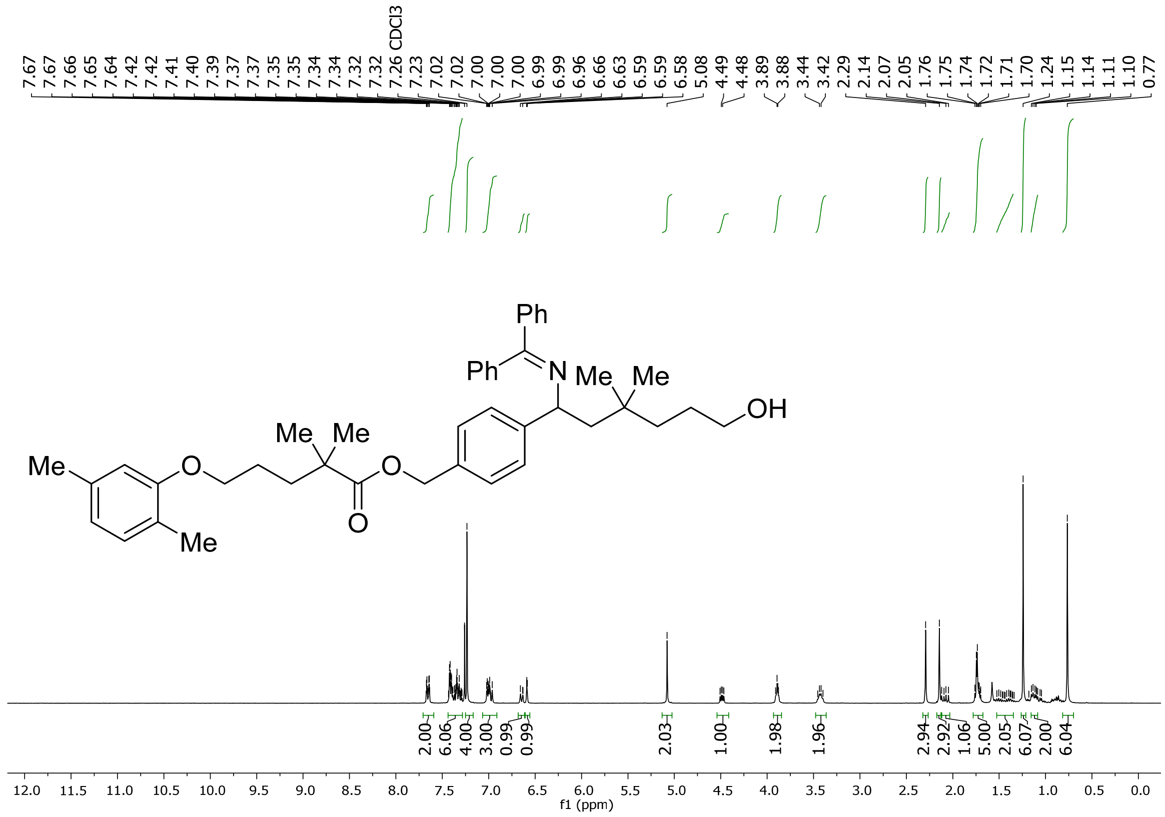

^{1}H NMR (300 MHz, $CDCl_3$) of compound **3r**.

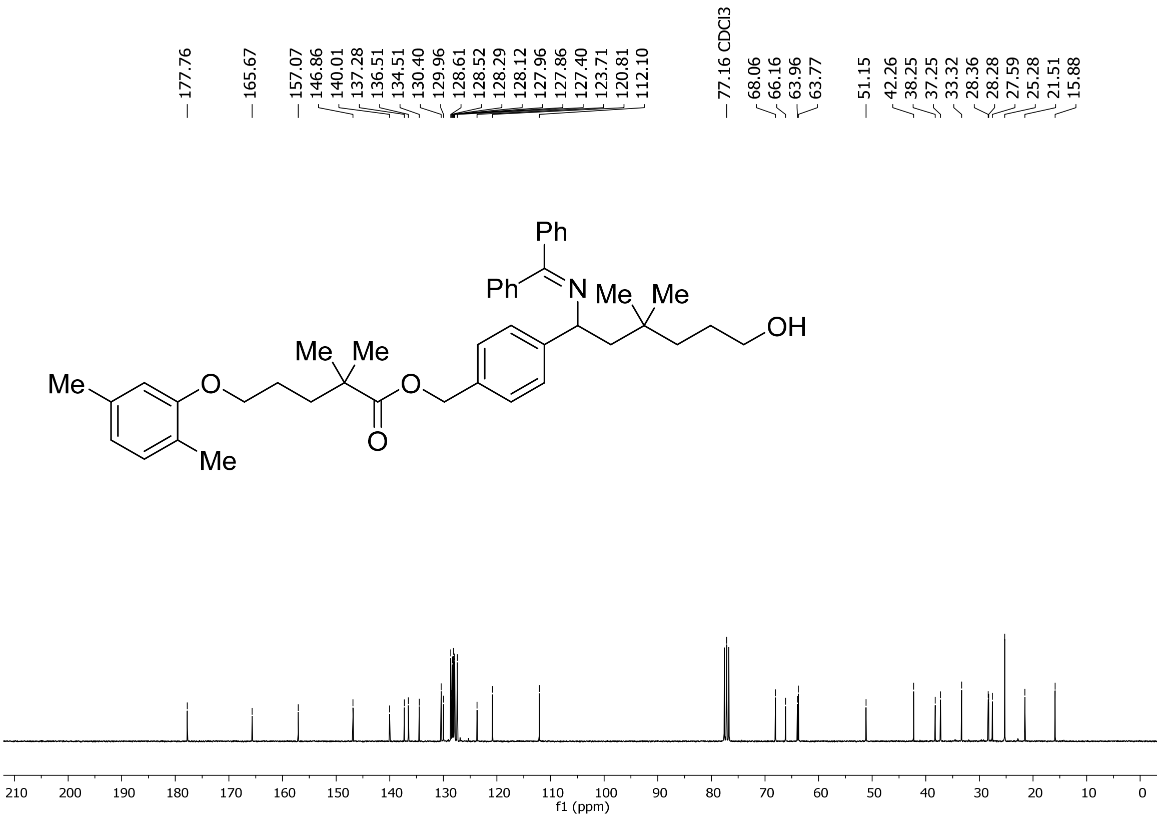

$^{13}C\{^{1}H\}$ NMR (75 MHz, $CDCl_3$) of compound **3r.**

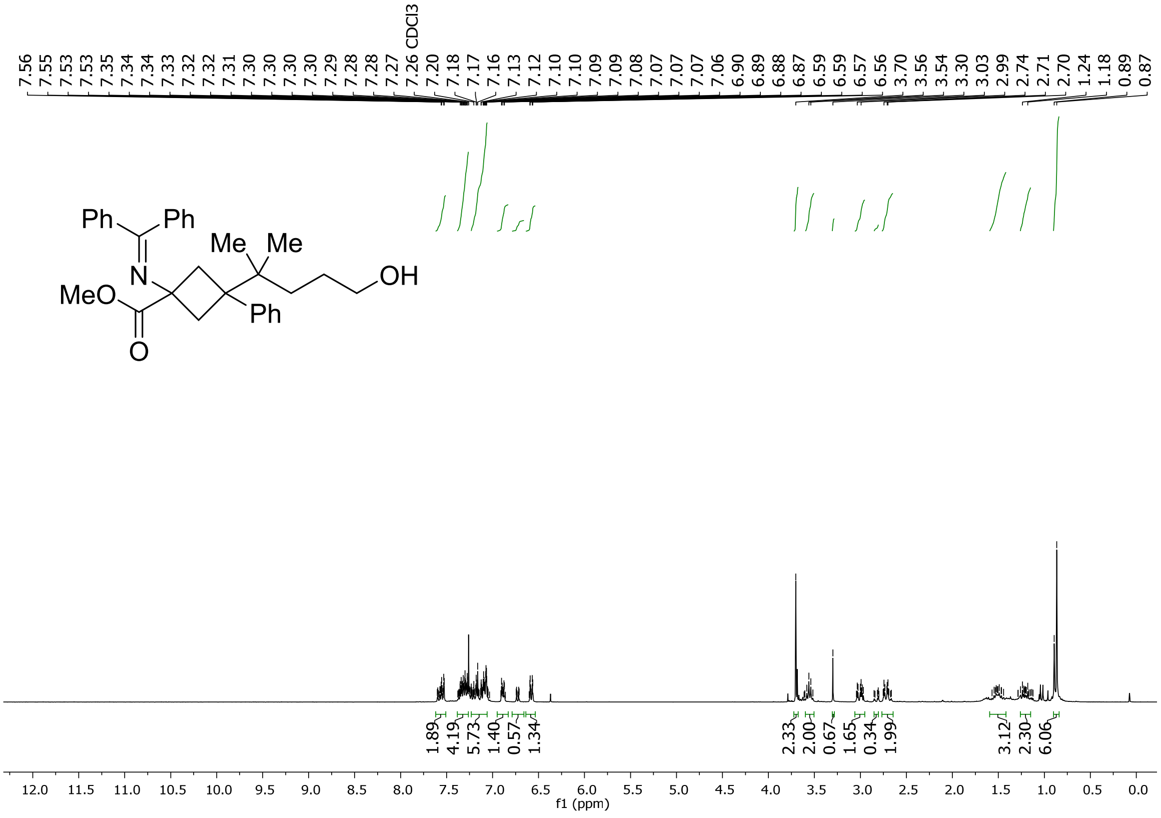

^{1}H NMR (300 MHz, $CDCl_3$) of compound **3s**.

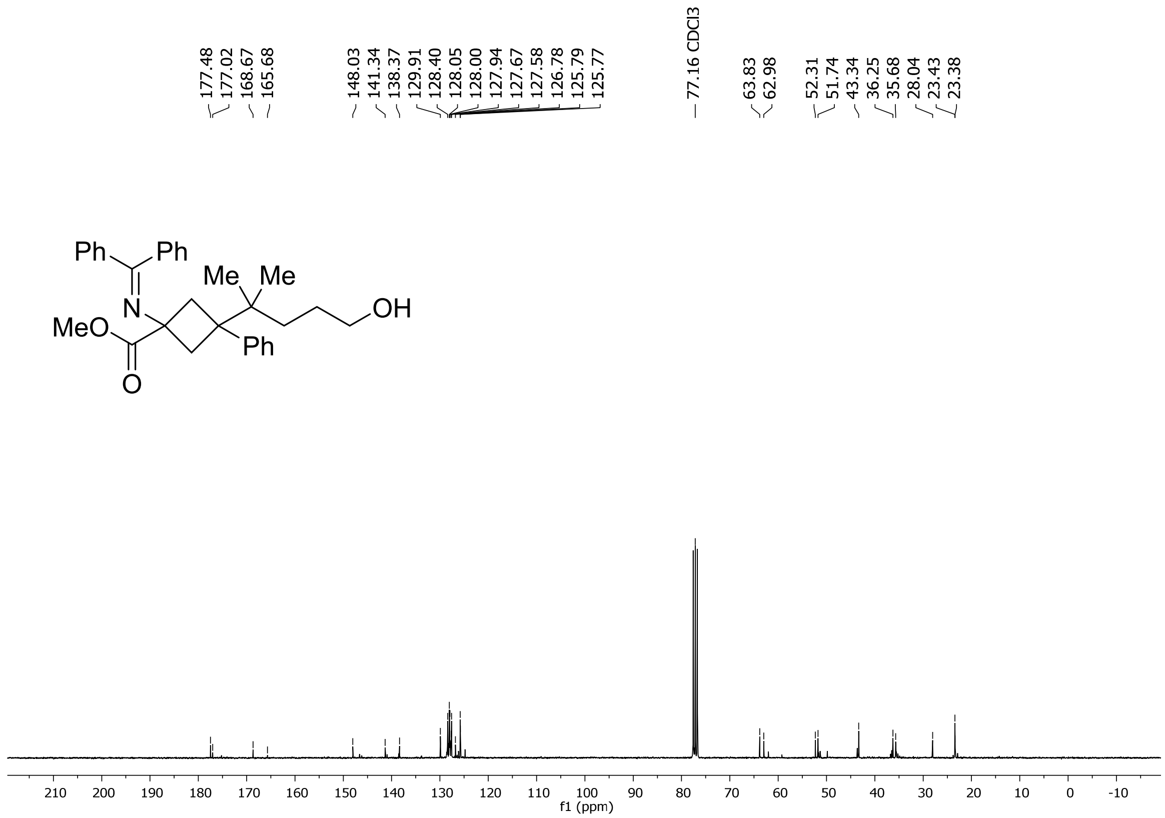

$^{13}C\{^{1}H\}$ NMR (76 MHz, $CDCl_3$) of compound **3s.**

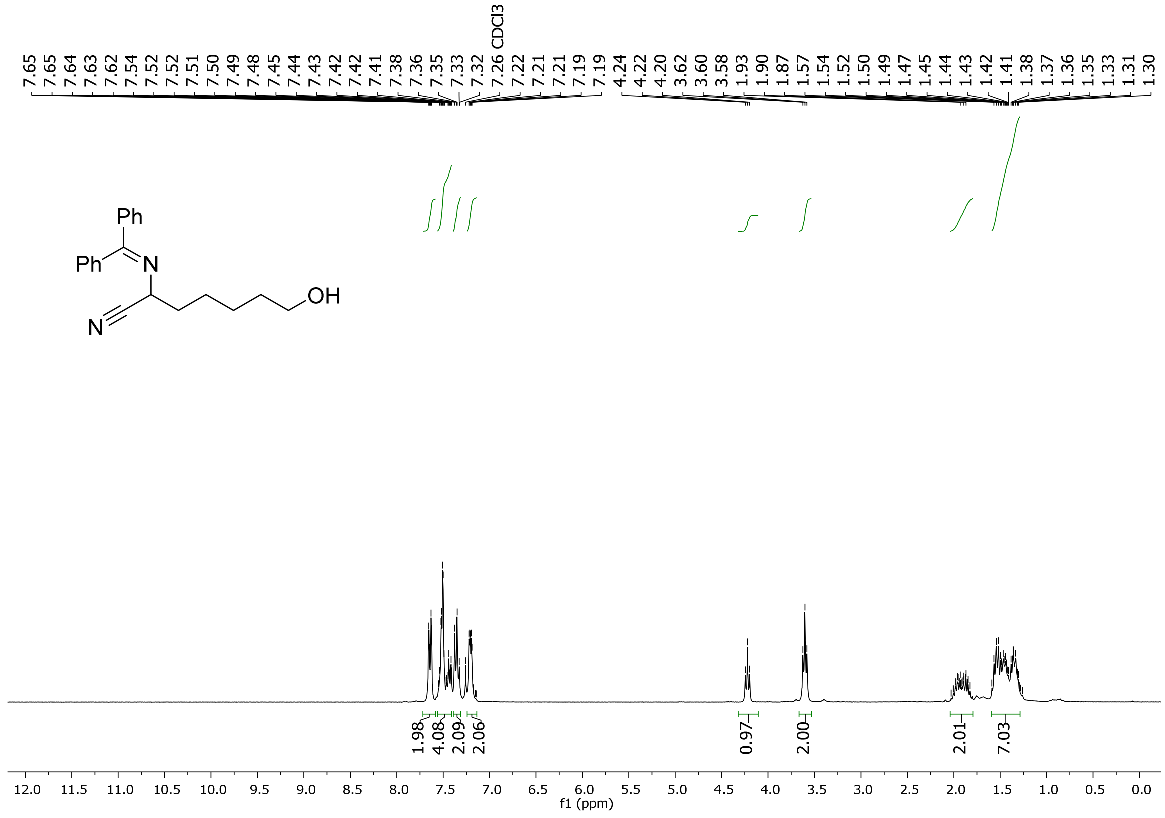

^{1}H NMR (300 MHz, $CDCl_3$) of compound **3t**.

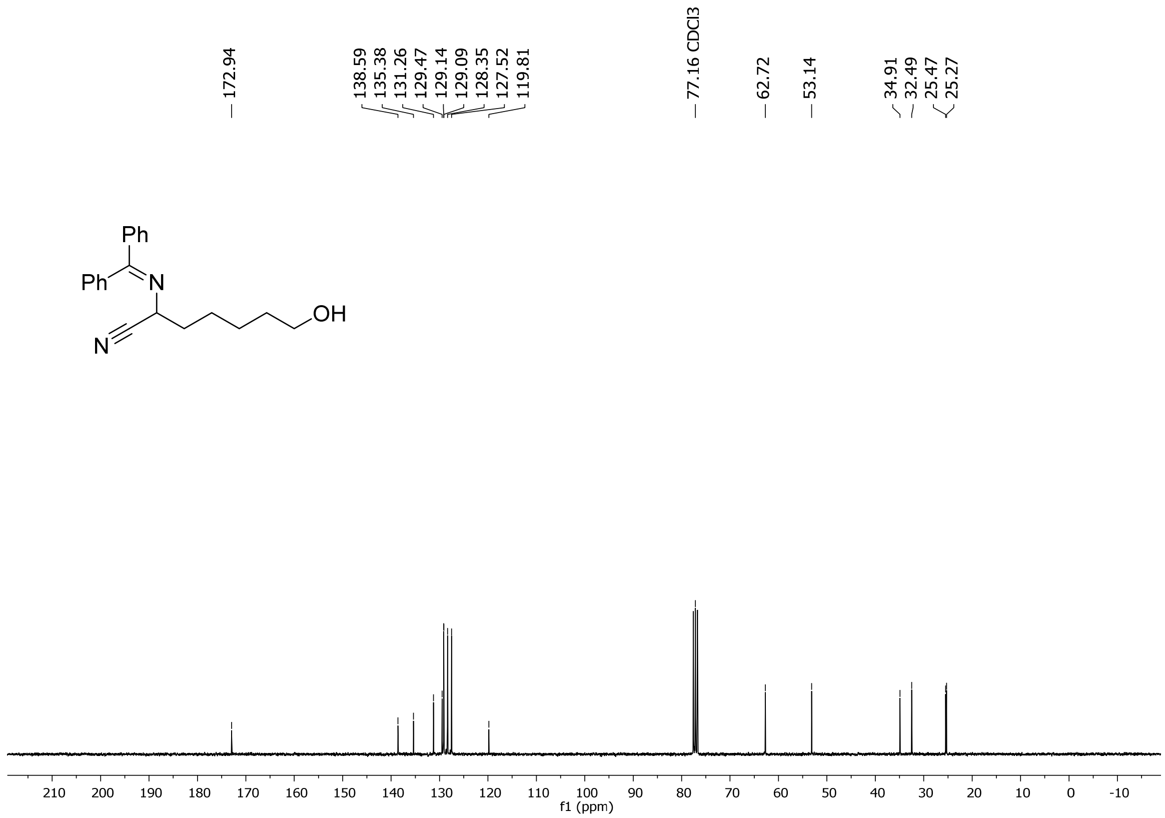

$^{13}C\{^{1}H\}$ NMR (75 MHz, $CDCl_3$) of compound **3t.**

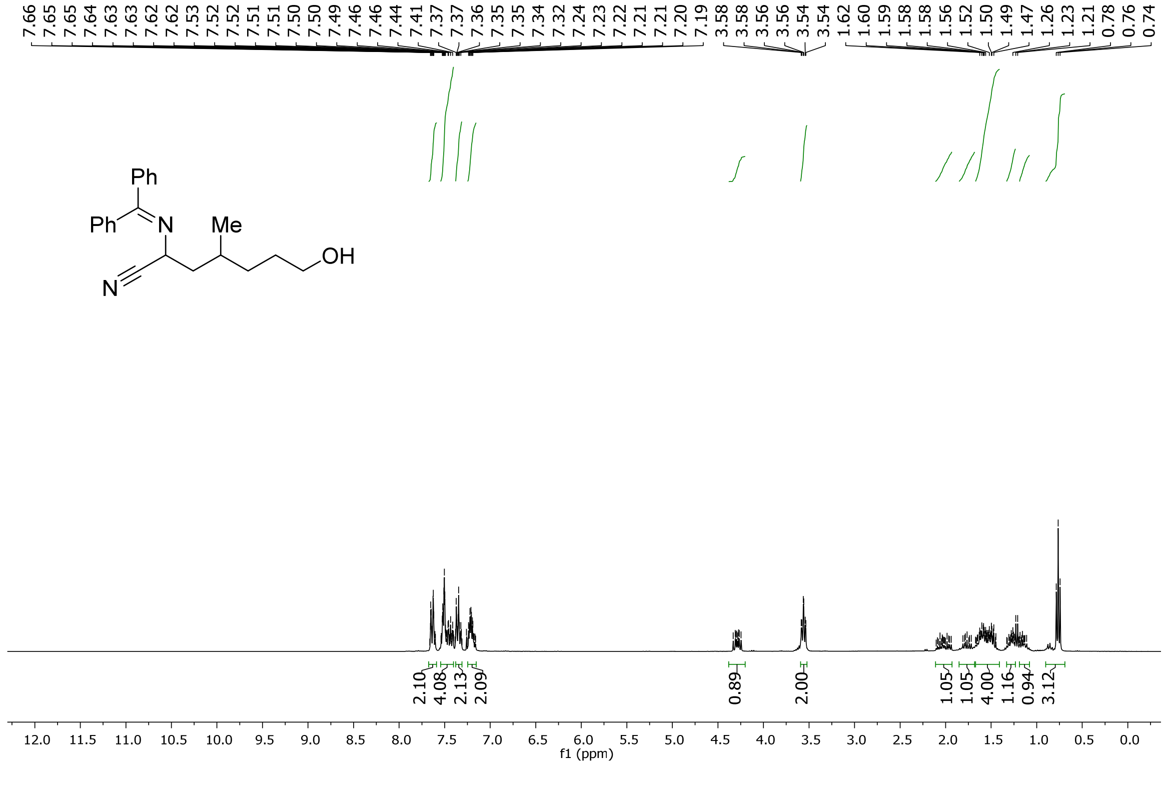

^{1}H NMR (300 MHz, $CDCl_3$) of compound **3u**.

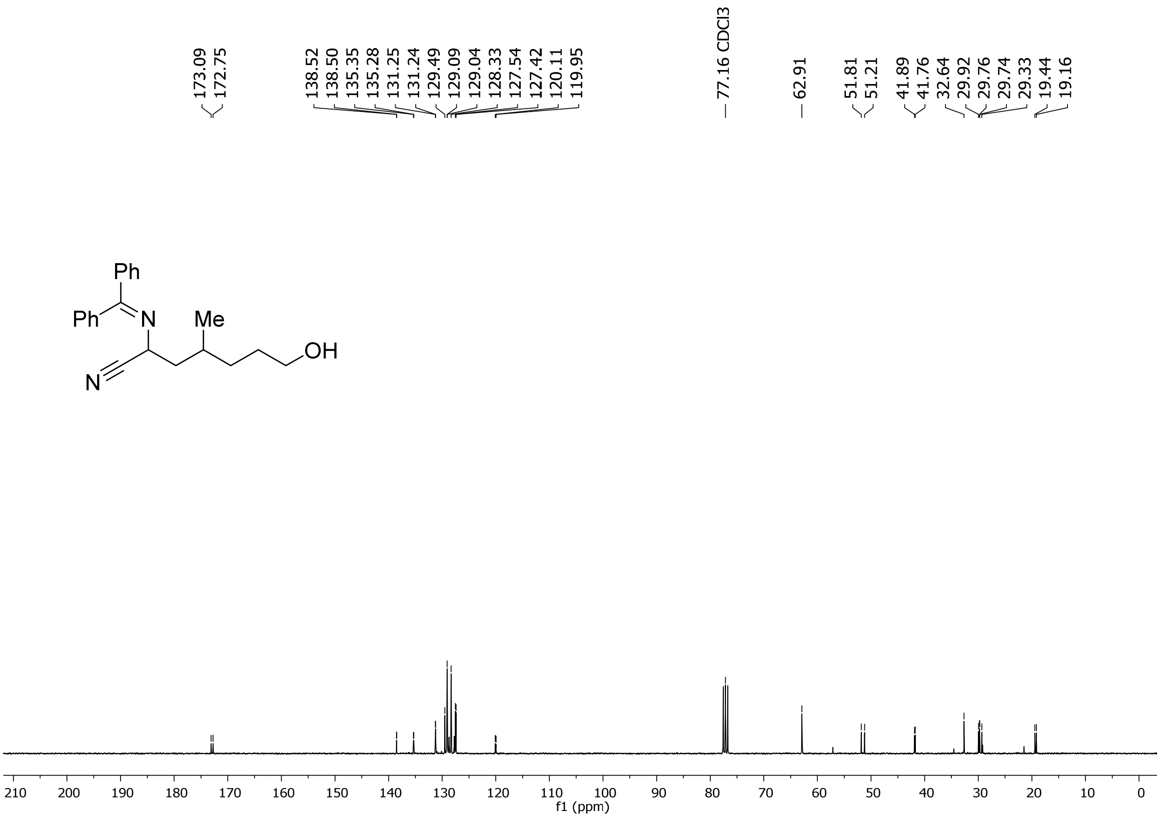

^{13}C{^{1}H} NMR (75 MHz, $CDCl_3$) of compound **3u.**

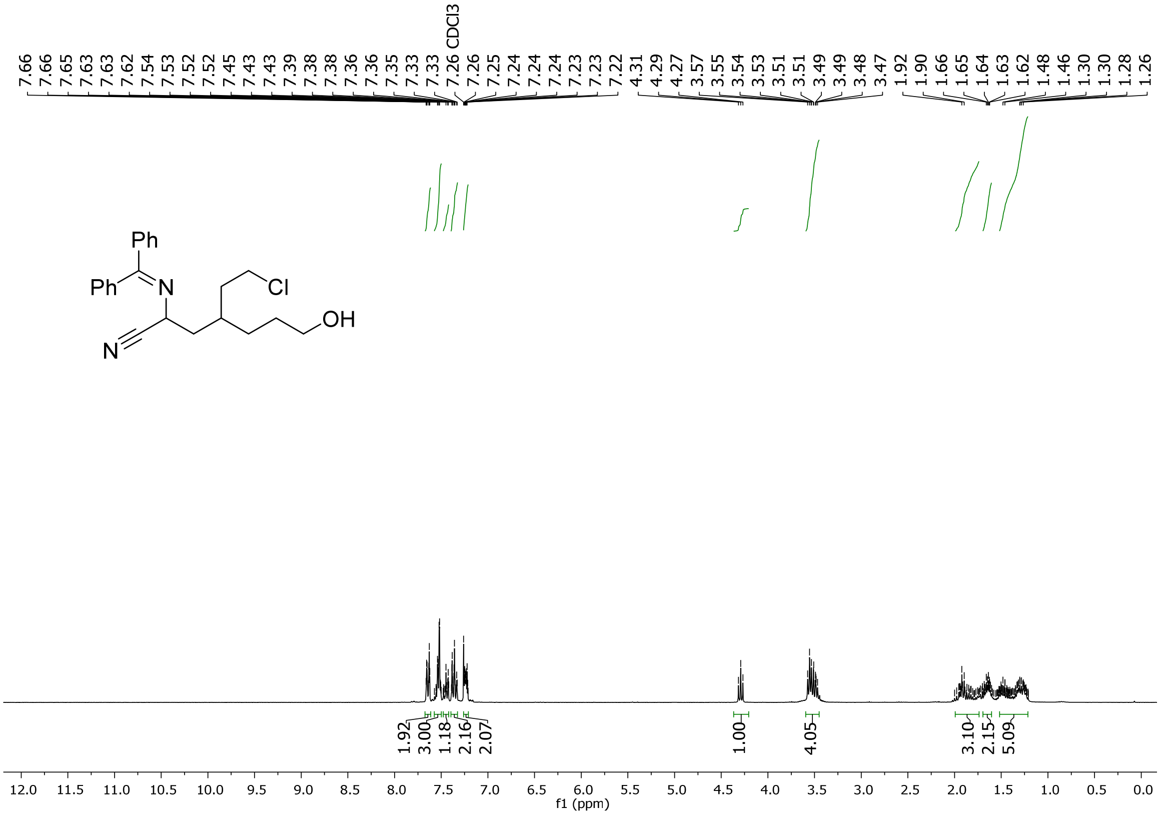

^{1}H NMR (300 MHz, $CDCl_3$) of compound **3v**.

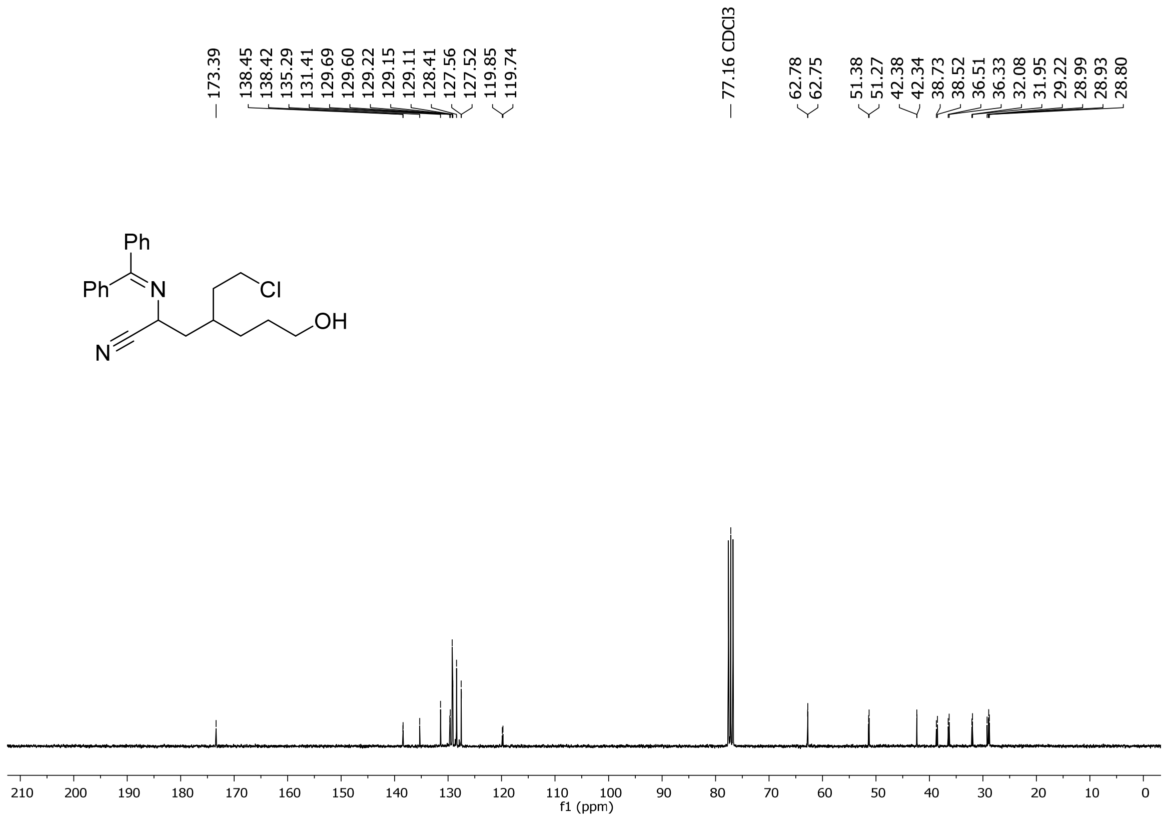

$^{13}C\{^{1}H\}$ NMR (75 MHz, $CDCl_3$) of compound **3v.**

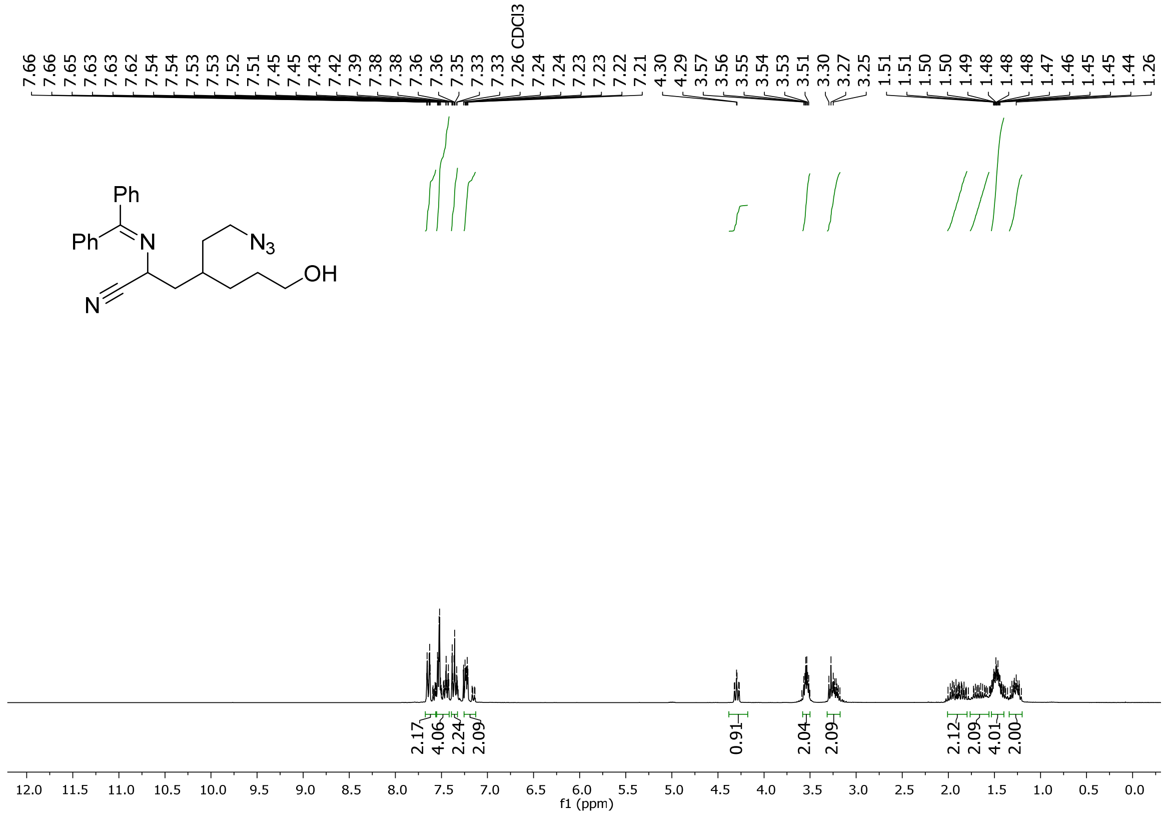

^{1}H NMR (300 MHz, $CDCl_3$) of compound **3w**.

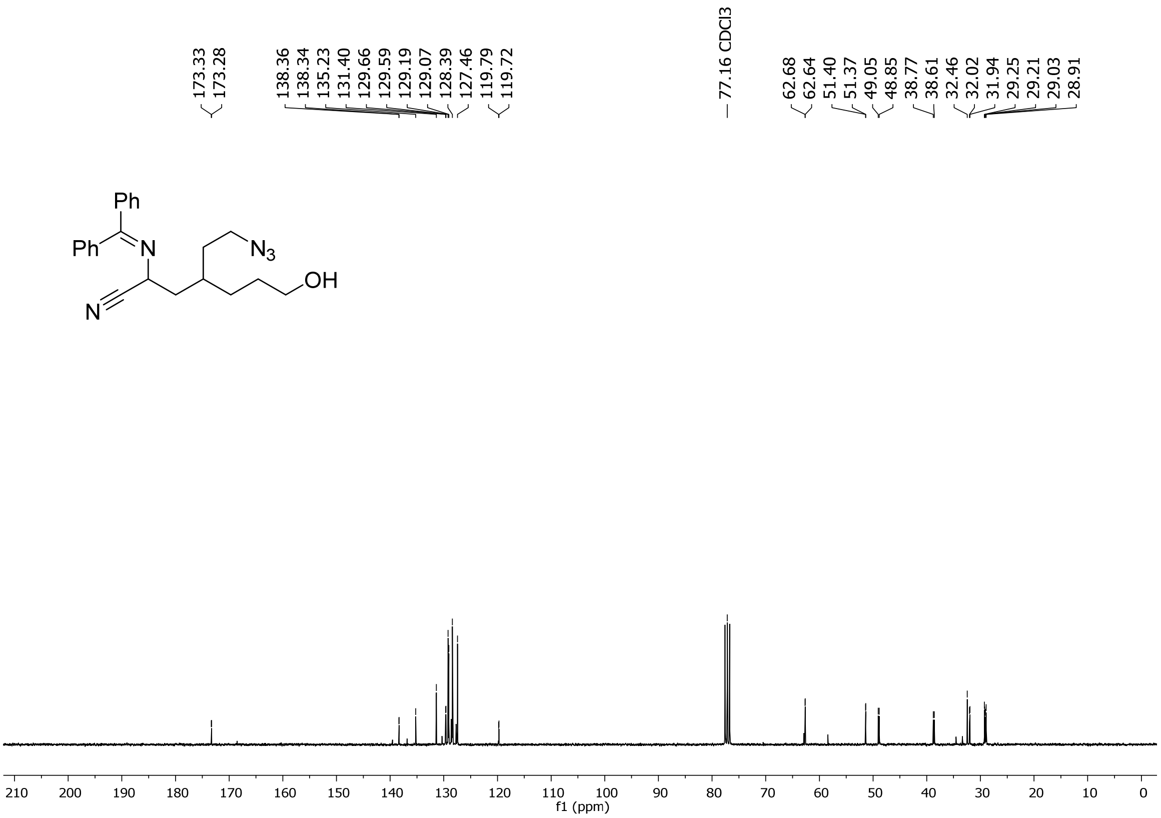

$^{13}C\{^{1}H\}$ NMR (75 MHz, $CDCl_3$) of compound **3w.**

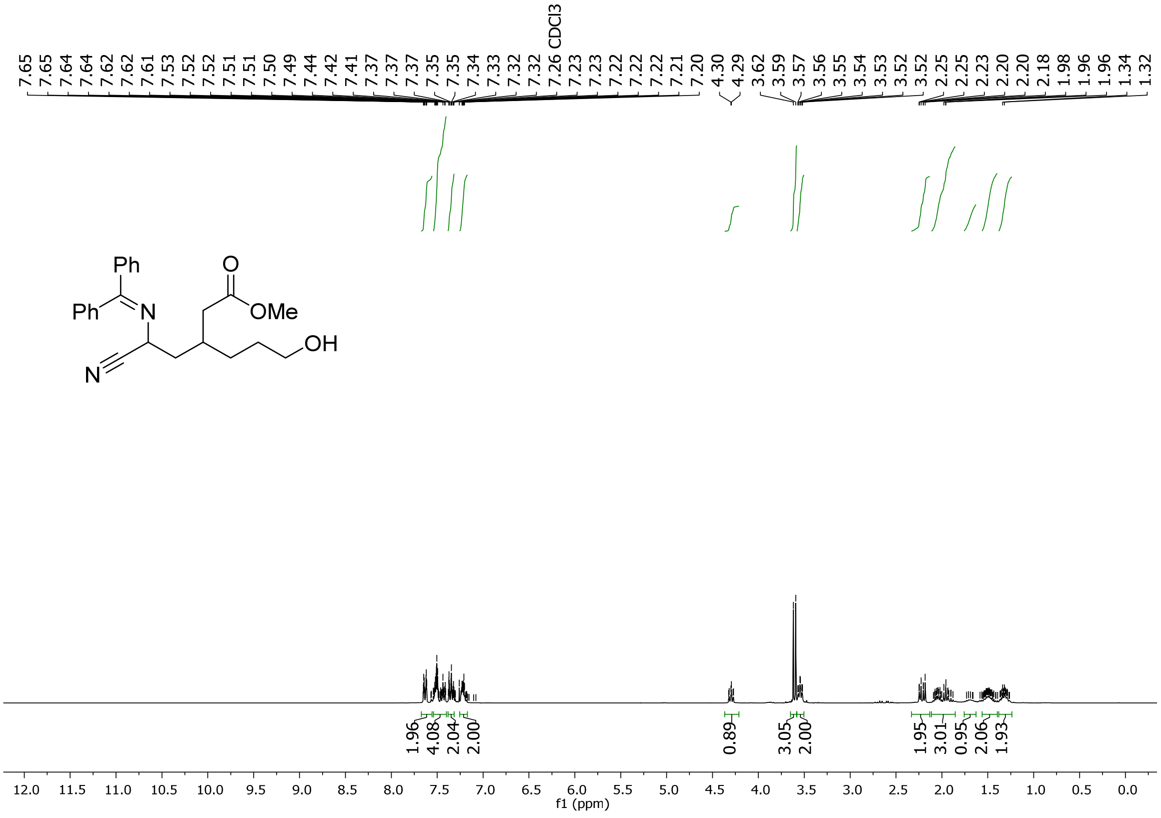

^{1}H NMR (300 MHz, $CDCl_3$) of compound **3x**.

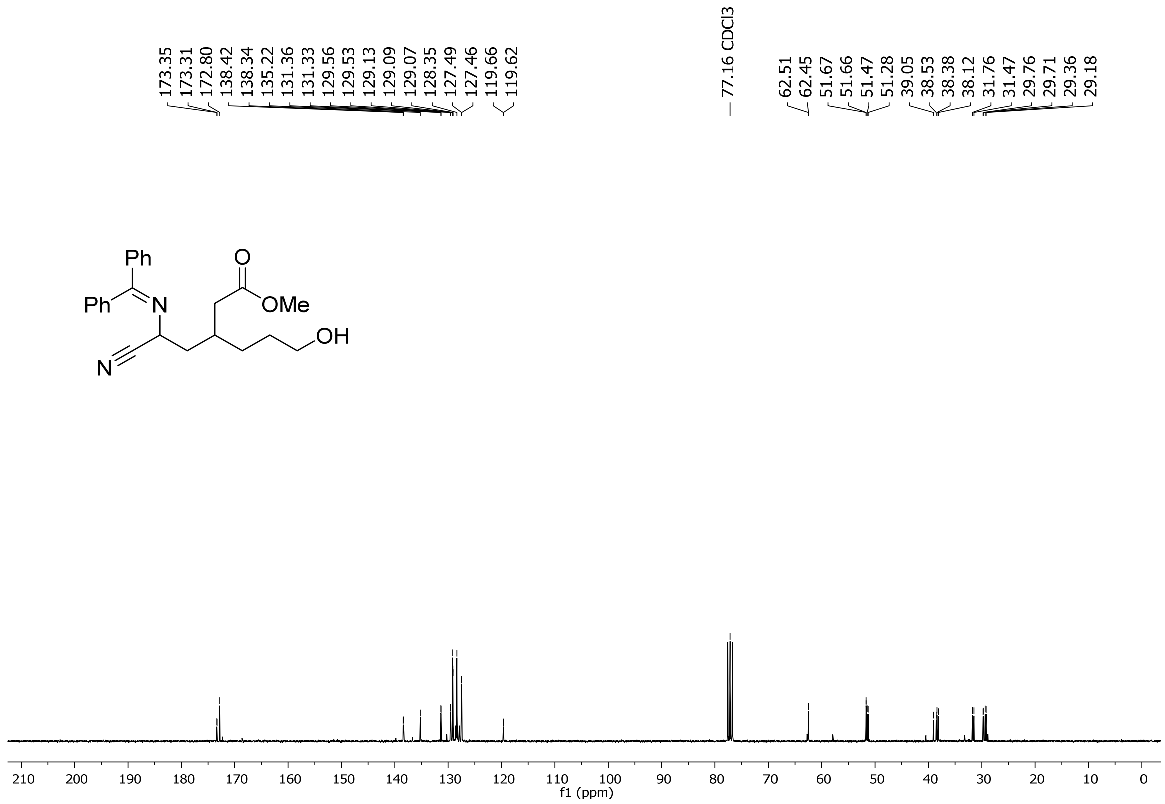

$^{13}C\{^{1}H\}$ NMR (75 MHz, $CDCl_3$) of compound **3x.**

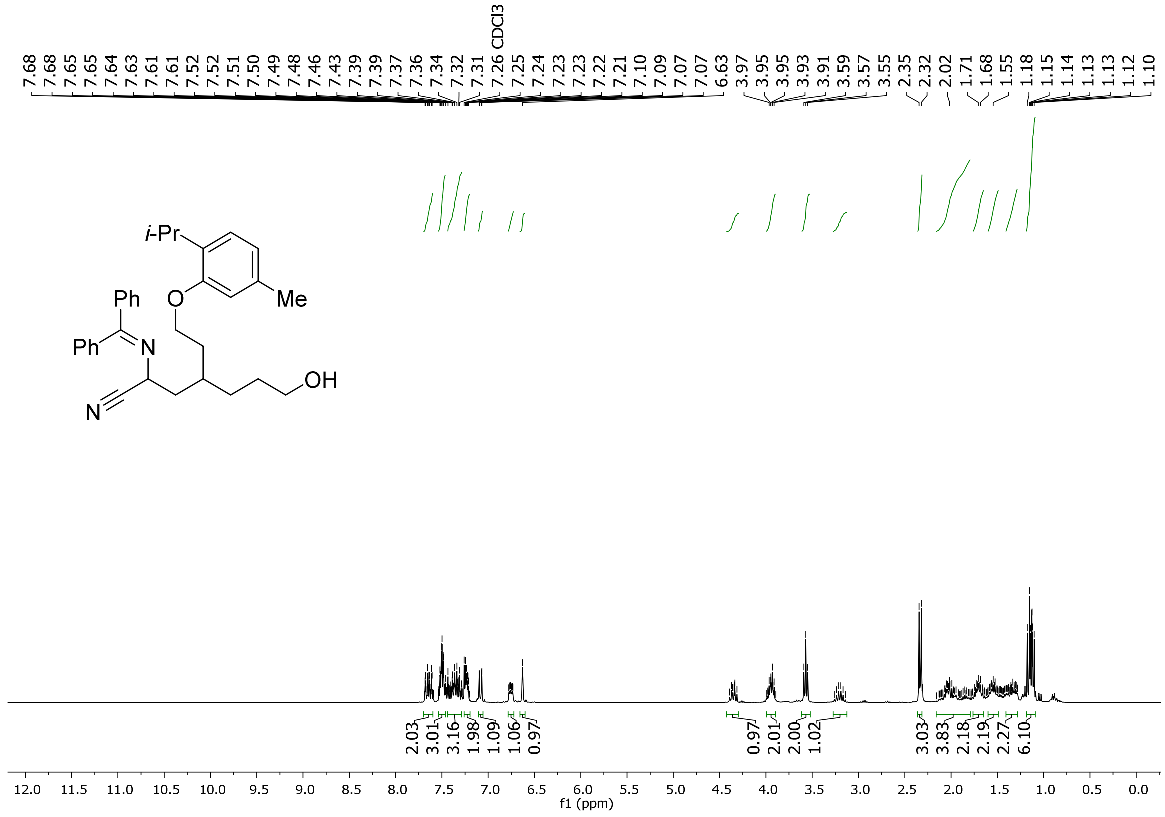

^{1}H NMR (300 MHz, $CDCl_3$) of compound **3y**.

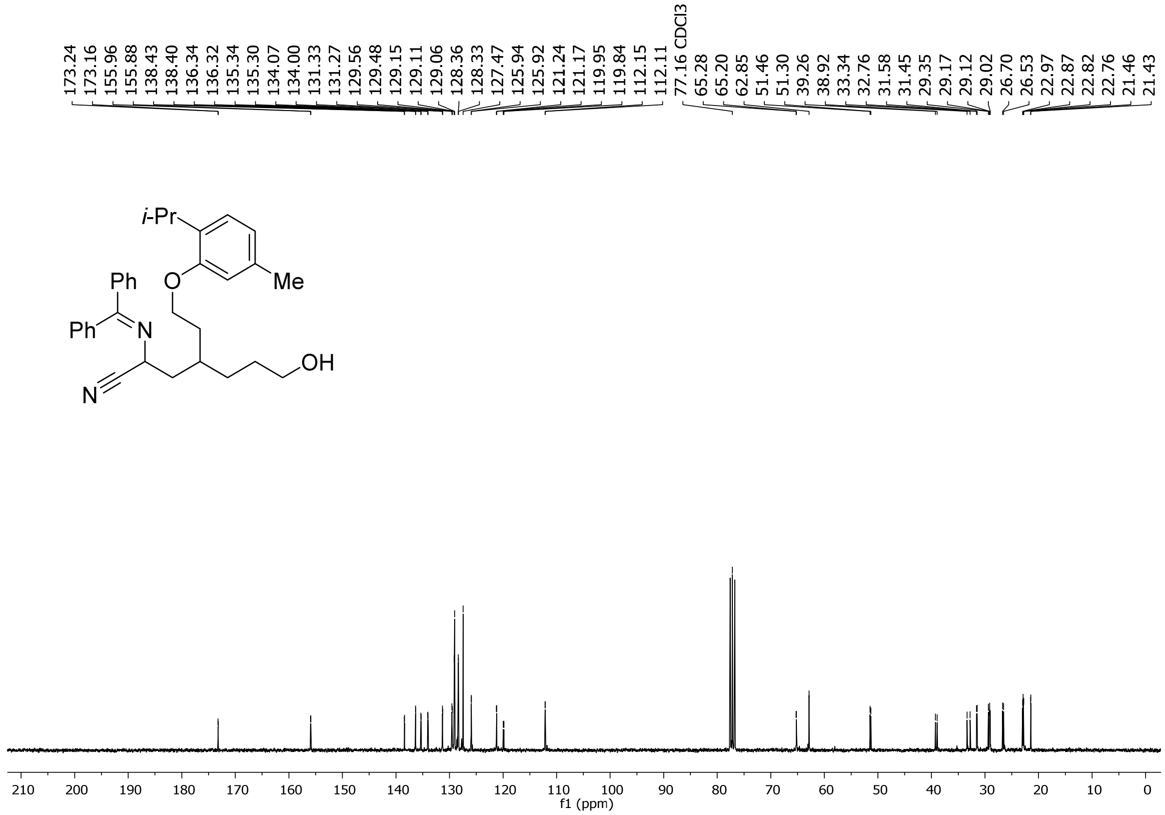

$^{13}C\{^{1}H\}$ NMR (76 MHz, $CDCl_3$) of compound **3y.**

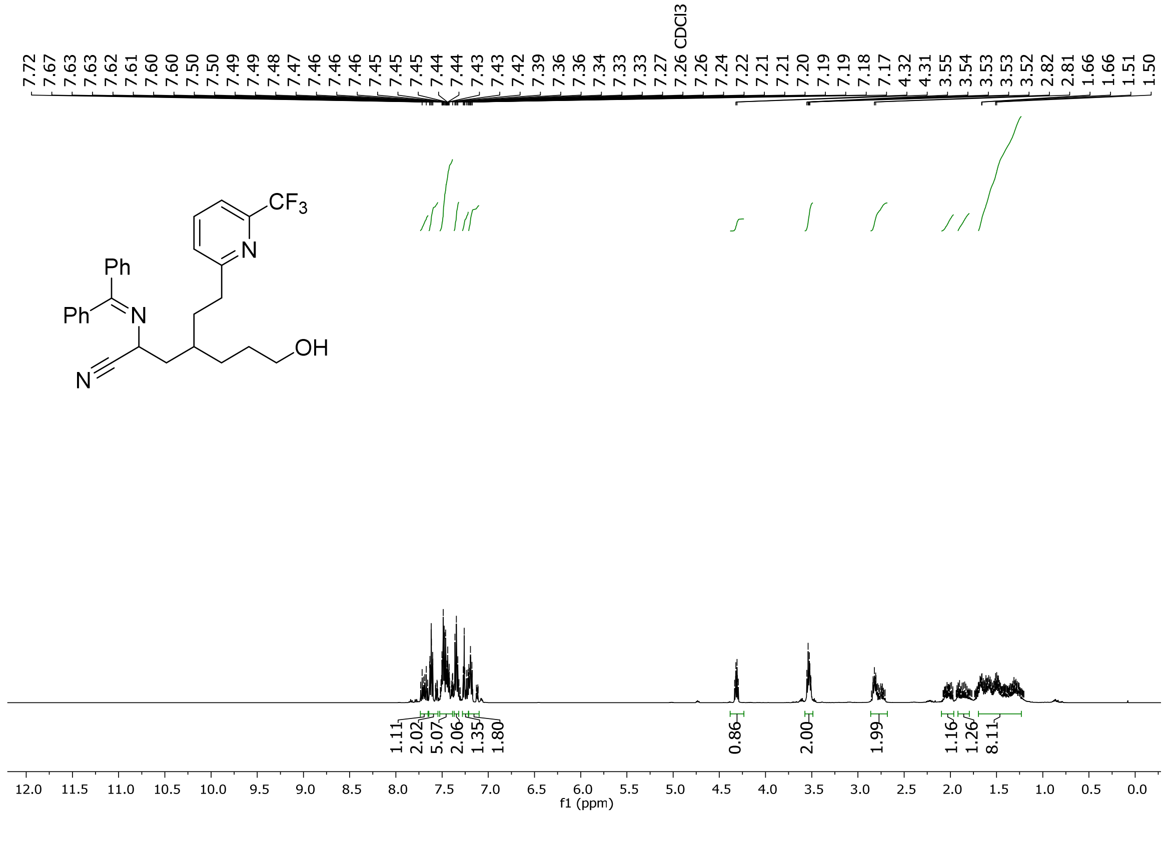

^{1}H NMR (500 MHz, $CDCl_3$) of compound **3z**.

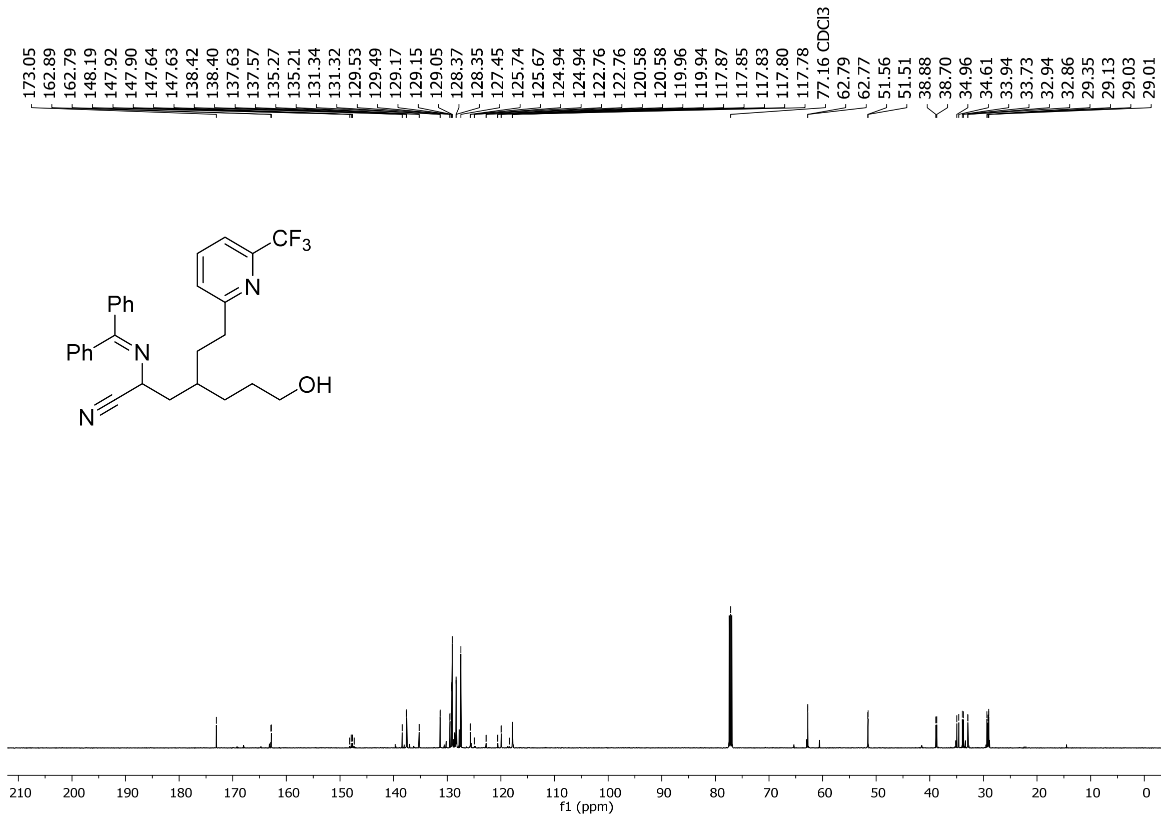

$^{13}C\{^1H\}$ NMR (126 MHz, $CDCl_3$) of compound **3z.**

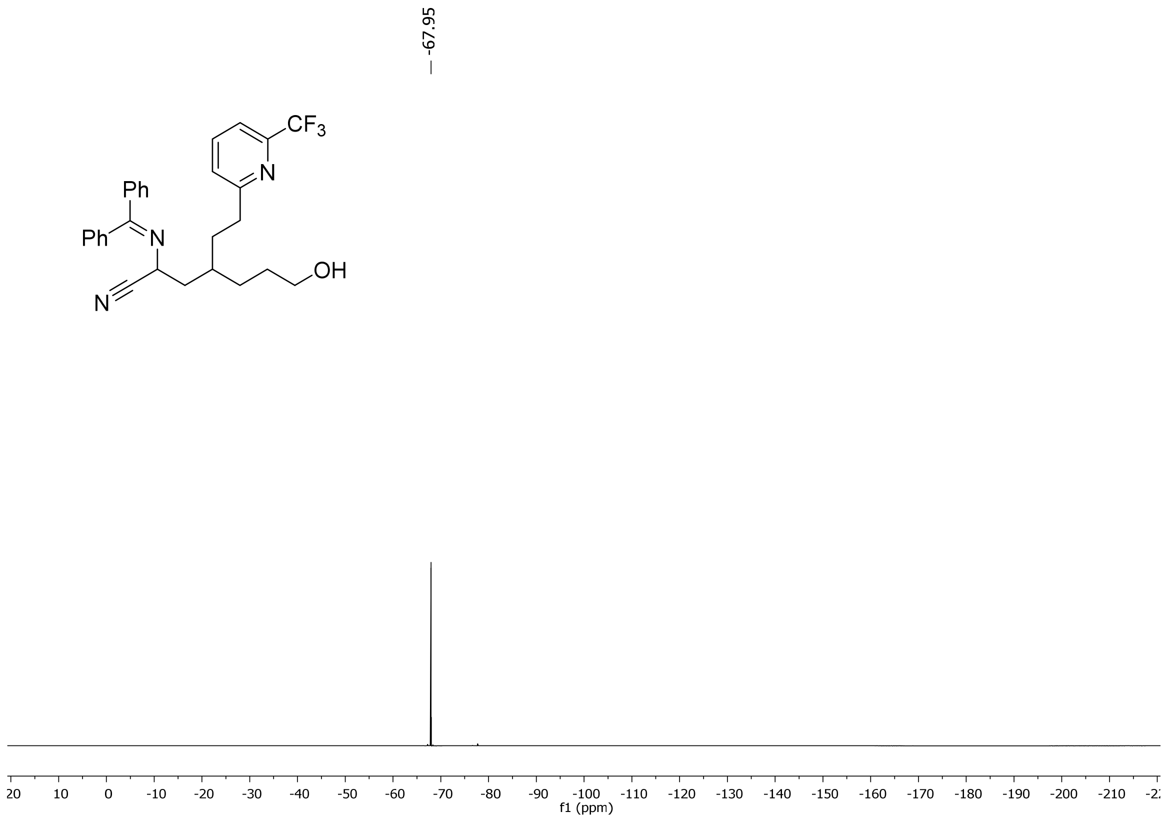

$^{19}F\{^1H\}$ NMR (471 MHz, $CDCl_3$) of compound **3z.**

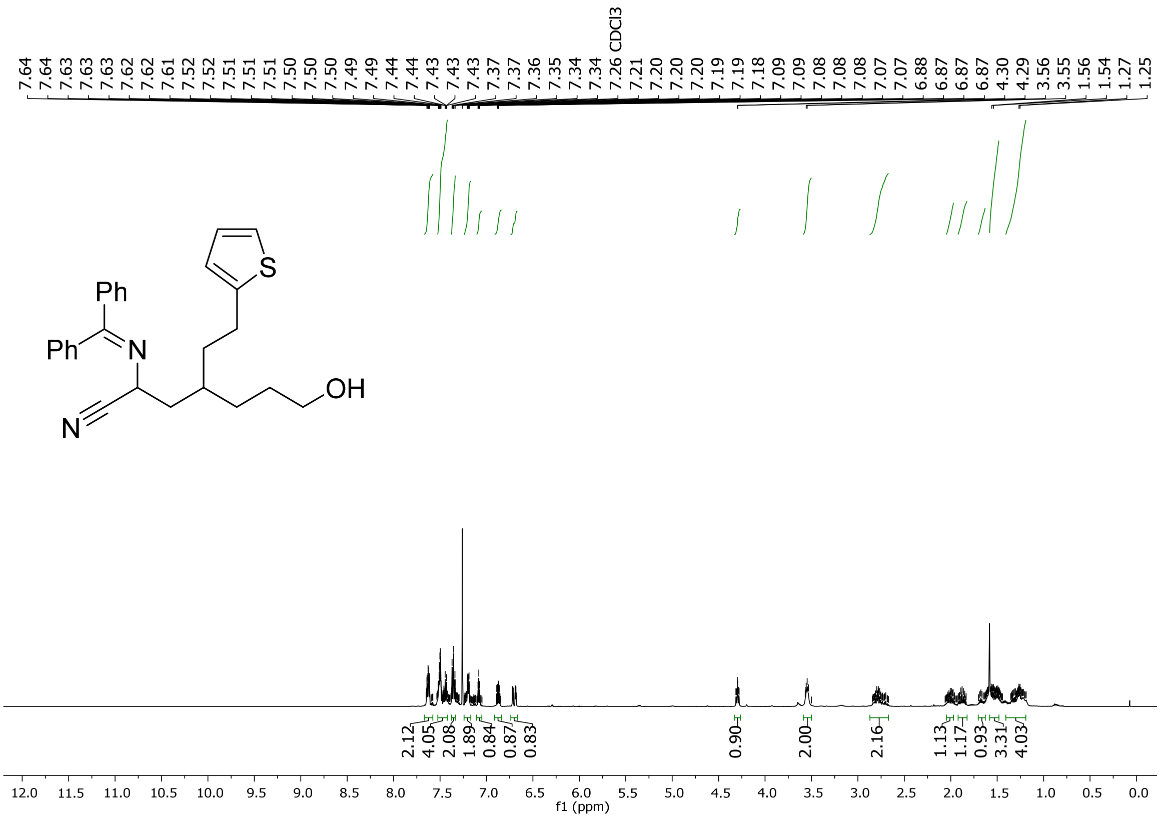

^{1}H NMR (500 MHz, $CDCl_3$) of compound **3aa**.

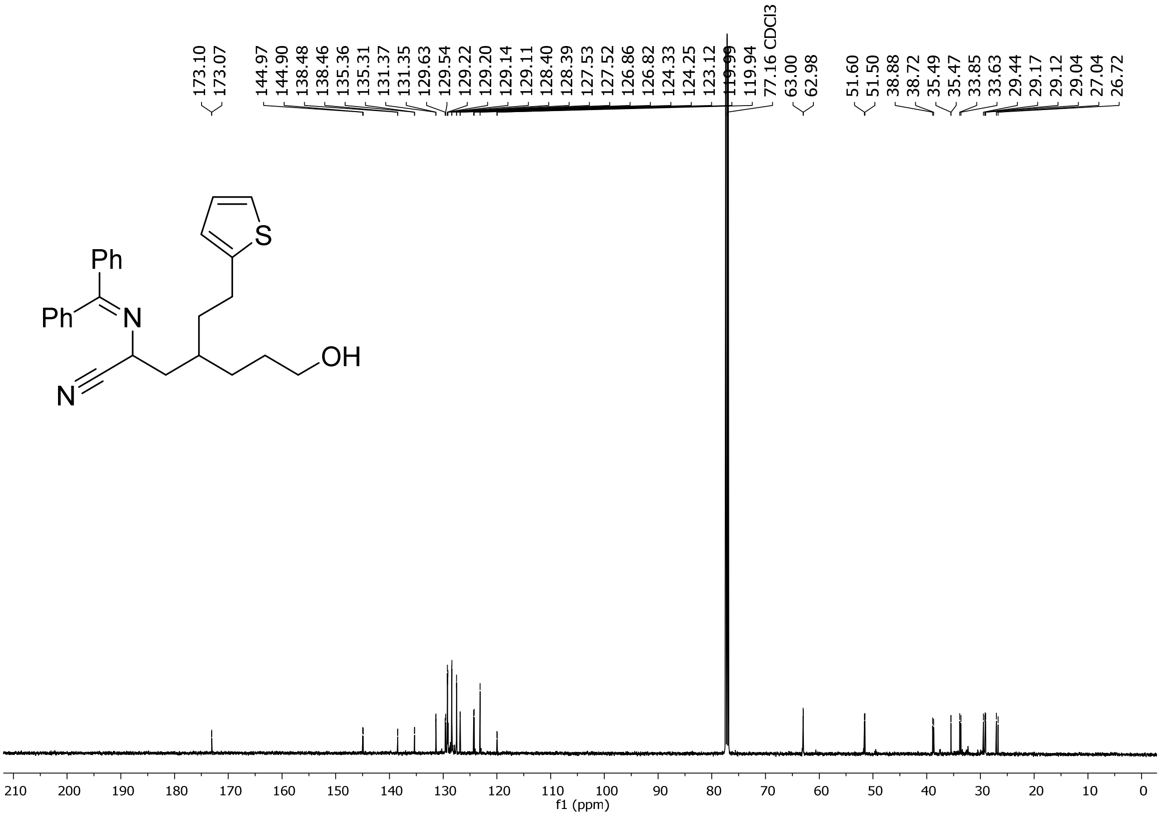

^{13}C{^{1}H} NMR (126 MHz, $CDCl_3$) of compound **3aa.**

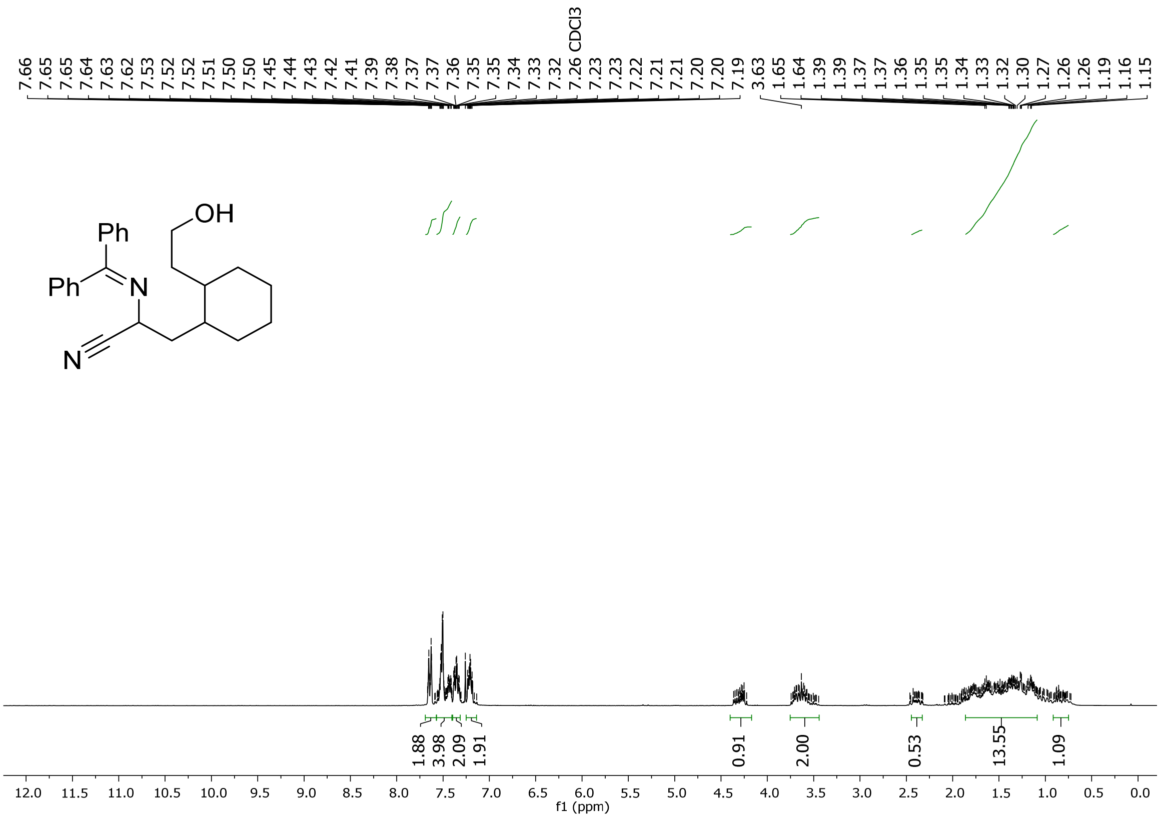

^{1}H NMR (300 MHz, $CDCl_3$) of compound **3ab**.

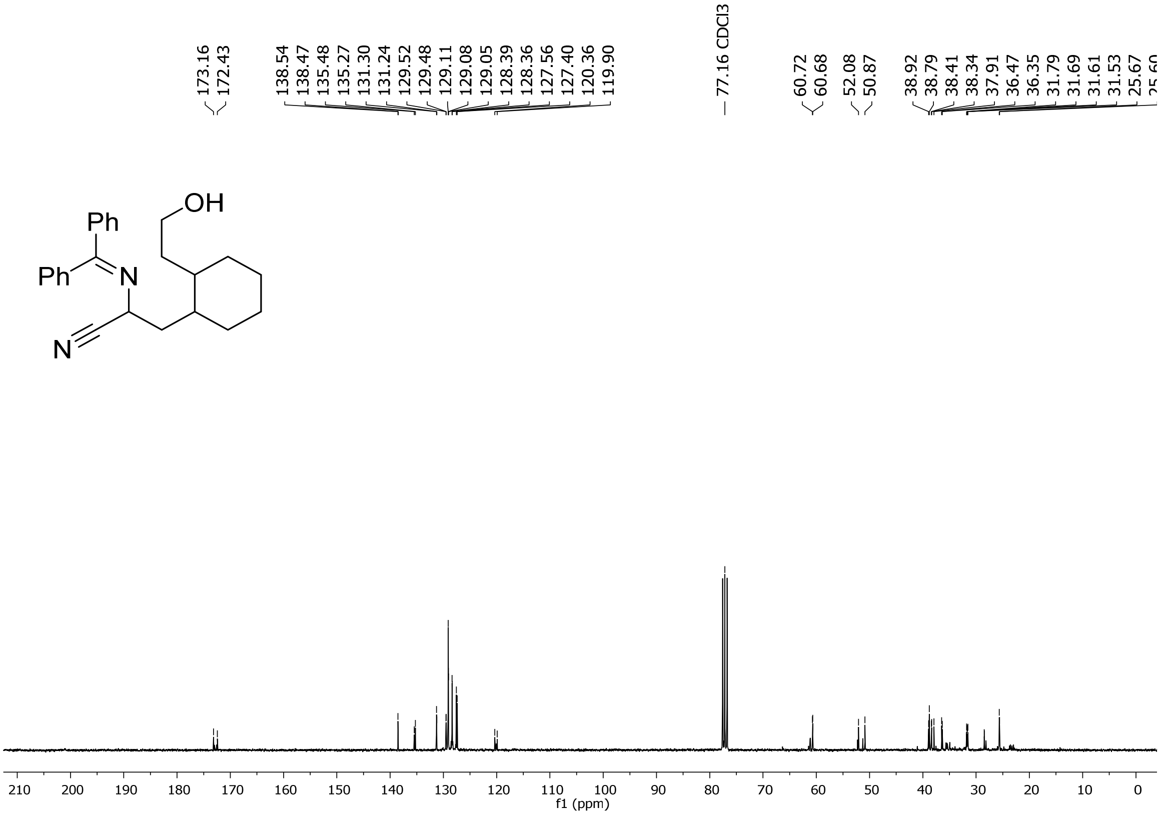

$^{13}C\{^{1}H\}$ NMR (76 MHz, $CDCl_3$) of compound **3ab.**

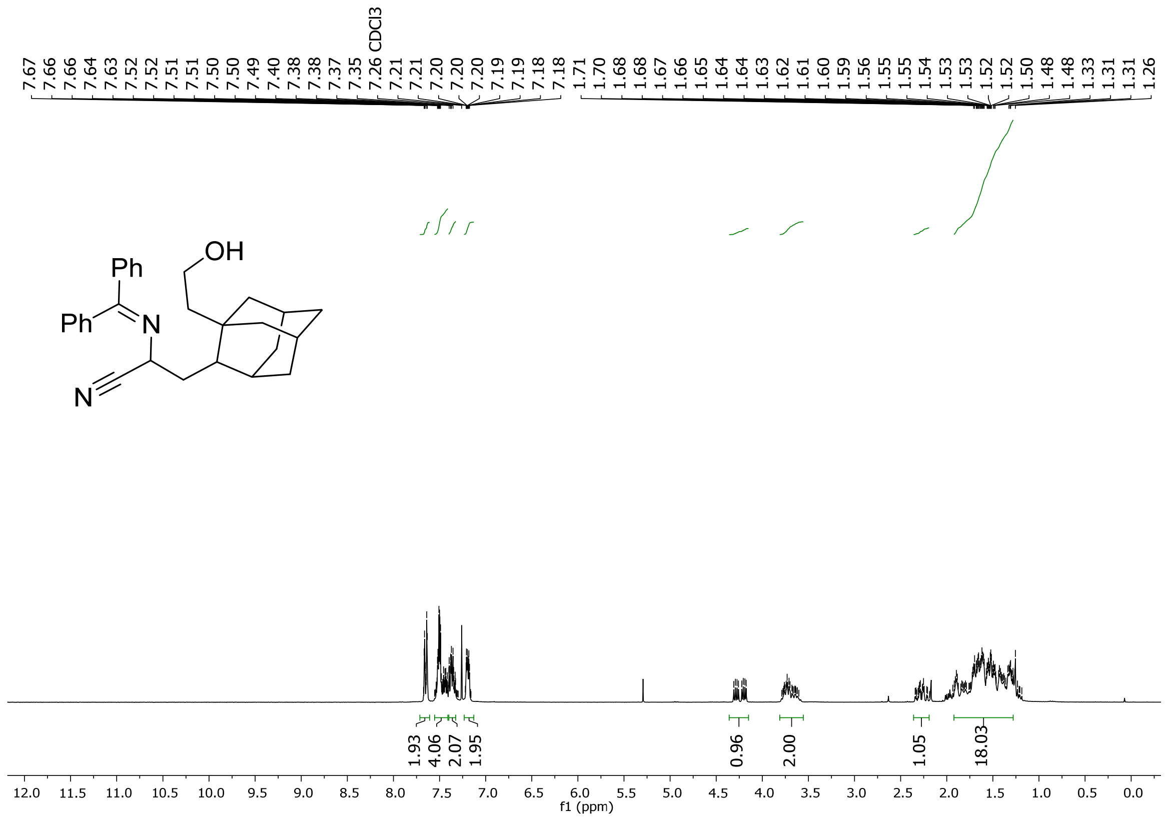

^{1}H NMR (300 MHz, $CDCl_3$) of compound **3ac**.

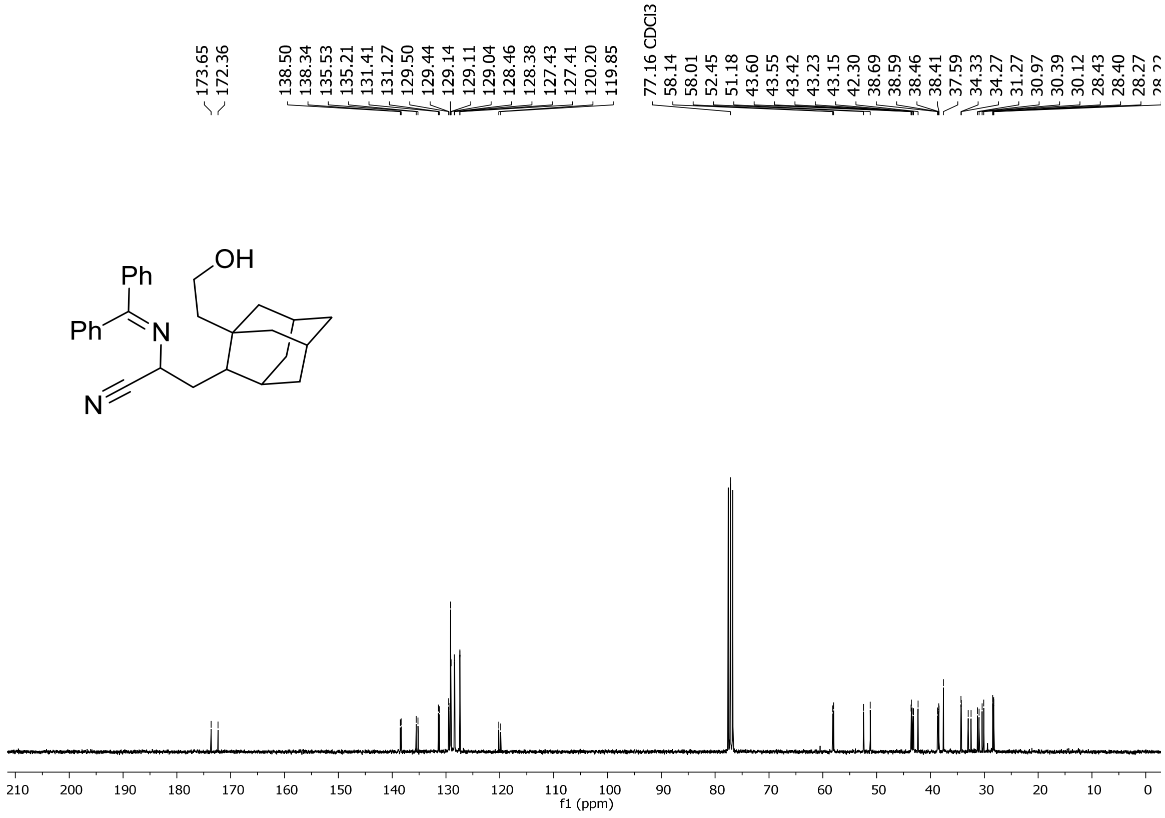

^{13}C{^{1}H} NMR (76 MHz, $CDCl_3$) of compound **3ac.**

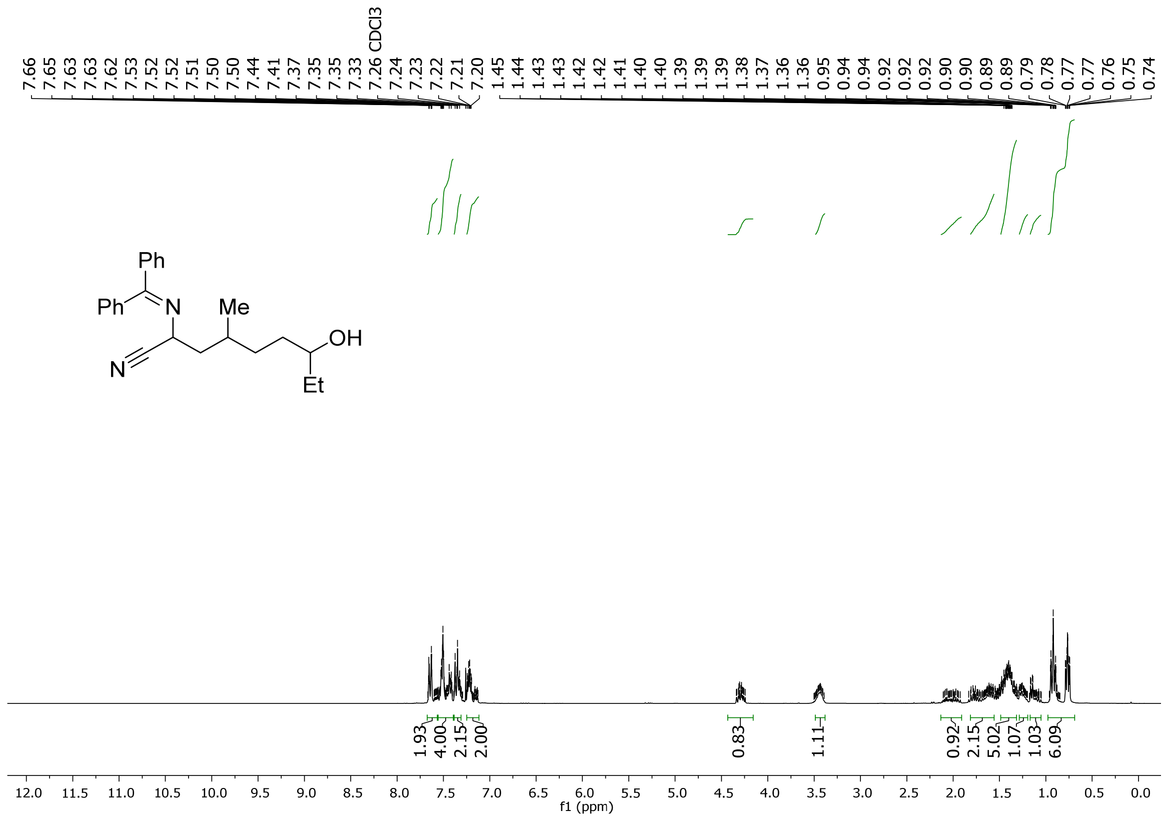

^{1}H NMR (300 MHz, $CDCl_3$) of compound **3ad**.

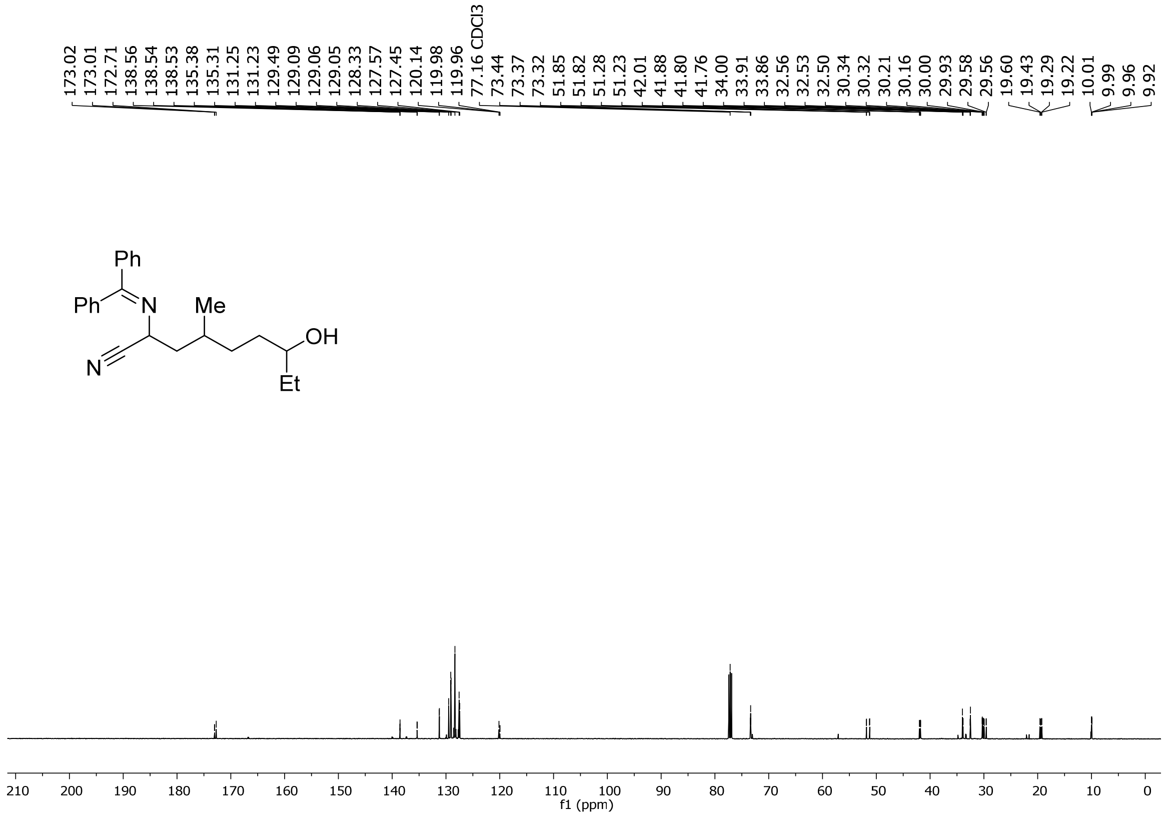

$^{13}C\{^{1}H\}$ NMR (126 MHz, $CDCl_3$) of compound **3ad.**

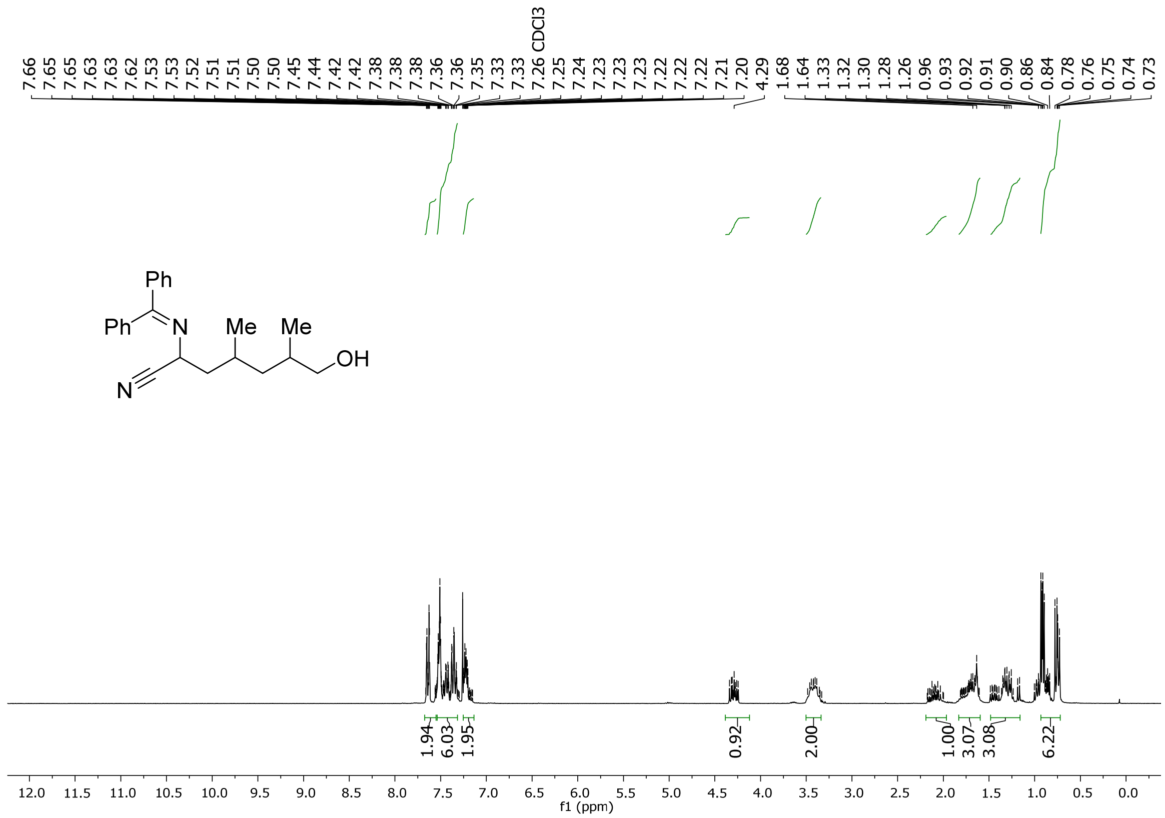

^{1}H NMR (300 MHz, $CDCl_3$) of compound **3ae**.

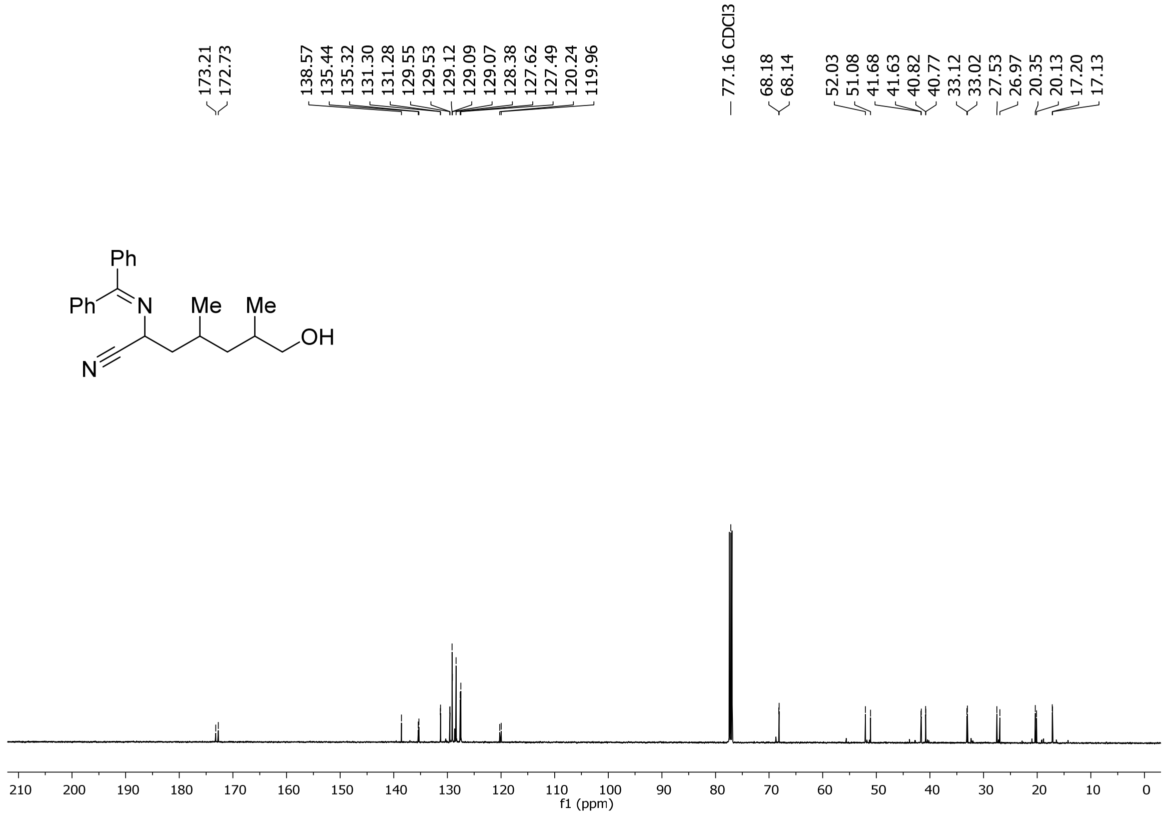

^{13}C{^{1}H} NMR (126 MHz, $CDCl_3$) of compound **3ae.**

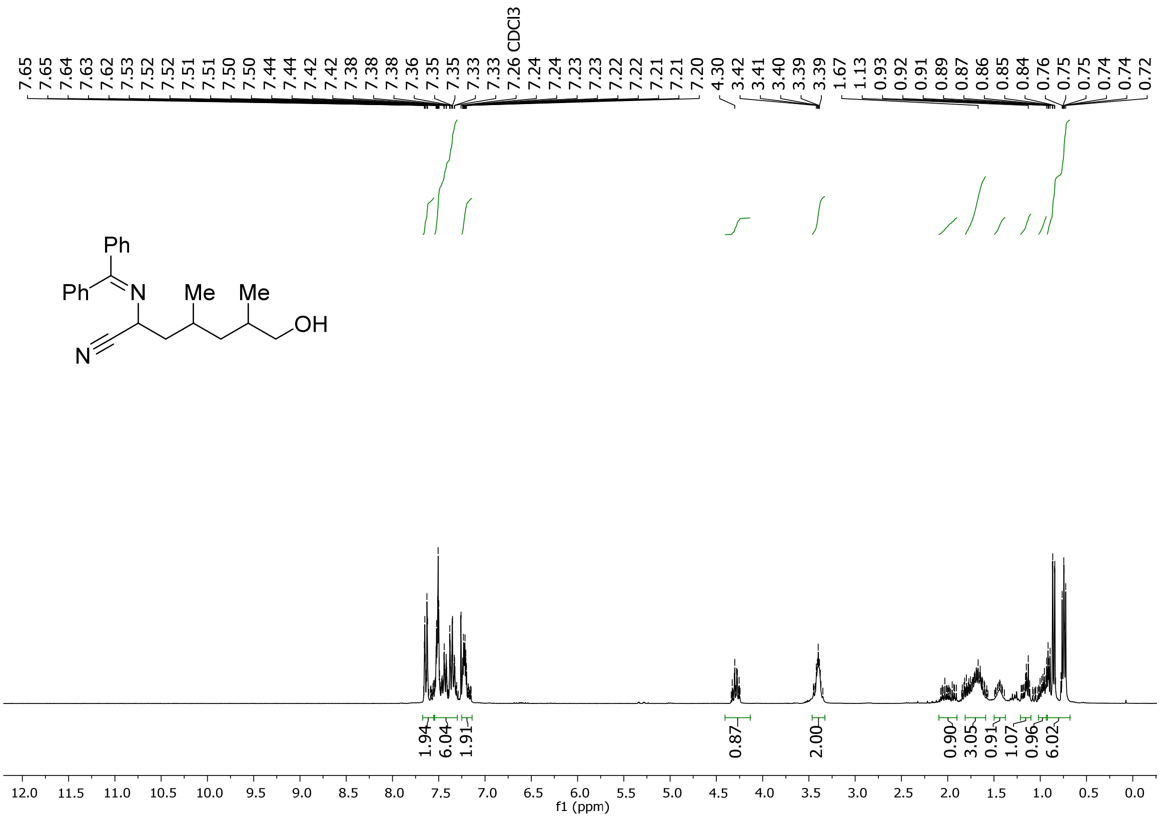

^{1}H NMR (300 MHz, $CDCl_3$) of compound **3ae'**.

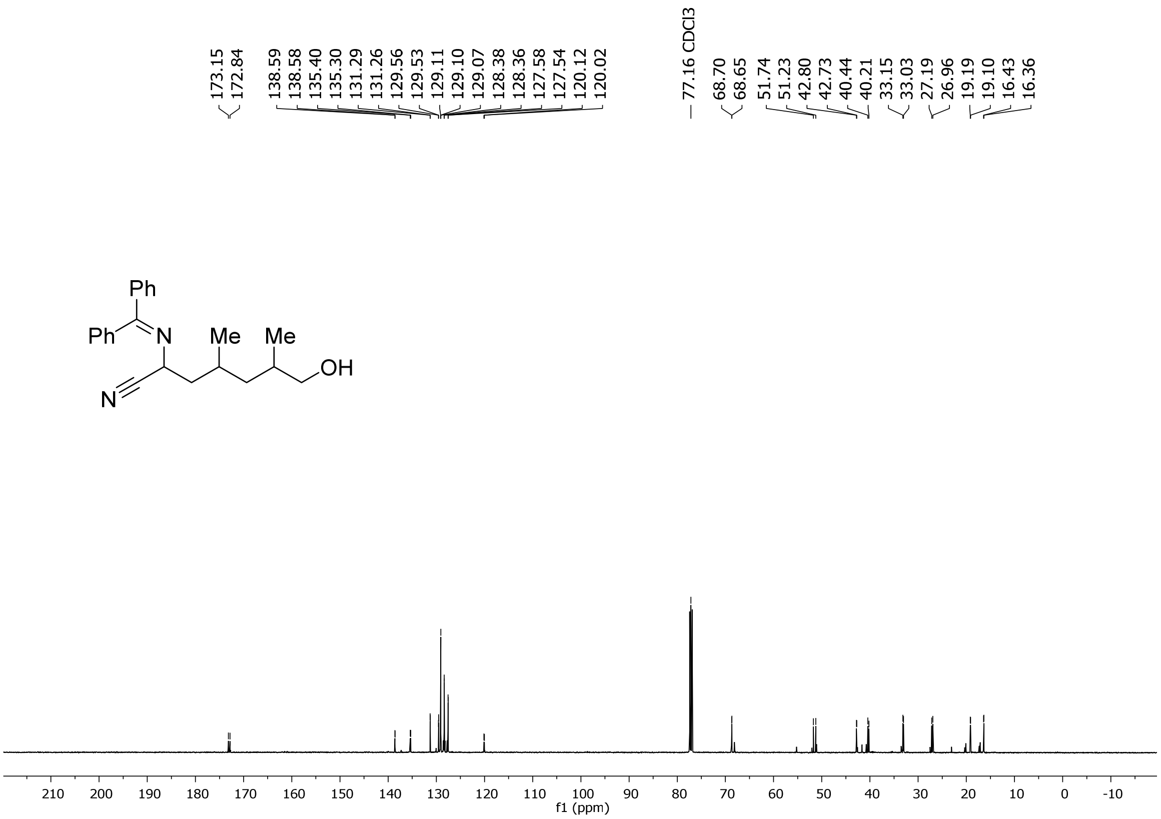

^{13}C{^{1}H} NMR (126 MHz, $CDCl_3$) of compound **3ae'.**

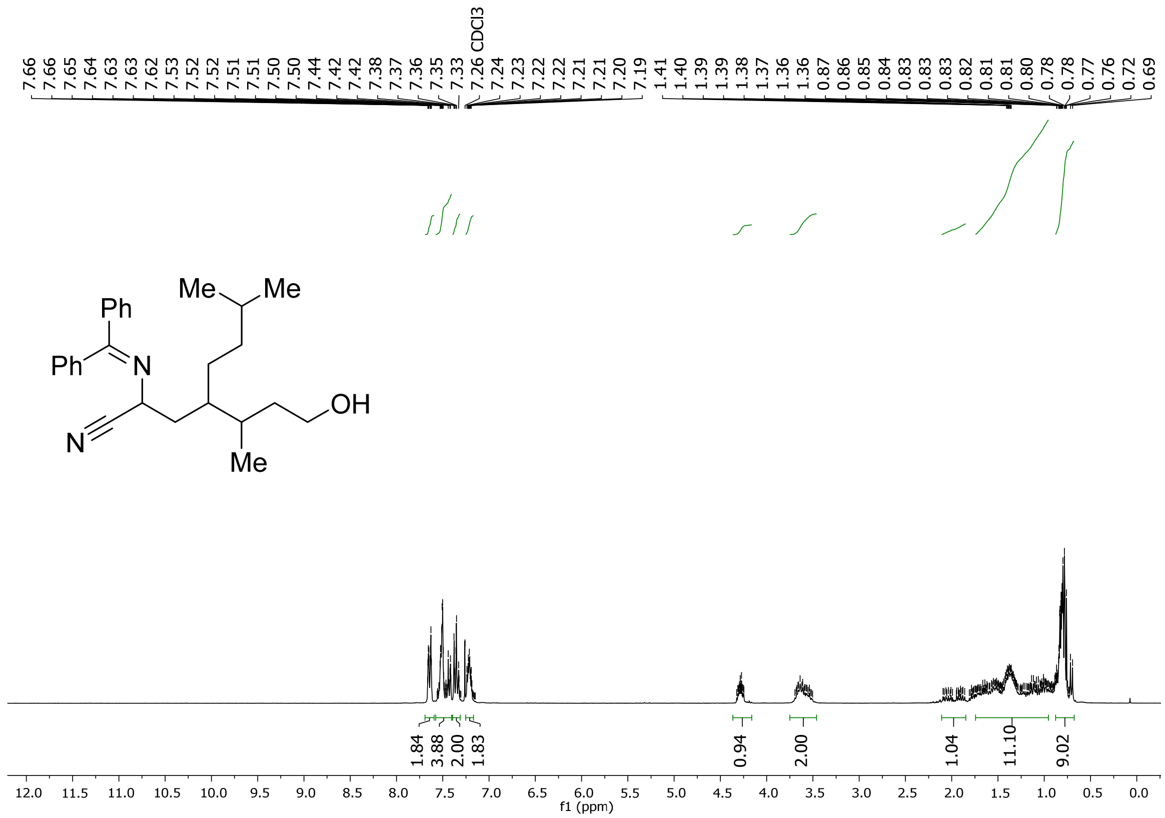

^{1}H NMR (300 MHz, $CDCl_3$) of compound **3af**.

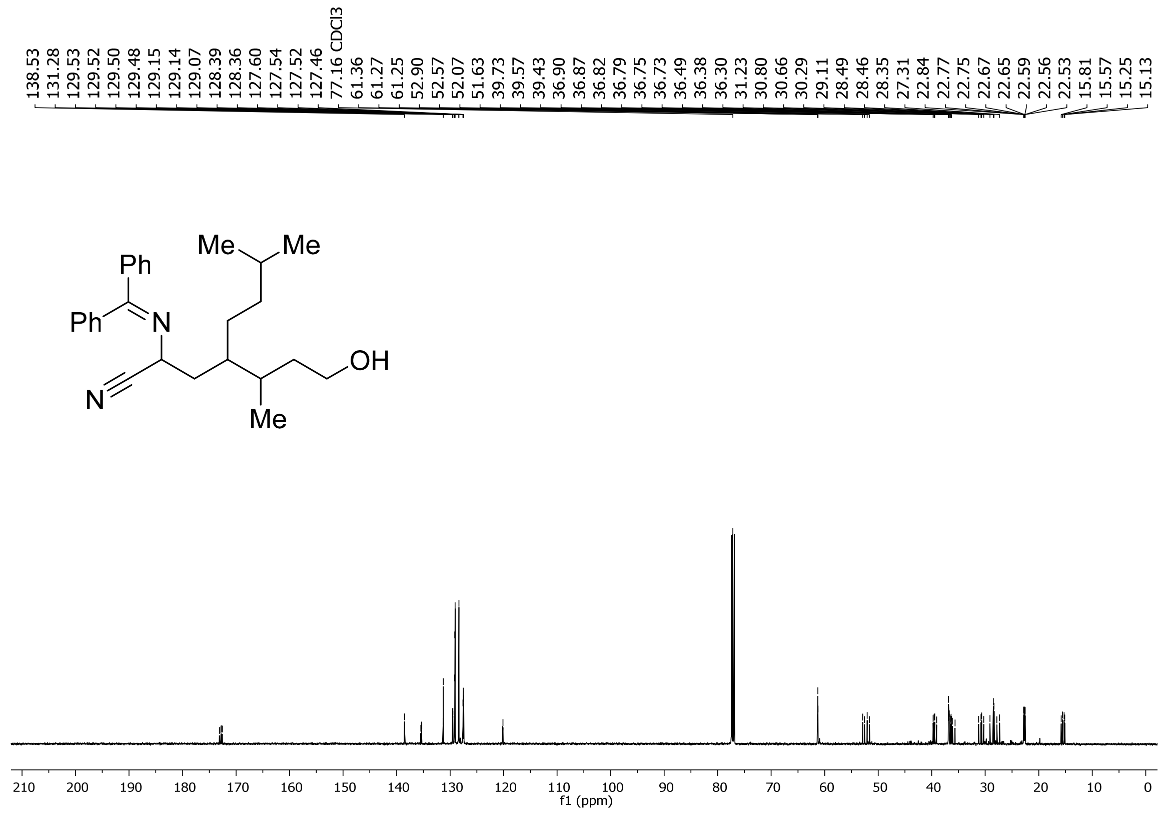

^{13}C{^{1}H} NMR (126 MHz, $CDCl_3$) of compound **3af.**

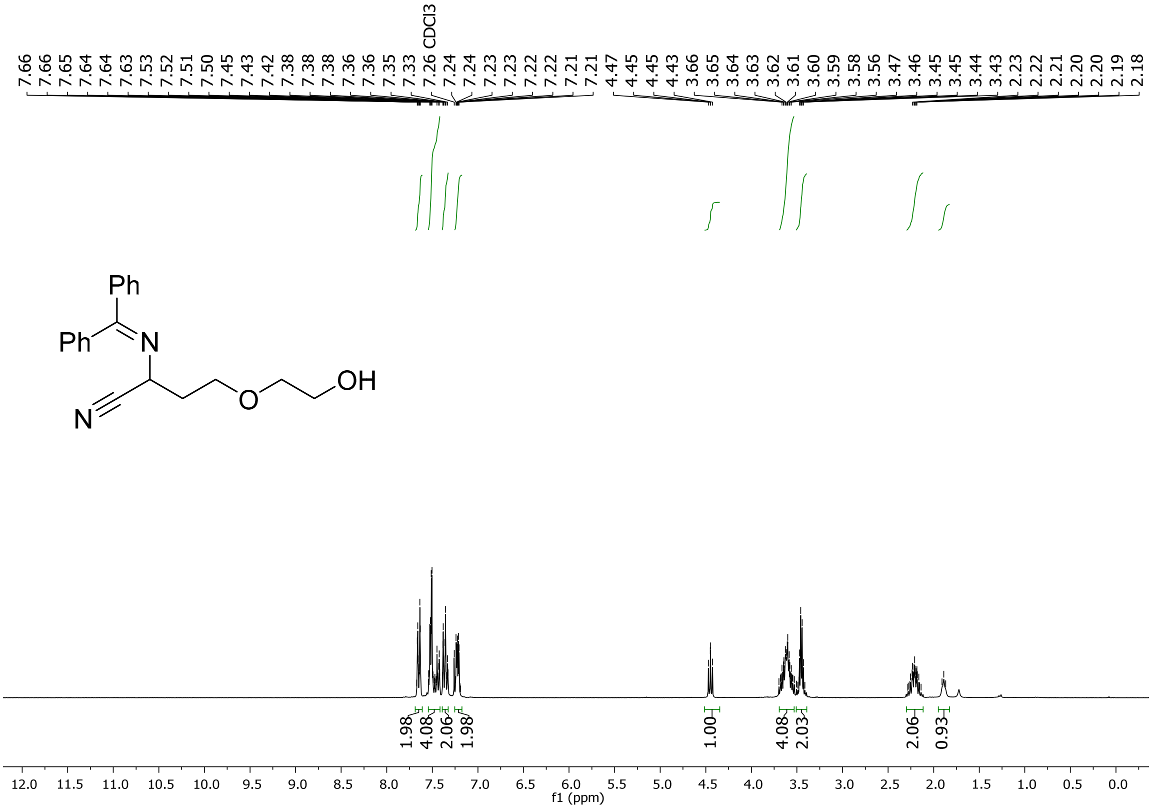

^{1}H NMR (300 MHz, CDCl$_3$) of compound **3ag**.

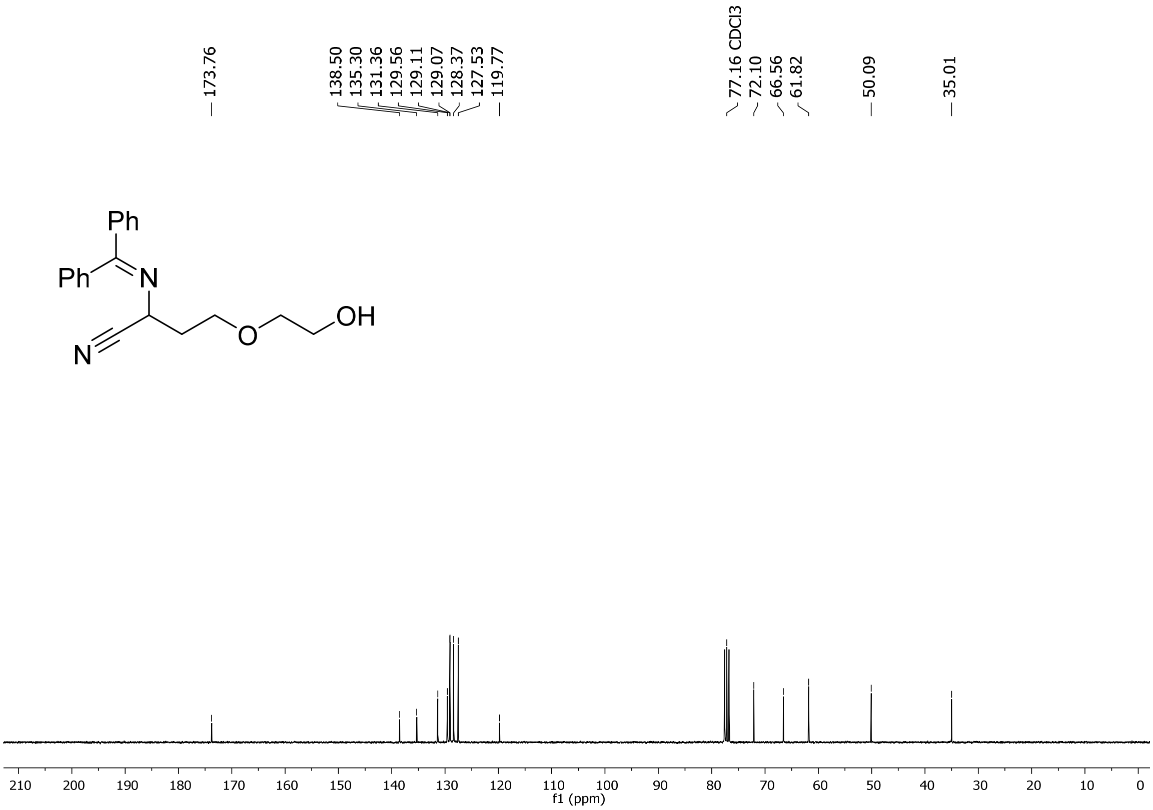

^{13}C{^{1}H} NMR (75 MHz, CDCl$_3$) of compound **3ag.**

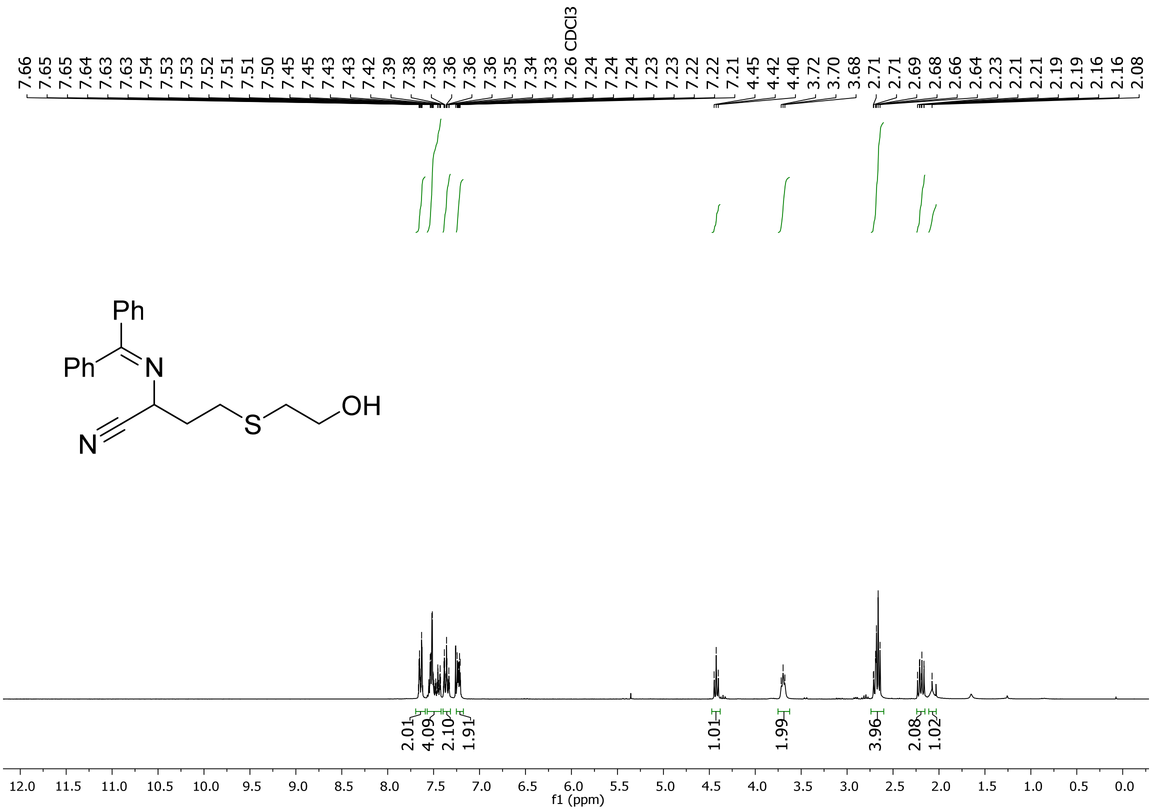

^{1}H NMR (300 MHz, $CDCl_3$) of compound **3ah**.

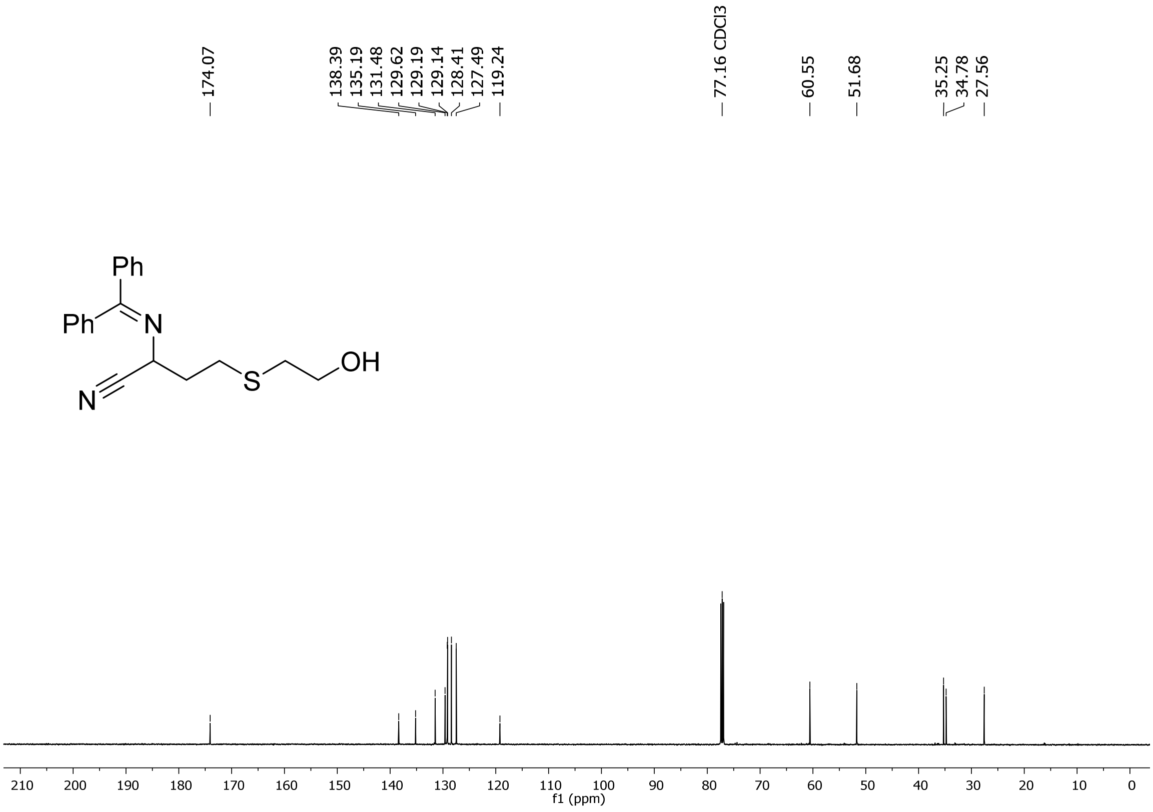

^{13}C{^{1}H} NMR (126 MHz, $CDCl_3$) of compound **3ah.**

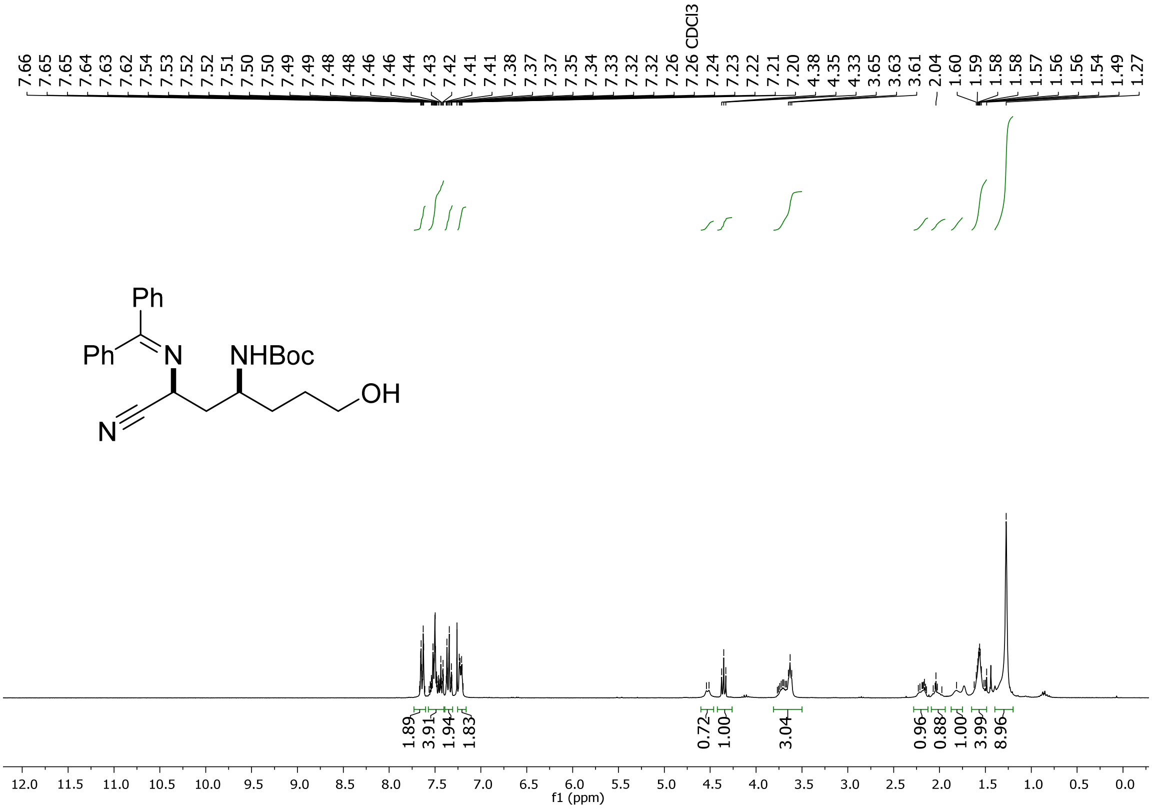

^{1}H NMR (300 MHz, $CDCl_3$) of compound ***syn*-3ai**.

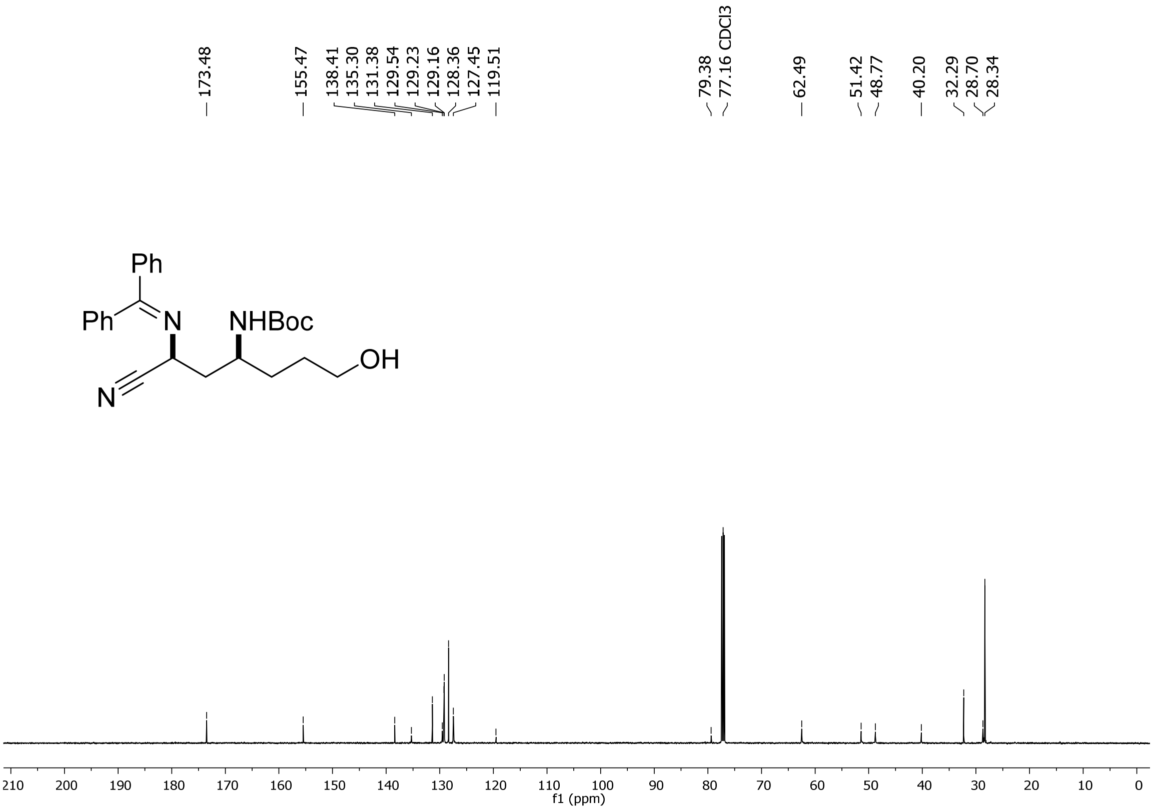

$^{13}C\{^{1}H\}$ NMR (126 MHz, $CDCl_3$) of compound ***syn*-3ai.**

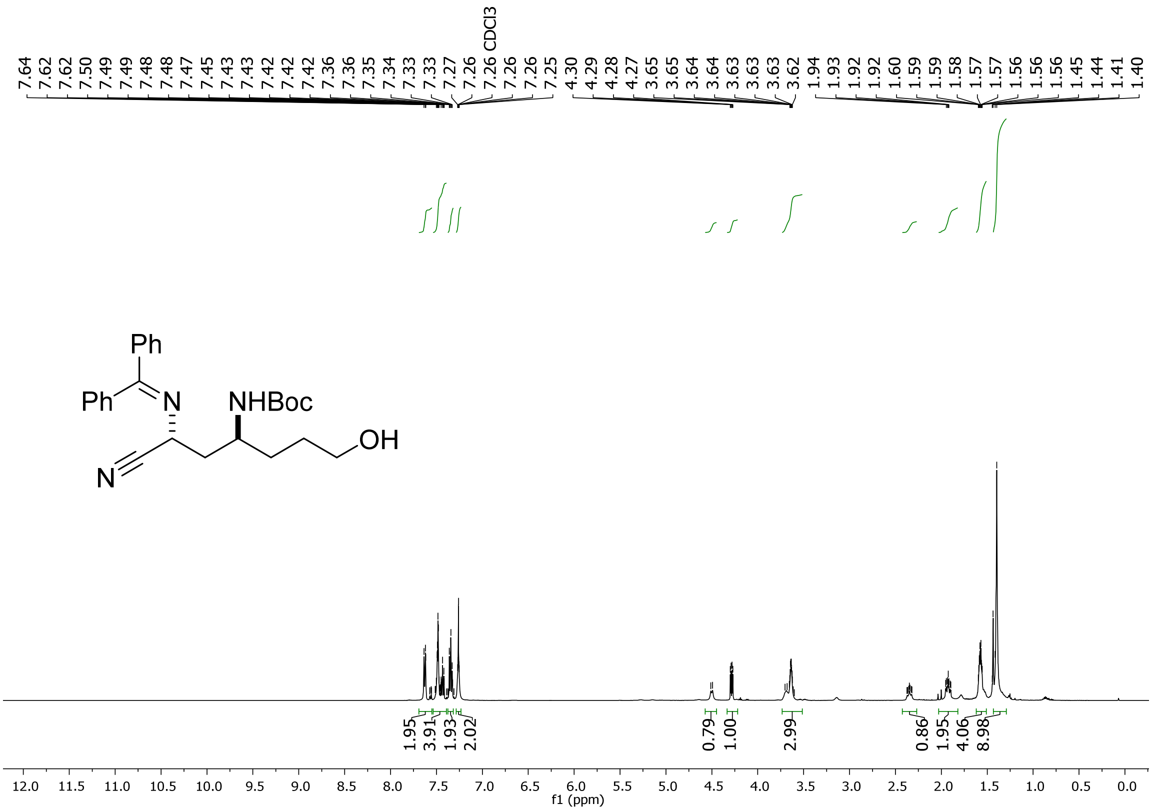

^{1}H NMR (500 MHz, $CDCl_3$) of compound ***anti*-3ai'**.

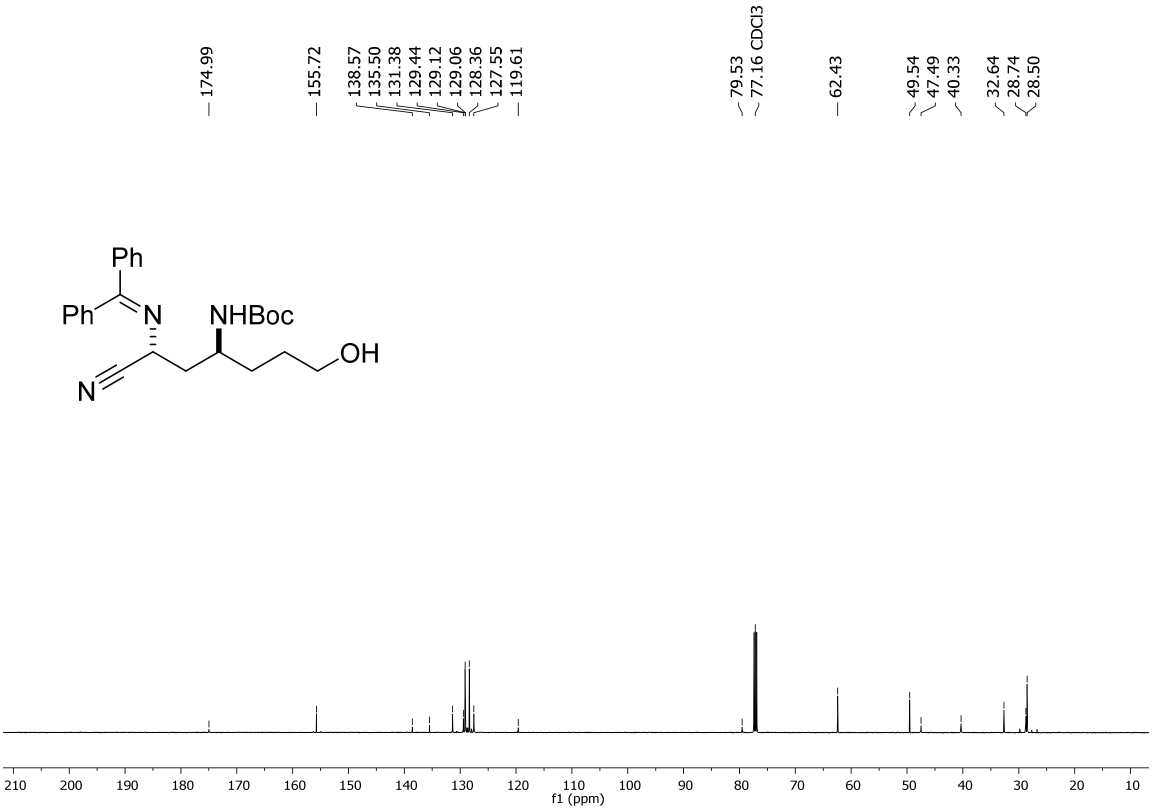

$^{13}C\{^{1}H\}$ NMR (126 MHz, $CDCl_3$) of compound ***anti*-3ai'.**

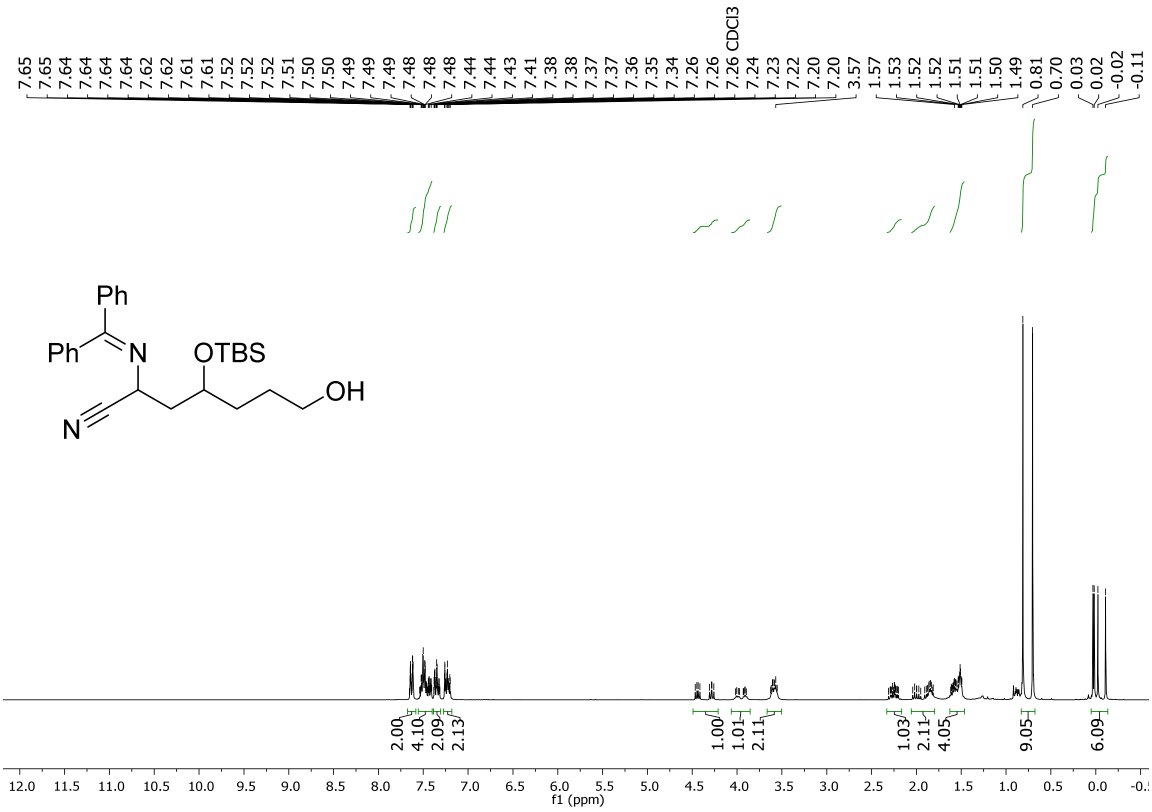

^{1}H NMR (300 MHz, $CDCl_3$) of compound **3aj**.

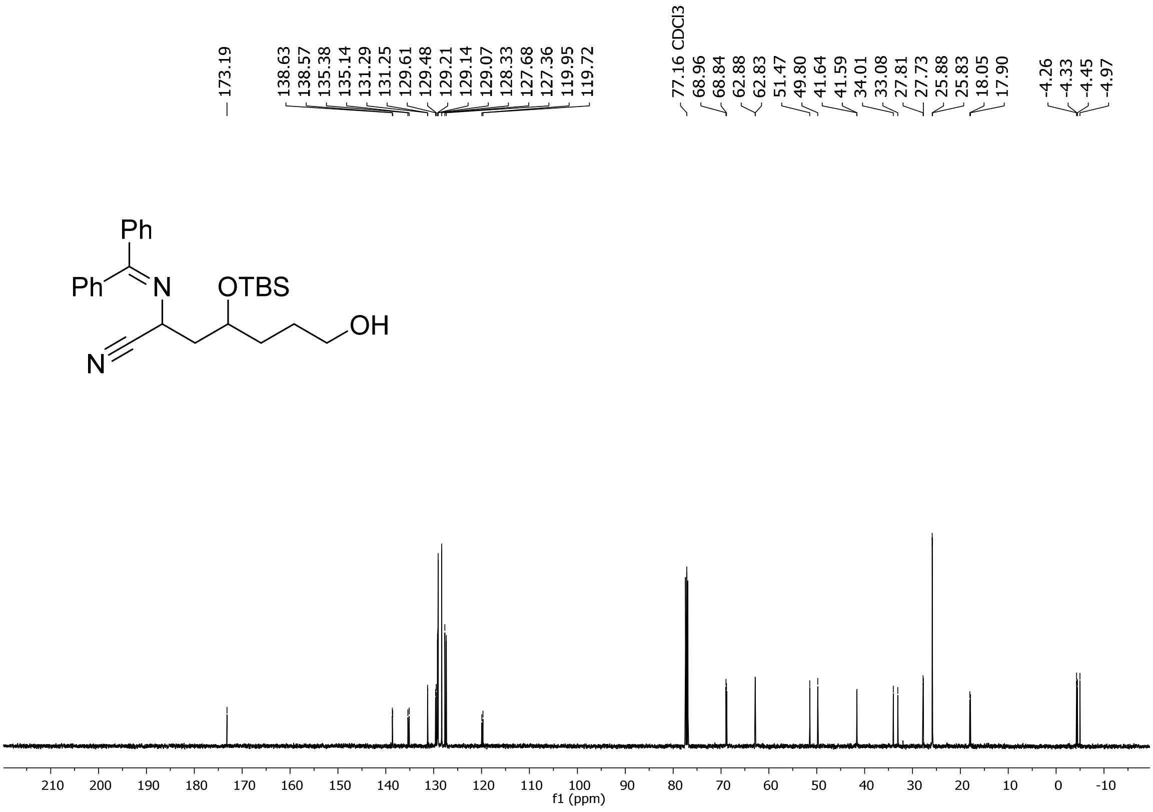

^{13}C{^{1}H} NMR (126 MHz, $CDCl_3$) of compound **3aj.**

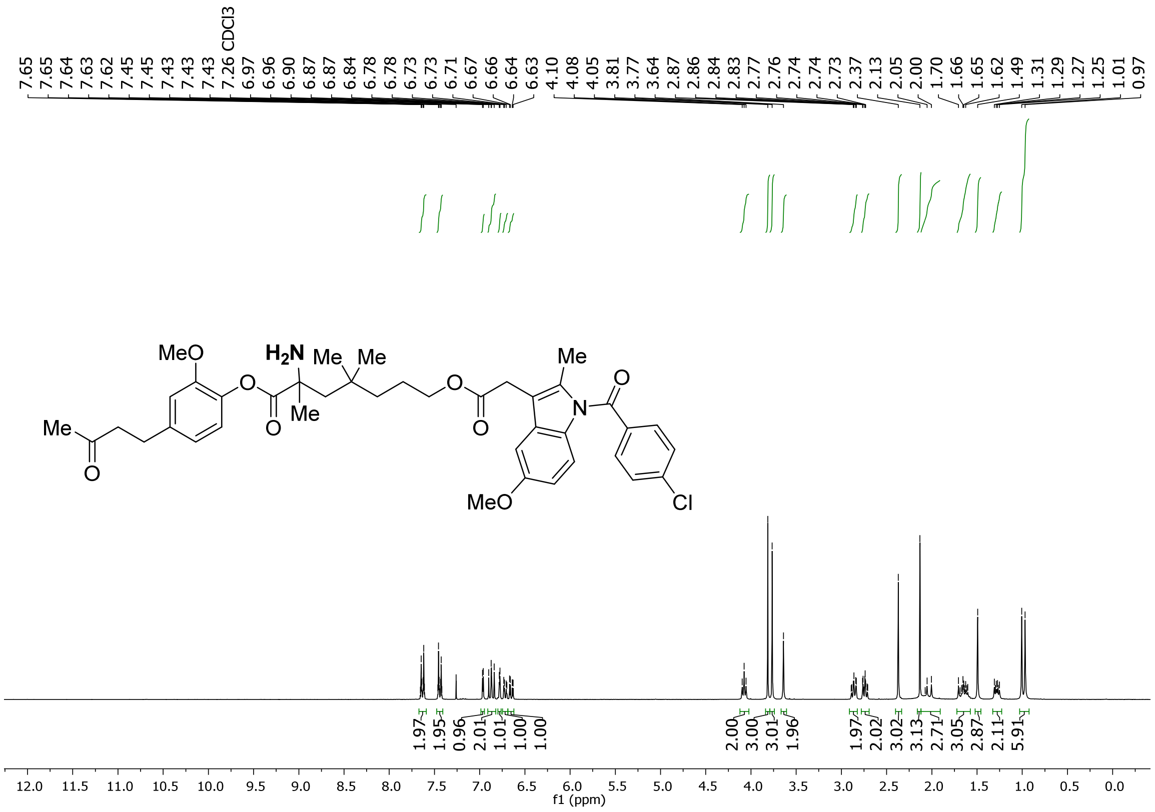

^{1}H NMR (300 MHz, $CDCl_3$) of compound **4**.

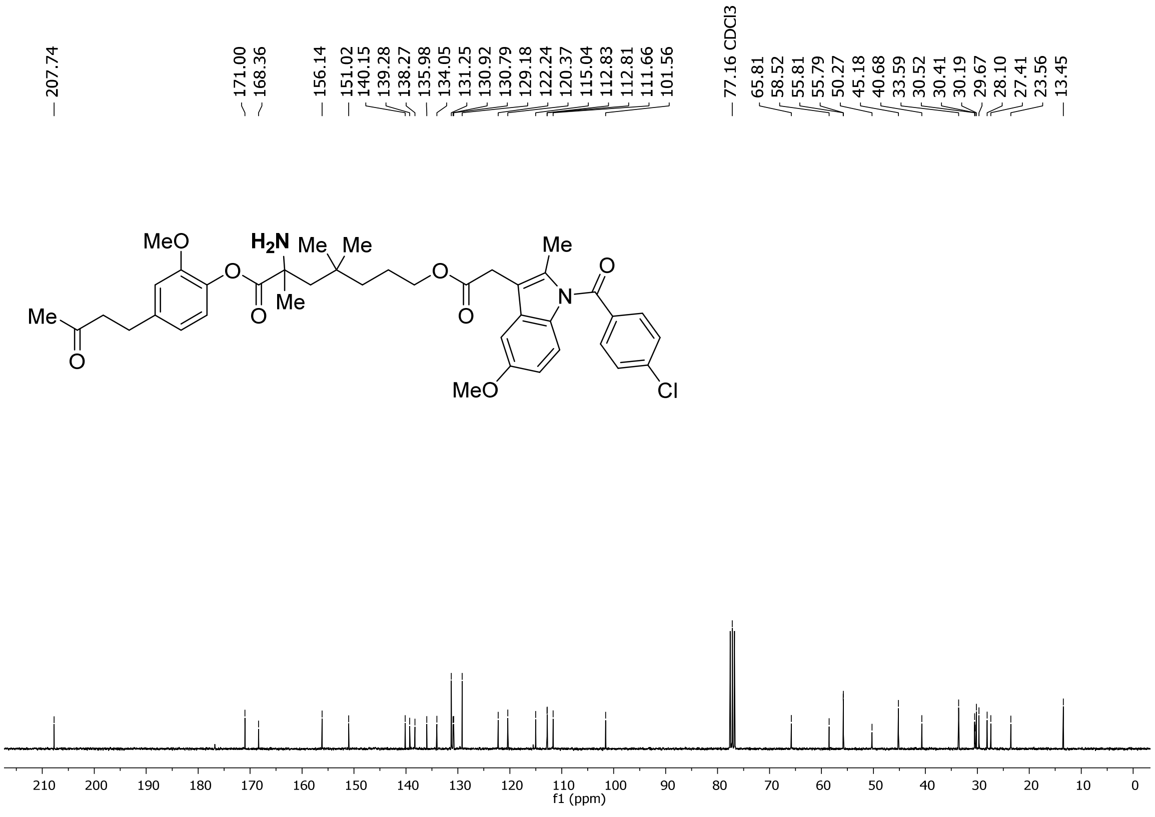

$^{13}C\{^{1}H\}$ NMR (76 MHz, $CDCl_3$) of compound **4.**

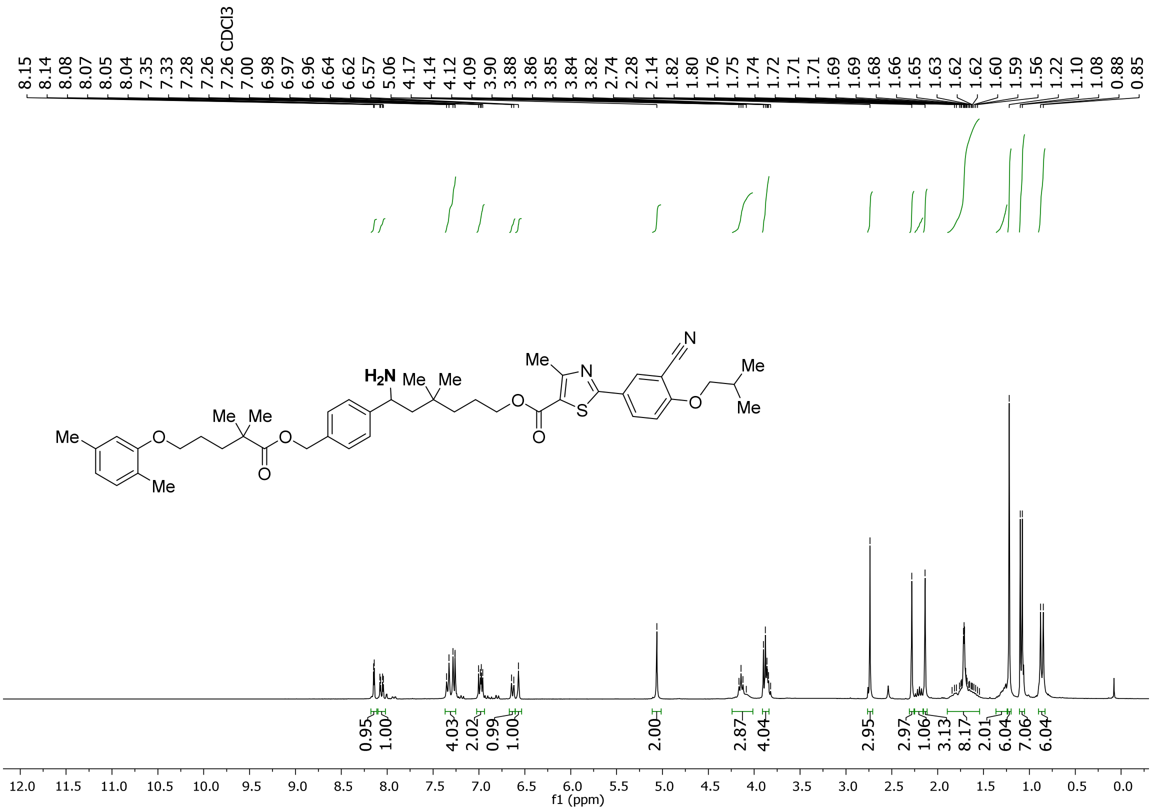

^{1}H NMR (300 MHz, $CDCl_3$) of compound **5**.

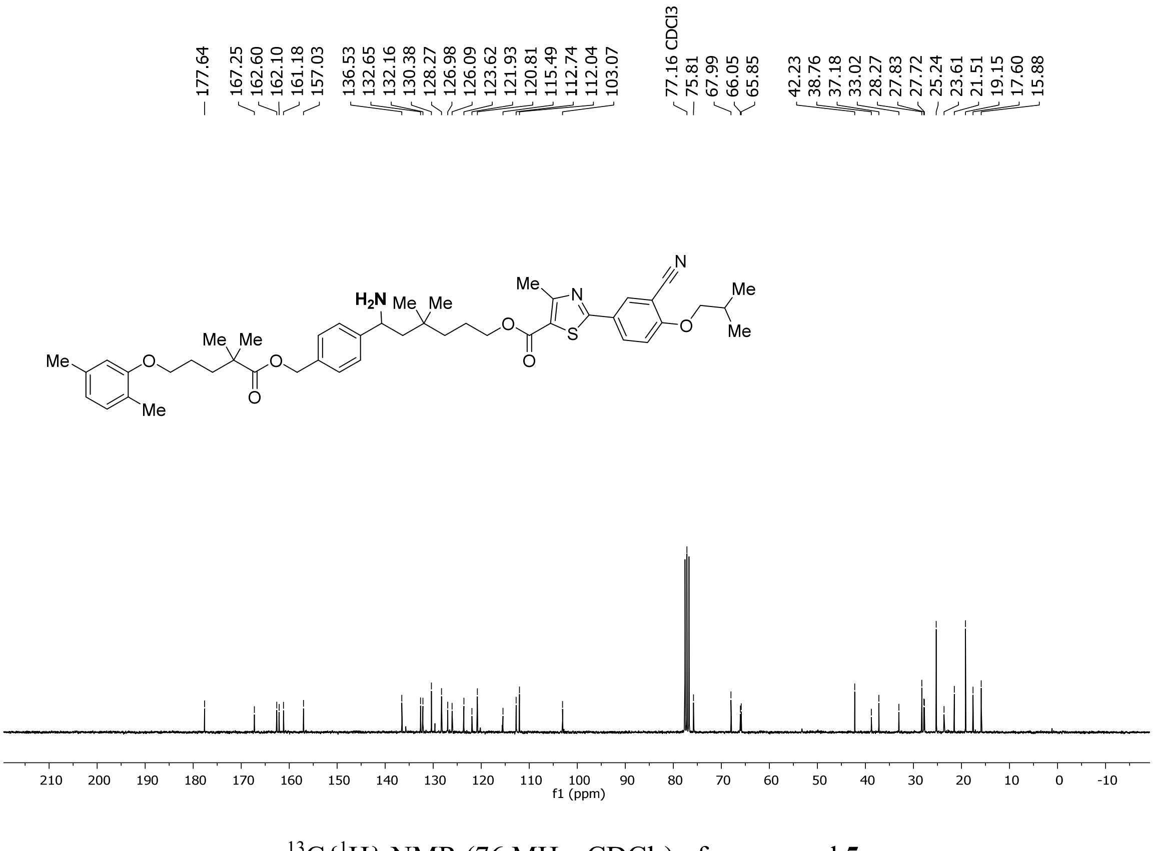

^{13}C{^{1}H} NMR (76 MHz, $CDCl_3$) of compound **5.**

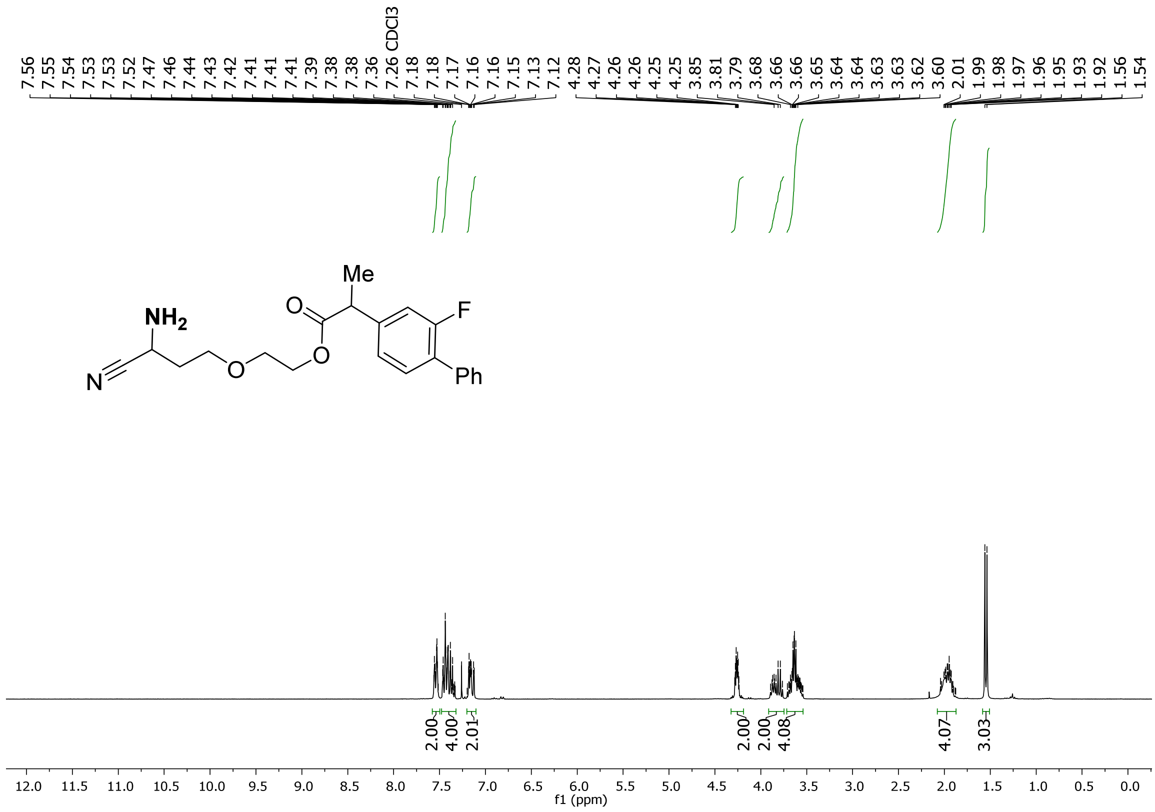

^{1}H NMR (300 MHz, $CDCl_3$) of compound **6**.

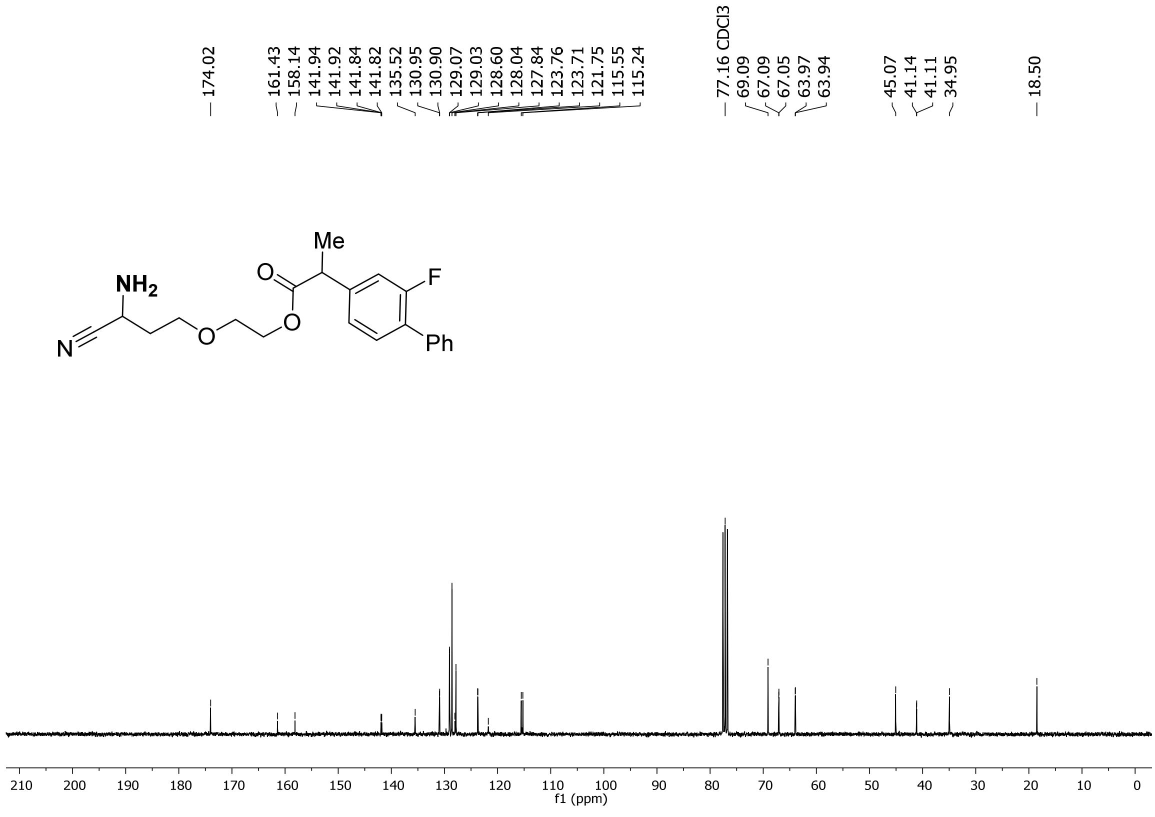

^{13}C{^{1}H} NMR (76 MHz, $CDCl_3$) of compound **6.**

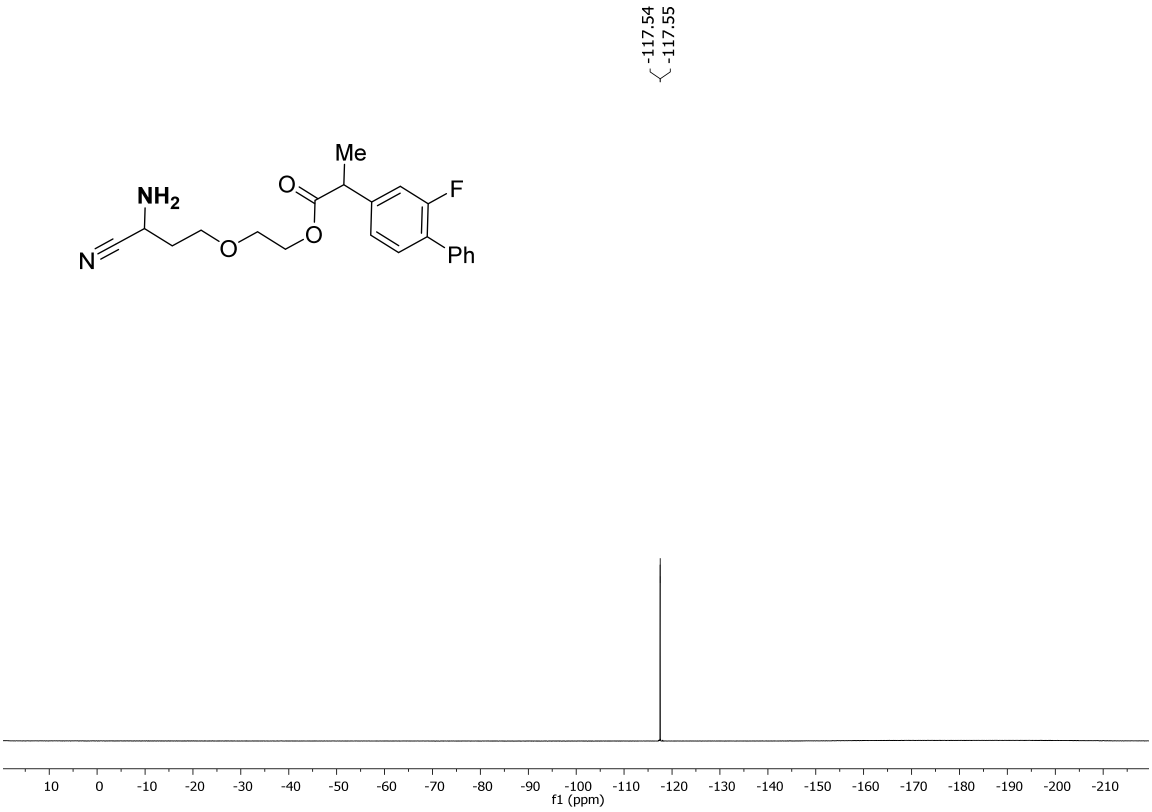

$^{19}F\{^{1}H\}$ NMR (282 MHz, $CDCl_3$) of compound **6.**

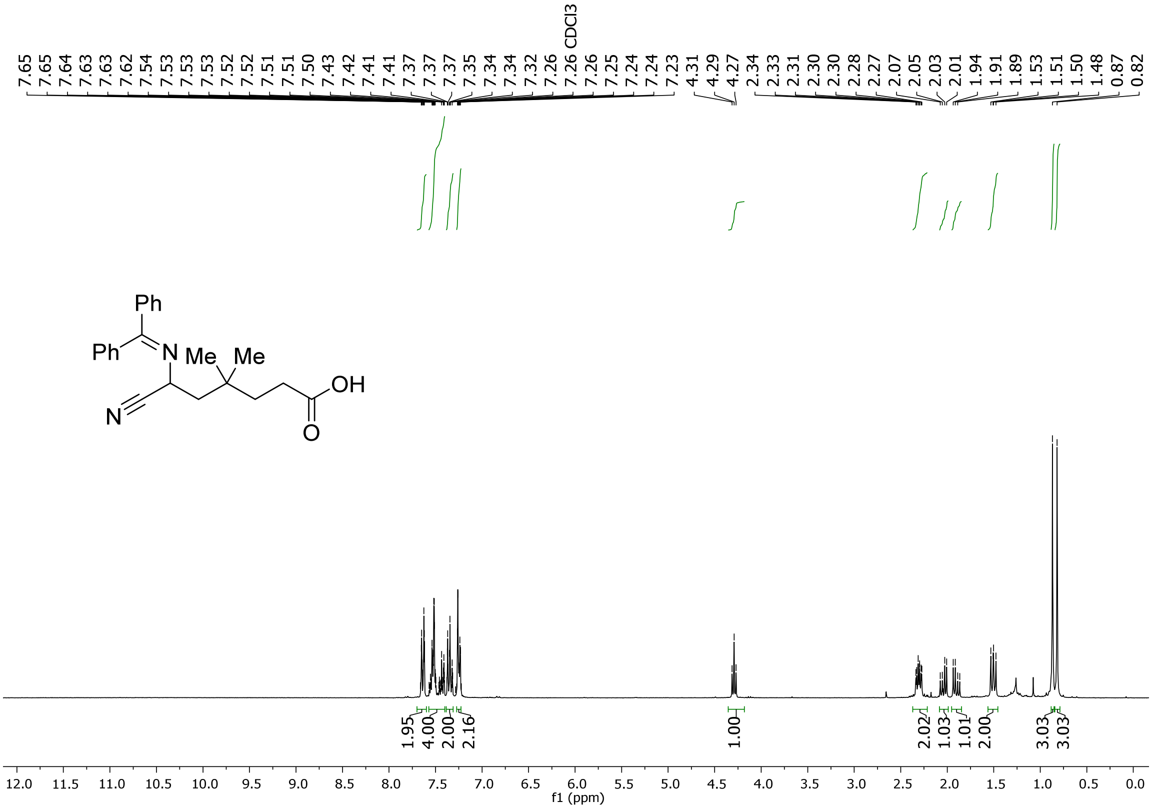

^{1}H NMR (300 MHz, $CDCl_3$) of compound **7**.

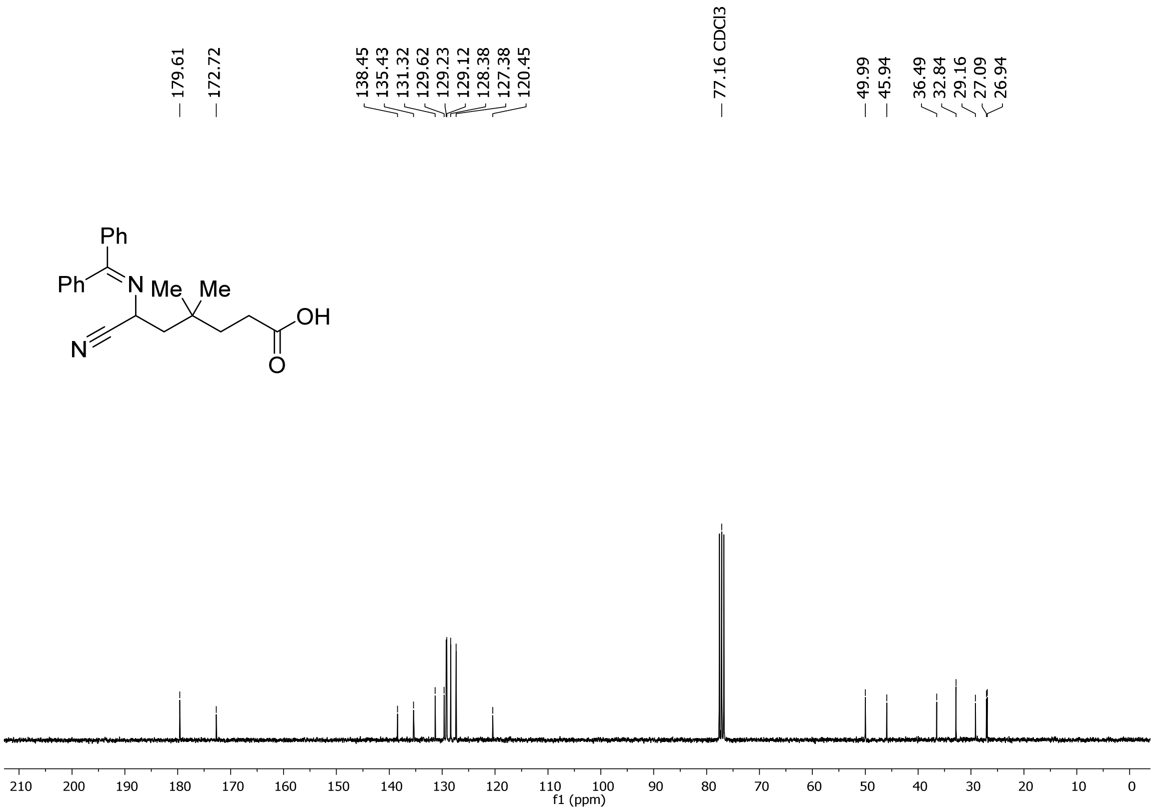

$^{13}C\{^1H\}$ NMR (76 MHz, $CDCl_3$) of compound **7.**
